# Identification of Hypertension in Electronic Health Records Through Computable Phenotype Development and Validation for Use in Public Health Surveillance: Retrospective Study

**DOI:** 10.2196/46413

**Published:** 2023-12-27

**Authors:** Nimish Valvi, Timothy McFarlane, Katie S Allen, P Joseph Gibson, Brian Edward Dixon

**Affiliations:** 1 Center for Biomedical Informatics Regenstrief Institute Indianapolis, IN United States; 2 Department of Nutrition and Health Science College of Health Ball State University Muncie, IN United States; 3 Indiana Family and Social Services Administration Indianapolis, IN United States; 4 Department of Health Policy & Management Fairbanks School of Public Health Indiana University Indianapolis, IN United States; 5 CDC Foundation Atlanta, GA United States

**Keywords:** computable phenotypes, electronic health records, health information exchange, hypertension, population surveillance, public health informatics

## Abstract

**Background:**

Electronic health record (EHR) systems are widely used in the United States to document care delivery and outcomes. Health information exchange (HIE) networks, which integrate EHR data from the various health care providers treating patients, are increasingly used to analyze population-level data. Existing methods for population health surveillance of essential hypertension by public health authorities may be complemented using EHR data from HIE networks to characterize disease burden at the community level.

**Objective:**

We aimed to derive and validate computable phenotypes (CPs) to estimate hypertension prevalence for population-based surveillance using an HIE network.

**Methods:**

Using existing data available from an HIE network, we developed 6 candidate CPs for essential (primary) hypertension in an adult population from a medium-sized Midwestern metropolitan area in the United States. A total of 2 independent clinician reviewers validated the phenotypes through a manual chart review of 150 randomly selected patient records. We assessed the precision of CPs by calculating sensitivity, specificity, positive predictive value (PPV), *F*_1_-score, and validity of chart reviews using prevalence-adjusted bias-adjusted κ. We further used the most balanced CP to estimate the prevalence of hypertension in the population.

**Results:**

Among a cohort of 548,232 adults, 6 CPs produced PPVs ranging from 71% (95% CI 64.3%-76.9%) to 95.7% (95% CI 84.9%-98.9%). The *F*_1_-score ranged from 0.40 to 0.91. The prevalence-adjusted bias-adjusted κ revealed a high percentage agreement of 0.88 for hypertension. Similarly, interrater agreement for individual phenotype determination demonstrated substantial agreement (range 0.70-0.88) for all 6 phenotypes examined. A phenotype based solely on diagnostic codes possessed reasonable performance (*F*_1_-score=0.63; PPV=95.1%) but was imbalanced with low sensitivity (47.6%). The most balanced phenotype (*F*_1_-score=0.91; PPV=83.5%) included diagnosis, blood pressure measurements, and medications and identified 210,764 (38.4%) individuals with hypertension during the study period (2014-2015).

**Conclusions:**

We identified several high-performing phenotypes to identify essential hypertension prevalence for local public health surveillance using EHR data. Given the increasing availability of EHR systems in the United States and other nations, leveraging EHR data has the potential to enhance surveillance of chronic disease in health systems and communities. Yet given variability in performance, public health authorities will need to decide whether to seek optimal balance or declare a preference for algorithms that lean toward sensitivity or specificity to estimate population prevalence of disease.

## Introduction

Hypertension is the most prevalent risk factor for mortality throughout the world, and it was reported as a primary or contributing cause of death for over 500,000 Americans in 2019 [1]. Moreover, hypertension is reported as a comorbid condition for nearly 70% of individuals who have their first myocardial infarction and almost 80% of those who have their first stroke [2]. Overall, approximately 1 out of 3 adults in the United States is diagnosed with hypertension, which translates to almost 75 million Americans [3]. Moreover, given recent changes to guidelines for the prevention, detection, evaluation, and management of hypertension, the number of Americans considered to have this condition is expected to increase in the future [4].

The surveillance of conditions such as hypertension is a cornerstone of public health practice [5], yet quantifying the prevalence of hypertension at granular person, place, and time levels remains a challenge for local health departments (LHDs). Existing methods for capturing prevalence data for chronic disease within a geographic area rely upon community-based surveys. The 2 surveys most widely used by LHDs are the Behavioral Risk Factor Surveillance System and the National Health and Nutrition Examination Survey [6,7]. Those surveys provide reliable hypertension estimates at state and national levels, respectively, but have important limitations in their timeliness, breadth, and cost [8]. Moreover, these surveys provide very imprecise disease estimates at the local level (eg, county and neighborhood), where most public health interventions occur.

In addition to the Behavioral Risk Factor Surveillance System and the National Health and Nutrition Examination Survey, some LHDs conduct their own community surveys of health and health behaviors. While community surveys may be representative of the local population and powered for granular estimation, they can be costly to perform and are often spaced years apart [9]. Furthermore, community surveys typically do not include any medical, dental, or physiological measurements, limiting their ability to reliably measure true disease states as well as adherence to clinical guidelines, such as proper management of diabetes, hypertension, and other chronic illnesses.

Given the limitations of existing methods, LHDs seek alternative methods for obtaining timely information on health behaviors and risk factors prevalent in their community. Since the passing of the Health Information Technology for Economic and Clinical Health Act of 2009, electronic health records (EHR) systems have become more common in the United States [10], representing a potential source of chronic disease surveillance data among those seeking health care.

Over 70% of ambulatory providers in the United States have adopted EHR systems [10]. As health care systems increasingly capture data from routine health care visits in EHR systems, national initiatives, including the digital Learning Health System of the National Academy of Medicine, the Robert Wood Johnson Foundation’s Data for Health, and the Multi-State EHR-Based Network for Disease Surveillance [11], aim to leverage such data to improve the delivery of health care and community health outcomes [12]. The hope is that by leveraging existing digital data sources, public health agencies may access more timely and complete information to better assess and improve health in their communities.

Previous studies leveraged EHR data from primary care information systems funded by public health agencies but did not capture EHR data from other systems [13]. To explore the use of data combined from multiple EHR systems covering a large population in a community, we sought to develop and validate hypertension computable phenotypes (CPs) using EHR data available through a community-based health information exchange (HIE) network. We further sought to assess the HIE network as a source for accurate estimates of hypertension prevalence for a geographically defined population. HIE networks are increasingly available in many jurisdictions and nations [14], potentially providing public health authorities with a mechanism to access multiple electronic records for the same patient from a wide array of hospitals, physician practices, and other health care organizations that account for a representative subset of the population.

To assess the measurement of hypertension prevalence using an HIE network for an LHD population, we empirically derived and examined 6 hypertension CPs using data extracted from multiple EHR systems deployed across 3 distinct, large integrated delivery networks operating in Marion County, Indiana. This paper reports the prevalence rates derived from 6 hypertension phenotypes as well as the performance of each CP compared to a human chart review.

## Methods

### Data Source

The Indiana Network for Patient Care (INPC), launched in the 1990s, is one of the largest interorganizational clinical data repository in the United States, with more than 10 billion clinical data elements [15]. The INPC serves as the primary platform for the Indiana Health Information Exchange, which connects more than 100 hospitals, 14,000 practices, and nearly 40,000 providers. Each participating institution submits clinical data from its EHR system to a centralized repository (the INPC) managed by the Indiana Health Information Exchange. Incoming data are matched to existing patients using an enterprise master person index (eg, master patient index or client registry), and clinical concepts (eg, laboratory results or blood pressure readings) are mapped to standardized terminologies for storage in the common data repository structure [16].

The INPC captures information on 99% of the population of Marion County, Indiana, which is home to Indianapolis. According to the 2020 census [17], Marion County had a resident population of 971,102 with a racial composition of 62.4% White, 29.6% Black or African American, and 11.3% Hispanic; 51.6% female; and 13.1% adults aged 65 years or older.

For this study, a subset of 3 health systems was used, representing at least 80% (776,882/971,102) of the population of Marion County. The first is an essential health provider consisting of a large tertiary hospital and 10 federally qualified health centers geographically spread across the county. The second is the state’s largest health system, with 3 tertiary hospitals in Marion County, including the region’s largest children’s hospital, a level 1 trauma center, and the state’s largest cancer center. The final system is a regional health system that includes another large children’s hospital, a tertiary hospital, and a large network of primary care and neighborhood emergency departments across the county. Each health system independently represents a large portion of Marion County residents based on available health market share data, and each included system-approved participation in the study.

### Study Population

To examine CPs for hypertension, we extracted a cohort of all adults (at least 18 years of age as of January 1, 2014) living in Marion County (all patient addresses in INPC are geocoded [18]) who sought care at 1 of the 3 large integrated delivery networks between January 1, 2014, and December 31, 2015.

### CPs for Essential (Primary) Hypertension

There is no standard CP for defining essential (primary) hypertension based on clinically derived data. To that effect, a central goal of this work was to propose and test EHR-based phenotypes of essential hypertension. These algorithms were developed using available definitions for essential hypertension from the US Centers for Disease Control and Prevention (CDC) as well as the American Heart Association (AHA) [19] along with input from epidemiologists and medical doctors. We examined a range of phenotype definitions (Table 1) because evidence of hypertension exists in several places across a patient’s EHR, with data contributed from multiple health systems. All included data elements were required to be within the 2014-2015 period specified for this analysis. While many clinicians document hypertension through routine billing using International Classification of Diseases (ICD) codes, previous studies found this kind of documentation to be incomplete. In addition to clinical diagnoses, we incorporated physiologic blood pressure readings for some phenotypes, given that the CDC and the US Preventative Services Taskforce [19,20] recommend screening for hypertension using office-based measurements. When using blood pressure readings, hypertension was characterized by systolic blood pressure measurements of at least 140 mm Hg or diastolic blood pressure measurements of at least 90 mm Hg based on guidelines published before 2017 by the American College of Cardiology and the AHA [3]. However, blood pressure recordings were not available for all individuals in the population. Furthermore, a meta-analysis revealed major accuracy limitations, including misdiagnosis for office-based blood pressure measurements [21]. In addition, previous studies reported that medication data can be useful when identifying individuals with chronic illness, as they are typically on maintenance medication [22-24]. We therefore examined 1 phenotype that included pharmacy data downloaded from a national repository [25] of claims data. The list of hypertension medications used was originally created by pharmacist health services researchers at the Regenstrief Institute (in Multimedia Appendix 1).

**Table 1:** Computable phenotype definitions used to examine hypertension prevalence among adults in Marion County, Indiana (2014-2015).

Computable phenotype	Definition	Data examined
1	Individual has at least 1 hypertension ICD-9-CM^a^ or ICD-10-CM^b^ diagnostic code documented for at least inpatient or outpatient encounter (≥1 hypertension diagnostic code)	Primary diagnosis as well as secondary diagnoses or comorbidities associated with a clinical encounter documented in the patient’s EHR^c^
2	Individual has at least 1 BP^d^ measurement in which the systolic BP was at least 140 mm Hg or diastolic BP was at least 90 mm Hg (≥1 BP reading)	Point-of-care- and laboratory-based physiologic measurements recorded in the patient’s EHR
3	Individual has at least 2 BP measurements in which the systolic BP was at least 140 mm Hg or diastolic BP was at least 90 mm Hg (≥2 BP readings)	Point-of-care- and laboratory-based physiologic measurements recorded in the patient’s EHR
4	Individual has at least 1 BP measurement in which the systolic BP was at least 140 mm Hg or diastolic was at least 90 mm Hg (≥1 BP reading), and individual has at least 1 hypertension ICD-9-CM or ICD-10-CM diagnostic code documented for at least inpatient or outpatient encounter (≥1 hypertension diagnostic code)	Primary diagnosis as well as secondary diagnoses or comorbidities, along with point-of-care-based physiologic measurements recorded in the patient’s EHR
5	Individual has at least 1 BP measurement in which the systolic BP was at least 140 mm Hg or diastolic BP was at least 90 mm Hg (≥ 1 BP reading), and individual has at least 2 hypertension ICD-9-CM or ICD-10-CM diagnostic codes documented for 2 different inpatient or outpatient encounters (≥2 hypertension diagnostic codes)	Primary diagnosis as well as secondary diagnoses or comorbidities, along with point-of-care- and laboratory-based physiologic measurements recorded in the patient’s EHR
6	Individual has at least 1 BP measurement in which the systolic BP was at least 140 mm Hg or diastolic BP was at least 90 mm Hg (≥1 BP reading), individual has at least 1 hypertension ICD-9-CM or ICD-10-CM diagnostic code documented for at least inpatient or outpatient encounter (≥1 hypertension diagnostic code), or individual has filled at least 1 prescription for any medication associated with hypertension (≥1 hypertension medication)	Primary diagnosis as well as secondary diagnoses or comorbidities, along with point-of-care- and laboratory-based physiologic measurements recorded in the patient’s EHR and pharmacy claims records for filled prescriptions downloaded from a national repository

^a^ICD-9-CM: International Classification of Disease-9 Clinical Modification.

^b^ICD-10-CM: International Classification of Disease-10 Clinical Modification.

^c^EHR: electronic health record.

^d^BP: blood pressure.

### Validation of CPs and Chart Review

To validate the phenotypes, we used a chart review using a sample of 299 individuals randomly selected from the population. A retired outpatient cardiovascular nurse with more than 40 years of service reviewed the merged, longitudinal EHR for patients in the sample using the INPC. In the chart review, based on clinical judgment, it was noted whether the patient had hypertension during the study period based on the recorded data in the chart. Chart reviewers had access to the full clinical record contained within the HIE, which included clinical text as well as structured data. In addition, the reviewer documented any evidence supporting the clinical assessment of hypertension. The phenotype definitions were applied to the 299 individuals in the random sample to examine their performance. Phenotype performance was quantified using sensitivity, specificity, and positive predictive value (PPV), including the 95% CIs for each measure, and the *F*_1_-score.

These phenotypes were then applied to the entire population extracted from the INPC to estimate hypertension prevalence for Marion County, Indiana. Prevalence rates were calculated by age, sex, race, and ethnicity.

A blinded chart review was conducted on patient records at the Regenstrief Institute. The chart review was conducted independently, and the 2 reviewers were trained before the review. A total of 299 charts were randomly selected from the INPC, of which 233 were reviewed by reviewer 1. To calculate interrater reliability, we compared the hypertension classifications from 150 charts reviewed by a second reviewer, another outpatient nurse with 20 years of experience, with those of the first reviewer.

The standard measure of agreement is Cohen κ coefficient (κ). It adjusts the observed agreement by the agreement expected by chance. However, if no further adjustments are made, κ can be deceptive because it is sensitive to both the bias in reporting “yes” to the classification of hypertension, if any exists, and the prevalence of “yes” relative to “no” in the sample. We therefore calculated the prevalence-adjusted bias-adjusted κ (PABAK) [26-28] for the case definition and the phenotypes. PABAK values were interpreted according to the guidelines for κ provided by Landis and Koch [29]: 0.81-1.00=almost perfect agreement; 0.61-0.80=substantial agreement; 0.41-0.60=moderate agreement; 0.21-0.40=fair agreement; and 0.01-0.20=slight agreement.

All analyses and phenotype coding were conducted using SAS (version 9.4; SAS Institute Inc).

### Ethical Considerations

Study approval was obtained from the Indiana University Institutional Review Board (exempt protocol 1701925087). Informed consent was waived due to the retrospective use of preexisting, deidentified data from medical records.

Data managers at the Regenstrief Institute extracted EHR data from the 3 health systems using the INPC clinical data repository. Data from a third health system were extracted by analysts at the health system, then linked and merged with the INPC data as this health system does not contribute ambulatory clinic data to the INPC repository. A total of 6 CPs were derived from the data (Table 1), and prevalence was calculated by dividing the number of individuals meeting each essential hypertension case definition by the total cohort population.

## Results

### Population Characteristics

Data were extracted from the INPC from January 1, 2014, to December 31, 2015, and included 548,232 adults aged 18 years or older from the 3 health systems within Marion County. The majority of people were White (308,213/548,232, 56.2%), female (335,548/548,232, 61.2%), and adults aged 65 years or older (93,513/548,232, 17.1%; Table 2).

**Table 2:** Study characteristics and demographics using electronic health records from the Indiana Network for Patient Care, 2014-2015 (N=548,232).

Characteristic			Overall (N=548,232)		Hypertension		
					No (n=337,468)		Yes (n=210,764)
**Age (years), n (%)**							
	18-39	214,655 (39.1)		161,878 (75.4)		52,777 (24.6)	
	40-64	240,064 (43.8)		138,648 (57.8)		101,416 (42.2)	
	≥65	93,513 (17.1)		36,942 (39.5)		56,571 (60.5)	
**Sex, n (%)**							
	Female	335,548 (61.2)		214,241 (63.8)		121,307 (36.2)	
	Male	212,684 (38.8)		123,227 (57.9)		89,457 (42.1)	
**Race, n (%)**							
	White	308,213 (56.2)		187,381 (60.8)		120,832 (39.2)	
	Black	148,117 (27)		78,057 (52.7)		70,060 (47.3)	
	Other	91,902 (16.8)		72,030 (78.4)		19,872 (21.6)	

### CPs using INPC

A total of 299 records were randomly drawn from the INPC. The chart review was completed for 233 records by reviewer 1 (Table 3). According to chart reviewer 1 expert opinion, 167 individuals were identified with hypertension, a prevalence of 71.7%. Of the 167 individuals with hypertension, the phenotype algorithms identified 82 (CP1), 107 (CP2), 55 (CP3), 47 (CP4), 142 (CP5), and 200 (CP6), respectively. The final CP (CP6) was determined to have a sensitivity of 99.9% (95% CI 97.8%-100.0%) with a PPV of 83.5% (95% CI 79.9%-86.6%). However, CP6 had a comparatively lower specificity of 50% (95% CI 37.4%-62.6%) compared to CP1, with a specificity of 93.9% (95% CI 85.2%-98.3%). For CP6, the *F*_1_-score was closest to 1.0 at 0.91, suggesting that this algorithm has the most balanced performance across error types. Using the *F*_1_-score as an evaluation metric, the next best-performing algorithm is CP5 (0.71), followed by CP1 (0.63).

**Table 3:** Performance of computable phenotypes (CPs) for hypertension validated using randomly selected electronic health records from the Indiana Network for Patient Care (n=233). The prevalence estimate on manual chart review (reviewer 1) was determined to be 71.7% (n=167).

Phenotypes	Total confirmed hypertension cases, n	Prevalence, n (%)	Sensitivity, % (95% CI)	Specificity, % (95% CI)	Positive predictive value, % (95% CI)	*F*_1_-score
≥1 Clinical diagnosis (CP1)	78	82 (35.2)	46.7 (38.9-54.6)	93.9 (85.2-98.3)	95.1 (88.1-98.0)	0.63
≥1 Vitals indicated (CP2)	76	107 (45.9)	45.5 (37.8-53.4)	53.0 (40.3-65.4)	71.0 (64.3-76.9)	0.55
≥2 Vitals indicated (CP3)	44	55 (23.6)	26.3 (19.8-33.7)	83.3 (72.1-91.4)	80.0 (68.8-87.9)	0.40
≥1 Clinical diagnosis and ≥1 vitals indicated (CP4)	45	47 (20.2)	26.9 (20.4-34.3)	97.0 (89.5-99.6)	95.7 (84.9-98.9)	0.42
≥1 Clinical diagnosis or ≥1 vitals indicated (CP5)	109	142 (60.9)	65.3 (57.5-72.5)	50.0 (37.4-62.6)	76.7 (71.7-81.2)	0.71
≥1 Clinical diagnosis, ≥1 vitals indicated, or ≥1 medications indicated (CP6)	167	200 (85.8)	99.9 (97.8-100.0)	50.0 (37.4-62.6)	83.5 (79.9-86.6)	0.91

### Interrater Reliability

We assessed interrater reliability between the 2 raters using PABAK. A total of 150 charts were reviewed by both reviewers out of the 233 charts that reviewer 1 examined. The interrater agreement (PABAK=0.91) was an almost perfect agreement for the 2 reviewers. The estimated PABAK (Table 4) for the phenotypes ranged from substantial (0.70) to almost perfect agreement (0.88).

**Table 4:** Hypertension phenotypes agreement between 2 reviewers and prevalence-adjusted bias-adjusted κ (PABAK; n=150).

Phenotype	HTN^a^, n	HTN prevalence estimate, % (95% CI)	PABAK^b^
HTN case determination	101	67.3 (59.2-74.8)	0.91
≥1 Clinical diagnosis (CP1^c^)	35	23.3 (16.8-30.9)	0.70
≥1 Vitals indicated overall (CP2)	55	36.7 (28.9-44.9)	0.73
≥2 Vitals indicated (CP3)	29	19.3 (13.3-26.6)	0.81
≥1 Clinical diagnosis and ≥1 vitals indicated (CP4)	20	13.3 (8.3-19.8)	0.76
≥1 Clinical diagnosis or ≥1 vitals indicated (CP5)	71	47.3 (39.1-55.6)	0.71
≥1 Clinical diagnosis, ≥1 vitals indicated, or ≥1 medications indicated (CP6)	117	78.0 (70.5-84.3)	0.88

^a^HTN: hypertension.

^b^PABAK = 2 × ([positive agreement + negative agreement]/N) – 1.

^c^CP: computable phenotype.

In Table 2, the overall hypertension prevalence using CP6 during the study period was estimated at (210,764/548,232, 38.4%). The prevalence of hypertension was highest among adults aged 65 years or older (56,571/95,513, 60.5%), female individuals (121,307/335,548, 36.2%), and Black individuals (70,060/148,117, 47.3%) during the study period.

## Discussion

### Principal Findings

We developed and evaluated 6 CPs using a combination of ICD-9 and 10 codes, medications, and vitals for essential hypertension using EHR data extracted from multiple health systems. Despite variation in CP performance, most CPs possessed PPVs above 75%, making these CPs reasonable for use in public health surveillance. Unlike in controlled trials or other research contexts in which investigators seek to maximize specificity (eg, the ability to identify individuals without disease), public health surveillance seeks to balance specificity with sensitivity (eg, the ability to identify all cases in the population). In this study, CP1, having good specificity, might serve as an initial screening for selecting individuals who might benefit from more targeted interventions.

### Comparison With Previous Work

Our findings align with previous work examining the performance of approaches that rely primarily on diagnostic codes compared to other CP approaches. Relying solely on diagnostic codes (CP1) resulted in low sensitivity (46.7%) yet high specificity (93.9%) and PPV (95.1%) to identify individuals with hypertension. This performance is quite similar to the use of ICD codes for identifying cases of sexually transmitted infections, which also possess high specificity (99.9%) and reasonable PPV (≥85%) yet low sensitivity (<15%) [30]. The imbalance is also reflected in its *F*_1_-score of 0.63, which is adequate but may not be sufficient for public health surveillance, where most epidemiologists prefer to err on the side of sensitivity in case detection.

The broadest phenotype (CP6) that allowed the identification of individuals on antihypertensive therapies along with clinical diagnoses and blood pressure readings possessed high sensitivity (99.9%) but lower specificity (50%) and PPV (83.5%). However, the *F*_1_-score (0.91) suggests CP6 as the best algorithm for overall balance. It is worth noting that one potential reason for the low specificity could be due to the treatment of hypertension, resulting in a reduced blood pressure measurement during the window of observation. This would indicate individuals with hypertension who meet the CP definition based on medication but may not have evidence of hypertension as a reason for a visit in the clinical text or abnormal blood pressure readings during the observation window. This and other indications for hypertensive classification could be explored further in future studies.

Multiple strategies to identify the prevalence of chronic diseases have been used previously, either from a single hospital or within a network-based EHR system [31-35]. Our hypertension phenotype based on diagnostic codes alone (CP1) was much lower in sensitivity (46.7%) compared to previous studies from the United States (83%) [36], Canada (85%) [34], and Switzerland (83%) [35]. A likely explanation for this is that the previous studies had participants mainly from primary-care centers, while this study used data from both outpatient and hospitalization encounters. Furthermore, many small primary care practices do not contribute data to the INPC, limiting data capture in some parts of the metropolitan area. When additional sources of evidence of hypertension were included in the phenotype, the sensitivity increased. However, after including additional sources, the specificity was reduced to 50%. The increase in sensitivity after including medications can partly be explained by a study [37] that found that individuals with an appropriate diagnosis of hypertension were more likely to be treated (92.6%). Population prevalence (38.4%) calculated using CP6 was much higher compared to a diverse patient population in a California [37] health system (28.7%), which used a similar definition of hypertension [38]. However, this population prevalence was comparable to a study from New York (38.4% vs 39.2%) [36], with a large distributed EHR network having a similar time period.

Increasing the availability of standardized data within EHR-based surveillance systems, including the adoption and use of the US Core Data for Interoperability (USCDI), can further improve the accuracy and completeness of CPs to estimate the prevalence of hypertension. EHR-based diagnoses capture an individual’s state or disease status; however, they are based on the interpretation of the clinical staff treating the patient. Using secondary data for estimating prevalence, public health professionals often do not have the power to enforce standardization of data capture from the originating health systems [39]. Further studies should explore the comparison of prevalence from EHR-based surveillance systems with that of standardized, representative [40] data captured through other methods. With the Office of the National Coordinator for Health Information Technology requiring implementation of USCDI, all of the CPs used in this study could be implemented through the Fast Healthcare Interoperability Resources interfaces throughout the United States in all certified EHR systems updated after December 31, 2022.

The data for this study were extracted from 3 different health systems that participate in the INPC, managed by the Indiana HIE. Leveraging HIE infrastructures to aggregate data across health care facilities for public health purposes is increasingly viewed as critical in the wake of the COVID-19 pandemic [41]. As discussed during the 2022 American College of Medical Informatics Symposium, many HIE networks are working to become health data utilities that serve as information infrastructures in support of public health [42]. Unfortunately, few states have robust HIE infrastructures such as that of the INPC. Yet, there is hope that policies such as TEFCA (Trusted Exchange Framework and Common Agreement) [43] and the emphasis by the Office of the National Coordinator for Health Information Technology can spur to creation and expansion of HIE infrastructures across the nation [44]. This will remain a key area for research and development as public health seeks to modernize its data infrastructure and HIE networks expand their support for population health. The work presented here is just the beginning of efforts to better support public health surveillance through the EHR and HIE systems.

### Limitations

We acknowledge the limitations of using EHR data, some of which are due to the administrative nature that can produce misclassification (ie, coding errors or missed fields). Additionally, in-care adult populations are more likely to be female, older, non-Hispanic, and insured compared to the not-in-care adult population [45]. In comparison to the demographic distribution in the general population, there was a higher representation of women and older adults in our EHR cohort. This could either underestimate hypertension prevalence for younger age groups and for male individuals or overestimate for female individuals and older adults in our population. Data obtained from EHR-HIE systems can have variable quality since they are obtained from diverse health delivery systems due to differences in documentation processes across providers and clinicians. For instance, some practices may record information only from standardized fields, while others may capture values using free-text fields. All 3 health systems included in this study use a commercial EHR system. Once clinical encounter data are submitted from the EHR to the HIE, they undergo quality control steps to normalize data to the extent possible, including standardization of terminology. Additionally, the phenotypes did not leverage unstructured data, which may have resulted in missing data, depending on how the institutions store the elements required for the phenotype. We did anticipate some data quality issues such as missingness or data inaccuracies, but this limitation is applicable to a minority of cases [46,47]. We further note that the *F*_1_-measure, which is widely used in information retrieval, is calculated based on precision and recall, and it does not account for accuracy (eg, true negatives), which means it may not be an ideal measure for performance in cases involving clinical diagnosis [48]. Lastly, we used both ICD-9 Clinical Modification and ICD-10 Clinical Modification codes to identify this study population, yet we could not account for performance differences between the coding systems.

Despite limitations, using EHR-based prevalence estimates for population health has several benefits. They provide larger sample sizes while affording granular person, place, and time that are unavailable from existing population-based self-reported surveys. The data are more timely and more affordable than locally commissioned surveys. Furthermore, the collection and tabulation of estimates can be done more frequently and require less human effort than traditional approaches. This would enable resource-limited LHDs to routinely assess chronic disease burden and trend data over time.

### Conclusions

We constructed 6 CPs to estimate the prevalence of hypertension in support of public health surveillance for chronic disease. With the help of manual chart reviews, we were able to capture the variation between phenotypes. In the future, we plan to use these phenotypes to compare prevalence with estimates from population-based health surveys. EHR-based estimates for chronic illnesses are helpful for public health surveillance and regional quality improvement efforts at much lower costs compared to traditional population survey approaches. As the adoption and use of HIE systems and standards such as the USCDI increase, the quality of population health metrics should improve over time.

## Appendix

Multimedia Appendix 1 List of hypertension medications using National Drug Codes.

## Abbreviations

AHA: American Heart Association
CDC: US Centers for Disease Control and Prevention
CP: computable phenotype
EHR: electronic health record
HIE: health information exchange
ICD: International Classification of Diseases
INPC: Indiana Network for Patient Care
LHD: local health department
PABAK: prevalence-adjusted bias-adjusted κ
PPV: positive predictive value
TEFCA: Trusted Exchange Framework and Common Agreement
USCDI: US Core Data for Interoperability

## Data Availability

The data sets generated and analyzed during this study are not publicly available due to privacy and governance related issues but are available from the corresponding author on reasonable request and with appropriate governance.
