# Supplementary material for: Identification of Hypertension in Electronic Health Records Through Computable Phenotype Development and Validation for Use in Public Health Surveillance: Retrospective Study
Source: JMIR Form Res. 2023 Dec 27;7:e46413. doi: 10.2196/46413 (PMC10782284; doi:10.2196/46413)
Supplement: Multimedia Appendix 1 [file formative_v7i1e46413_app1.docx]

|  |  |  |
| --- | --- | --- |
| **Multimedia Appendix 1. List of hypertension medications using National Drug Codes (NDC)** | | |
| NDC_CODE | NAME | GPI_GENERIC_NAME |
| 00078048661 | Aliskiren Fumarate | Aliskiren Fumarate Tab 300 MG (Base Equivalent) |
| 54868577200 | Aliskiren Fumarate | Aliskiren Fumarate Tab 150 MG (Base Equivalent) |
| 54569627400 | Aliskiren Fumarate | Aliskiren Fumarate Tab 300 MG (Base Equivalent) |
| 54569627300 | Aliskiren Fumarate | Aliskiren Fumarate Tab 150 MG (Base Equivalent) |
| 00078048615 | Aliskiren Fumarate | Aliskiren Fumarate Tab 300 MG (Base Equivalent) |
| 00078048515 | Aliskiren Fumarate | Aliskiren Fumarate Tab 150 MG (Base Equivalent) |
| 35356013330 | Aliskiren Fumarate | Aliskiren Fumarate Tab 150 MG (Base Equivalent) |
| 12280040030 | Aliskiren Fumarate | Aliskiren Fumarate Tab 150 MG (Base Equivalent) |
| 00078048635 | Aliskiren Fumarate | Aliskiren Fumarate Tab 300 MG (Base Equivalent) |
| 00078048535 | Aliskiren Fumarate | Aliskiren Fumarate Tab 150 MG (Base Equivalent) |
| 54868604200 | Aliskiren Fumarate | Aliskiren Fumarate Tab 300 MG (Base Equivalent) |
| 00078048561 | Aliskiren Fumarate | Aliskiren Fumarate Tab 150 MG (Base Equivalent) |
| 00078060615 | Aliskiren-Amlodipine | Aliskiren-Amlodipine Tab 300-10 MG |
| 00078060315 | Aliskiren-Amlodipine | Aliskiren-Amlodipine Tab 150-5 MG |
| 00078060415 | Aliskiren-Amlodipine | Aliskiren-Amlodipine Tab 150-10 MG |
| 00078060515 | Aliskiren-Amlodipine | Aliskiren-Amlodipine Tab 300-5 MG |
| 00078061115 | Aliskiren-Amlodipine-Hydrochlorothiazide | Aliskiren-Amlodipine-Hydrochlorothiazide Tab 300-5-12.5 MG |
| 00078061015 | Aliskiren-Amlodipine-Hydrochlorothiazide | Aliskiren-Amlodipine-Hydrochlorothiazide Tab 150-5-12.5 MG |
| 00078061215 | Aliskiren-Amlodipine-Hydrochlorothiazide | Aliskiren-Amlodipine-Hydrochlorothiazide Tab 300-5-25 MG |
| 00078061315 | Aliskiren-Amlodipine-Hydrochlorothiazide | Aliskiren-Amlodipine-Hydrochlorothiazide Tab 300-10-12.5 MG |
| 00078061415 | Aliskiren-Amlodipine-Hydrochlorothiazide | Aliskiren-Amlodipine-Hydrochlorothiazide Tab 300-10-25 MG |
| 00078052315 | Aliskiren-Hydrochlorothiazide | Aliskiren-Hydrochlorothiazide Tab 300-12.5 MG |
| 54868604100 | Aliskiren-Hydrochlorothiazide | Aliskiren-Hydrochlorothiazide Tab 150-12.5 MG |
| 00078052215 | Aliskiren-Hydrochlorothiazide | Aliskiren-Hydrochlorothiazide Tab 150-25 MG |
| 54868619400 | Aliskiren-Hydrochlorothiazide | Aliskiren-Hydrochlorothiazide Tab 300-25 MG |
| 54868610300 | Aliskiren-Hydrochlorothiazide | Aliskiren-Hydrochlorothiazide Tab 300-12.5 MG |
| 00078052115 | Aliskiren-Hydrochlorothiazide | Aliskiren-Hydrochlorothiazide Tab 150-12.5 MG |
| 00078052415 | Aliskiren-Hydrochlorothiazide | Aliskiren-Hydrochlorothiazide Tab 300-25 MG |
| 00078057415 | Aliskiren-Valsartan | Aliskiren-Valsartan Tab 300-320 MG |
| 00078057215 | Aliskiren-Valsartan | Aliskiren-Valsartan Tab 150-160 MG |
| 55048002030 | Amlodipine Besylate-Benazepril HCl | Amlodipine Besylate-Benazepril HCl Cap 5-10 MG |
| 55048002130 | Amlodipine Besylate-Benazepril HCl | Amlodipine Besylate-Benazepril HCl Cap 10-20 MG |
| 55048002230 | Amlodipine Besylate-Benazepril HCl | Amlodipine Besylate-Benazepril HCl Cap 5-20 MG |
| 42254007230 | Amlodipine Besylate-Benazepril HCl | Amlodipine Besylate-Benazepril HCl Cap 5-20 MG |
| 42254007330 | Amlodipine Besylate-Benazepril HCl | Amlodipine Besylate-Benazepril HCl Cap 5-40 MG |
| 42254007430 | Amlodipine Besylate-Benazepril HCl | Amlodipine Besylate-Benazepril HCl Cap 10-40 MG |
| 00378689501 | Amlodipine Besylate-Benazepril HCl | Amlodipine Besylate-Benazepril HCl Cap 2.5-10 MG |
| 00378689601 | Amlodipine Besylate-Benazepril HCl | Amlodipine Besylate-Benazepril HCl Cap 5-10 MG |
| 00378689605 | Amlodipine Besylate-Benazepril HCl | Amlodipine Besylate-Benazepril HCl Cap 5-10 MG |
| 00378689701 | Amlodipine Besylate-Benazepril HCl | Amlodipine Besylate-Benazepril HCl Cap 5-20 MG |
| 00378689705 | Amlodipine Besylate-Benazepril HCl | Amlodipine Besylate-Benazepril HCl Cap 5-20 MG |
| 00378689801 | Amlodipine Besylate-Benazepril HCl | Amlodipine Besylate-Benazepril HCl Cap 10-20 MG |
| 00378689805 | Amlodipine Besylate-Benazepril HCl | Amlodipine Besylate-Benazepril HCl Cap 10-20 MG |
| 00378689901 | Amlodipine Besylate-Benazepril HCl | Amlodipine Besylate-Benazepril HCl Cap 5-40 MG |
| 00378690001 | Amlodipine Besylate-Benazepril HCl | Amlodipine Besylate-Benazepril HCl Cap 10-40 MG |
| 55048012330 | Amlodipine Besylate-Benazepril HCl | Amlodipine Besylate-Benazepril HCl Cap 5-20 MG |
| 55048012430 | Amlodipine Besylate-Benazepril HCl | Amlodipine Besylate-Benazepril HCl Cap 10-20 MG |
| 55048012530 | Amlodipine Besylate-Benazepril HCl | Amlodipine Besylate-Benazepril HCl Cap 10-40 MG |
| 54868629700 | Amlodipine Besylate-Benazepril HCl | Amlodipine Besylate-Benazepril HCl Cap 10-40 MG |
| 68258606003 | Amlodipine Besylate-Benazepril HCl | Amlodipine Besylate-Benazepril HCl Cap 5-40 MG |
| 21695086690 | Amlodipine Besylate-Benazepril HCl | Amlodipine Besylate-Benazepril HCl Cap 5-10 MG |
| 00781227701 | Amlodipine Besylate-Benazepril HCl | Amlodipine Besylate-Benazepril HCl Cap 5-40 MG |
| 00781227901 | Amlodipine Besylate-Benazepril HCl | Amlodipine Besylate-Benazepril HCl Cap 10-40 MG |
| 49884092901 | Amlodipine Besylate-Benazepril HCl | Amlodipine Besylate-Benazepril HCl Cap 2.5-10 MG |
| 49884093001 | Amlodipine Besylate-Benazepril HCl | Amlodipine Besylate-Benazepril HCl Cap 5-10 MG |
| 49884093101 | Amlodipine Besylate-Benazepril HCl | Amlodipine Besylate-Benazepril HCl Cap 5-20 MG |
| 49884093201 | Amlodipine Besylate-Benazepril HCl | Amlodipine Besylate-Benazepril HCl Cap 10-20 MG |
| 49884095201 | Amlodipine Besylate-Benazepril HCl | Amlodipine Besylate-Benazepril HCl Cap 5-40 MG |
| 49884095301 | Amlodipine Besylate-Benazepril HCl | Amlodipine Besylate-Benazepril HCl Cap 10-40 MG |
| 54868579300 | Amlodipine Besylate-Benazepril HCl | Amlodipine Besylate-Benazepril HCl Cap 2.5-10 MG |
| 54868579301 | Amlodipine Besylate-Benazepril HCl | Amlodipine Besylate-Benazepril HCl Cap 2.5-10 MG |
| 21695091630 | Amlodipine Besylate-Benazepril HCl | Amlodipine Besylate-Benazepril HCl Cap 10-20 MG |
| 21695091690 | Amlodipine Besylate-Benazepril HCl | Amlodipine Besylate-Benazepril HCl Cap 10-20 MG |
| 00093737210 | Amlodipine Besylate-Benazepril HCl | Amlodipine Besylate-Benazepril HCl Cap 5-20 MG |
| 49884092905 | Amlodipine Besylate-Benazepril HCl | Amlodipine Besylate-Benazepril HCl Cap 2.5-10 MG |
| 49884093005 | Amlodipine Besylate-Benazepril HCl | Amlodipine Besylate-Benazepril HCl Cap 5-10 MG |
| 49884093105 | Amlodipine Besylate-Benazepril HCl | Amlodipine Besylate-Benazepril HCl Cap 5-20 MG |
| 49884093205 | Amlodipine Besylate-Benazepril HCl | Amlodipine Besylate-Benazepril HCl Cap 10-20 MG |
| 49884095205 | Amlodipine Besylate-Benazepril HCl | Amlodipine Besylate-Benazepril HCl Cap 5-40 MG |
| 49884095305 | Amlodipine Besylate-Benazepril HCl | Amlodipine Besylate-Benazepril HCl Cap 10-40 MG |
| 23490706001 | Amlodipine Besylate-Benazepril HCl | Amlodipine Besylate-Benazepril HCl Cap 5-10 MG |
| 23490706201 | Amlodipine Besylate-Benazepril HCl | Amlodipine Besylate-Benazepril HCl Cap 10-20 MG |
| 00093767001 | Amlodipine Besylate-Benazepril HCl | Amlodipine Besylate-Benazepril HCl Cap 5-40 MG |
| 00093767101 | Amlodipine Besylate-Benazepril HCl | Amlodipine Besylate-Benazepril HCl Cap 10-40 MG |
| 68180075901 | Amlodipine Besylate-Benazepril HCl | Amlodipine Besylate-Benazepril HCl Cap 5-40 MG |
| 68180076001 | Amlodipine Besylate-Benazepril HCl | Amlodipine Besylate-Benazepril HCl Cap 10-40 MG |
| 68258602503 | Amlodipine Besylate-Benazepril HCl | Amlodipine Besylate-Benazepril HCl Cap 10-20 MG |
| 68258602509 | Amlodipine Besylate-Benazepril HCl | Amlodipine Besylate-Benazepril HCl Cap 10-20 MG |
| 55111058601 | Amlodipine Besylate-Benazepril HCl | Amlodipine Besylate-Benazepril HCl Cap 10-40 MG |
| 55111058701 | Amlodipine Besylate-Benazepril HCl | Amlodipine Besylate-Benazepril HCl Cap 5-40 MG |
| 51138041830 | Amlodipine Besylate-Benazepril HCl | Amlodipine Besylate-Benazepril HCl Cap 2.5-10 MG |
| 51138041930 | Amlodipine Besylate-Benazepril HCl | Amlodipine Besylate-Benazepril HCl Cap 5-10 MG |
| 51138042030 | Amlodipine Besylate-Benazepril HCl | Amlodipine Besylate-Benazepril HCl Cap 5-20 MG |
| 51138042230 | Amlodipine Besylate-Benazepril HCl | Amlodipine Besylate-Benazepril HCl Cap 10-20 MG |
| 51138043230 | Amlodipine Besylate-Benazepril HCl | Amlodipine Besylate-Benazepril HCl Cap 2.5-10 MG |
| 51138043330 | Amlodipine Besylate-Benazepril HCl | Amlodipine Besylate-Benazepril HCl Cap 5-10 MG |
| 51138043430 | Amlodipine Besylate-Benazepril HCl | Amlodipine Besylate-Benazepril HCl Cap 5-20 MG |
| 51138043530 | Amlodipine Besylate-Benazepril HCl | Amlodipine Besylate-Benazepril HCl Cap 10-20 MG |
| 00591376101 | Amlodipine Besylate-Benazepril HCl | Amlodipine Besylate-Benazepril HCl Cap 5-40 MG |
| 00591376201 | Amlodipine Besylate-Benazepril HCl | Amlodipine Besylate-Benazepril HCl Cap 10-40 MG |
| 51138053110 | Amlodipine Besylate-Benazepril HCl | Amlodipine Besylate-Benazepril HCl Cap 2.5-10 MG |
| 51138053130 | Amlodipine Besylate-Benazepril HCl | Amlodipine Besylate-Benazepril HCl Cap 2.5-10 MG |
| 51138053210 | Amlodipine Besylate-Benazepril HCl | Amlodipine Besylate-Benazepril HCl Cap 5-10 MG |
| 51138053230 | Amlodipine Besylate-Benazepril HCl | Amlodipine Besylate-Benazepril HCl Cap 5-10 MG |
| 51138053310 | Amlodipine Besylate-Benazepril HCl | Amlodipine Besylate-Benazepril HCl Cap 5-20 MG |
| 51138053330 | Amlodipine Besylate-Benazepril HCl | Amlodipine Besylate-Benazepril HCl Cap 5-20 MG |
| 51138053410 | Amlodipine Besylate-Benazepril HCl | Amlodipine Besylate-Benazepril HCl Cap 5-40 MG |
| 51138053430 | Amlodipine Besylate-Benazepril HCl | Amlodipine Besylate-Benazepril HCl Cap 5-40 MG |
| 51138053510 | Amlodipine Besylate-Benazepril HCl | Amlodipine Besylate-Benazepril HCl Cap 10-20 MG |
| 51138053530 | Amlodipine Besylate-Benazepril HCl | Amlodipine Besylate-Benazepril HCl Cap 10-20 MG |
| 51138053610 | Amlodipine Besylate-Benazepril HCl | Amlodipine Besylate-Benazepril HCl Cap 10-40 MG |
| 51138053630 | Amlodipine Besylate-Benazepril HCl | Amlodipine Besylate-Benazepril HCl Cap 10-40 MG |
| 54868578204 | Amlodipine Besylate-Benazepril HCl | Amlodipine Besylate-Benazepril HCl Cap 5-20 MG |
| 68180075501 | Amlodipine Besylate-Benazepril HCl | Amlodipine Besylate-Benazepril HCl Cap 2.5-10 MG |
| 68180075601 | Amlodipine Besylate-Benazepril HCl | Amlodipine Besylate-Benazepril HCl Cap 5-10 MG |
| 68180075701 | Amlodipine Besylate-Benazepril HCl | Amlodipine Besylate-Benazepril HCl Cap 5-20 MG |
| 68180075801 | Amlodipine Besylate-Benazepril HCl | Amlodipine Besylate-Benazepril HCl Cap 10-20 MG |
| 68180075602 | Amlodipine Besylate-Benazepril HCl | Amlodipine Besylate-Benazepril HCl Cap 5-10 MG |
| 68180075702 | Amlodipine Besylate-Benazepril HCl | Amlodipine Besylate-Benazepril HCl Cap 5-20 MG |
| 68180075802 | Amlodipine Besylate-Benazepril HCl | Amlodipine Besylate-Benazepril HCl Cap 10-20 MG |
| 43063023230 | Amlodipine Besylate-Benazepril HCl | Amlodipine Besylate-Benazepril HCl Cap 10-20 MG |
| 43063023260 | Amlodipine Besylate-Benazepril HCl | Amlodipine Besylate-Benazepril HCl Cap 10-20 MG |
| 43063023360 | Amlodipine Besylate-Benazepril HCl | Amlodipine Besylate-Benazepril HCl Cap 5-10 MG |
| 43063023460 | Amlodipine Besylate-Benazepril HCl | Amlodipine Besylate-Benazepril HCl Cap 5-20 MG |
| 55111033801 | Amlodipine Besylate-Benazepril HCl | Amlodipine Besylate-Benazepril HCl Cap 2.5-10 MG |
| 55111033901 | Amlodipine Besylate-Benazepril HCl | Amlodipine Besylate-Benazepril HCl Cap 5-10 MG |
| 55111034001 | Amlodipine Besylate-Benazepril HCl | Amlodipine Besylate-Benazepril HCl Cap 5-20 MG |
| 55111034101 | Amlodipine Besylate-Benazepril HCl | Amlodipine Besylate-Benazepril HCl Cap 10-20 MG |
| 00781227164 | Amlodipine Besylate-Benazepril HCl | Amlodipine Besylate-Benazepril HCl Cap 2.5-10 MG |
| 00781227264 | Amlodipine Besylate-Benazepril HCl | Amlodipine Besylate-Benazepril HCl Cap 5-10 MG |
| 00781227364 | Amlodipine Besylate-Benazepril HCl | Amlodipine Besylate-Benazepril HCl Cap 5-20 MG |
| 00781227464 | Amlodipine Besylate-Benazepril HCl | Amlodipine Besylate-Benazepril HCl Cap 10-20 MG |
| 55111033905 | Amlodipine Besylate-Benazepril HCl | Amlodipine Besylate-Benazepril HCl Cap 5-10 MG |
| 55111034005 | Amlodipine Besylate-Benazepril HCl | Amlodipine Besylate-Benazepril HCl Cap 5-20 MG |
| 55111034105 | Amlodipine Besylate-Benazepril HCl | Amlodipine Besylate-Benazepril HCl Cap 10-20 MG |
| 63629145401 | Amlodipine Besylate-Benazepril HCl | Amlodipine Besylate-Benazepril HCl Cap 5-20 MG |
| 00093737110 | Amlodipine Besylate-Benazepril HCl | Amlodipine Besylate-Benazepril HCl Cap 5-10 MG |
| 00093737310 | Amlodipine Besylate-Benazepril HCl | Amlodipine Besylate-Benazepril HCl Cap 10-20 MG |
| 68084034911 | Amlodipine Besylate-Benazepril HCl | Amlodipine Besylate-Benazepril HCl Cap 5-10 MG |
| 68084034921 | Amlodipine Besylate-Benazepril HCl | Amlodipine Besylate-Benazepril HCl Cap 5-10 MG |
| 68084035001 | Amlodipine Besylate-Benazepril HCl | Amlodipine Besylate-Benazepril HCl Cap 5-20 MG |
| 68084035011 | Amlodipine Besylate-Benazepril HCl | Amlodipine Besylate-Benazepril HCl Cap 5-20 MG |
| 68084035111 | Amlodipine Besylate-Benazepril HCl | Amlodipine Besylate-Benazepril HCl Cap 10-20 MG |
| 68084035121 | Amlodipine Besylate-Benazepril HCl | Amlodipine Besylate-Benazepril HCl Cap 10-20 MG |
| 00591375701 | Amlodipine Besylate-Benazepril HCl | Amlodipine Besylate-Benazepril HCl Cap 2.5-10 MG |
| 00591375801 | Amlodipine Besylate-Benazepril HCl | Amlodipine Besylate-Benazepril HCl Cap 5-10 MG |
| 00591375805 | Amlodipine Besylate-Benazepril HCl | Amlodipine Besylate-Benazepril HCl Cap 5-10 MG |
| 00591375901 | Amlodipine Besylate-Benazepril HCl | Amlodipine Besylate-Benazepril HCl Cap 5-20 MG |
| 00591375905 | Amlodipine Besylate-Benazepril HCl | Amlodipine Besylate-Benazepril HCl Cap 5-20 MG |
| 00591376001 | Amlodipine Besylate-Benazepril HCl | Amlodipine Besylate-Benazepril HCl Cap 10-20 MG |
| 00591376005 | Amlodipine Besylate-Benazepril HCl | Amlodipine Besylate-Benazepril HCl Cap 10-20 MG |
| 47463002030 | Amlodipine Besylate-Benazepril HCl | Amlodipine Besylate-Benazepril HCl Cap 5-10 MG |
| 47463002130 | Amlodipine Besylate-Benazepril HCl | Amlodipine Besylate-Benazepril HCl Cap 10-20 MG |
| 47463002230 | Amlodipine Besylate-Benazepril HCl | Amlodipine Besylate-Benazepril HCl Cap 5-20 MG |
| 33358022200 | Amlodipine Besylate-Benazepril HCl | Amlodipine Besylate-Benazepril HCl Cap 5-20 MG |
| 54868578102 | Amlodipine Besylate-Benazepril HCl | Amlodipine Besylate-Benazepril HCl Cap 10-20 MG |
| 54868579201 | Amlodipine Besylate-Benazepril HCl | Amlodipine Besylate-Benazepril HCl Cap 5-10 MG |
| 54868579202 | Amlodipine Besylate-Benazepril HCl | Amlodipine Besylate-Benazepril HCl Cap 5-10 MG |
| 54868578203 | Amlodipine Besylate-Benazepril HCl | Amlodipine Besylate-Benazepril HCl Cap 5-20 MG |
| 00440714030 | Amlodipine Besylate-Benazepril HCl | Amlodipine Besylate-Benazepril HCl Cap 2.5-10 MG |
| 00440714090 | Amlodipine Besylate-Benazepril HCl | Amlodipine Besylate-Benazepril HCl Cap 2.5-10 MG |
| 00440714130 | Amlodipine Besylate-Benazepril HCl | Amlodipine Besylate-Benazepril HCl Cap 5-10 MG |
| 00440714190 | Amlodipine Besylate-Benazepril HCl | Amlodipine Besylate-Benazepril HCl Cap 5-10 MG |
| 00440714230 | Amlodipine Besylate-Benazepril HCl | Amlodipine Besylate-Benazepril HCl Cap 5-20 MG |
| 00440714290 | Amlodipine Besylate-Benazepril HCl | Amlodipine Besylate-Benazepril HCl Cap 5-20 MG |
| 00440714330 | Amlodipine Besylate-Benazepril HCl | Amlodipine Besylate-Benazepril HCl Cap 10-20 MG |
| 00440714390 | Amlodipine Besylate-Benazepril HCl | Amlodipine Besylate-Benazepril HCl Cap 10-20 MG |
| 54868578103 | Amlodipine Besylate-Benazepril HCl | Amlodipine Besylate-Benazepril HCl Cap 10-20 MG |
| 54868569003 | Amlodipine Besylate-Benazepril HCl | Amlodipine Besylate-Benazepril HCl Cap 10-40 MG |
| 00781227210 | Amlodipine Besylate-Benazepril HCl | Amlodipine Besylate-Benazepril HCl Cap 5-10 MG |
| 00781227310 | Amlodipine Besylate-Benazepril HCl | Amlodipine Besylate-Benazepril HCl Cap 5-20 MG |
| 00781227410 | Amlodipine Besylate-Benazepril HCl | Amlodipine Besylate-Benazepril HCl Cap 10-20 MG |
| 43063017114 | Amlodipine Besylate-Benazepril HCl | Amlodipine Besylate-Benazepril HCl Cap 2.5-10 MG |
| 68115065000 | Amlodipine Besylate-Benazepril HCl | Amlodipine Besylate-Benazepril HCl Cap 10-20 MG |
| 68115067300 | Amlodipine Besylate-Benazepril HCl | Amlodipine Besylate-Benazepril HCl Cap 5-20 MG |
| 68115077800 | Amlodipine Besylate-Benazepril HCl | Amlodipine Besylate-Benazepril HCl Cap 5-10 MG |
| 00093737001 | Amlodipine Besylate-Benazepril HCl | Amlodipine Besylate-Benazepril HCl Cap 2.5-10 MG |
| 00093737101 | Amlodipine Besylate-Benazepril HCl | Amlodipine Besylate-Benazepril HCl Cap 5-10 MG |
| 00093737201 | Amlodipine Besylate-Benazepril HCl | Amlodipine Besylate-Benazepril HCl Cap 5-20 MG |
| 00093737301 | Amlodipine Besylate-Benazepril HCl | Amlodipine Besylate-Benazepril HCl Cap 10-20 MG |
| 00078037905 | Amlodipine Besylate-Benazepril HCl | Amlodipine Besylate-Benazepril HCl Cap 10-40 MG |
| 00078038405 | Amlodipine Besylate-Benazepril HCl | Amlodipine Besylate-Benazepril HCl Cap 5-40 MG |
| 00781227101 | Amlodipine Besylate-Benazepril HCl | Amlodipine Besylate-Benazepril HCl Cap 2.5-10 MG |
| 00781227201 | Amlodipine Besylate-Benazepril HCl | Amlodipine Besylate-Benazepril HCl Cap 5-10 MG |
| 00781227301 | Amlodipine Besylate-Benazepril HCl | Amlodipine Besylate-Benazepril HCl Cap 5-20 MG |
| 00781227401 | Amlodipine Besylate-Benazepril HCl | Amlodipine Besylate-Benazepril HCl Cap 10-20 MG |
| 49999094630 | Amlodipine Besylate-Benazepril HCl | Amlodipine Besylate-Benazepril HCl Cap 10-20 MG |
| 49999094730 | Amlodipine Besylate-Benazepril HCl | Amlodipine Besylate-Benazepril HCl Cap 5-10 MG |
| 49999094830 | Amlodipine Besylate-Benazepril HCl | Amlodipine Besylate-Benazepril HCl Cap 5-20 MG |
| 54569587800 | Amlodipine Besylate-Benazepril HCl | Amlodipine Besylate-Benazepril HCl Cap 10-20 MG |
| 54868487002 | Amlodipine Besylate-Benazepril HCl | Amlodipine Besylate-Benazepril HCl Cap 10-20 MG |
| 54868578100 | Amlodipine Besylate-Benazepril HCl | Amlodipine Besylate-Benazepril HCl Cap 10-20 MG |
| 54868578200 | Amlodipine Besylate-Benazepril HCl | Amlodipine Besylate-Benazepril HCl Cap 5-20 MG |
| 54868578201 | Amlodipine Besylate-Benazepril HCl | Amlodipine Besylate-Benazepril HCl Cap 5-20 MG |
| 54868578300 | Amlodipine Besylate-Benazepril HCl | Amlodipine Besylate-Benazepril HCl Cap 5-40 MG |
| 54868578301 | Amlodipine Besylate-Benazepril HCl | Amlodipine Besylate-Benazepril HCl Cap 5-40 MG |
| 55289098130 | Amlodipine Besylate-Benazepril HCl | Amlodipine Besylate-Benazepril HCl Cap 10-20 MG |
| 55289003930 | Amlodipine Besylate-Benazepril HCl | Amlodipine Besylate-Benazepril HCl Cap 5-20 MG |
| 55289009630 | Amlodipine Besylate-Benazepril HCl | Amlodipine Besylate-Benazepril HCl Cap 5-10 MG |
| 54868569000 | Amlodipine Besylate-Benazepril HCl | Amlodipine Besylate-Benazepril HCl Cap 10-40 MG |
| 54868569001 | Amlodipine Besylate-Benazepril HCl | Amlodipine Besylate-Benazepril HCl Cap 10-40 MG |
| 54868569002 | Amlodipine Besylate-Benazepril HCl | Amlodipine Besylate-Benazepril HCl Cap 10-40 MG |
| 68115067330 | Amlodipine Besylate-Benazepril HCl | Amlodipine Besylate-Benazepril HCl Cap 5-20 MG |
| 54868578101 | Amlodipine Besylate-Benazepril HCl | Amlodipine Besylate-Benazepril HCl Cap 10-20 MG |
| 54868578202 | Amlodipine Besylate-Benazepril HCl | Amlodipine Besylate-Benazepril HCl Cap 5-20 MG |
| 54868579200 | Amlodipine Besylate-Benazepril HCl | Amlodipine Besylate-Benazepril HCl Cap 5-10 MG |
| 54569593700 | Amlodipine Besylate-Benazepril HCl | Amlodipine Besylate-Benazepril HCl Cap 5-10 MG |
| 54569593800 | Amlodipine Besylate-Benazepril HCl | Amlodipine Besylate-Benazepril HCl Cap 5-20 MG |
| 00083225530 | Amlodipine Besylate-Benazepril HCl | Amlodipine Besylate-Benazepril HCl Cap 2.5-10 MG |
| 00083226030 | Amlodipine Besylate-Benazepril HCl | Amlodipine Besylate-Benazepril HCl Cap 5-10 MG |
| 00083226530 | Amlodipine Besylate-Benazepril HCl | Amlodipine Besylate-Benazepril HCl Cap 5-20 MG |
| 00078036405 | Amlodipine Besylate-Benazepril HCl | Amlodipine Besylate-Benazepril HCl Cap 10-20 MG |
| 00078040405 | Amlodipine Besylate-Benazepril HCl | Amlodipine Besylate-Benazepril HCl Cap 2.5-10 MG |
| 00078040505 | Amlodipine Besylate-Benazepril HCl | Amlodipine Besylate-Benazepril HCl Cap 5-10 MG |
| 00078040605 | Amlodipine Besylate-Benazepril HCl | Amlodipine Besylate-Benazepril HCl Cap 5-20 MG |
| 54569469600 | Amlodipine Besylate-Benazepril HCl | Amlodipine Besylate-Benazepril HCl Cap 5-10 MG |
| 54569469601 | Amlodipine Besylate-Benazepril HCl | Amlodipine Besylate-Benazepril HCl Cap 5-10 MG |
| 54569523200 | Amlodipine Besylate-Benazepril HCl | Amlodipine Besylate-Benazepril HCl Cap 5-20 MG |
| 54569523201 | Amlodipine Besylate-Benazepril HCl | Amlodipine Besylate-Benazepril HCl Cap 5-20 MG |
| 54569528200 | Amlodipine Besylate-Benazepril HCl | Amlodipine Besylate-Benazepril HCl Cap 2.5-10 MG |
| 54868487000 | Amlodipine Besylate-Benazepril HCl | Amlodipine Besylate-Benazepril HCl Cap 10-20 MG |
| 54868487001 | Amlodipine Besylate-Benazepril HCl | Amlodipine Besylate-Benazepril HCl Cap 10-20 MG |
| 54868407300 | Amlodipine Besylate-Benazepril HCl | Amlodipine Besylate-Benazepril HCl Cap 5-10 MG |
| 54868407301 | Amlodipine Besylate-Benazepril HCl | Amlodipine Besylate-Benazepril HCl Cap 5-10 MG |
| 54868407302 | Amlodipine Besylate-Benazepril HCl | Amlodipine Besylate-Benazepril HCl Cap 5-10 MG |
| 54868407303 | Amlodipine Besylate-Benazepril HCl | Amlodipine Besylate-Benazepril HCl Cap 5-10 MG |
| 54868407400 | Amlodipine Besylate-Benazepril HCl | Amlodipine Besylate-Benazepril HCl Cap 5-20 MG |
| 54868407401 | Amlodipine Besylate-Benazepril HCl | Amlodipine Besylate-Benazepril HCl Cap 5-20 MG |
| 54868407402 | Amlodipine Besylate-Benazepril HCl | Amlodipine Besylate-Benazepril HCl Cap 5-20 MG |
| 54868407403 | Amlodipine Besylate-Benazepril HCl | Amlodipine Besylate-Benazepril HCl Cap 5-20 MG |
| 54868407404 | Amlodipine Besylate-Benazepril HCl | Amlodipine Besylate-Benazepril HCl Cap 5-20 MG |
| 54868406600 | Amlodipine Besylate-Benazepril HCl | Amlodipine Besylate-Benazepril HCl Cap 2.5-10 MG |
| 54868406601 | Amlodipine Besylate-Benazepril HCl | Amlodipine Besylate-Benazepril HCl Cap 2.5-10 MG |
| 65597011330 | Amlodipine Besylate-Olmesartan Medoxomil | Amlodipine Besylate-Olmesartan Medoxomil Tab 10-40 MG |
| 65597011310 | Amlodipine Besylate-Olmesartan Medoxomil | Amlodipine Besylate-Olmesartan Medoxomil Tab 10-40 MG |
| 65597011290 | Amlodipine Besylate-Olmesartan Medoxomil | Amlodipine Besylate-Olmesartan Medoxomil Tab 5-40 MG |
| 65597011230 | Amlodipine Besylate-Olmesartan Medoxomil | Amlodipine Besylate-Olmesartan Medoxomil Tab 5-40 MG |
| 65597011210 | Amlodipine Besylate-Olmesartan Medoxomil | Amlodipine Besylate-Olmesartan Medoxomil Tab 5-40 MG |
| 65597011190 | Amlodipine Besylate-Olmesartan Medoxomil | Amlodipine Besylate-Olmesartan Medoxomil Tab 10-20 MG |
| 65597011130 | Amlodipine Besylate-Olmesartan Medoxomil | Amlodipine Besylate-Olmesartan Medoxomil Tab 10-20 MG |
| 65597011390 | Amlodipine Besylate-Olmesartan Medoxomil | Amlodipine Besylate-Olmesartan Medoxomil Tab 10-40 MG |
| 54868625000 | Amlodipine Besylate-Olmesartan Medoxomil | Amlodipine Besylate-Olmesartan Medoxomil Tab 10-40 MG |
| 65597011010 | Amlodipine Besylate-Olmesartan Medoxomil | Amlodipine Besylate-Olmesartan Medoxomil Tab 5-20 MG |
| 65597011030 | Amlodipine Besylate-Olmesartan Medoxomil | Amlodipine Besylate-Olmesartan Medoxomil Tab 5-20 MG |
| 65597011090 | Amlodipine Besylate-Olmesartan Medoxomil | Amlodipine Besylate-Olmesartan Medoxomil Tab 5-20 MG |
| 65597011110 | Amlodipine Besylate-Olmesartan Medoxomil | Amlodipine Besylate-Olmesartan Medoxomil Tab 10-20 MG |
| 54868603600 | Amlodipine Besylate-Olmesartan Medoxomil | Amlodipine Besylate-Olmesartan Medoxomil Tab 5-20 MG |
| 54868580400 | Amlodipine Besylate-Valsartan | Amlodipine Besylate-Valsartan Tab 10-160 MG |
| 54868599701 | Amlodipine Besylate-Valsartan | Amlodipine Besylate-Valsartan Tab 5-160 MG |
| 47463002430 | Amlodipine Besylate-Valsartan | Amlodipine Besylate-Valsartan Tab 5-160 MG |
| 47463002330 | Amlodipine Besylate-Valsartan | Amlodipine Besylate-Valsartan Tab 10-320 MG |
| 00078049115 | Amlodipine Besylate-Valsartan | Amlodipine Besylate-Valsartan Tab 10-320 MG |
| 55048002330 | Amlodipine Besylate-Valsartan | Amlodipine Besylate-Valsartan Tab 10-320 MG |
| 55048002430 | Amlodipine Besylate-Valsartan | Amlodipine Besylate-Valsartan Tab 5-160 MG |
| 00078049015 | Amlodipine Besylate-Valsartan | Amlodipine Besylate-Valsartan Tab 5-320 MG |
| 00078048915 | Amlodipine Besylate-Valsartan | Amlodipine Besylate-Valsartan Tab 10-160 MG |
| 00078048815 | Amlodipine Besylate-Valsartan | Amlodipine Besylate-Valsartan Tab 5-160 MG |
| 54868599700 | Amlodipine Besylate-Valsartan | Amlodipine Besylate-Valsartan Tab 5-160 MG |
| 54868599601 | Amlodipine Besylate-Valsartan | Amlodipine Besylate-Valsartan Tab 5-320 MG |
| 54868598300 | Amlodipine Besylate-Valsartan | Amlodipine Besylate-Valsartan Tab 10-320 MG |
| 54868598301 | Amlodipine Besylate-Valsartan | Amlodipine Besylate-Valsartan Tab 10-320 MG |
| 54868599600 | Amlodipine Besylate-Valsartan | Amlodipine Besylate-Valsartan Tab 5-320 MG |
| 00078056215 | Amlodipine-Valsartan-Hydrochlorothiazide | Amlodipine-Valsartan-Hydrochlorothiazide Tab 10-160-25 MG |
| 00078056115 | Amlodipine-Valsartan-Hydrochlorothiazide | Amlodipine-Valsartan-Hydrochlorothiazide Tab 10-160-12.5 MG |
| 00078056015 | Amlodipine-Valsartan-Hydrochlorothiazide | Amlodipine-Valsartan-Hydrochlorothiazide Tab 5-160-25 MG |
| 00078055915 | Amlodipine-Valsartan-Hydrochlorothiazide | Amlodipine-Valsartan-Hydrochlorothiazide Tab 5-160-12.5 MG |
| 00078056315 | Amlodipine-Valsartan-Hydrochlorothiazide | Amlodipine-Valsartan-Hydrochlorothiazide Tab 10-320-25 MG |
| 54868612300 | Amlodipine-Valsartan-Hydrochlorothiazide | Amlodipine-Valsartan-Hydrochlorothiazide Tab 10-320-25 MG |
| 54569059600 | Atenolol & Chlorthalidone | Atenolol & Chlorthalidone Tab 50-25 MG |
| 54868032100 | Atenolol & Chlorthalidone | Atenolol & Chlorthalidone Tab 50-25 MG |
| 54868268300 | Atenolol & Chlorthalidone | Atenolol & Chlorthalidone Tab 50-25 MG |
| 54868268301 | Atenolol & Chlorthalidone | Atenolol & Chlorthalidone Tab 50-25 MG |
| 54868268302 | Atenolol & Chlorthalidone | Atenolol & Chlorthalidone Tab 50-25 MG |
| 54868306400 | Atenolol & Chlorthalidone | Atenolol & Chlorthalidone Tab 100-25 MG |
| 55289098830 | Atenolol & Chlorthalidone | Atenolol & Chlorthalidone Tab 100-25 MG |
| 55289099330 | Atenolol & Chlorthalidone | Atenolol & Chlorthalidone Tab 50-25 MG |
| 55887058530 | Atenolol & Chlorthalidone | Atenolol & Chlorthalidone Tab 50-25 MG |
| 55887058560 | Atenolol & Chlorthalidone | Atenolol & Chlorthalidone Tab 50-25 MG |
| 55887058582 | Atenolol & Chlorthalidone | Atenolol & Chlorthalidone Tab 50-25 MG |
| 55887058590 | Atenolol & Chlorthalidone | Atenolol & Chlorthalidone Tab 50-25 MG |
| 55887061330 | Atenolol & Chlorthalidone | Atenolol & Chlorthalidone Tab 100-25 MG |
| 55887061360 | Atenolol & Chlorthalidone | Atenolol & Chlorthalidone Tab 100-25 MG |
| 55887061382 | Atenolol & Chlorthalidone | Atenolol & Chlorthalidone Tab 100-25 MG |
| 55887061390 | Atenolol & Chlorthalidone | Atenolol & Chlorthalidone Tab 100-25 MG |
| 58016033100 | Atenolol & Chlorthalidone | Atenolol & Chlorthalidone Tab 100-25 MG |
| 58016033130 | Atenolol & Chlorthalidone | Atenolol & Chlorthalidone Tab 100-25 MG |
| 58016033160 | Atenolol & Chlorthalidone | Atenolol & Chlorthalidone Tab 100-25 MG |
| 58016033190 | Atenolol & Chlorthalidone | Atenolol & Chlorthalidone Tab 100-25 MG |
| 58016052600 | Atenolol & Chlorthalidone | Atenolol & Chlorthalidone Tab 50-25 MG |
| 58016052602 | Atenolol & Chlorthalidone | Atenolol & Chlorthalidone Tab 50-25 MG |
| 58016052630 | Atenolol & Chlorthalidone | Atenolol & Chlorthalidone Tab 50-25 MG |
| 23490530003 | Atenolol & Chlorthalidone | Atenolol & Chlorthalidone Tab 100-25 MG |
| 23490530009 | Atenolol & Chlorthalidone | Atenolol & Chlorthalidone Tab 100-25 MG |
| 55289099390 | Atenolol & Chlorthalidone | Atenolol & Chlorthalidone Tab 50-25 MG |
| 43683014230 | Atenolol & Chlorthalidone | Atenolol & Chlorthalidone Tab 50-25 MG |
| 43683014330 | Atenolol & Chlorthalidone | Atenolol & Chlorthalidone Tab 100-25 MG |
| 21695074430 | Atenolol & Chlorthalidone | Atenolol & Chlorthalidone Tab 100-25 MG |
| 55045275400 | Atenolol & Chlorthalidone | Atenolol & Chlorthalidone Tab 100-25 MG |
| 55045275500 | Atenolol & Chlorthalidone | Atenolol & Chlorthalidone Tab 50-25 MG |
| 00904620060 | Atenolol & Chlorthalidone | Atenolol & Chlorthalidone Tab 50-25 MG |
| 63629290901 | Atenolol & Chlorthalidone | Atenolol & Chlorthalidone Tab 50-25 MG |
| 63629290902 | Atenolol & Chlorthalidone | Atenolol & Chlorthalidone Tab 50-25 MG |
| 63629290903 | Atenolol & Chlorthalidone | Atenolol & Chlorthalidone Tab 50-25 MG |
| 63629290904 | Atenolol & Chlorthalidone | Atenolol & Chlorthalidone Tab 50-25 MG |
| 63629373801 | Atenolol & Chlorthalidone | Atenolol & Chlorthalidone Tab 100-25 MG |
| 52555054801 | Atenolol & Chlorthalidone | Atenolol & Chlorthalidone Tab 100-25 MG |
| 54569059601 | Atenolol & Chlorthalidone | Atenolol & Chlorthalidone Tab 50-25 MG |
| 54569059800 | Atenolol & Chlorthalidone | Atenolol & Chlorthalidone Tab 100-25 MG |
| 54569059801 | Atenolol & Chlorthalidone | Atenolol & Chlorthalidone Tab 100-25 MG |
| 54921011510 | Atenolol & Chlorthalidone | Atenolol & Chlorthalidone Tab 50-25 MG |
| 54921011710 | Atenolol & Chlorthalidone | Atenolol & Chlorthalidone Tab 100-25 MG |
| 59772259201 | Atenolol & Chlorthalidone | Atenolol & Chlorthalidone Tab 50-25 MG |
| 59772259301 | Atenolol & Chlorthalidone | Atenolol & Chlorthalidone Tab 100-25 MG |
| 21695074330 | Atenolol & Chlorthalidone | Atenolol & Chlorthalidone Tab 50-25 MG |
| 54868306401 | Atenolol & Chlorthalidone | Atenolol & Chlorthalidone Tab 100-25 MG |
| 66336081190 | Atenolol & Chlorthalidone | Atenolol & Chlorthalidone Tab 50-25 MG |
| 00378206493 | Atenolol & Chlorthalidone | Atenolol & Chlorthalidone Tab 100-25 MG |
| 58016052660 | Atenolol & Chlorthalidone | Atenolol & Chlorthalidone Tab 50-25 MG |
| 58016052690 | Atenolol & Chlorthalidone | Atenolol & Chlorthalidone Tab 50-25 MG |
| 68115045830 | Atenolol & Chlorthalidone | Atenolol & Chlorthalidone Tab 50-25 MG |
| 55289099360 | Atenolol & Chlorthalidone | Atenolol & Chlorthalidone Tab 50-25 MG |
| 00182194201 | Atenolol & Chlorthalidone | Atenolol & Chlorthalidone Tab 50-25 MG |
| 00182194301 | Atenolol & Chlorthalidone | Atenolol & Chlorthalidone Tab 100-25 MG |
| 00339583912 | Atenolol & Chlorthalidone | Atenolol & Chlorthalidone Tab 50-25 MG |
| 00339584112 | Atenolol & Chlorthalidone | Atenolol & Chlorthalidone Tab 100-25 MG |
| 00364252701 | Atenolol & Chlorthalidone | Atenolol & Chlorthalidone Tab 50-25 MG |
| 00364252801 | Atenolol & Chlorthalidone | Atenolol & Chlorthalidone Tab 100-25 MG |
| 00405410301 | Atenolol & Chlorthalidone | Atenolol & Chlorthalidone Tab 50-25 MG |
| 00405410401 | Atenolol & Chlorthalidone | Atenolol & Chlorthalidone Tab 100-25 MG |
| 00536333201 | Atenolol & Chlorthalidone | Atenolol & Chlorthalidone Tab 50-25 MG |
| 00536333301 | Atenolol & Chlorthalidone | Atenolol & Chlorthalidone Tab 100-25 MG |
| 00781131501 | Atenolol & Chlorthalidone | Atenolol & Chlorthalidone Tab 50-25 MG |
| 00781131601 | Atenolol & Chlorthalidone | Atenolol & Chlorthalidone Tab 100-25 MG |
| 00839780706 | Atenolol & Chlorthalidone | Atenolol & Chlorthalidone Tab 50-25 MG |
| 00839780806 | Atenolol & Chlorthalidone | Atenolol & Chlorthalidone Tab 100-25 MG |
| 52555045001 | Atenolol & Chlorthalidone | Atenolol & Chlorthalidone Tab 50-25 MG |
| 52555045010 | Atenolol & Chlorthalidone | Atenolol & Chlorthalidone Tab 50-25 MG |
| 52555045110 | Atenolol & Chlorthalidone | Atenolol & Chlorthalidone Tab 100-25 MG |
| 52555054701 | Atenolol & Chlorthalidone | Atenolol & Chlorthalidone Tab 50-25 MG |
| 00310011510 | Atenolol & Chlorthalidone | Atenolol & Chlorthalidone Tab 50-25 MG |
| 00310011710 | Atenolol & Chlorthalidone | Atenolol & Chlorthalidone Tab 100-25 MG |
| 00378206301 | Atenolol & Chlorthalidone | Atenolol & Chlorthalidone Tab 50-25 MG |
| 00378206401 | Atenolol & Chlorthalidone | Atenolol & Chlorthalidone Tab 100-25 MG |
| 00591578201 | Atenolol & Chlorthalidone | Atenolol & Chlorthalidone Tab 50-25 MG |
| 00591578301 | Atenolol & Chlorthalidone | Atenolol & Chlorthalidone Tab 100-25 MG |
| 00603237421 | Atenolol & Chlorthalidone | Atenolol & Chlorthalidone Tab 50-25 MG |
| 00603237521 | Atenolol & Chlorthalidone | Atenolol & Chlorthalidone Tab 100-25 MG |
| 00677148001 | Atenolol & Chlorthalidone | Atenolol & Chlorthalidone Tab 50-25 MG |
| 00677148101 | Atenolol & Chlorthalidone | Atenolol & Chlorthalidone Tab 100-25 MG |
| 00904788160 | Atenolol & Chlorthalidone | Atenolol & Chlorthalidone Tab 50-25 MG |
| 00904788260 | Atenolol & Chlorthalidone | Atenolol & Chlorthalidone Tab 100-25 MG |
| 49999051230 | Atenolol & Chlorthalidone | Atenolol & Chlorthalidone Tab 50-25 MG |
| 52555045101 | Atenolol & Chlorthalidone | Atenolol & Chlorthalidone Tab 100-25 MG |
| 53489053101 | Atenolol & Chlorthalidone | Atenolol & Chlorthalidone Tab 50-25 MG |
| 53489053201 | Atenolol & Chlorthalidone | Atenolol & Chlorthalidone Tab 100-25 MG |
| 64764084430 | Azilsartan Medoxomil | Azilsartan Medoxomil Tab 40 MG |
| 64764088430 | Azilsartan Medoxomil | Azilsartan Medoxomil Tab 80 MG |
| 64764094430 | Azilsartan Medoxomil-Chlorthalidone | Azilsartan Medoxomil-Chlorthalidone Tab 40-12.5 MG |
| 64764099430 | Azilsartan Medoxomil-Chlorthalidone | Azilsartan Medoxomil-Chlorthalidone Tab 40-25 MG |
| 49884086101 | Benazepril & Hydrochlorothiazide | Benazepril & Hydrochlorothiazide Tab 5-6.25 MG |
| 49884086201 | Benazepril & Hydrochlorothiazide | Benazepril & Hydrochlorothiazide Tab 10-12.5 MG |
| 49884086301 | Benazepril & Hydrochlorothiazide | Benazepril & Hydrochlorothiazide Tab 20-12.5 MG |
| 49884086401 | Benazepril & Hydrochlorothiazide | Benazepril & Hydrochlorothiazide Tab 20-25 MG |
| 00083005730 | Benazepril & Hydrochlorothiazide | Benazepril & Hydrochlorothiazide Tab 5-6.25 MG |
| 00083007230 | Benazepril & Hydrochlorothiazide | Benazepril & Hydrochlorothiazide Tab 10-12.5 MG |
| 00083007430 | Benazepril & Hydrochlorothiazide | Benazepril & Hydrochlorothiazide Tab 20-12.5 MG |
| 00083007530 | Benazepril & Hydrochlorothiazide | Benazepril & Hydrochlorothiazide Tab 20-25 MG |
| 00172536060 | Benazepril & Hydrochlorothiazide | Benazepril & Hydrochlorothiazide Tab 5-6.25 MG |
| 00172536160 | Benazepril & Hydrochlorothiazide | Benazepril & Hydrochlorothiazide Tab 10-12.5 MG |
| 00172536170 | Benazepril & Hydrochlorothiazide | Benazepril & Hydrochlorothiazide Tab 10-12.5 MG |
| 00172536260 | Benazepril & Hydrochlorothiazide | Benazepril & Hydrochlorothiazide Tab 20-12.5 MG |
| 00172536270 | Benazepril & Hydrochlorothiazide | Benazepril & Hydrochlorothiazide Tab 20-12.5 MG |
| 00172536360 | Benazepril & Hydrochlorothiazide | Benazepril & Hydrochlorothiazide Tab 20-25 MG |
| 00172536370 | Benazepril & Hydrochlorothiazide | Benazepril & Hydrochlorothiazide Tab 20-25 MG |
| 00185020401 | Benazepril & Hydrochlorothiazide | Benazepril & Hydrochlorothiazide Tab 10-12.5 MG |
| 00185021101 | Benazepril & Hydrochlorothiazide | Benazepril & Hydrochlorothiazide Tab 20-12.5 MG |
| 00185027701 | Benazepril & Hydrochlorothiazide | Benazepril & Hydrochlorothiazide Tab 20-25 MG |
| 54868390602 | Benazepril & Hydrochlorothiazide | Benazepril & Hydrochlorothiazide Tab 20-12.5 MG |
| 68258605903 | Benazepril & Hydrochlorothiazide | Benazepril & Hydrochlorothiazide Tab 20-25 MG |
| 63629180902 | Benazepril & Hydrochlorothiazide | Benazepril & Hydrochlorothiazide Tab 10-12.5 MG |
| 63629268003 | Benazepril & Hydrochlorothiazide | Benazepril & Hydrochlorothiazide Tab 20-12.5 MG |
| 33261045330 | Benazepril & Hydrochlorothiazide | Benazepril & Hydrochlorothiazide Tab 10-12.5 MG |
| 33261045360 | Benazepril & Hydrochlorothiazide | Benazepril & Hydrochlorothiazide Tab 10-12.5 MG |
| 33261045390 | Benazepril & Hydrochlorothiazide | Benazepril & Hydrochlorothiazide Tab 10-12.5 MG |
| 54569568501 | Benazepril & Hydrochlorothiazide | Benazepril & Hydrochlorothiazide Tab 20-25 MG |
| 51138039230 | Benazepril & Hydrochlorothiazide | Benazepril & Hydrochlorothiazide Tab 5-6.25 MG |
| 51138039330 | Benazepril & Hydrochlorothiazide | Benazepril & Hydrochlorothiazide Tab 10-12.5 MG |
| 51138039430 | Benazepril & Hydrochlorothiazide | Benazepril & Hydrochlorothiazide Tab 20-12.5 MG |
| 51138039530 | Benazepril & Hydrochlorothiazide | Benazepril & Hydrochlorothiazide Tab 20-25 MG |
| 63629273501 | Benazepril & Hydrochlorothiazide | Benazepril & Hydrochlorothiazide Tab 20-25 MG |
| 63304015501 | Benazepril & Hydrochlorothiazide | Benazepril & Hydrochlorothiazide Tab 10-12.5 MG |
| 63304015601 | Benazepril & Hydrochlorothiazide | Benazepril & Hydrochlorothiazide Tab 20-12.5 MG |
| 63304015701 | Benazepril & Hydrochlorothiazide | Benazepril & Hydrochlorothiazide Tab 20-25 MG |
| 52959090730 | Benazepril & Hydrochlorothiazide | Benazepril & Hydrochlorothiazide Tab 20-25 MG |
| 33358004730 | Benazepril & Hydrochlorothiazide | Benazepril & Hydrochlorothiazide Tab 10-12.5 MG |
| 33358005030 | Benazepril & Hydrochlorothiazide | Benazepril & Hydrochlorothiazide Tab 20-25 MG |
| 63629180901 | Benazepril & Hydrochlorothiazide | Benazepril & Hydrochlorothiazide Tab 10-12.5 MG |
| 63629268001 | Benazepril & Hydrochlorothiazide | Benazepril & Hydrochlorothiazide Tab 20-12.5 MG |
| 63629268002 | Benazepril & Hydrochlorothiazide | Benazepril & Hydrochlorothiazide Tab 20-12.5 MG |
| 00078045105 | Benazepril & Hydrochlorothiazide | Benazepril & Hydrochlorothiazide Tab 5-6.25 MG |
| 00078045205 | Benazepril & Hydrochlorothiazide | Benazepril & Hydrochlorothiazide Tab 10-12.5 MG |
| 00078045305 | Benazepril & Hydrochlorothiazide | Benazepril & Hydrochlorothiazide Tab 20-12.5 MG |
| 00078045405 | Benazepril & Hydrochlorothiazide | Benazepril & Hydrochlorothiazide Tab 20-25 MG |
| 12280022000 | Benazepril & Hydrochlorothiazide | Benazepril & Hydrochlorothiazide Tab 20-25 MG |
| 58016006500 | Benazepril & Hydrochlorothiazide | Benazepril & Hydrochlorothiazide Tab 20-12.5 MG |
| 58016006530 | Benazepril & Hydrochlorothiazide | Benazepril & Hydrochlorothiazide Tab 20-12.5 MG |
| 58016006560 | Benazepril & Hydrochlorothiazide | Benazepril & Hydrochlorothiazide Tab 20-12.5 MG |
| 58016006590 | Benazepril & Hydrochlorothiazide | Benazepril & Hydrochlorothiazide Tab 20-12.5 MG |
| 54868529600 | Benazepril & Hydrochlorothiazide | Benazepril & Hydrochlorothiazide Tab 20-25 MG |
| 54868531300 | Benazepril & Hydrochlorothiazide | Benazepril & Hydrochlorothiazide Tab 20-25 MG |
| 54868531301 | Benazepril & Hydrochlorothiazide | Benazepril & Hydrochlorothiazide Tab 20-25 MG |
| 54868525600 | Benazepril & Hydrochlorothiazide | Benazepril & Hydrochlorothiazide Tab 10-12.5 MG |
| 55887036630 | Benazepril & Hydrochlorothiazide | Benazepril & Hydrochlorothiazide Tab 10-12.5 MG |
| 55887036660 | Benazepril & Hydrochlorothiazide | Benazepril & Hydrochlorothiazide Tab 10-12.5 MG |
| 55887036690 | Benazepril & Hydrochlorothiazide | Benazepril & Hydrochlorothiazide Tab 10-12.5 MG |
| 62037075601 | Benazepril & Hydrochlorothiazide | Benazepril & Hydrochlorothiazide Tab 5-6.25 MG |
| 62037075701 | Benazepril & Hydrochlorothiazide | Benazepril & Hydrochlorothiazide Tab 10-12.5 MG |
| 62037075801 | Benazepril & Hydrochlorothiazide | Benazepril & Hydrochlorothiazide Tab 20-12.5 MG |
| 62037075901 | Benazepril & Hydrochlorothiazide | Benazepril & Hydrochlorothiazide Tab 20-25 MG |
| 54569528300 | Benazepril & Hydrochlorothiazide | Benazepril & Hydrochlorothiazide Tab 5-6.25 MG |
| 54569528400 | Benazepril & Hydrochlorothiazide | Benazepril & Hydrochlorothiazide Tab 10-12.5 MG |
| 54569528401 | Benazepril & Hydrochlorothiazide | Benazepril & Hydrochlorothiazide Tab 10-12.5 MG |
| 54569568500 | Benazepril & Hydrochlorothiazide | Benazepril & Hydrochlorothiazide Tab 20-25 MG |
| 54569528500 | Benazepril & Hydrochlorothiazide | Benazepril & Hydrochlorothiazide Tab 20-12.5 MG |
| 54569528600 | Benazepril & Hydrochlorothiazide | Benazepril & Hydrochlorothiazide Tab 20-25 MG |
| 54868390600 | Benazepril & Hydrochlorothiazide | Benazepril & Hydrochlorothiazide Tab 20-12.5 MG |
| 54868390601 | Benazepril & Hydrochlorothiazide | Benazepril & Hydrochlorothiazide Tab 20-12.5 MG |
| 54868490400 | Benazepril & Hydrochlorothiazide | Benazepril & Hydrochlorothiazide Tab 20-12.5 MG |
| 54868490401 | Benazepril & Hydrochlorothiazide | Benazepril & Hydrochlorothiazide Tab 20-12.5 MG |
| 00185012401 | Benazepril & Hydrochlorothiazide | Benazepril & Hydrochlorothiazide Tab 5-6.25 MG |
| 00378472501 | Benazepril & Hydrochlorothiazide | Benazepril & Hydrochlorothiazide Tab 5-6.25 MG |
| 00378473501 | Benazepril & Hydrochlorothiazide | Benazepril & Hydrochlorothiazide Tab 10-12.5 MG |
| 00378474501 | Benazepril & Hydrochlorothiazide | Benazepril & Hydrochlorothiazide Tab 20-12.5 MG |
| 00378477501 | Benazepril & Hydrochlorothiazide | Benazepril & Hydrochlorothiazide Tab 20-25 MG |
| 00781513101 | Benazepril & Hydrochlorothiazide | Benazepril & Hydrochlorothiazide Tab 5-6.25 MG |
| 00781513201 | Benazepril & Hydrochlorothiazide | Benazepril & Hydrochlorothiazide Tab 10-12.5 MG |
| 00781513301 | Benazepril & Hydrochlorothiazide | Benazepril & Hydrochlorothiazide Tab 20-12.5 MG |
| 00781513401 | Benazepril & Hydrochlorothiazide | Benazepril & Hydrochlorothiazide Tab 20-25 MG |
| 12280012030 | Benazepril & Hydrochlorothiazide | Benazepril & Hydrochlorothiazide Tab 20-25 MG |
| 12280012130 | Benazepril & Hydrochlorothiazide | Benazepril & Hydrochlorothiazide Tab 20-12.5 MG |
| 12280012330 | Benazepril & Hydrochlorothiazide | Benazepril & Hydrochlorothiazide Tab 10-12.5 MG |
| 51138038030 | Benazepril HCl | Benazepril HCl Tab 10 MG |
| 51138038130 | Benazepril HCl | Benazepril HCl Tab 20 MG |
| 51138038230 | Benazepril HCl | Benazepril HCl Tab 40 MG |
| 55048003930 | Benazepril HCl | Benazepril HCl Tab 20 MG |
| 55048004030 | Benazepril HCl | Benazepril HCl Tab 40 MG |
| 55048004330 | Benazepril HCl | Benazepril HCl Tab 10 MG |
| 23490511902 | Benazepril HCl | Benazepril HCl Tab 20 MG |
| 66336077360 | Benazepril HCl | Benazepril HCl Tab 10 MG |
| 42254015690 | Benazepril HCl | Benazepril HCl Tab 20 MG |
| 42254019290 | Benazepril HCl | Benazepril HCl Tab 5 MG |
| 63629292302 | Benazepril HCl | Benazepril HCl Tab 40 MG |
| 63629267203 | Benazepril HCl | Benazepril HCl Tab 10 MG |
| 63629267901 | Benazepril HCl | Benazepril HCl Tab 5 MG |
| 00904619040 | Benazepril HCl | Benazepril HCl Tab 10 MG |
| 00904619140 | Benazepril HCl | Benazepril HCl Tab 20 MG |
| 00904619240 | Benazepril HCl | Benazepril HCl Tab 40 MG |
| 47463003930 | Benazepril HCl | Benazepril HCl Tab 20 MG |
| 47463004030 | Benazepril HCl | Benazepril HCl Tab 40 MG |
| 47463004330 | Benazepril HCl | Benazepril HCl Tab 10 MG |
| 00904618940 | Benazepril HCl | Benazepril HCl Tab 5 MG |
| 43063028830 | Benazepril HCl | Benazepril HCl Tab 10 MG |
| 54868235000 | Benazepril HCl | Benazepril HCl Tab 10 MG |
| 54868235101 | Benazepril HCl | Benazepril HCl Tab 20 MG |
| 54868507902 | Benazepril HCl | Benazepril HCl Tab 20 MG |
| 33261018002 | Benazepril HCl | Benazepril HCl Tab 20 MG |
| 33261018030 | Benazepril HCl | Benazepril HCl Tab 20 MG |
| 33261018060 | Benazepril HCl | Benazepril HCl Tab 20 MG |
| 33261018090 | Benazepril HCl | Benazepril HCl Tab 20 MG |
| 66336069190 | Benazepril HCl | Benazepril HCl Tab 20 MG |
| 54569566802 | Benazepril HCl | Benazepril HCl Tab 10 MG |
| 54569566901 | Benazepril HCl | Benazepril HCl Tab 20 MG |
| 54569567001 | Benazepril HCl | Benazepril HCl Tab 40 MG |
| 42291016090 | Benazepril HCl | Benazepril HCl Tab 5 MG |
| 42291016190 | Benazepril HCl | Benazepril HCl Tab 10 MG |
| 42291016218 | Benazepril HCl | Benazepril HCl Tab 20 MG |
| 42291016290 | Benazepril HCl | Benazepril HCl Tab 20 MG |
| 42291016318 | Benazepril HCl | Benazepril HCl Tab 40 MG |
| 42291016390 | Benazepril HCl | Benazepril HCl Tab 40 MG |
| 66336012490 | Benazepril HCl | Benazepril HCl Tab 40 MG |
| 51138039630 | Benazepril HCl | Benazepril HCl Tab 5 MG |
| 51138039730 | Benazepril HCl | Benazepril HCl Tab 10 MG |
| 51138039830 | Benazepril HCl | Benazepril HCl Tab 20 MG |
| 51138039930 | Benazepril HCl | Benazepril HCl Tab 40 MG |
| 51138037930 | Benazepril HCl | Benazepril HCl Tab 5 MG |
| 63629292301 | Benazepril HCl | Benazepril HCl Tab 40 MG |
| 55289096330 | Benazepril HCl | Benazepril HCl Tab 5 MG |
| 54868520403 | Benazepril HCl | Benazepril HCl Tab 40 MG |
| 21695032660 | Benazepril HCl | Benazepril HCl Tab 10 MG |
| 21695032760 | Benazepril HCl | Benazepril HCl Tab 20 MG |
| 21695032790 | Benazepril HCl | Benazepril HCl Tab 20 MG |
| 21695087730 | Benazepril HCl | Benazepril HCl Tab 40 MG |
| 21695087790 | Benazepril HCl | Benazepril HCl Tab 40 MG |
| 35356043230 | Benazepril HCl | Benazepril HCl Tab 40 MG |
| 54458095610 | Benazepril HCl | Benazepril HCl Tab 40 MG |
| 54458095710 | Benazepril HCl | Benazepril HCl Tab 20 MG |
| 54458095810 | Benazepril HCl | Benazepril HCl Tab 10 MG |
| 54458095910 | Benazepril HCl | Benazepril HCl Tab 5 MG |
| 16590025930 | Benazepril HCl | Benazepril HCl Tab 20 MG |
| 16590028030 | Benazepril HCl | Benazepril HCl Tab 10 MG |
| 43063013130 | Benazepril HCl | Benazepril HCl Tab 20 MG |
| 43063013230 | Benazepril HCl | Benazepril HCl Tab 40 MG |
| 65162075110 | Benazepril HCl | Benazepril HCl Tab 5 MG |
| 65162075150 | Benazepril HCl | Benazepril HCl Tab 5 MG |
| 65162075210 | Benazepril HCl | Benazepril HCl Tab 10 MG |
| 65162075250 | Benazepril HCl | Benazepril HCl Tab 10 MG |
| 65162075310 | Benazepril HCl | Benazepril HCl Tab 20 MG |
| 65162075350 | Benazepril HCl | Benazepril HCl Tab 20 MG |
| 65162075410 | Benazepril HCl | Benazepril HCl Tab 40 MG |
| 65162075450 | Benazepril HCl | Benazepril HCl Tab 40 MG |
| 65862011601 | Benazepril HCl | Benazepril HCl Tab 10 MG |
| 65862011701 | Benazepril HCl | Benazepril HCl Tab 20 MG |
| 65862011801 | Benazepril HCl | Benazepril HCl Tab 40 MG |
| 68071014630 | Benazepril HCl | Benazepril HCl Tab 20 MG |
| 68071002630 | Benazepril HCl | Benazepril HCl Tab 40 MG |
| 68071002660 | Benazepril HCl | Benazepril HCl Tab 40 MG |
| 68071002690 | Benazepril HCl | Benazepril HCl Tab 40 MG |
| 68071014660 | Benazepril HCl | Benazepril HCl Tab 20 MG |
| 68071014690 | Benazepril HCl | Benazepril HCl Tab 20 MG |
| 68071014691 | Benazepril HCl | Benazepril HCl Tab 20 MG |
| 59762314601 | Benazepril HCl | Benazepril HCl Tab 10 MG |
| 59762314602 | Benazepril HCl | Benazepril HCl Tab 10 MG |
| 59762314701 | Benazepril HCl | Benazepril HCl Tab 20 MG |
| 59762314702 | Benazepril HCl | Benazepril HCl Tab 20 MG |
| 59762314801 | Benazepril HCl | Benazepril HCl Tab 40 MG |
| 59762314802 | Benazepril HCl | Benazepril HCl Tab 40 MG |
| 21695032690 | Benazepril HCl | Benazepril HCl Tab 10 MG |
| 21695061290 | Benazepril HCl | Benazepril HCl Tab 5 MG |
| 35356058730 | Benazepril HCl | Benazepril HCl Tab 5 MG |
| 35356058760 | Benazepril HCl | Benazepril HCl Tab 5 MG |
| 35356058790 | Benazepril HCl | Benazepril HCl Tab 5 MG |
| 66336077390 | Benazepril HCl | Benazepril HCl Tab 10 MG |
| 63304033701 | Benazepril HCl | Benazepril HCl Tab 5 MG |
| 63304033801 | Benazepril HCl | Benazepril HCl Tab 10 MG |
| 63304033805 | Benazepril HCl | Benazepril HCl Tab 10 MG |
| 63304033901 | Benazepril HCl | Benazepril HCl Tab 20 MG |
| 63304033905 | Benazepril HCl | Benazepril HCl Tab 20 MG |
| 63304034001 | Benazepril HCl | Benazepril HCl Tab 40 MG |
| 63304034005 | Benazepril HCl | Benazepril HCl Tab 40 MG |
| 63304073801 | Benazepril HCl | Benazepril HCl Tab 20 MG |
| 63304073901 | Benazepril HCl | Benazepril HCl Tab 40 MG |
| 66336023230 | Benazepril HCl | Benazepril HCl Tab 5 MG |
| 66336069130 | Benazepril HCl | Benazepril HCl Tab 20 MG |
| 66336077330 | Benazepril HCl | Benazepril HCl Tab 10 MG |
| 66336012430 | Benazepril HCl | Benazepril HCl Tab 40 MG |
| 68258906201 | Benazepril HCl | Benazepril HCl Tab 5 MG |
| 68115049060 | Benazepril HCl | Benazepril HCl Tab 10 MG |
| 68258105301 | Benazepril HCl | Benazepril HCl Tab 10 MG |
| 68258105401 | Benazepril HCl | Benazepril HCl Tab 20 MG |
| 68258105501 | Benazepril HCl | Benazepril HCl Tab 40 MG |
| 54569373300 | Benazepril HCl | Benazepril HCl Tab 5 MG |
| 54569373301 | Benazepril HCl | Benazepril HCl Tab 5 MG |
| 54569342302 | Benazepril HCl | Benazepril HCl Tab 10 MG |
| 54569342303 | Benazepril HCl | Benazepril HCl Tab 10 MG |
| 55175144201 | Benazepril HCl | Benazepril HCl Tab 10 MG |
| 63874112301 | Benazepril HCl | Benazepril HCl Tab 20 MG |
| 63874112303 | Benazepril HCl | Benazepril HCl Tab 20 MG |
| 33358004830 | Benazepril HCl | Benazepril HCl Tab 10 MG |
| 33358004930 | Benazepril HCl | Benazepril HCl Tab 20 MG |
| 00440719030 | Benazepril HCl | Benazepril HCl Tab 10 MG |
| 00440719230 | Benazepril HCl | Benazepril HCl Tab 20 MG |
| 00440719330 | Benazepril HCl | Benazepril HCl Tab 40 MG |
| 00440719390 | Benazepril HCl | Benazepril HCl Tab 40 MG |
| 54868520402 | Benazepril HCl | Benazepril HCl Tab 40 MG |
| 23629000710 | Benazepril HCl | Benazepril HCl Tab 5 MG |
| 00440719090 | Benazepril HCl | Benazepril HCl Tab 10 MG |
| 00440719290 | Benazepril HCl | Benazepril HCl Tab 20 MG |
| 63629172801 | Benazepril HCl | Benazepril HCl Tab 20 MG |
| 63629172802 | Benazepril HCl | Benazepril HCl Tab 20 MG |
| 63629172803 | Benazepril HCl | Benazepril HCl Tab 20 MG |
| 63629267201 | Benazepril HCl | Benazepril HCl Tab 10 MG |
| 63629267202 | Benazepril HCl | Benazepril HCl Tab 10 MG |
| 63304073805 | Benazepril HCl | Benazepril HCl Tab 20 MG |
| 63304073890 | Benazepril HCl | Benazepril HCl Tab 20 MG |
| 63304073905 | Benazepril HCl | Benazepril HCl Tab 40 MG |
| 63304073990 | Benazepril HCl | Benazepril HCl Tab 40 MG |
| 66116023730 | Benazepril HCl | Benazepril HCl Tab 10 MG |
| 68115021530 | Benazepril HCl | Benazepril HCl Tab 10 MG |
| 68115021630 | Benazepril HCl | Benazepril HCl Tab 20 MG |
| 68115021730 | Benazepril HCl | Benazepril HCl Tab 40 MG |
| 68115059700 | Benazepril HCl | Benazepril HCl Tab 40 MG |
| 68115049030 | Benazepril HCl | Benazepril HCl Tab 10 MG |
| 68115049130 | Benazepril HCl | Benazepril HCl Tab 20 MG |
| 68115061500 | Benazepril HCl | Benazepril HCl Tab 10 MG |
| 68115062100 | Benazepril HCl | Benazepril HCl Tab 5 MG |
| 68115065400 | Benazepril HCl | Benazepril HCl Tab 20 MG |
| 68115082400 | Benazepril HCl | Benazepril HCl Tab 5 MG |
| 21695032630 | Benazepril HCl | Benazepril HCl Tab 10 MG |
| 21695032730 | Benazepril HCl | Benazepril HCl Tab 20 MG |
| 23490511801 | Benazepril HCl | Benazepril HCl Tab 10 MG |
| 23490511901 | Benazepril HCl | Benazepril HCl Tab 20 MG |
| 23490512003 | Benazepril HCl | Benazepril HCl Tab 40 MG |
| 23490512009 | Benazepril HCl | Benazepril HCl Tab 40 MG |
| 23490512101 | Benazepril HCl | Benazepril HCl Tab 5 MG |
| 49999075930 | Benazepril HCl | Benazepril HCl Tab 20 MG |
| 51079014501 | Benazepril HCl | Benazepril HCl Tab 10 MG |
| 51079014601 | Benazepril HCl | Benazepril HCl Tab 20 MG |
| 52959083130 | Benazepril HCl | Benazepril HCl Tab 20 MG |
| 52959083530 | Benazepril HCl | Benazepril HCl Tab 40 MG |
| 52959083560 | Benazepril HCl | Benazepril HCl Tab 40 MG |
| 52959084130 | Benazepril HCl | Benazepril HCl Tab 10 MG |
| 52959084160 | Benazepril HCl | Benazepril HCl Tab 10 MG |
| 54868520401 | Benazepril HCl | Benazepril HCl Tab 40 MG |
| 54868539200 | Benazepril HCl | Benazepril HCl Tab 5 MG |
| 55887022330 | Benazepril HCl | Benazepril HCl Tab 40 MG |
| 55887032230 | Benazepril HCl | Benazepril HCl Tab 20 MG |
| 60505026501 | Benazepril HCl | Benazepril HCl Tab 5 MG |
| 60505026601 | Benazepril HCl | Benazepril HCl Tab 10 MG |
| 60505026605 | Benazepril HCl | Benazepril HCl Tab 10 MG |
| 60505026701 | Benazepril HCl | Benazepril HCl Tab 20 MG |
| 60505026705 | Benazepril HCl | Benazepril HCl Tab 20 MG |
| 60505026801 | Benazepril HCl | Benazepril HCl Tab 40 MG |
| 60505026805 | Benazepril HCl | Benazepril HCl Tab 40 MG |
| 63304073601 | Benazepril HCl | Benazepril HCl Tab 5 MG |
| 63304073701 | Benazepril HCl | Benazepril HCl Tab 10 MG |
| 54868500100 | Benazepril HCl | Benazepril HCl Tab 10 MG |
| 54868500101 | Benazepril HCl | Benazepril HCl Tab 10 MG |
| 54868507900 | Benazepril HCl | Benazepril HCl Tab 20 MG |
| 54868507901 | Benazepril HCl | Benazepril HCl Tab 20 MG |
| 54868520400 | Benazepril HCl | Benazepril HCl Tab 40 MG |
| 55045220101 | Benazepril HCl | Benazepril HCl Tab 40 MG |
| 55289008630 | Benazepril HCl | Benazepril HCl Tab 20 MG |
| 55289010930 | Benazepril HCl | Benazepril HCl Tab 10 MG |
| 55289010997 | Benazepril HCl | Benazepril HCl Tab 10 MG |
| 58016042000 | Benazepril HCl | Benazepril HCl Tab 10 MG |
| 58016042010 | Benazepril HCl | Benazepril HCl Tab 10 MG |
| 58016042030 | Benazepril HCl | Benazepril HCl Tab 10 MG |
| 58016042060 | Benazepril HCl | Benazepril HCl Tab 10 MG |
| 58016042090 | Benazepril HCl | Benazepril HCl Tab 10 MG |
| 58016068500 | Benazepril HCl | Benazepril HCl Tab 20 MG |
| 58016068510 | Benazepril HCl | Benazepril HCl Tab 20 MG |
| 58016068530 | Benazepril HCl | Benazepril HCl Tab 20 MG |
| 58016068560 | Benazepril HCl | Benazepril HCl Tab 20 MG |
| 58016068590 | Benazepril HCl | Benazepril HCl Tab 20 MG |
| 58016068600 | Benazepril HCl | Benazepril HCl Tab 40 MG |
| 58016068610 | Benazepril HCl | Benazepril HCl Tab 40 MG |
| 58016068630 | Benazepril HCl | Benazepril HCl Tab 40 MG |
| 58016068660 | Benazepril HCl | Benazepril HCl Tab 40 MG |
| 58016068690 | Benazepril HCl | Benazepril HCl Tab 40 MG |
| 58177034104 | Benazepril HCl | Benazepril HCl Tab 5 MG |
| 58177034204 | Benazepril HCl | Benazepril HCl Tab 10 MG |
| 58177034208 | Benazepril HCl | Benazepril HCl Tab 10 MG |
| 58177034304 | Benazepril HCl | Benazepril HCl Tab 20 MG |
| 58177034308 | Benazepril HCl | Benazepril HCl Tab 20 MG |
| 58177034404 | Benazepril HCl | Benazepril HCl Tab 40 MG |
| 58177034408 | Benazepril HCl | Benazepril HCl Tab 40 MG |
| 62037051601 | Benazepril HCl | Benazepril HCl Tab 5 MG |
| 62037051701 | Benazepril HCl | Benazepril HCl Tab 10 MG |
| 62037051801 | Benazepril HCl | Benazepril HCl Tab 20 MG |
| 62037051901 | Benazepril HCl | Benazepril HCl Tab 40 MG |
| 62682601406 | Benazepril HCl | Benazepril HCl Tab 20 MG |
| 63304073605 | Benazepril HCl | Benazepril HCl Tab 5 MG |
| 63304073690 | Benazepril HCl | Benazepril HCl Tab 5 MG |
| 63304073705 | Benazepril HCl | Benazepril HCl Tab 10 MG |
| 63304073790 | Benazepril HCl | Benazepril HCl Tab 10 MG |
| 51079014520 | Benazepril HCl | Benazepril HCl Tab 10 MG |
| 51079014620 | Benazepril HCl | Benazepril HCl Tab 20 MG |
| 54348009930 | Benazepril HCl | Benazepril HCl Tab 5 MG |
| 54348010030 | Benazepril HCl | Benazepril HCl Tab 10 MG |
| 54569373302 | Benazepril HCl | Benazepril HCl Tab 5 MG |
| 54569335900 | Benazepril HCl | Benazepril HCl Tab 20 MG |
| 54569335901 | Benazepril HCl | Benazepril HCl Tab 20 MG |
| 54569335902 | Benazepril HCl | Benazepril HCl Tab 20 MG |
| 54569342300 | Benazepril HCl | Benazepril HCl Tab 10 MG |
| 54569342301 | Benazepril HCl | Benazepril HCl Tab 10 MG |
| 54569342304 | Benazepril HCl | Benazepril HCl Tab 10 MG |
| 54569478800 | Benazepril HCl | Benazepril HCl Tab 40 MG |
| 54569478801 | Benazepril HCl | Benazepril HCl Tab 40 MG |
| 54569566800 | Benazepril HCl | Benazepril HCl Tab 10 MG |
| 54569566801 | Benazepril HCl | Benazepril HCl Tab 10 MG |
| 54569566900 | Benazepril HCl | Benazepril HCl Tab 20 MG |
| 54569567000 | Benazepril HCl | Benazepril HCl Tab 40 MG |
| 54868235001 | Benazepril HCl | Benazepril HCl Tab 10 MG |
| 54868235002 | Benazepril HCl | Benazepril HCl Tab 10 MG |
| 54868235003 | Benazepril HCl | Benazepril HCl Tab 10 MG |
| 54868235004 | Benazepril HCl | Benazepril HCl Tab 10 MG |
| 54868235100 | Benazepril HCl | Benazepril HCl Tab 20 MG |
| 54868235102 | Benazepril HCl | Benazepril HCl Tab 20 MG |
| 54868235103 | Benazepril HCl | Benazepril HCl Tab 20 MG |
| 54868235200 | Benazepril HCl | Benazepril HCl Tab 40 MG |
| 54868235201 | Benazepril HCl | Benazepril HCl Tab 40 MG |
| 54868369001 | Benazepril HCl | Benazepril HCl Tab 5 MG |
| 00185082001 | Benazepril HCl | Benazepril HCl Tab 20 MG |
| 00185082005 | Benazepril HCl | Benazepril HCl Tab 20 MG |
| 00185004801 | Benazepril HCl | Benazepril HCl Tab 40 MG |
| 00185004805 | Benazepril HCl | Benazepril HCl Tab 40 MG |
| 00185005301 | Benazepril HCl | Benazepril HCl Tab 10 MG |
| 00185005305 | Benazepril HCl | Benazepril HCl Tab 10 MG |
| 00378044101 | Benazepril HCl | Benazepril HCl Tab 5 MG |
| 00378044301 | Benazepril HCl | Benazepril HCl Tab 10 MG |
| 00378044401 | Benazepril HCl | Benazepril HCl Tab 20 MG |
| 00378044701 | Benazepril HCl | Benazepril HCl Tab 40 MG |
| 00781189101 | Benazepril HCl | Benazepril HCl Tab 5 MG |
| 00781189201 | Benazepril HCl | Benazepril HCl Tab 10 MG |
| 00781189301 | Benazepril HCl | Benazepril HCl Tab 20 MG |
| 00781189401 | Benazepril HCl | Benazepril HCl Tab 40 MG |
| 12280003300 | Benazepril HCl | Benazepril HCl Tab 40 MG |
| 49999028730 | Benazepril HCl | Benazepril HCl Tab 20 MG |
| 00083005930 | Benazepril HCl | Benazepril HCl Tab 5 MG |
| 00083005932 | Benazepril HCl | Benazepril HCl Tab 5 MG |
| 00083005990 | Benazepril HCl | Benazepril HCl Tab 5 MG |
| 00083006330 | Benazepril HCl | Benazepril HCl Tab 10 MG |
| 00083006332 | Benazepril HCl | Benazepril HCl Tab 10 MG |
| 00083006390 | Benazepril HCl | Benazepril HCl Tab 10 MG |
| 00083007930 | Benazepril HCl | Benazepril HCl Tab 20 MG |
| 00083007932 | Benazepril HCl | Benazepril HCl Tab 20 MG |
| 00083007990 | Benazepril HCl | Benazepril HCl Tab 20 MG |
| 00083009430 | Benazepril HCl | Benazepril HCl Tab 40 MG |
| 00083009432 | Benazepril HCl | Benazepril HCl Tab 40 MG |
| 00083009490 | Benazepril HCl | Benazepril HCl Tab 40 MG |
| 00078044705 | Benazepril HCl | Benazepril HCl Tab 5 MG |
| 00078044805 | Benazepril HCl | Benazepril HCl Tab 10 MG |
| 00078044905 | Benazepril HCl | Benazepril HCl Tab 20 MG |
| 00078045005 | Benazepril HCl | Benazepril HCl Tab 40 MG |
| 00093512401 | Benazepril HCl | Benazepril HCl Tab 5 MG |
| 00093512501 | Benazepril HCl | Benazepril HCl Tab 10 MG |
| 00093512505 | Benazepril HCl | Benazepril HCl Tab 10 MG |
| 00093512601 | Benazepril HCl | Benazepril HCl Tab 20 MG |
| 00093512605 | Benazepril HCl | Benazepril HCl Tab 20 MG |
| 00093512701 | Benazepril HCl | Benazepril HCl Tab 40 MG |
| 00172535060 | Benazepril HCl | Benazepril HCl Tab 5 MG |
| 00172535070 | Benazepril HCl | Benazepril HCl Tab 5 MG |
| 00172535110 | Benazepril HCl | Benazepril HCl Tab 10 MG |
| 00172535160 | Benazepril HCl | Benazepril HCl Tab 10 MG |
| 00172535170 | Benazepril HCl | Benazepril HCl Tab 10 MG |
| 00172535180 | Benazepril HCl | Benazepril HCl Tab 10 MG |
| 00172535210 | Benazepril HCl | Benazepril HCl Tab 20 MG |
| 00172535260 | Benazepril HCl | Benazepril HCl Tab 20 MG |
| 00172535270 | Benazepril HCl | Benazepril HCl Tab 20 MG |
| 00172535280 | Benazepril HCl | Benazepril HCl Tab 20 MG |
| 00172535310 | Benazepril HCl | Benazepril HCl Tab 40 MG |
| 00172535360 | Benazepril HCl | Benazepril HCl Tab 40 MG |
| 00172535370 | Benazepril HCl | Benazepril HCl Tab 40 MG |
| 00172535380 | Benazepril HCl | Benazepril HCl Tab 40 MG |
| 00185050501 | Benazepril HCl | Benazepril HCl Tab 5 MG |
| 00185050505 | Benazepril HCl | Benazepril HCl Tab 5 MG |
| 00003076951 | Bendroflumethiazide/Rauwolfia | Bendroflumethiazide & Rauwolfia Tab 4-50 MG |
| 00003076980 | Bendroflumethiazide/Rauwolfia | Bendroflumethiazide & Rauwolfia Tab 4-50 MG |
| 00304188401 | Bendroflumethiazide/Rauwolfia | Bendroflumethiazide & Rauwolfia Tab 4-50 MG |
| 00536450201 | Bendroflumethiazide/Rauwolfia | Bendroflumethiazide & Rauwolfia Tab 4-50 MG |
| 00677120101 | Bendroflumethiazide/Rauwolfia | Bendroflumethiazide & Rauwolfia Tab 4-50 MG |
| 57664031508 | Bendroflumethiazide/Rauwolfia | Bendroflumethiazide & Rauwolfia Tab 4-50 MG |
| 00904253760 | Bendroflumethiazide/Rauwolfia | Bendroflumethiazide & Rauwolfia Tab 4-50 MG |
| 47202284201 | Bendroflumethiazide/Rauwolfia | Bendroflumethiazide & Rauwolfia Tab 4-50 MG |
| 51991053501 | Bendroflumethiazide/Rauwolfia | Bendroflumethiazide & Rauwolfia Tab 4-50 MG |
| 54569058700 | Bendroflumethiazide/Rauwolfia | Bendroflumethiazide & Rauwolfia Tab 4-50 MG |
| 55053006901 | Bendroflumethiazide/Rauwolfia | Bendroflumethiazide & Rauwolfia Tab 4-50 MG |
| 00839740806 | Bendroflumethiazide/Rauwolfia | Bendroflumethiazide & Rauwolfia Tab 4-50 MG |
| 51285005002 | Bisoprolol & Hydrochlorothiazide | Bisoprolol & Hydrochlorothiazide Tab 5-6.25 MG |
| 00005323423 | Bisoprolol & Hydrochlorothiazide | Bisoprolol & Hydrochlorothiazide Tab 5-6.25 MG |
| 00005323538 | Bisoprolol & Hydrochlorothiazide | Bisoprolol & Hydrochlorothiazide Tab 10-6.25 MG |
| 00005323823 | Bisoprolol & Hydrochlorothiazide | Bisoprolol & Hydrochlorothiazide Tab 2.5-6.25 MG |
| 00172573060 | Bisoprolol & Hydrochlorothiazide | Bisoprolol & Hydrochlorothiazide Tab 2.5-6.25 MG |
| 00172573070 | Bisoprolol & Hydrochlorothiazide | Bisoprolol & Hydrochlorothiazide Tab 2.5-6.25 MG |
| 00172573160 | Bisoprolol & Hydrochlorothiazide | Bisoprolol & Hydrochlorothiazide Tab 5-6.25 MG |
| 00172573170 | Bisoprolol & Hydrochlorothiazide | Bisoprolol & Hydrochlorothiazide Tab 5-6.25 MG |
| 00172573260 | Bisoprolol & Hydrochlorothiazide | Bisoprolol & Hydrochlorothiazide Tab 10-6.25 MG |
| 00172573270 | Bisoprolol & Hydrochlorothiazide | Bisoprolol & Hydrochlorothiazide Tab 10-6.25 MG |
| 00185070101 | Bisoprolol & Hydrochlorothiazide | Bisoprolol & Hydrochlorothiazide Tab 2.5-6.25 MG |
| 00185070105 | Bisoprolol & Hydrochlorothiazide | Bisoprolol & Hydrochlorothiazide Tab 2.5-6.25 MG |
| 00185070130 | Bisoprolol & Hydrochlorothiazide | Bisoprolol & Hydrochlorothiazide Tab 2.5-6.25 MG |
| 55887062560 | Bisoprolol & Hydrochlorothiazide | Bisoprolol & Hydrochlorothiazide Tab 10-6.25 MG |
| 55887062590 | Bisoprolol & Hydrochlorothiazide | Bisoprolol & Hydrochlorothiazide Tab 10-6.25 MG |
| 58864078430 | Bisoprolol & Hydrochlorothiazide | Bisoprolol & Hydrochlorothiazide Tab 5-6.25 MG |
| 59911706502 | Bisoprolol & Hydrochlorothiazide | Bisoprolol & Hydrochlorothiazide Tab 2.5-6.25 MG |
| 59911706602 | Bisoprolol & Hydrochlorothiazide | Bisoprolol & Hydrochlorothiazide Tab 5-6.25 MG |
| 59911706701 | Bisoprolol & Hydrochlorothiazide | Bisoprolol & Hydrochlorothiazide Tab 10-6.25 MG |
| 51079095401 | Bisoprolol & Hydrochlorothiazide | Bisoprolol & Hydrochlorothiazide Tab 2.5-6.25 MG |
| 51079095420 | Bisoprolol & Hydrochlorothiazide | Bisoprolol & Hydrochlorothiazide Tab 2.5-6.25 MG |
| 51079095501 | Bisoprolol & Hydrochlorothiazide | Bisoprolol & Hydrochlorothiazide Tab 5-6.25 MG |
| 51079095520 | Bisoprolol & Hydrochlorothiazide | Bisoprolol & Hydrochlorothiazide Tab 5-6.25 MG |
| 51079095601 | Bisoprolol & Hydrochlorothiazide | Bisoprolol & Hydrochlorothiazide Tab 10-6.25 MG |
| 51079095620 | Bisoprolol & Hydrochlorothiazide | Bisoprolol & Hydrochlorothiazide Tab 10-6.25 MG |
| 52544084101 | Bisoprolol & Hydrochlorothiazide | Bisoprolol & Hydrochlorothiazide Tab 2.5-6.25 MG |
| 52544084105 | Bisoprolol & Hydrochlorothiazide | Bisoprolol & Hydrochlorothiazide Tab 2.5-6.25 MG |
| 52544084201 | Bisoprolol & Hydrochlorothiazide | Bisoprolol & Hydrochlorothiazide Tab 5-6.25 MG |
| 52544084205 | Bisoprolol & Hydrochlorothiazide | Bisoprolol & Hydrochlorothiazide Tab 5-6.25 MG |
| 52544084330 | Bisoprolol & Hydrochlorothiazide | Bisoprolol & Hydrochlorothiazide Tab 10-6.25 MG |
| 52959024130 | Bisoprolol & Hydrochlorothiazide | Bisoprolol & Hydrochlorothiazide Tab 10-6.25 MG |
| 52959033710 | Bisoprolol & Hydrochlorothiazide | Bisoprolol & Hydrochlorothiazide Tab 5-6.25 MG |
| 52959033730 | Bisoprolol & Hydrochlorothiazide | Bisoprolol & Hydrochlorothiazide Tab 5-6.25 MG |
| 54569470700 | Bisoprolol & Hydrochlorothiazide | Bisoprolol & Hydrochlorothiazide Tab 2.5-6.25 MG |
| 54569470800 | Bisoprolol & Hydrochlorothiazide | Bisoprolol & Hydrochlorothiazide Tab 5-6.25 MG |
| 54569536800 | Bisoprolol & Hydrochlorothiazide | Bisoprolol & Hydrochlorothiazide Tab 10-6.25 MG |
| 54569540400 | Bisoprolol & Hydrochlorothiazide | Bisoprolol & Hydrochlorothiazide Tab 5-6.25 MG |
| 54569541700 | Bisoprolol & Hydrochlorothiazide | Bisoprolol & Hydrochlorothiazide Tab 2.5-6.25 MG |
| 54569541900 | Bisoprolol & Hydrochlorothiazide | Bisoprolol & Hydrochlorothiazide Tab 10-6.25 MG |
| 54868417300 | Bisoprolol & Hydrochlorothiazide | Bisoprolol & Hydrochlorothiazide Tab 5-6.25 MG |
| 54868417900 | Bisoprolol & Hydrochlorothiazide | Bisoprolol & Hydrochlorothiazide Tab 10-6.25 MG |
| 54868457600 | Bisoprolol & Hydrochlorothiazide | Bisoprolol & Hydrochlorothiazide Tab 5-6.25 MG |
| 54868457601 | Bisoprolol & Hydrochlorothiazide | Bisoprolol & Hydrochlorothiazide Tab 5-6.25 MG |
| 54868457701 | Bisoprolol & Hydrochlorothiazide | Bisoprolol & Hydrochlorothiazide Tab 2.5-6.25 MG |
| 54868457800 | Bisoprolol & Hydrochlorothiazide | Bisoprolol & Hydrochlorothiazide Tab 10-6.25 MG |
| 54868457801 | Bisoprolol & Hydrochlorothiazide | Bisoprolol & Hydrochlorothiazide Tab 10-6.25 MG |
| 00185070401 | Bisoprolol & Hydrochlorothiazide | Bisoprolol & Hydrochlorothiazide Tab 5-6.25 MG |
| 00185070405 | Bisoprolol & Hydrochlorothiazide | Bisoprolol & Hydrochlorothiazide Tab 5-6.25 MG |
| 00185070430 | Bisoprolol & Hydrochlorothiazide | Bisoprolol & Hydrochlorothiazide Tab 5-6.25 MG |
| 00185070701 | Bisoprolol & Hydrochlorothiazide | Bisoprolol & Hydrochlorothiazide Tab 10-6.25 MG |
| 00185070705 | Bisoprolol & Hydrochlorothiazide | Bisoprolol & Hydrochlorothiazide Tab 10-6.25 MG |
| 00185070730 | Bisoprolol & Hydrochlorothiazide | Bisoprolol & Hydrochlorothiazide Tab 10-6.25 MG |
| 00228265010 | Bisoprolol & Hydrochlorothiazide | Bisoprolol & Hydrochlorothiazide Tab 2.5-6.25 MG |
| 00228265110 | Bisoprolol & Hydrochlorothiazide | Bisoprolol & Hydrochlorothiazide Tab 5-6.25 MG |
| 00228265203 | Bisoprolol & Hydrochlorothiazide | Bisoprolol & Hydrochlorothiazide Tab 10-6.25 MG |
| 00378050101 | Bisoprolol & Hydrochlorothiazide | Bisoprolol & Hydrochlorothiazide Tab 2.5-6.25 MG |
| 00378050110 | Bisoprolol & Hydrochlorothiazide | Bisoprolol & Hydrochlorothiazide Tab 2.5-6.25 MG |
| 00378050301 | Bisoprolol & Hydrochlorothiazide | Bisoprolol & Hydrochlorothiazide Tab 5-6.25 MG |
| 00378050310 | Bisoprolol & Hydrochlorothiazide | Bisoprolol & Hydrochlorothiazide Tab 5-6.25 MG |
| 00378050501 | Bisoprolol & Hydrochlorothiazide | Bisoprolol & Hydrochlorothiazide Tab 10-6.25 MG |
| 00378050505 | Bisoprolol & Hydrochlorothiazide | Bisoprolol & Hydrochlorothiazide Tab 10-6.25 MG |
| 00591084101 | Bisoprolol & Hydrochlorothiazide | Bisoprolol & Hydrochlorothiazide Tab 2.5-6.25 MG |
| 00591084105 | Bisoprolol & Hydrochlorothiazide | Bisoprolol & Hydrochlorothiazide Tab 2.5-6.25 MG |
| 00591084201 | Bisoprolol & Hydrochlorothiazide | Bisoprolol & Hydrochlorothiazide Tab 5-6.25 MG |
| 00591084205 | Bisoprolol & Hydrochlorothiazide | Bisoprolol & Hydrochlorothiazide Tab 5-6.25 MG |
| 00591084301 | Bisoprolol & Hydrochlorothiazide | Bisoprolol & Hydrochlorothiazide Tab 10-6.25 MG |
| 00591084330 | Bisoprolol & Hydrochlorothiazide | Bisoprolol & Hydrochlorothiazide Tab 10-6.25 MG |
| 00781182401 | Bisoprolol & Hydrochlorothiazide | Bisoprolol & Hydrochlorothiazide Tab 5-6.25 MG |
| 00781182405 | Bisoprolol & Hydrochlorothiazide | Bisoprolol & Hydrochlorothiazide Tab 5-6.25 MG |
| 00781183301 | Bisoprolol & Hydrochlorothiazide | Bisoprolol & Hydrochlorothiazide Tab 10-6.25 MG |
| 00781183305 | Bisoprolol & Hydrochlorothiazide | Bisoprolol & Hydrochlorothiazide Tab 10-6.25 MG |
| 00781184101 | Bisoprolol & Hydrochlorothiazide | Bisoprolol & Hydrochlorothiazide Tab 2.5-6.25 MG |
| 00781184105 | Bisoprolol & Hydrochlorothiazide | Bisoprolol & Hydrochlorothiazide Tab 2.5-6.25 MG |
| 51285004001 | Bisoprolol & Hydrochlorothiazide | Bisoprolol & Hydrochlorothiazide Tab 10-6.25 MG |
| 51285004702 | Bisoprolol & Hydrochlorothiazide | Bisoprolol & Hydrochlorothiazide Tab 2.5-6.25 MG |
| 51285004802 | Bisoprolol & Hydrochlorothiazide | Bisoprolol & Hydrochlorothiazide Tab 5-6.25 MG |
| 51285004901 | Bisoprolol & Hydrochlorothiazide | Bisoprolol & Hydrochlorothiazide Tab 10-6.25 MG |
| 51138050230 | Bisoprolol & Hydrochlorothiazide | Bisoprolol & Hydrochlorothiazide Tab 2.5-6.25 MG |
| 51138050330 | Bisoprolol & Hydrochlorothiazide | Bisoprolol & Hydrochlorothiazide Tab 5-6.25 MG |
| 51138050430 | Bisoprolol & Hydrochlorothiazide | Bisoprolol & Hydrochlorothiazide Tab 10-6.25 MG |
| 66336061230 | Bisoprolol & Hydrochlorothiazide | Bisoprolol & Hydrochlorothiazide Tab 10-6.25 MG |
| 68258606303 | Bisoprolol & Hydrochlorothiazide | Bisoprolol & Hydrochlorothiazide Tab 2.5-6.25 MG |
| 68258606403 | Bisoprolol & Hydrochlorothiazide | Bisoprolol & Hydrochlorothiazide Tab 5-6.25 MG |
| 63629167401 | Bisoprolol & Hydrochlorothiazide | Bisoprolol & Hydrochlorothiazide Tab 2.5-6.25 MG |
| 42291017190 | Bisoprolol & Hydrochlorothiazide | Bisoprolol & Hydrochlorothiazide Tab 2.5-6.25 MG |
| 42291017290 | Bisoprolol & Hydrochlorothiazide | Bisoprolol & Hydrochlorothiazide Tab 5-6.25 MG |
| 42291017390 | Bisoprolol & Hydrochlorothiazide | Bisoprolol & Hydrochlorothiazide Tab 10-6.25 MG |
| 63629407201 | Bisoprolol & Hydrochlorothiazide | Bisoprolol & Hydrochlorothiazide Tab 5-6.25 MG |
| 63629407501 | Bisoprolol & Hydrochlorothiazide | Bisoprolol & Hydrochlorothiazide Tab 10-6.25 MG |
| 29300018701 | Bisoprolol & Hydrochlorothiazide | Bisoprolol & Hydrochlorothiazide Tab 2.5-6.25 MG |
| 29300018705 | Bisoprolol & Hydrochlorothiazide | Bisoprolol & Hydrochlorothiazide Tab 2.5-6.25 MG |
| 29300018713 | Bisoprolol & Hydrochlorothiazide | Bisoprolol & Hydrochlorothiazide Tab 2.5-6.25 MG |
| 29300018801 | Bisoprolol & Hydrochlorothiazide | Bisoprolol & Hydrochlorothiazide Tab 5-6.25 MG |
| 29300018805 | Bisoprolol & Hydrochlorothiazide | Bisoprolol & Hydrochlorothiazide Tab 5-6.25 MG |
| 29300018813 | Bisoprolol & Hydrochlorothiazide | Bisoprolol & Hydrochlorothiazide Tab 5-6.25 MG |
| 29300018901 | Bisoprolol & Hydrochlorothiazide | Bisoprolol & Hydrochlorothiazide Tab 10-6.25 MG |
| 29300018905 | Bisoprolol & Hydrochlorothiazide | Bisoprolol & Hydrochlorothiazide Tab 10-6.25 MG |
| 29300018913 | Bisoprolol & Hydrochlorothiazide | Bisoprolol & Hydrochlorothiazide Tab 10-6.25 MG |
| 43063013430 | Bisoprolol & Hydrochlorothiazide | Bisoprolol & Hydrochlorothiazide Tab 10-6.25 MG |
| 54868457602 | Bisoprolol & Hydrochlorothiazide | Bisoprolol & Hydrochlorothiazide Tab 5-6.25 MG |
| 51138000930 | Bisoprolol & Hydrochlorothiazide | Bisoprolol & Hydrochlorothiazide Tab 2.5-6.25 MG |
| 51138001030 | Bisoprolol & Hydrochlorothiazide | Bisoprolol & Hydrochlorothiazide Tab 5-6.25 MG |
| 51138001130 | Bisoprolol & Hydrochlorothiazide | Bisoprolol & Hydrochlorothiazide Tab 10-6.25 MG |
| 21695080830 | Bisoprolol & Hydrochlorothiazide | Bisoprolol & Hydrochlorothiazide Tab 5-6.25 MG |
| 21695080930 | Bisoprolol & Hydrochlorothiazide | Bisoprolol & Hydrochlorothiazide Tab 10-6.25 MG |
| 66336061290 | Bisoprolol & Hydrochlorothiazide | Bisoprolol & Hydrochlorothiazide Tab 10-6.25 MG |
| 43063013490 | Bisoprolol & Hydrochlorothiazide | Bisoprolol & Hydrochlorothiazide Tab 10-6.25 MG |
| 43063013590 | Bisoprolol & Hydrochlorothiazide | Bisoprolol & Hydrochlorothiazide Tab 2.5-6.25 MG |
| 55289063090 | Bisoprolol & Hydrochlorothiazide | Bisoprolol & Hydrochlorothiazide Tab 5-6.25 MG |
| 54868457603 | Bisoprolol & Hydrochlorothiazide | Bisoprolol & Hydrochlorothiazide Tab 5-6.25 MG |
| 54868457703 | Bisoprolol & Hydrochlorothiazide | Bisoprolol & Hydrochlorothiazide Tab 2.5-6.25 MG |
| 54868457803 | Bisoprolol & Hydrochlorothiazide | Bisoprolol & Hydrochlorothiazide Tab 10-6.25 MG |
| 00781182431 | Bisoprolol & Hydrochlorothiazide | Bisoprolol & Hydrochlorothiazide Tab 5-6.25 MG |
| 00781183331 | Bisoprolol & Hydrochlorothiazide | Bisoprolol & Hydrochlorothiazide Tab 10-6.25 MG |
| 00781184131 | Bisoprolol & Hydrochlorothiazide | Bisoprolol & Hydrochlorothiazide Tab 2.5-6.25 MG |
| 67544105530 | Bisoprolol & Hydrochlorothiazide | Bisoprolol & Hydrochlorothiazide Tab 5-6.25 MG |
| 23490650403 | Bisoprolol & Hydrochlorothiazide | Bisoprolol & Hydrochlorothiazide Tab 5-6.25 MG |
| 23490650406 | Bisoprolol & Hydrochlorothiazide | Bisoprolol & Hydrochlorothiazide Tab 5-6.25 MG |
| 23490650409 | Bisoprolol & Hydrochlorothiazide | Bisoprolol & Hydrochlorothiazide Tab 5-6.25 MG |
| 54569540401 | Bisoprolol & Hydrochlorothiazide | Bisoprolol & Hydrochlorothiazide Tab 5-6.25 MG |
| 54569541701 | Bisoprolol & Hydrochlorothiazide | Bisoprolol & Hydrochlorothiazide Tab 2.5-6.25 MG |
| 54569541901 | Bisoprolol & Hydrochlorothiazide | Bisoprolol & Hydrochlorothiazide Tab 10-6.25 MG |
| 54868457802 | Bisoprolol & Hydrochlorothiazide | Bisoprolol & Hydrochlorothiazide Tab 10-6.25 MG |
| 00490005300 | Bisoprolol & Hydrochlorothiazide | Bisoprolol & Hydrochlorothiazide Tab 2.5-6.25 MG |
| 00490005330 | Bisoprolol & Hydrochlorothiazide | Bisoprolol & Hydrochlorothiazide Tab 2.5-6.25 MG |
| 00490005360 | Bisoprolol & Hydrochlorothiazide | Bisoprolol & Hydrochlorothiazide Tab 2.5-6.25 MG |
| 00490005390 | Bisoprolol & Hydrochlorothiazide | Bisoprolol & Hydrochlorothiazide Tab 2.5-6.25 MG |
| 23490650303 | Bisoprolol & Hydrochlorothiazide | Bisoprolol & Hydrochlorothiazide Tab 10-6.25 MG |
| 23490786103 | Bisoprolol & Hydrochlorothiazide | Bisoprolol & Hydrochlorothiazide Tab 2.5-6.25 MG |
| 54868457700 | Bisoprolol & Hydrochlorothiazide | Bisoprolol & Hydrochlorothiazide Tab 2.5-6.25 MG |
| 58016028600 | Bisoprolol & Hydrochlorothiazide | Bisoprolol & Hydrochlorothiazide Tab 5-6.25 MG |
| 58016028602 | Bisoprolol & Hydrochlorothiazide | Bisoprolol & Hydrochlorothiazide Tab 5-6.25 MG |
| 58016028630 | Bisoprolol & Hydrochlorothiazide | Bisoprolol & Hydrochlorothiazide Tab 5-6.25 MG |
| 58016028660 | Bisoprolol & Hydrochlorothiazide | Bisoprolol & Hydrochlorothiazide Tab 5-6.25 MG |
| 58016028690 | Bisoprolol & Hydrochlorothiazide | Bisoprolol & Hydrochlorothiazide Tab 5-6.25 MG |
| 55289063030 | Bisoprolol & Hydrochlorothiazide | Bisoprolol & Hydrochlorothiazide Tab 5-6.25 MG |
| 55887026730 | Bisoprolol & Hydrochlorothiazide | Bisoprolol & Hydrochlorothiazide Tab 2.5-6.25 MG |
| 55887026760 | Bisoprolol & Hydrochlorothiazide | Bisoprolol & Hydrochlorothiazide Tab 2.5-6.25 MG |
| 55887026782 | Bisoprolol & Hydrochlorothiazide | Bisoprolol & Hydrochlorothiazide Tab 2.5-6.25 MG |
| 55887026790 | Bisoprolol & Hydrochlorothiazide | Bisoprolol & Hydrochlorothiazide Tab 2.5-6.25 MG |
| 55887062530 | Bisoprolol & Hydrochlorothiazide | Bisoprolol & Hydrochlorothiazide Tab 10-6.25 MG |
| 00186000431 | Candesartan Cilexetil | Candesartan Cilexetil Tab 4 MG |
| 35356042730 | Candesartan Cilexetil | Candesartan Cilexetil Tab 8 MG |
| 00186001628 | Candesartan Cilexetil | Candesartan Cilexetil Tab 16 MG |
| 00186001631 | Candesartan Cilexetil | Candesartan Cilexetil Tab 16 MG |
| 00186001654 | Candesartan Cilexetil | Candesartan Cilexetil Tab 16 MG |
| 00186003228 | Candesartan Cilexetil | Candesartan Cilexetil Tab 32 MG |
| 00186003231 | Candesartan Cilexetil | Candesartan Cilexetil Tab 32 MG |
| 00186003254 | Candesartan Cilexetil | Candesartan Cilexetil Tab 32 MG |
| 00186000831 | Candesartan Cilexetil | Candesartan Cilexetil Tab 8 MG |
| 12280034030 | Candesartan Cilexetil | Candesartan Cilexetil Tab 32 MG |
| 63629337604 | Candesartan Cilexetil | Candesartan Cilexetil Tab 16 MG |
| 63629337603 | Candesartan Cilexetil | Candesartan Cilexetil Tab 16 MG |
| 63629337602 | Candesartan Cilexetil | Candesartan Cilexetil Tab 16 MG |
| 63629337601 | Candesartan Cilexetil | Candesartan Cilexetil Tab 16 MG |
| 51138049430 | Candesartan Cilexetil | Candesartan Cilexetil Tab 32 MG |
| 51138049330 | Candesartan Cilexetil | Candesartan Cilexetil Tab 16 MG |
| 61113003231 | Candesartan Cilexetil | Candesartan Cilexetil Tab 32 MG |
| 61113003228 | Candesartan Cilexetil | Candesartan Cilexetil Tab 32 MG |
| 61113001631 | Candesartan Cilexetil | Candesartan Cilexetil Tab 16 MG |
| 61113001628 | Candesartan Cilexetil | Candesartan Cilexetil Tab 16 MG |
| 61113000831 | Candesartan Cilexetil | Candesartan Cilexetil Tab 8 MG |
| 61113000431 | Candesartan Cilexetil | Candesartan Cilexetil Tab 4 MG |
| 54868559100 | Candesartan Cilexetil | Candesartan Cilexetil Tab 4 MG |
| 49999030530 | Candesartan Cilexetil | Candesartan Cilexetil Tab 16 MG |
| 54868461200 | Candesartan Cilexetil | Candesartan Cilexetil Tab 32 MG |
| 54868441300 | Candesartan Cilexetil | Candesartan Cilexetil Tab 16 MG |
| 54569471400 | Candesartan Cilexetil | Candesartan Cilexetil Tab 16 MG |
| 54569471900 | Candesartan Cilexetil | Candesartan Cilexetil Tab 8 MG |
| 54569471901 | Candesartan Cilexetil | Candesartan Cilexetil Tab 8 MG |
| 67544017745 | Candesartan Cilexetil | Candesartan Cilexetil Tab 32 MG |
| 67544021245 | Candesartan Cilexetil | Candesartan Cilexetil Tab 16 MG |
| 67544021253 | Candesartan Cilexetil | Candesartan Cilexetil Tab 16 MG |
| 49999098830 | Candesartan Cilexetil | Candesartan Cilexetil Tab 32 MG |
| 54868548900 | Candesartan Cilexetil | Candesartan Cilexetil Tab 8 MG |
| 63629337701 | Candesartan Cilexetil-Hydrochlorothiazide | Candesartan Cilexetil-Hydrochlorothiazide Tab 32-12.5 MG |
| 00186016228 | Candesartan Cilexetil-Hydrochlorothiazide | Candesartan Cilexetil-Hydrochlorothiazide Tab 16-12.5 MG |
| 68258605803 | Candesartan Cilexetil-Hydrochlorothiazide | Candesartan Cilexetil-Hydrochlorothiazide Tab 32-12.5 MG |
| 68115070390 | Candesartan Cilexetil-Hydrochlorothiazide | Candesartan Cilexetil-Hydrochlorothiazide Tab 32-12.5 MG |
| 63629337704 | Candesartan Cilexetil-Hydrochlorothiazide | Candesartan Cilexetil-Hydrochlorothiazide Tab 32-12.5 MG |
| 63629337703 | Candesartan Cilexetil-Hydrochlorothiazide | Candesartan Cilexetil-Hydrochlorothiazide Tab 32-12.5 MG |
| 54868472900 | Candesartan Cilexetil-Hydrochlorothiazide | Candesartan Cilexetil-Hydrochlorothiazide Tab 16-12.5 MG |
| 54569580100 | Candesartan Cilexetil-Hydrochlorothiazide | Candesartan Cilexetil-Hydrochlorothiazide Tab 32-12.5 MG |
| 63629337702 | Candesartan Cilexetil-Hydrochlorothiazide | Candesartan Cilexetil-Hydrochlorothiazide Tab 32-12.5 MG |
| 68115070330 | Candesartan Cilexetil-Hydrochlorothiazide | Candesartan Cilexetil-Hydrochlorothiazide Tab 32-12.5 MG |
| 54868486900 | Candesartan Cilexetil-Hydrochlorothiazide | Candesartan Cilexetil-Hydrochlorothiazide Tab 32-12.5 MG |
| 00186032254 | Candesartan Cilexetil-Hydrochlorothiazide | Candesartan Cilexetil-Hydrochlorothiazide Tab 32-12.5 MG |
| 00186032228 | Candesartan Cilexetil-Hydrochlorothiazide | Candesartan Cilexetil-Hydrochlorothiazide Tab 32-12.5 MG |
| 00186016254 | Candesartan Cilexetil-Hydrochlorothiazide | Candesartan Cilexetil-Hydrochlorothiazide Tab 16-12.5 MG |
| 00186032454 | Candesartan Cilexetil-Hydrochlorothiazide | Candesartan Cilexetil-Hydrochlorothiazide Tab 32-25 MG |
| 00003048250 | Captopril | Captopril Tab 50 MG |
| 00003048251 | Captopril | Captopril Tab 50 MG |
| 00003048275 | Captopril | Captopril Tab 50 MG |
| 00003048550 | Captopril | Captopril Tab 100 MG |
| 00093813201 | Captopril | Captopril Tab 12.5 MG |
| 00093813210 | Captopril | Captopril Tab 12.5 MG |
| 00093813301 | Captopril | Captopril Tab 25 MG |
| 00093813310 | Captopril | Captopril Tab 25 MG |
| 00093813401 | Captopril | Captopril Tab 50 MG |
| 00093813410 | Captopril | Captopril Tab 50 MG |
| 00093813501 | Captopril | Captopril Tab 100 MG |
| 00093009101 | Captopril | Captopril Tab 12.5 MG |
| 00093009110 | Captopril | Captopril Tab 12.5 MG |
| 00093009201 | Captopril | Captopril Tab 25 MG |
| 00093009210 | Captopril | Captopril Tab 25 MG |
| 00093009701 | Captopril | Captopril Tab 50 MG |
| 00093009710 | Captopril | Captopril Tab 50 MG |
| 00093009801 | Captopril | Captopril Tab 100 MG |
| 00143117101 | Captopril | Captopril Tab 12.5 MG |
| 00143117110 | Captopril | Captopril Tab 12.5 MG |
| 00143117125 | Captopril | Captopril Tab 12.5 MG |
| 00143117201 | Captopril | Captopril Tab 25 MG |
| 00143117210 | Captopril | Captopril Tab 25 MG |
| 00143117225 | Captopril | Captopril Tab 25 MG |
| 00143117301 | Captopril | Captopril Tab 50 MG |
| 00143117310 | Captopril | Captopril Tab 50 MG |
| 00143117325 | Captopril | Captopril Tab 50 MG |
| 00143117401 | Captopril | Captopril Tab 100 MG |
| 00143117425 | Captopril | Captopril Tab 100 MG |
| 00182262389 | Captopril | Captopril Tab 25 MG |
| 00904504740 | Captopril | Captopril Tab 50 MG |
| 00904504760 | Captopril | Captopril Tab 50 MG |
| 00904504761 | Captopril | Captopril Tab 50 MG |
| 00904504780 | Captopril | Captopril Tab 50 MG |
| 00904504860 | Captopril | Captopril Tab 100 MG |
| 38245031210 | Captopril | Captopril Tab 50 MG |
| 38245031220 | Captopril | Captopril Tab 50 MG |
| 38245047110 | Captopril | Captopril Tab 25 MG |
| 38245047120 | Captopril | Captopril Tab 25 MG |
| 38245065310 | Captopril | Captopril Tab 100 MG |
| 38245074310 | Captopril | Captopril Tab 12.5 MG |
| 38245074320 | Captopril | Captopril Tab 12.5 MG |
| 44514067499 | Captopril | Captopril Tab 12.5 MG |
| 44514067599 | Captopril | Captopril Tab 25 MG |
| 44514067699 | Captopril | Captopril Tab 50 MG |
| 49884061901 | Captopril | Captopril Tab 12.5 MG |
| 49884061910 | Captopril | Captopril Tab 12.5 MG |
| 49884062001 | Captopril | Captopril Tab 25 MG |
| 49884062010 | Captopril | Captopril Tab 25 MG |
| 49884062101 | Captopril | Captopril Tab 50 MG |
| 49884062110 | Captopril | Captopril Tab 50 MG |
| 49884062201 | Captopril | Captopril Tab 100 MG |
| 49884079301 | Captopril | Captopril Tab 12.5 MG |
| 49884079374 | Captopril | Captopril Tab 12.5 MG |
| 49884079401 | Captopril | Captopril Tab 25 MG |
| 00247122200 | Captopril | Captopril Tab 50 MG |
| 00378300701 | Captopril | Captopril Tab 12.5 MG |
| 00378300710 | Captopril | Captopril Tab 12.5 MG |
| 00378301201 | Captopril | Captopril Tab 25 MG |
| 00378301210 | Captopril | Captopril Tab 25 MG |
| 00378301701 | Captopril | Captopril Tab 50 MG |
| 00378301710 | Captopril | Captopril Tab 50 MG |
| 00378302201 | Captopril | Captopril Tab 100 MG |
| 00440723060 | Captopril | Captopril Tab 12.5 MG |
| 00440723090 | Captopril | Captopril Tab 12.5 MG |
| 00440723092 | Captopril | Captopril Tab 12.5 MG |
| 00440723094 | Captopril | Captopril Tab 12.5 MG |
| 00440723130 | Captopril | Captopril Tab 25 MG |
| 00440723160 | Captopril | Captopril Tab 25 MG |
| 00440723190 | Captopril | Captopril Tab 25 MG |
| 00440723191 | Captopril | Captopril Tab 25 MG |
| 00440723192 | Captopril | Captopril Tab 25 MG |
| 00440723194 | Captopril | Captopril Tab 25 MG |
| 00440723230 | Captopril | Captopril Tab 50 MG |
| 00440723260 | Captopril | Captopril Tab 50 MG |
| 00440723290 | Captopril | Captopril Tab 50 MG |
| 00440723291 | Captopril | Captopril Tab 50 MG |
| 00440723292 | Captopril | Captopril Tab 50 MG |
| 00440723294 | Captopril | Captopril Tab 50 MG |
| 00003045051 | Captopril | Captopril Tab 12.5 MG |
| 00003045054 | Captopril | Captopril Tab 12.5 MG |
| 00003045075 | Captopril | Captopril Tab 12.5 MG |
| 00003045250 | Captopril | Captopril Tab 25 MG |
| 00003045251 | Captopril | Captopril Tab 25 MG |
| 00003045275 | Captopril | Captopril Tab 25 MG |
| 54569424703 | Captopril | Captopril Tab 50 MG |
| 54868066901 | Captopril | Captopril Tab 25 MG |
| 54868066902 | Captopril | Captopril Tab 25 MG |
| 54868066903 | Captopril | Captopril Tab 25 MG |
| 54868066905 | Captopril | Captopril Tab 25 MG |
| 54868066906 | Captopril | Captopril Tab 25 MG |
| 54868141501 | Captopril | Captopril Tab 50 MG |
| 54868177501 | Captopril | Captopril Tab 12.5 MG |
| 54868177504 | Captopril | Captopril Tab 12.5 MG |
| 54868372301 | Captopril | Captopril Tab 12.5 MG |
| 54868372302 | Captopril | Captopril Tab 12.5 MG |
| 54868372303 | Captopril | Captopril Tab 12.5 MG |
| 54868372304 | Captopril | Captopril Tab 12.5 MG |
| 54868372401 | Captopril | Captopril Tab 25 MG |
| 54868372402 | Captopril | Captopril Tab 25 MG |
| 54868372403 | Captopril | Captopril Tab 25 MG |
| 54868372404 | Captopril | Captopril Tab 25 MG |
| 54868372501 | Captopril | Captopril Tab 50 MG |
| 54868372502 | Captopril | Captopril Tab 50 MG |
| 49884079410 | Captopril | Captopril Tab 25 MG |
| 49884079474 | Captopril | Captopril Tab 25 MG |
| 49884079501 | Captopril | Captopril Tab 50 MG |
| 49884079510 | Captopril | Captopril Tab 50 MG |
| 49884079574 | Captopril | Captopril Tab 50 MG |
| 49884079601 | Captopril | Captopril Tab 100 MG |
| 49999010500 | Captopril | Captopril Tab 25 MG |
| 49999010530 | Captopril | Captopril Tab 25 MG |
| 49999010560 | Captopril | Captopril Tab 25 MG |
| 49999051160 | Captopril | Captopril Tab 50 MG |
| 51079086301 | Captopril | Captopril Tab 12.5 MG |
| 51079086320 | Captopril | Captopril Tab 12.5 MG |
| 51079086401 | Captopril | Captopril Tab 25 MG |
| 51079086420 | Captopril | Captopril Tab 25 MG |
| 51655097524 | Captopril | Captopril Tab 12.5 MG |
| 51655027825 | Captopril | Captopril Tab 50 MG |
| 51655027924 | Captopril | Captopril Tab 100 MG |
| 51655031125 | Captopril | Captopril Tab 25 MG |
| 00615451953 | Captopril | Captopril Tab 12.5 MG |
| 00615451963 | Captopril | Captopril Tab 12.5 MG |
| 00615452053 | Captopril | Captopril Tab 25 MG |
| 00615452063 | Captopril | Captopril Tab 25 MG |
| 00615452153 | Captopril | Captopril Tab 50 MG |
| 00615452163 | Captopril | Captopril Tab 50 MG |
| 00781182801 | Captopril | Captopril Tab 12.5 MG |
| 00781182810 | Captopril | Captopril Tab 12.5 MG |
| 00781182901 | Captopril | Captopril Tab 25 MG |
| 00781182910 | Captopril | Captopril Tab 25 MG |
| 00781183801 | Captopril | Captopril Tab 50 MG |
| 00781183810 | Captopril | Captopril Tab 50 MG |
| 00781183901 | Captopril | Captopril Tab 100 MG |
| 00904504560 | Captopril | Captopril Tab 12.5 MG |
| 00904504561 | Captopril | Captopril Tab 12.5 MG |
| 00904504580 | Captopril | Captopril Tab 12.5 MG |
| 00904504660 | Captopril | Captopril Tab 25 MG |
| 00904504661 | Captopril | Captopril Tab 25 MG |
| 00904504680 | Captopril | Captopril Tab 25 MG |
| 60429003060 | Captopril | Captopril Tab 25 MG |
| 60429003130 | Captopril | Captopril Tab 50 MG |
| 60346077830 | Captopril | Captopril Tab 25 MG |
| 60346077890 | Captopril | Captopril Tab 25 MG |
| 60346086830 | Captopril | Captopril Tab 50 MG |
| 54868519600 | Captopril | Captopril Tab 100 MG |
| 54868519601 | Captopril | Captopril Tab 100 MG |
| 55045237600 | Captopril | Captopril Tab 25 MG |
| 55045237608 | Captopril | Captopril Tab 25 MG |
| 55045237609 | Captopril | Captopril Tab 25 MG |
| 55045242400 | Captopril | Captopril Tab 50 MG |
| 55045242406 | Captopril | Captopril Tab 50 MG |
| 55289050630 | Captopril | Captopril Tab 25 MG |
| 55289050697 | Captopril | Captopril Tab 25 MG |
| 55289034430 | Captopril | Captopril Tab 25 MG |
| 55289034490 | Captopril | Captopril Tab 25 MG |
| 55289021230 | Captopril | Captopril Tab 50 MG |
| 55289021290 | Captopril | Captopril Tab 50 MG |
| 55370014207 | Captopril | Captopril Tab 25 MG |
| 55370014209 | Captopril | Captopril Tab 25 MG |
| 55370014407 | Captopril | Captopril Tab 50 MG |
| 55370014409 | Captopril | Captopril Tab 50 MG |
| 55370014507 | Captopril | Captopril Tab 100 MG |
| 55370016407 | Captopril | Captopril Tab 12.5 MG |
| 55370016409 | Captopril | Captopril Tab 12.5 MG |
| 55887058230 | Captopril | Captopril Tab 50 MG |
| 55887058260 | Captopril | Captopril Tab 50 MG |
| 55887058290 | Captopril | Captopril Tab 50 MG |
| 55953013240 | Captopril | Captopril Tab 12.5 MG |
| 55953013440 | Captopril | Captopril Tab 50 MG |
| 55953013540 | Captopril | Captopril Tab 100 MG |
| 57866610301 | Captopril | Captopril Tab 50 MG |
| 57866610302 | Captopril | Captopril Tab 50 MG |
| 57866610303 | Captopril | Captopril Tab 50 MG |
| 57866610304 | Captopril | Captopril Tab 50 MG |
| 57866610601 | Captopril | Captopril Tab 25 MG |
| 57866610602 | Captopril | Captopril Tab 25 MG |
| 57866610603 | Captopril | Captopril Tab 25 MG |
| 57866610604 | Captopril | Captopril Tab 25 MG |
| 54569052200 | Captopril | Captopril Tab 12.5 MG |
| 54569052201 | Captopril | Captopril Tab 12.5 MG |
| 54569052203 | Captopril | Captopril Tab 12.5 MG |
| 54569052300 | Captopril | Captopril Tab 25 MG |
| 54569052302 | Captopril | Captopril Tab 25 MG |
| 54569459300 | Captopril | Captopril Tab 12.5 MG |
| 54569459301 | Captopril | Captopril Tab 12.5 MG |
| 54569424600 | Captopril | Captopril Tab 25 MG |
| 54569424601 | Captopril | Captopril Tab 25 MG |
| 54569424603 | Captopril | Captopril Tab 25 MG |
| 54569424604 | Captopril | Captopril Tab 25 MG |
| 54569424605 | Captopril | Captopril Tab 25 MG |
| 54569424700 | Captopril | Captopril Tab 50 MG |
| 54569424702 | Captopril | Captopril Tab 50 MG |
| 68115042590 | Captopril | Captopril Tab 100 MG |
| 13411018402 | Captopril | Captopril Tab 50 MG |
| 13411018403 | Captopril | Captopril Tab 50 MG |
| 13411018406 | Captopril | Captopril Tab 50 MG |
| 13411018409 | Captopril | Captopril Tab 50 MG |
| 13411018410 | Captopril | Captopril Tab 50 MG |
| 23490519101 | Captopril | Captopril Tab 100 MG |
| 23490519201 | Captopril | Captopril Tab 12.5 MG |
| 23490519301 | Captopril | Captopril Tab 25 MG |
| 23490519302 | Captopril | Captopril Tab 25 MG |
| 23490519303 | Captopril | Captopril Tab 25 MG |
| 23490519400 | Captopril | Captopril Tab 50 MG |
| 23490519401 | Captopril | Captopril Tab 50 MG |
| 23490519402 | Captopril | Captopril Tab 50 MG |
| 63739004201 | Captopril | Captopril Tab 12.5 MG |
| 63739004202 | Captopril | Captopril Tab 12.5 MG |
| 63739004203 | Captopril | Captopril Tab 12.5 MG |
| 63739004215 | Captopril | Captopril Tab 12.5 MG |
| 63739004301 | Captopril | Captopril Tab 25 MG |
| 63739004302 | Captopril | Captopril Tab 25 MG |
| 63739004303 | Captopril | Captopril Tab 25 MG |
| 63739004315 | Captopril | Captopril Tab 25 MG |
| 63874034701 | Captopril | Captopril Tab 25 MG |
| 63874034702 | Captopril | Captopril Tab 25 MG |
| 63874034705 | Captopril | Captopril Tab 25 MG |
| 63874034720 | Captopril | Captopril Tab 25 MG |
| 63874034730 | Captopril | Captopril Tab 25 MG |
| 63874034740 | Captopril | Captopril Tab 25 MG |
| 63874034742 | Captopril | Captopril Tab 25 MG |
| 63874034745 | Captopril | Captopril Tab 25 MG |
| 63874034790 | Captopril | Captopril Tab 25 MG |
| 63874034801 | Captopril | Captopril Tab 50 MG |
| 63874034810 | Captopril | Captopril Tab 50 MG |
| 63874034814 | Captopril | Captopril Tab 50 MG |
| 63874034820 | Captopril | Captopril Tab 50 MG |
| 63874034830 | Captopril | Captopril Tab 50 MG |
| 63874034860 | Captopril | Captopril Tab 50 MG |
| 63874034890 | Captopril | Captopril Tab 50 MG |
| 63874034901 | Captopril | Captopril Tab 100 MG |
| 63874034910 | Captopril | Captopril Tab 100 MG |
| 63874034930 | Captopril | Captopril Tab 100 MG |
| 57866024901 | Captopril | Captopril Tab 25 MG |
| 58864006628 | Captopril | Captopril Tab 25 MG |
| 59930165501 | Captopril | Captopril Tab 12.5 MG |
| 59930165502 | Captopril | Captopril Tab 12.5 MG |
| 59930165503 | Captopril | Captopril Tab 12.5 MG |
| 59930165601 | Captopril | Captopril Tab 25 MG |
| 59930165602 | Captopril | Captopril Tab 25 MG |
| 59930165603 | Captopril | Captopril Tab 25 MG |
| 59930165701 | Captopril | Captopril Tab 50 MG |
| 59930165702 | Captopril | Captopril Tab 50 MG |
| 59930165703 | Captopril | Captopril Tab 50 MG |
| 59930165801 | Captopril | Captopril Tab 100 MG |
| 59772704501 | Captopril | Captopril Tab 12.5 MG |
| 59772704503 | Captopril | Captopril Tab 12.5 MG |
| 59772704601 | Captopril | Captopril Tab 25 MG |
| 59772704603 | Captopril | Captopril Tab 25 MG |
| 59772704701 | Captopril | Captopril Tab 50 MG |
| 59772704703 | Captopril | Captopril Tab 50 MG |
| 59772704801 | Captopril | Captopril Tab 100 MG |
| 66336094660 | Captopril | Captopril Tab 25 MG |
| 66336094690 | Captopril | Captopril Tab 25 MG |
| 67544015045 | Captopril | Captopril Tab 12.5 MG |
| 67544015060 | Captopril | Captopril Tab 12.5 MG |
| 67544015073 | Captopril | Captopril Tab 12.5 MG |
| 67544015092 | Captopril | Captopril Tab 12.5 MG |
| 68115006090 | Captopril | Captopril Tab 50 MG |
| 00003045006 | Captopril | Captopril Tab 12.5 MG |
| 00003045239 | Captopril | Captopril Tab 25 MG |
| 00003048206 | Captopril | Captopril Tab 50 MG |
| 00003048551 | Captopril | Captopril Tab 100 MG |
| 00047052224 | Captopril | Captopril Tab 25 MG |
| 00047052232 | Captopril | Captopril Tab 25 MG |
| 00047054224 | Captopril | Captopril Tab 50 MG |
| 00047054232 | Captopril | Captopril Tab 50 MG |
| 00047054324 | Captopril | Captopril Tab 100 MG |
| 00047055924 | Captopril | Captopril Tab 12.5 MG |
| 00047055932 | Captopril | Captopril Tab 12.5 MG |
| 49999010510 | Captopril | Captopril Tab 25 MG |
| 49999051130 | Captopril | Captopril Tab 50 MG |
| 54868519602 | Captopril | Captopril Tab 100 MG |
| 54868372503 | Captopril | Captopril Tab 50 MG |
| 60429002990 | Captopril | Captopril Tab 12.5 MG |
| 60429003012 | Captopril | Captopril Tab 25 MG |
| 60429003027 | Captopril | Captopril Tab 25 MG |
| 60429003090 | Captopril | Captopril Tab 25 MG |
| 60429003112 | Captopril | Captopril Tab 50 MG |
| 60429003127 | Captopril | Captopril Tab 50 MG |
| 60429003160 | Captopril | Captopril Tab 50 MG |
| 60429003190 | Captopril | Captopril Tab 50 MG |
| 60505000306 | Captopril | Captopril Tab 12.5 MG |
| 60505000309 | Captopril | Captopril Tab 12.5 MG |
| 60505000406 | Captopril | Captopril Tab 25 MG |
| 60505000409 | Captopril | Captopril Tab 25 MG |
| 60505000506 | Captopril | Captopril Tab 50 MG |
| 60505000509 | Captopril | Captopril Tab 50 MG |
| 60505000606 | Captopril | Captopril Tab 100 MG |
| 60505000609 | Captopril | Captopril Tab 100 MG |
| 63739004210 | Captopril | Captopril Tab 12.5 MG |
| 63739004310 | Captopril | Captopril Tab 25 MG |
| 64679090201 | Captopril | Captopril Tab 12.5 MG |
| 64679090202 | Captopril | Captopril Tab 12.5 MG |
| 64679090301 | Captopril | Captopril Tab 25 MG |
| 64679090302 | Captopril | Captopril Tab 25 MG |
| 64679090401 | Captopril | Captopril Tab 50 MG |
| 64679090402 | Captopril | Captopril Tab 50 MG |
| 64679090501 | Captopril | Captopril Tab 100 MG |
| 68115005900 | Captopril | Captopril Tab 25 MG |
| 68115005930 | Captopril | Captopril Tab 25 MG |
| 68115005960 | Captopril | Captopril Tab 25 MG |
| 68115005990 | Captopril | Captopril Tab 25 MG |
| 68115006030 | Captopril | Captopril Tab 50 MG |
| 68115006060 | Captopril | Captopril Tab 50 MG |
| 00615452165 | Captopril | Captopril Tab 50 MG |
| 00677159201 | Captopril | Captopril Tab 12.5 MG |
| 00677159210 | Captopril | Captopril Tab 12.5 MG |
| 00677159301 | Captopril | Captopril Tab 25 MG |
| 00677159310 | Captopril | Captopril Tab 25 MG |
| 00677159401 | Captopril | Captopril Tab 50 MG |
| 00677159410 | Captopril | Captopril Tab 50 MG |
| 00677159501 | Captopril | Captopril Tab 100 MG |
| 00536427201 | Captopril | Captopril Tab 12.5 MG |
| 00536427205 | Captopril | Captopril Tab 12.5 MG |
| 00536427301 | Captopril | Captopril Tab 25 MG |
| 00536427310 | Captopril | Captopril Tab 25 MG |
| 00536427401 | Captopril | Captopril Tab 50 MG |
| 00536427405 | Captopril | Captopril Tab 50 MG |
| 00536427501 | Captopril | Captopril Tab 100 MG |
| 00603255521 | Captopril | Captopril Tab 12.5 MG |
| 00603255532 | Captopril | Captopril Tab 12.5 MG |
| 00603255621 | Captopril | Captopril Tab 25 MG |
| 00603255632 | Captopril | Captopril Tab 25 MG |
| 00603255721 | Captopril | Captopril Tab 50 MG |
| 00603255732 | Captopril | Captopril Tab 50 MG |
| 00603255821 | Captopril | Captopril Tab 100 MG |
| 00364262801 | Captopril | Captopril Tab 12.5 MG |
| 00364262802 | Captopril | Captopril Tab 12.5 MG |
| 00364262805 | Captopril | Captopril Tab 12.5 MG |
| 00364262901 | Captopril | Captopril Tab 25 MG |
| 00364262902 | Captopril | Captopril Tab 25 MG |
| 00364262905 | Captopril | Captopril Tab 25 MG |
| 00364263001 | Captopril | Captopril Tab 50 MG |
| 00364263002 | Captopril | Captopril Tab 50 MG |
| 00364263005 | Captopril | Captopril Tab 50 MG |
| 00364263101 | Captopril | Captopril Tab 100 MG |
| 00403052730 | Captopril | Captopril Tab 25 MG |
| 00536347101 | Captopril | Captopril Tab 12.5 MG |
| 00536347105 | Captopril | Captopril Tab 12.5 MG |
| 00536347201 | Captopril | Captopril Tab 25 MG |
| 00536347210 | Captopril | Captopril Tab 25 MG |
| 00536347301 | Captopril | Captopril Tab 50 MG |
| 00536347305 | Captopril | Captopril Tab 50 MG |
| 00536347401 | Captopril | Captopril Tab 100 MG |
| 00185003101 | Captopril | Captopril Tab 12.5 MG |
| 00185003110 | Captopril | Captopril Tab 12.5 MG |
| 00185006101 | Captopril | Captopril Tab 25 MG |
| 00185006110 | Captopril | Captopril Tab 25 MG |
| 00185047101 | Captopril | Captopril Tab 50 MG |
| 00185047110 | Captopril | Captopril Tab 50 MG |
| 00185059101 | Captopril | Captopril Tab 100 MG |
| 00185059105 | Captopril | Captopril Tab 100 MG |
| 00185059110 | Captopril | Captopril Tab 100 MG |
| 00182262201 | Captopril | Captopril Tab 12.5 MG |
| 00182262205 | Captopril | Captopril Tab 12.5 MG |
| 00182262210 | Captopril | Captopril Tab 12.5 MG |
| 00182262301 | Captopril | Captopril Tab 25 MG |
| 00182262305 | Captopril | Captopril Tab 25 MG |
| 00182262310 | Captopril | Captopril Tab 25 MG |
| 00182262401 | Captopril | Captopril Tab 50 MG |
| 00182262405 | Captopril | Captopril Tab 50 MG |
| 00182262410 | Captopril | Captopril Tab 50 MG |
| 00182262501 | Captopril | Captopril Tab 100 MG |
| 66336061860 | Captopril | Captopril Tab 12.5 MG |
| 66336075060 | Captopril | Captopril Tab 50 MG |
| 66336075090 | Captopril | Captopril Tab 50 MG |
| 66336079460 | Captopril | Captopril Tab 100 MG |
| 66336094630 | Captopril | Captopril Tab 25 MG |
| 52544069010 | Captopril | Captopril Tab 50 MG |
| 52544069101 | Captopril | Captopril Tab 100 MG |
| 52493062701 | Captopril | Captopril Tab 25 MG |
| 52493062760 | Captopril | Captopril Tab 25 MG |
| 52555063701 | Captopril | Captopril Tab 12.5 MG |
| 52555063710 | Captopril | Captopril Tab 12.5 MG |
| 52555063801 | Captopril | Captopril Tab 25 MG |
| 52555063810 | Captopril | Captopril Tab 25 MG |
| 52555063901 | Captopril | Captopril Tab 50 MG |
| 52555063910 | Captopril | Captopril Tab 50 MG |
| 52555064001 | Captopril | Captopril Tab 100 MG |
| 49884044401 | Captopril | Captopril Tab 12.5 MG |
| 49884044410 | Captopril | Captopril Tab 12.5 MG |
| 49884044501 | Captopril | Captopril Tab 25 MG |
| 49884044510 | Captopril | Captopril Tab 25 MG |
| 49884044601 | Captopril | Captopril Tab 50 MG |
| 49884044610 | Captopril | Captopril Tab 50 MG |
| 49884044701 | Captopril | Captopril Tab 100 MG |
| 51285095002 | Captopril | Captopril Tab 12.5 MG |
| 51285095005 | Captopril | Captopril Tab 12.5 MG |
| 51285095102 | Captopril | Captopril Tab 25 MG |
| 51285095105 | Captopril | Captopril Tab 25 MG |
| 51285095202 | Captopril | Captopril Tab 50 MG |
| 51285095205 | Captopril | Captopril Tab 50 MG |
| 51285095302 | Captopril | Captopril Tab 100 MG |
| 51285095502 | Captopril | Captopril Tab 12.5 MG |
| 51285095505 | Captopril | Captopril Tab 12.5 MG |
| 51285095602 | Captopril | Captopril Tab 25 MG |
| 51285095605 | Captopril | Captopril Tab 25 MG |
| 51285095702 | Captopril | Captopril Tab 50 MG |
| 51285095705 | Captopril | Captopril Tab 50 MG |
| 51285095802 | Captopril | Captopril Tab 100 MG |
| 51079086317 | Captopril | Captopril Tab 12.5 MG |
| 51079086319 | Captopril | Captopril Tab 12.5 MG |
| 51079086417 | Captopril | Captopril Tab 25 MG |
| 51079086419 | Captopril | Captopril Tab 25 MG |
| 11845112001 | Captopril | Captopril Tab 12.5 MG |
| 11845112004 | Captopril | Captopril Tab 12.5 MG |
| 11845112101 | Captopril | Captopril Tab 25 MG |
| 11845112104 | Captopril | Captopril Tab 25 MG |
| 11845112201 | Captopril | Captopril Tab 50 MG |
| 11845112204 | Captopril | Captopril Tab 50 MG |
| 11845112301 | Captopril | Captopril Tab 100 MG |
| 40893062701 | Captopril | Captopril Tab 25 MG |
| 40893062760 | Captopril | Captopril Tab 25 MG |
| 00839799406 | Captopril | Captopril Tab 12.5 MG |
| 00839799412 | Captopril | Captopril Tab 12.5 MG |
| 00839799416 | Captopril | Captopril Tab 12.5 MG |
| 00839799506 | Captopril | Captopril Tab 25 MG |
| 00839799512 | Captopril | Captopril Tab 25 MG |
| 00839799516 | Captopril | Captopril Tab 25 MG |
| 00839799606 | Captopril | Captopril Tab 50 MG |
| 00839799612 | Captopril | Captopril Tab 50 MG |
| 00839799616 | Captopril | Captopril Tab 50 MG |
| 00839806406 | Captopril | Captopril Tab 100 MG |
| 00839806416 | Captopril | Captopril Tab 100 MG |
| 00615451929 | Captopril | Captopril Tab 12.5 MG |
| 00615451943 | Captopril | Captopril Tab 12.5 MG |
| 00615451965 | Captopril | Captopril Tab 12.5 MG |
| 00615452029 | Captopril | Captopril Tab 25 MG |
| 00615452043 | Captopril | Captopril Tab 25 MG |
| 00615452065 | Captopril | Captopril Tab 25 MG |
| 00615452129 | Captopril | Captopril Tab 50 MG |
| 00615452143 | Captopril | Captopril Tab 50 MG |
| 54977005430 | Captopril | Captopril Tab 50 MG |
| 54977005499 | Captopril | Captopril Tab 50 MG |
| 54977005660 | Captopril | Captopril Tab 12.5 MG |
| 54977005699 | Captopril | Captopril Tab 12.5 MG |
| 54977005730 | Captopril | Captopril Tab 25 MG |
| 54977005799 | Captopril | Captopril Tab 25 MG |
| 55289050601 | Captopril | Captopril Tab 25 MG |
| 55175516501 | Captopril | Captopril Tab 50 MG |
| 55947040730 | Captopril | Captopril Tab 25 MG |
| 55953013270 | Captopril | Captopril Tab 12.5 MG |
| 55953013280 | Captopril | Captopril Tab 12.5 MG |
| 55953013340 | Captopril | Captopril Tab 25 MG |
| 55953013370 | Captopril | Captopril Tab 25 MG |
| 55953013380 | Captopril | Captopril Tab 25 MG |
| 55953013470 | Captopril | Captopril Tab 50 MG |
| 55953013480 | Captopril | Captopril Tab 50 MG |
| 55953013570 | Captopril | Captopril Tab 100 MG |
| 55953013580 | Captopril | Captopril Tab 100 MG |
| 57362029011 | Captopril | Captopril Tab 25 MG |
| 57362029013 | Captopril | Captopril Tab 25 MG |
| 57362029019 | Captopril | Captopril Tab 25 MG |
| 57362029073 | Captopril | Captopril Tab 25 MG |
| 57362029083 | Captopril | Captopril Tab 25 MG |
| 53506005930 | Captopril | Captopril Tab 25 MG |
| 53506072130 | Captopril | Captopril Tab 25 MG |
| 54124025802 | Captopril | Captopril Tab 25 MG |
| 54124025812 | Captopril | Captopril Tab 25 MG |
| 54124025815 | Captopril | Captopril Tab 25 MG |
| 54124025830 | Captopril | Captopril Tab 25 MG |
| 54124025860 | Captopril | Captopril Tab 25 MG |
| 54124025890 | Captopril | Captopril Tab 25 MG |
| 54124049130 | Captopril | Captopril Tab 50 MG |
| 54124049160 | Captopril | Captopril Tab 50 MG |
| 54124049190 | Captopril | Captopril Tab 50 MG |
| 54569052202 | Captopril | Captopril Tab 12.5 MG |
| 54569052301 | Captopril | Captopril Tab 25 MG |
| 54569052400 | Captopril | Captopril Tab 50 MG |
| 54569052401 | Captopril | Captopril Tab 50 MG |
| 54569052500 | Captopril | Captopril Tab 100 MG |
| 51875034801 | Captopril | Captopril Tab 25 MG |
| 51875034802 | Captopril | Captopril Tab 25 MG |
| 51875034804 | Captopril | Captopril Tab 25 MG |
| 51875034901 | Captopril | Captopril Tab 50 MG |
| 51875034902 | Captopril | Captopril Tab 50 MG |
| 51875034904 | Captopril | Captopril Tab 50 MG |
| 51875035001 | Captopril | Captopril Tab 100 MG |
| 51875035501 | Captopril | Captopril Tab 12.5 MG |
| 51875035502 | Captopril | Captopril Tab 12.5 MG |
| 51875035504 | Captopril | Captopril Tab 12.5 MG |
| 52246060490 | Captopril | Captopril Tab 25 MG |
| 52544068801 | Captopril | Captopril Tab 12.5 MG |
| 52544068805 | Captopril | Captopril Tab 12.5 MG |
| 52544068810 | Captopril | Captopril Tab 12.5 MG |
| 52544068901 | Captopril | Captopril Tab 25 MG |
| 52544068905 | Captopril | Captopril Tab 25 MG |
| 52544068910 | Captopril | Captopril Tab 25 MG |
| 52544069001 | Captopril | Captopril Tab 50 MG |
| 52544069005 | Captopril | Captopril Tab 50 MG |
| 59772704606 | Captopril | Captopril Tab 25 MG |
| 59772704704 | Captopril | Captopril Tab 50 MG |
| 59772704705 | Captopril | Captopril Tab 50 MG |
| 60951072170 | Captopril | Captopril Tab 12.5 MG |
| 60951072190 | Captopril | Captopril Tab 12.5 MG |
| 60951072270 | Captopril | Captopril Tab 25 MG |
| 60951072290 | Captopril | Captopril Tab 25 MG |
| 60951072470 | Captopril | Captopril Tab 50 MG |
| 60951072490 | Captopril | Captopril Tab 50 MG |
| 60951072770 | Captopril | Captopril Tab 100 MG |
| 60951072790 | Captopril | Captopril Tab 100 MG |
| 62584063601 | Captopril | Captopril Tab 12.5 MG |
| 62584063633 | Captopril | Captopril Tab 12.5 MG |
| 62584063701 | Captopril | Captopril Tab 25 MG |
| 57362029084 | Captopril | Captopril Tab 25 MG |
| 57362029085 | Captopril | Captopril Tab 25 MG |
| 57362030211 | Captopril | Captopril Tab 50 MG |
| 57362030213 | Captopril | Captopril Tab 50 MG |
| 57362030219 | Captopril | Captopril Tab 50 MG |
| 57362030273 | Captopril | Captopril Tab 50 MG |
| 57362030283 | Captopril | Captopril Tab 50 MG |
| 57362030284 | Captopril | Captopril Tab 50 MG |
| 57362030285 | Captopril | Captopril Tab 50 MG |
| 57480083801 | Captopril | Captopril Tab 12.5 MG |
| 57480083806 | Captopril | Captopril Tab 12.5 MG |
| 57480083901 | Captopril | Captopril Tab 25 MG |
| 57480083906 | Captopril | Captopril Tab 25 MG |
| 57480084001 | Captopril | Captopril Tab 50 MG |
| 57480084006 | Captopril | Captopril Tab 50 MG |
| 57480084101 | Captopril | Captopril Tab 100 MG |
| 57480084106 | Captopril | Captopril Tab 100 MG |
| 57362051311 | Captopril | Captopril Tab 12.5 MG |
| 57362051319 | Captopril | Captopril Tab 12.5 MG |
| 57362051373 | Captopril | Captopril Tab 12.5 MG |
| 57362051384 | Captopril | Captopril Tab 12.5 MG |
| 59911583201 | Captopril | Captopril Tab 12.5 MG |
| 59911583202 | Captopril | Captopril Tab 12.5 MG |
| 59911583301 | Captopril | Captopril Tab 25 MG |
| 59911583302 | Captopril | Captopril Tab 25 MG |
| 59911583401 | Captopril | Captopril Tab 50 MG |
| 59911583402 | Captopril | Captopril Tab 50 MG |
| 59911583501 | Captopril | Captopril Tab 100 MG |
| 59930165802 | Captopril | Captopril Tab 100 MG |
| 59930165803 | Captopril | Captopril Tab 100 MG |
| 59772704504 | Captopril | Captopril Tab 12.5 MG |
| 59772704505 | Captopril | Captopril Tab 12.5 MG |
| 59772704506 | Captopril | Captopril Tab 12.5 MG |
| 59772704507 | Captopril | Captopril Tab 12.5 MG |
| 59772704602 | Captopril | Captopril Tab 25 MG |
| 59772704604 | Captopril | Captopril Tab 25 MG |
| 54569291600 | Captopril | Captopril Tab 50 MG |
| 54569700400 | Captopril | Captopril Tab 25 MG |
| 54569424602 | Captopril | Captopril Tab 25 MG |
| 54569424701 | Captopril | Captopril Tab 50 MG |
| 54569853700 | Captopril | Captopril Tab 25 MG |
| 54569853701 | Captopril | Captopril Tab 25 MG |
| 54569854900 | Captopril | Captopril Tab 50 MG |
| 54868177502 | Captopril | Captopril Tab 12.5 MG |
| 21695047778 | Captopril | Captopril Tab 25 MG |
| 60429002901 | Captopril | Captopril Tab 12.5 MG |
| 60429002910 | Captopril | Captopril Tab 12.5 MG |
| 60429003001 | Captopril | Captopril Tab 25 MG |
| 60429003010 | Captopril | Captopril Tab 25 MG |
| 60429003101 | Captopril | Captopril Tab 50 MG |
| 60429003110 | Captopril | Captopril Tab 50 MG |
| 60429025701 | Captopril | Captopril Tab 100 MG |
| 54569424607 | Captopril | Captopril Tab 25 MG |
| 54569424704 | Captopril | Captopril Tab 50 MG |
| 54569459303 | Captopril | Captopril Tab 12.5 MG |
| 54868372305 | Captopril | Captopril Tab 12.5 MG |
| 51138001330 | Captopril | Captopril Tab 12.5 MG |
| 51138001430 | Captopril | Captopril Tab 25 MG |
| 51138001530 | Captopril | Captopril Tab 50 MG |
| 51138001630 | Captopril | Captopril Tab 100 MG |
| 51138050515 | Captopril | Captopril Tab 12.5 MG |
| 51138050530 | Captopril | Captopril Tab 12.5 MG |
| 51138050630 | Captopril | Captopril Tab 25 MG |
| 51138050730 | Captopril | Captopril Tab 50 MG |
| 51138050820 | Captopril | Captopril Tab 100 MG |
| 51138050845 | Captopril | Captopril Tab 100 MG |
| 68645016359 | Captopril | Captopril Tab 100 MG |
| 53002043100 | Captopril | Captopril Tab 25 MG |
| 53002043130 | Captopril | Captopril Tab 25 MG |
| 53002043160 | Captopril | Captopril Tab 25 MG |
| 53002108600 | Captopril | Captopril Tab 50 MG |
| 53002108603 | Captopril | Captopril Tab 50 MG |
| 53002108606 | Captopril | Captopril Tab 50 MG |
| 66336075030 | Captopril | Captopril Tab 50 MG |
| 63629254103 | Captopril | Captopril Tab 50 MG |
| 63629254104 | Captopril | Captopril Tab 50 MG |
| 62584063733 | Captopril | Captopril Tab 25 MG |
| 54868372504 | Captopril | Captopril Tab 50 MG |
| 63629133801 | Captopril | Captopril Tab 25 MG |
| 63629133802 | Captopril | Captopril Tab 25 MG |
| 63629133803 | Captopril | Captopril Tab 25 MG |
| 63629170601 | Captopril | Captopril Tab 100 MG |
| 63629170602 | Captopril | Captopril Tab 100 MG |
| 63629170603 | Captopril | Captopril Tab 100 MG |
| 63629254101 | Captopril | Captopril Tab 50 MG |
| 63629254102 | Captopril | Captopril Tab 50 MG |
| 63629289601 | Captopril | Captopril Tab 12.5 MG |
| 21695047730 | Captopril | Captopril Tab 25 MG |
| 21695047830 | Captopril | Captopril Tab 50 MG |
| 68645016059 | Captopril | Captopril Tab 12.5 MG |
| 68645016159 | Captopril | Captopril Tab 25 MG |
| 68645016259 | Captopril | Captopril Tab 50 MG |
| 43063014630 | Captopril | Captopril Tab 12.5 MG |
| 59772704605 | Captopril | Captopril Tab 25 MG |
| 00093017601 | Captopril & Hydrochlorothiazide | Captopril & Hydrochlorothiazide Tab 25-15 MG |
| 54569054501 | Captopril & Hydrochlorothiazide | Captopril & Hydrochlorothiazide Tab 50-25 MG |
| 00093018101 | Captopril & Hydrochlorothiazide | Captopril & Hydrochlorothiazide Tab 50-15 MG |
| 00093018201 | Captopril & Hydrochlorothiazide | Captopril & Hydrochlorothiazide Tab 50-25 MG |
| 00172501560 | Captopril & Hydrochlorothiazide | Captopril & Hydrochlorothiazide Tab 50-15 MG |
| 00172502560 | Captopril & Hydrochlorothiazide | Captopril & Hydrochlorothiazide Tab 50-25 MG |
| 00172251560 | Captopril & Hydrochlorothiazide | Captopril & Hydrochlorothiazide Tab 25-15 MG |
| 00172252560 | Captopril & Hydrochlorothiazide | Captopril & Hydrochlorothiazide Tab 25-25 MG |
| 00378008101 | Captopril & Hydrochlorothiazide | Captopril & Hydrochlorothiazide Tab 25-15 MG |
| 00378008301 | Captopril & Hydrochlorothiazide | Captopril & Hydrochlorothiazide Tab 25-25 MG |
| 00378008401 | Captopril & Hydrochlorothiazide | Captopril & Hydrochlorothiazide Tab 50-15 MG |
| 00378008601 | Captopril & Hydrochlorothiazide | Captopril & Hydrochlorothiazide Tab 50-25 MG |
| 00003033850 | Captopril & Hydrochlorothiazide | Captopril & Hydrochlorothiazide Tab 25-15 MG |
| 00003034950 | Captopril & Hydrochlorothiazide | Captopril & Hydrochlorothiazide Tab 25-25 MG |
| 00003038450 | Captopril & Hydrochlorothiazide | Captopril & Hydrochlorothiazide Tab 50-15 MG |
| 00003039050 | Captopril & Hydrochlorothiazide | Captopril & Hydrochlorothiazide Tab 50-25 MG |
| 54868389100 | Captopril & Hydrochlorothiazide | Captopril & Hydrochlorothiazide Tab 50-25 MG |
| 54868376900 | Captopril & Hydrochlorothiazide | Captopril & Hydrochlorothiazide Tab 25-15 MG |
| 49884081501 | Captopril & Hydrochlorothiazide | Captopril & Hydrochlorothiazide Tab 25-15 MG |
| 49884081601 | Captopril & Hydrochlorothiazide | Captopril & Hydrochlorothiazide Tab 25-25 MG |
| 49884081701 | Captopril & Hydrochlorothiazide | Captopril & Hydrochlorothiazide Tab 50-15 MG |
| 49884081801 | Captopril & Hydrochlorothiazide | Captopril & Hydrochlorothiazide Tab 50-25 MG |
| 60951073170 | Captopril & Hydrochlorothiazide | Captopril & Hydrochlorothiazide Tab 50-25 MG |
| 60951073370 | Captopril & Hydrochlorothiazide | Captopril & Hydrochlorothiazide Tab 25-15 MG |
| 60951073970 | Captopril & Hydrochlorothiazide | Captopril & Hydrochlorothiazide Tab 50-15 MG |
| 60951074170 | Captopril & Hydrochlorothiazide | Captopril & Hydrochlorothiazide Tab 25-25 MG |
| 54868406200 | Captopril & Hydrochlorothiazide | Captopril & Hydrochlorothiazide Tab 50-25 MG |
| 59772516005 | Captopril & Hydrochlorothiazide | Captopril & Hydrochlorothiazide Tab 25-15 MG |
| 59772516105 | Captopril & Hydrochlorothiazide | Captopril & Hydrochlorothiazide Tab 25-25 MG |
| 59772516205 | Captopril & Hydrochlorothiazide | Captopril & Hydrochlorothiazide Tab 50-15 MG |
| 59772516305 | Captopril & Hydrochlorothiazide | Captopril & Hydrochlorothiazide Tab 50-25 MG |
| 54868578700 | Captopril & Hydrochlorothiazide | Captopril & Hydrochlorothiazide Tab 25-25 MG |
| 54868406201 | Captopril & Hydrochlorothiazide | Captopril & Hydrochlorothiazide Tab 50-25 MG |
| 54124038830 | Captopril & Hydrochlorothiazide | Captopril & Hydrochlorothiazide Tab 25-15 MG |
| 54569052100 | Captopril & Hydrochlorothiazide | Captopril & Hydrochlorothiazide Tab 25-15 MG |
| 54569052101 | Captopril & Hydrochlorothiazide | Captopril & Hydrochlorothiazide Tab 25-15 MG |
| 54569052102 | Captopril & Hydrochlorothiazide | Captopril & Hydrochlorothiazide Tab 25-15 MG |
| 54569054500 | Captopril & Hydrochlorothiazide | Captopril & Hydrochlorothiazide Tab 50-25 MG |
| 00093017701 | Captopril & Hydrochlorothiazide | Captopril & Hydrochlorothiazide Tab 25-25 MG |
| 00378007201 | Clonidine & Chlorthalidone | Clonidine & Chlorthalidone Tab 0.3-15 MG |
| 00405424801 | Clonidine & Chlorthalidone | Clonidine & Chlorthalidone Tab 0.1-15 MG |
| 00405424901 | Clonidine & Chlorthalidone | Clonidine & Chlorthalidone Tab 0.2-15 MG |
| 00405425001 | Clonidine & Chlorthalidone | Clonidine & Chlorthalidone Tab 0.3-15 MG |
| 00304176301 | Clonidine & Chlorthalidone | Clonidine & Chlorthalidone Tab 0.1-15 MG |
| 00304176401 | Clonidine & Chlorthalidone | Clonidine & Chlorthalidone Tab 0.2-15 MG |
| 00304176501 | Clonidine & Chlorthalidone | Clonidine & Chlorthalidone Tab 0.3-15 MG |
| 00304576301 | Clonidine & Chlorthalidone | Clonidine & Chlorthalidone Tab 0.1-15 MG |
| 00304576401 | Clonidine & Chlorthalidone | Clonidine & Chlorthalidone Tab 0.2-15 MG |
| 00304576501 | Clonidine & Chlorthalidone | Clonidine & Chlorthalidone Tab 0.3-15 MG |
| 00349866001 | Clonidine & Chlorthalidone | Clonidine & Chlorthalidone Tab 0.1-15 MG |
| 00093050301 | Clonidine & Chlorthalidone | Clonidine & Chlorthalidone Tab 0.2-15 MG |
| 00093050401 | Clonidine & Chlorthalidone | Clonidine & Chlorthalidone Tab 0.3-15 MG |
| 00102374001 | Clonidine & Chlorthalidone | Clonidine & Chlorthalidone Tab 0.1-15 MG |
| 00102374501 | Clonidine & Chlorthalidone | Clonidine & Chlorthalidone Tab 0.2-15 MG |
| 00182127501 | Clonidine & Chlorthalidone | Clonidine & Chlorthalidone Tab 0.1-15 MG |
| 00182127601 | Clonidine & Chlorthalidone | Clonidine & Chlorthalidone Tab 0.2-15 MG |
| 00182127701 | Clonidine & Chlorthalidone | Clonidine & Chlorthalidone Tab 0.3-15 MG |
| 00047098524 | Clonidine & Chlorthalidone | Clonidine & Chlorthalidone Tab 0.1-15 MG |
| 00047098624 | Clonidine & Chlorthalidone | Clonidine & Chlorthalidone Tab 0.2-15 MG |
| 00047098724 | Clonidine & Chlorthalidone | Clonidine & Chlorthalidone Tab 0.3-15 MG |
| 00093050201 | Clonidine & Chlorthalidone | Clonidine & Chlorthalidone Tab 0.1-15 MG |
| 62794000101 | Clonidine & Chlorthalidone | Clonidine & Chlorthalidone Tab 0.1-15 MG |
| 62794002701 | Clonidine & Chlorthalidone | Clonidine & Chlorthalidone Tab 0.2-15 MG |
| 62794007201 | Clonidine & Chlorthalidone | Clonidine & Chlorthalidone Tab 0.3-15 MG |
| 54868526700 | Clonidine & Chlorthalidone | Clonidine & Chlorthalidone Tab 0.2-15 MG |
| 54868526701 | Clonidine & Chlorthalidone | Clonidine & Chlorthalidone Tab 0.2-15 MG |
| 54868188800 | Clonidine & Chlorthalidone | Clonidine & Chlorthalidone Tab 0.1-15 MG |
| 54868188801 | Clonidine & Chlorthalidone | Clonidine & Chlorthalidone Tab 0.1-15 MG |
| 00597000801 | Clonidine & Chlorthalidone | Clonidine & Chlorthalidone Tab 0.1-15 MG |
| 00597000901 | Clonidine & Chlorthalidone | Clonidine & Chlorthalidone Tab 0.2-15 MG |
| 54569189300 | Clonidine & Chlorthalidone | Clonidine & Chlorthalidone Tab 0.1-15 MG |
| 54274043310 | Clonidine & Chlorthalidone | Clonidine & Chlorthalidone Tab 0.1-15 MG |
| 54274043410 | Clonidine & Chlorthalidone | Clonidine & Chlorthalidone Tab 0.2-15 MG |
| 54274043510 | Clonidine & Chlorthalidone | Clonidine & Chlorthalidone Tab 0.3-15 MG |
| 51432010103 | Clonidine & Chlorthalidone | Clonidine & Chlorthalidone Tab 0.3-15 MG |
| 51432010130 | Clonidine & Chlorthalidone | Clonidine & Chlorthalidone Tab 0.3-15 MG |
| 52446008521 | Clonidine & Chlorthalidone | Clonidine & Chlorthalidone Tab 0.1-15 MG |
| 52446008621 | Clonidine & Chlorthalidone | Clonidine & Chlorthalidone Tab 0.2-15 MG |
| 49884011301 | Clonidine & Chlorthalidone | Clonidine & Chlorthalidone Tab 0.1-15 MG |
| 49884011305 | Clonidine & Chlorthalidone | Clonidine & Chlorthalidone Tab 0.1-15 MG |
| 49884011310 | Clonidine & Chlorthalidone | Clonidine & Chlorthalidone Tab 0.1-15 MG |
| 49884011501 | Clonidine & Chlorthalidone | Clonidine & Chlorthalidone Tab 0.2-15 MG |
| 49884011505 | Clonidine & Chlorthalidone | Clonidine & Chlorthalidone Tab 0.2-15 MG |
| 49884011510 | Clonidine & Chlorthalidone | Clonidine & Chlorthalidone Tab 0.2-15 MG |
| 49884011601 | Clonidine & Chlorthalidone | Clonidine & Chlorthalidone Tab 0.3-15 MG |
| 51432009503 | Clonidine & Chlorthalidone | Clonidine & Chlorthalidone Tab 0.1-15 MG |
| 51432009703 | Clonidine & Chlorthalidone | Clonidine & Chlorthalidone Tab 0.2-15 MG |
| 17236067401 | Clonidine & Chlorthalidone | Clonidine & Chlorthalidone Tab 0.1-15 MG |
| 17236067501 | Clonidine & Chlorthalidone | Clonidine & Chlorthalidone Tab 0.2-15 MG |
| 47202278601 | Clonidine & Chlorthalidone | Clonidine & Chlorthalidone Tab 0.1-15 MG |
| 47202278701 | Clonidine & Chlorthalidone | Clonidine & Chlorthalidone Tab 0.2-15 MG |
| 47202278801 | Clonidine & Chlorthalidone | Clonidine & Chlorthalidone Tab 0.3-15 MG |
| 47202293901 | Clonidine & Chlorthalidone | Clonidine & Chlorthalidone Tab 0.1-15 MG |
| 47202294001 | Clonidine & Chlorthalidone | Clonidine & Chlorthalidone Tab 0.2-15 MG |
| 47202294101 | Clonidine & Chlorthalidone | Clonidine & Chlorthalidone Tab 0.3-15 MG |
| 00814173414 | Clonidine & Chlorthalidone | Clonidine & Chlorthalidone Tab 0.1-15 MG |
| 00839728506 | Clonidine & Chlorthalidone | Clonidine & Chlorthalidone Tab 0.1-15 MG |
| 00839728606 | Clonidine & Chlorthalidone | Clonidine & Chlorthalidone Tab 0.2-15 MG |
| 00839728706 | Clonidine & Chlorthalidone | Clonidine & Chlorthalidone Tab 0.3-15 MG |
| 00839794306 | Clonidine & Chlorthalidone | Clonidine & Chlorthalidone Tab 0.3-15 MG |
| 00904103060 | Clonidine & Chlorthalidone | Clonidine & Chlorthalidone Tab 0.1-15 MG |
| 00904103160 | Clonidine & Chlorthalidone | Clonidine & Chlorthalidone Tab 0.2-15 MG |
| 00904103260 | Clonidine & Chlorthalidone | Clonidine & Chlorthalidone Tab 0.3-15 MG |
| 00904103360 | Clonidine & Chlorthalidone | Clonidine & Chlorthalidone Tab 0.1-15 MG |
| 00904103460 | Clonidine & Chlorthalidone | Clonidine & Chlorthalidone Tab 0.2-15 MG |
| 00904103560 | Clonidine & Chlorthalidone | Clonidine & Chlorthalidone Tab 0.3-15 MG |
| 00677114601 | Clonidine & Chlorthalidone | Clonidine & Chlorthalidone Tab 0.1-15 MG |
| 00677114701 | Clonidine & Chlorthalidone | Clonidine & Chlorthalidone Tab 0.2-15 MG |
| 00677114801 | Clonidine & Chlorthalidone | Clonidine & Chlorthalidone Tab 0.3-15 MG |
| 00719125610 | Clonidine & Chlorthalidone | Clonidine & Chlorthalidone Tab 0.1-15 MG |
| 00719125710 | Clonidine & Chlorthalidone | Clonidine & Chlorthalidone Tab 0.3-15 MG |
| 00719125810 | Clonidine & Chlorthalidone | Clonidine & Chlorthalidone Tab 0.3-15 MG |
| 00781103701 | Clonidine & Chlorthalidone | Clonidine & Chlorthalidone Tab 0.1-15 MG |
| 00781103801 | Clonidine & Chlorthalidone | Clonidine & Chlorthalidone Tab 0.2-15 MG |
| 00781103901 | Clonidine & Chlorthalidone | Clonidine & Chlorthalidone Tab 0.3-15 MG |
| 00536353301 | Clonidine & Chlorthalidone | Clonidine & Chlorthalidone Tab 0.1-15 MG |
| 00536353501 | Clonidine & Chlorthalidone | Clonidine & Chlorthalidone Tab 0.2-15 MG |
| 00536493701 | Clonidine & Chlorthalidone | Clonidine & Chlorthalidone Tab 0.1-15 MG |
| 00536493801 | Clonidine & Chlorthalidone | Clonidine & Chlorthalidone Tab 0.2-15 MG |
| 00597000810 | Clonidine & Chlorthalidone | Clonidine & Chlorthalidone Tab 0.1-15 MG |
| 00597000910 | Clonidine & Chlorthalidone | Clonidine & Chlorthalidone Tab 0.2-15 MG |
| 00597001001 | Clonidine & Chlorthalidone | Clonidine & Chlorthalidone Tab 0.3-15 MG |
| 00603297821 | Clonidine & Chlorthalidone | Clonidine & Chlorthalidone Tab 0.1-15 MG |
| 00603297921 | Clonidine & Chlorthalidone | Clonidine & Chlorthalidone Tab 0.2-15 MG |
| 00603298021 | Clonidine & Chlorthalidone | Clonidine & Chlorthalidone Tab 0.3-15 MG |
| 00349866101 | Clonidine & Chlorthalidone | Clonidine & Chlorthalidone Tab 0.2-15 MG |
| 00349866201 | Clonidine & Chlorthalidone | Clonidine & Chlorthalidone Tab 0.3-15 MG |
| 00364217401 | Clonidine & Chlorthalidone | Clonidine & Chlorthalidone Tab 0.1-15 MG |
| 00364217501 | Clonidine & Chlorthalidone | Clonidine & Chlorthalidone Tab 0.2-15 MG |
| 00364217601 | Clonidine & Chlorthalidone | Clonidine & Chlorthalidone Tab 0.3-15 MG |
| 00378000101 | Clonidine & Chlorthalidone | Clonidine & Chlorthalidone Tab 0.1-15 MG |
| 00378000110 | Clonidine & Chlorthalidone | Clonidine & Chlorthalidone Tab 0.1-15 MG |
| 00378002701 | Clonidine & Chlorthalidone | Clonidine & Chlorthalidone Tab 0.2-15 MG |
| 00378002710 | Clonidine & Chlorthalidone | Clonidine & Chlorthalidone Tab 0.2-15 MG |
| 16714034102 | Clonidine HCl | Clonidine HCl Tab 0.1 MG |
| 16714034103 | Clonidine HCl | Clonidine HCl Tab 0.1 MG |
| 16714034104 | Clonidine HCl | Clonidine HCl Tab 0.1 MG |
| 16714034202 | Clonidine HCl | Clonidine HCl Tab 0.2 MG |
| 16714034203 | Clonidine HCl | Clonidine HCl Tab 0.2 MG |
| 16714034204 | Clonidine HCl | Clonidine HCl Tab 0.2 MG |
| 16714034303 | Clonidine HCl | Clonidine HCl Tab 0.3 MG |
| 16714034304 | Clonidine HCl | Clonidine HCl Tab 0.3 MG |
| 54868631000 | Clonidine HCl | Clonidine HCl TD Patch Weekly 0.1 MG/24HR |
| 66336091930 | Clonidine HCl | Clonidine HCl Tab 0.3 MG |
| 54868004906 | Clonidine HCl | Clonidine HCl Tab 0.2 MG |
| 50436352301 | Clonidine HCl | Clonidine HCl Tab 0.1 MG |
| 54569047808 | Clonidine HCl | Clonidine HCl Tab 0.1 MG |
| 54569185305 | Clonidine HCl | Clonidine HCl Tab 0.2 MG |
| 54569280101 | Clonidine HCl | Clonidine HCl Tab 0.3 MG |
| 52372811105 | Clonidine HCl | Clonidine HCl Powder |
| 52372811125 | Clonidine HCl | Clonidine HCl Powder |
| 43353066160 | Clonidine HCl | Clonidine HCl Tab 0.2 MG |
| 43353066180 | Clonidine HCl | Clonidine HCl Tab 0.2 MG |
| 33261062330 | Clonidine HCl | Clonidine HCl Tab 0.2 MG |
| 33261062360 | Clonidine HCl | Clonidine HCl Tab 0.2 MG |
| 33261062390 | Clonidine HCl | Clonidine HCl Tab 0.2 MG |
| 47463007730 | Clonidine HCl | Clonidine HCl Tab 0.1 MG |
| 47463007790 | Clonidine HCl | Clonidine HCl Tab 0.1 MG |
| 24478030120 | Clonidine HCl | Clonidine HCl Tab SR 24HR 0.17 MG (Base Equivalent) |
| 24478030210 | Clonidine HCl | Clonidine HCl Extended Release Susp 0.09 MG/ML (Base Equiv) |
| 54868621400 | Clonidine HCl | Clonidine HCl TD Patch Weekly 0.2 MG/24HR |
| 63275997405 | Clonidine HCl | Clonidine HCl Powder |
| 47463010230 | Clonidine HCl | Clonidine HCl Tab 0.2 MG |
| 33261049500 | Clonidine HCl | Clonidine HCl Tab 0.1 MG |
| 43353066080 | Clonidine HCl | Clonidine HCl Tab 0.1 MG |
| 00603295702 | Clonidine HCl | Clonidine HCl Tab 0.1 MG |
| 00603295704 | Clonidine HCl | Clonidine HCl Tab 0.1 MG |
| 66336078645 | Clonidine HCl | Clonidine HCl Tab 0.1 MG |
| 55289007415 | Clonidine HCl | Clonidine HCl Tab 0.2 MG |
| 52372811110 | Clonidine HCl | Clonidine HCl Powder |
| 66336078610 | Clonidine HCl | Clonidine HCl Tab 0.1 MG |
| 62991142203 | Clonidine HCl | Clonidine HCl Powder |
| 63629132806 | Clonidine HCl | Clonidine HCl Tab 0.1 MG |
| 60760002730 | Clonidine HCl | Clonidine HCl Tab 0.1 MG |
| 51138025305 | Clonidine HCl | Clonidine HCl Tab 0.1 MG |
| 51138025315 | Clonidine HCl | Clonidine HCl Tab 0.1 MG |
| 51138025320 | Clonidine HCl | Clonidine HCl Tab 0.1 MG |
| 51138025330 | Clonidine HCl | Clonidine HCl Tab 0.1 MG |
| 51138025430 | Clonidine HCl | Clonidine HCl Tab 0.2 MG |
| 51138025530 | Clonidine HCl | Clonidine HCl Tab 0.3 MG |
| 51138001730 | Clonidine HCl | Clonidine HCl Tab 0.1 MG |
| 51138001830 | Clonidine HCl | Clonidine HCl Tab 0.2 MG |
| 51138001930 | Clonidine HCl | Clonidine HCl Tab 0.3 MG |
| 62991142204 | Clonidine HCl | Clonidine HCl Powder |
| 16590026672 | Clonidine HCl | Clonidine HCl Tab 0.1 MG |
| 16590026715 | Clonidine HCl | Clonidine HCl Tab 0.2 MG |
| 55048007730 | Clonidine HCl | Clonidine HCl Tab 0.1 MG |
| 55048007790 | Clonidine HCl | Clonidine HCl Tab 0.1 MG |
| 55048010230 | Clonidine HCl | Clonidine HCl Tab 0.2 MG |
| 43353050980 | Clonidine HCl | Clonidine HCl Tab 0.2 MG |
| 49884077486 | Clonidine HCl | Clonidine HCl TD Patch Weekly 0.1 MG/24HR |
| 49884077586 | Clonidine HCl | Clonidine HCl TD Patch Weekly 0.2 MG/24HR |
| 49884077686 | Clonidine HCl | Clonidine HCl TD Patch Weekly 0.3 MG/24HR |
| 51079029966 | Clonidine HCl | Clonidine HCl Tab 0.1 MG |
| 51079030066 | Clonidine HCl | Clonidine HCl Tab 0.2 MG |
| 55289007490 | Clonidine HCl | Clonidine HCl Tab 0.2 MG |
| 66267046430 | Clonidine HCl | Clonidine HCl Tab 0.3 MG |
| 66267046460 | Clonidine HCl | Clonidine HCl Tab 0.3 MG |
| 66267006112 | Clonidine HCl | Clonidine HCl Tab 0.1 MG |
| 66267006130 | Clonidine HCl | Clonidine HCl Tab 0.1 MG |
| 66267006160 | Clonidine HCl | Clonidine HCl Tab 0.1 MG |
| 66267006190 | Clonidine HCl | Clonidine HCl Tab 0.1 MG |
| 66267006214 | Clonidine HCl | Clonidine HCl Tab 0.2 MG |
| 66267006230 | Clonidine HCl | Clonidine HCl Tab 0.2 MG |
| 66267006260 | Clonidine HCl | Clonidine HCl Tab 0.2 MG |
| 66267006290 | Clonidine HCl | Clonidine HCl Tab 0.2 MG |
| 66267006292 | Clonidine HCl | Clonidine HCl Tab 0.2 MG |
| 21695037260 | Clonidine HCl | Clonidine HCl Tab 0.2 MG |
| 55045116708 | Clonidine HCl | Clonidine HCl Tab 0.1 MG |
| 33261049502 | Clonidine HCl | Clonidine HCl Tab 0.1 MG |
| 33261049530 | Clonidine HCl | Clonidine HCl Tab 0.1 MG |
| 33261049560 | Clonidine HCl | Clonidine HCl Tab 0.1 MG |
| 33261049590 | Clonidine HCl | Clonidine HCl Tab 0.1 MG |
| 60429005001 | Clonidine HCl | Clonidine HCl Tab 0.1 MG |
| 60429005010 | Clonidine HCl | Clonidine HCl Tab 0.1 MG |
| 60429005101 | Clonidine HCl | Clonidine HCl Tab 0.2 MG |
| 60429005110 | Clonidine HCl | Clonidine HCl Tab 0.2 MG |
| 60429006001 | Clonidine HCl | Clonidine HCl Tab 0.3 MG |
| 63629430501 | Clonidine HCl | Clonidine HCl Tab 0.3 MG |
| 00378087199 | Clonidine HCl | Clonidine HCl TD Patch Weekly 0.1 MG/24HR |
| 00378087299 | Clonidine HCl | Clonidine HCl TD Patch Weekly 0.2 MG/24HR |
| 00378087399 | Clonidine HCl | Clonidine HCl TD Patch Weekly 0.3 MG/24HR |
| 00555100916 | Clonidine HCl | Clonidine HCl TD Patch Weekly 0.1 MG/24HR |
| 00555101016 | Clonidine HCl | Clonidine HCl TD Patch Weekly 0.2 MG/24HR |
| 00555101116 | Clonidine HCl | Clonidine HCl TD Patch Weekly 0.3 MG/24HR |
| 29300013510 | Clonidine HCl | Clonidine HCl Tab 0.1 MG |
| 29300013601 | Clonidine HCl | Clonidine HCl Tab 0.2 MG |
| 29300013701 | Clonidine HCl | Clonidine HCl Tab 0.3 MG |
| 63629132804 | Clonidine HCl | Clonidine HCl Tab 0.1 MG |
| 00603295730 | Clonidine HCl | Clonidine HCl Tab 0.1 MG |
| 00603295830 | Clonidine HCl | Clonidine HCl Tab 0.2 MG |
| 16590026730 | Clonidine HCl | Clonidine HCl Tab 0.2 MG |
| 16590026760 | Clonidine HCl | Clonidine HCl Tab 0.2 MG |
| 16590026790 | Clonidine HCl | Clonidine HCl Tab 0.2 MG |
| 21695037160 | Clonidine HCl | Clonidine HCl Tab 0.1 MG |
| 62584065733 | Clonidine HCl | Clonidine HCl Tab 0.1 MG |
| 00597003234 | Clonidine HCl | Clonidine HCl TD Patch Weekly 0.2 MG/24HR |
| 33358009200 | Clonidine HCl | Clonidine HCl Tab 0.1 MG |
| 33358009230 | Clonidine HCl | Clonidine HCl Tab 0.1 MG |
| 33358009330 | Clonidine HCl | Clonidine HCl Tab 0.2 MG |
| 33358009360 | Clonidine HCl | Clonidine HCl Tab 0.2 MG |
| 18837027230 | Clonidine HCl | Clonidine HCl Tab 0.1 MG |
| 18837028204 | Clonidine HCl | Clonidine HCl TD Patch Weekly 0.2 MG/24HR |
| 18837029204 | Clonidine HCl | Clonidine HCl TD Patch Weekly 0.1 MG/24HR |
| 54868196704 | Clonidine HCl | Clonidine HCl Tab 0.3 MG |
| 54569047807 | Clonidine HCl | Clonidine HCl Tab 0.1 MG |
| 16590047630 | Clonidine HCl | Clonidine HCl TD Patch Weekly 0.3 MG/24HR |
| 16590047704 | Clonidine HCl | Clonidine HCl TD Patch Weekly 0.1 MG/24HR |
| 16590047712 | Clonidine HCl | Clonidine HCl TD Patch Weekly 0.1 MG/24HR |
| 54569185304 | Clonidine HCl | Clonidine HCl Tab 0.2 MG |
| 21695037230 | Clonidine HCl | Clonidine HCl Tab 0.2 MG |
| 57866352404 | Clonidine HCl | Clonidine HCl Tab 0.2 MG |
| 00440732515 | Clonidine HCl | Clonidine HCl Tab 0.1 MG |
| 00440732630 | Clonidine HCl | Clonidine HCl Tab 0.2 MG |
| 00440732706 | Clonidine HCl | Clonidine HCl Tab 0.3 MG |
| 55289097030 | Clonidine HCl | Clonidine HCl Tab 0.3 MG |
| 49999012790 | Clonidine HCl | Clonidine HCl Tab 0.1 MG |
| 55289007320 | Clonidine HCl | Clonidine HCl Tab 0.1 MG |
| 63629132801 | Clonidine HCl | Clonidine HCl Tab 0.1 MG |
| 63629132802 | Clonidine HCl | Clonidine HCl Tab 0.1 MG |
| 63629132803 | Clonidine HCl | Clonidine HCl Tab 0.1 MG |
| 63629275301 | Clonidine HCl | Clonidine HCl Tab 0.2 MG |
| 63629275302 | Clonidine HCl | Clonidine HCl Tab 0.2 MG |
| 63629275303 | Clonidine HCl | Clonidine HCl Tab 0.2 MG |
| 51079030030 | Clonidine HCl | Clonidine HCl Tab 0.2 MG |
| 51079030056 | Clonidine HCl | Clonidine HCl Tab 0.2 MG |
| 55887047510 | Clonidine HCl | Clonidine HCl Tab 0.2 MG |
| 55289007393 | Clonidine HCl | Clonidine HCl Tab 0.1 MG |
| 38779056105 | Clonidine HCl | Clonidine HCl Powder |
| 21695037100 | Clonidine HCl | Clonidine HCl Tab 0.1 MG |
| 18837027298 | Clonidine HCl | Clonidine HCl Tab 0.1 MG |
| 63874047590 | Clonidine HCl | Clonidine HCl Tab 0.1 MG |
| 66267006191 | Clonidine HCl | Clonidine HCl Tab 0.1 MG |
| 12634046571 | Clonidine HCl | Clonidine HCl Tab 0.1 MG |
| 21695037115 | Clonidine HCl | Clonidine HCl Tab 0.1 MG |
| 21695037190 | Clonidine HCl | Clonidine HCl Tab 0.1 MG |
| 21695037290 | Clonidine HCl | Clonidine HCl Tab 0.2 MG |
| 21695037730 | Clonidine HCl | Clonidine HCl Tab 0.3 MG |
| 43353050960 | Clonidine HCl | Clonidine HCl Tab 0.2 MG |
| 60429005018 | Clonidine HCl | Clonidine HCl Tab 0.1 MG |
| 60429005027 | Clonidine HCl | Clonidine HCl Tab 0.1 MG |
| 60429005060 | Clonidine HCl | Clonidine HCl Tab 0.1 MG |
| 60429005090 | Clonidine HCl | Clonidine HCl Tab 0.1 MG |
| 60429005118 | Clonidine HCl | Clonidine HCl Tab 0.2 MG |
| 60429005160 | Clonidine HCl | Clonidine HCl Tab 0.2 MG |
| 60429005190 | Clonidine HCl | Clonidine HCl Tab 0.2 MG |
| 60809011455 | Clonidine HCl | Clonidine HCl Tab 0.1 MG |
| 60809011472 | Clonidine HCl | Clonidine HCl Tab 0.1 MG |
| 61392051330 | Clonidine HCl | Clonidine HCl Tab 0.1 MG |
| 61392051331 | Clonidine HCl | Clonidine HCl Tab 0.1 MG |
| 61392051332 | Clonidine HCl | Clonidine HCl Tab 0.1 MG |
| 61392051339 | Clonidine HCl | Clonidine HCl Tab 0.1 MG |
| 61392051345 | Clonidine HCl | Clonidine HCl Tab 0.1 MG |
| 61392051351 | Clonidine HCl | Clonidine HCl Tab 0.1 MG |
| 61392051354 | Clonidine HCl | Clonidine HCl Tab 0.1 MG |
| 61392051356 | Clonidine HCl | Clonidine HCl Tab 0.1 MG |
| 61392051360 | Clonidine HCl | Clonidine HCl Tab 0.1 MG |
| 61392051390 | Clonidine HCl | Clonidine HCl Tab 0.1 MG |
| 61392051391 | Clonidine HCl | Clonidine HCl Tab 0.1 MG |
| 61392051630 | Clonidine HCl | Clonidine HCl Tab 0.2 MG |
| 61392051631 | Clonidine HCl | Clonidine HCl Tab 0.2 MG |
| 61392051632 | Clonidine HCl | Clonidine HCl Tab 0.2 MG |
| 61392051639 | Clonidine HCl | Clonidine HCl Tab 0.2 MG |
| 61392051645 | Clonidine HCl | Clonidine HCl Tab 0.2 MG |
| 61392051651 | Clonidine HCl | Clonidine HCl Tab 0.2 MG |
| 61392051654 | Clonidine HCl | Clonidine HCl Tab 0.2 MG |
| 61392051656 | Clonidine HCl | Clonidine HCl Tab 0.2 MG |
| 61392051660 | Clonidine HCl | Clonidine HCl Tab 0.2 MG |
| 61392051690 | Clonidine HCl | Clonidine HCl Tab 0.2 MG |
| 61392051691 | Clonidine HCl | Clonidine HCl Tab 0.2 MG |
| 61392051930 | Clonidine HCl | Clonidine HCl Tab 0.3 MG |
| 61392051931 | Clonidine HCl | Clonidine HCl Tab 0.3 MG |
| 61392051932 | Clonidine HCl | Clonidine HCl Tab 0.3 MG |
| 61392051939 | Clonidine HCl | Clonidine HCl Tab 0.3 MG |
| 61392051945 | Clonidine HCl | Clonidine HCl Tab 0.3 MG |
| 61392051951 | Clonidine HCl | Clonidine HCl Tab 0.3 MG |
| 61392051954 | Clonidine HCl | Clonidine HCl Tab 0.3 MG |
| 61392051956 | Clonidine HCl | Clonidine HCl Tab 0.3 MG |
| 61392051960 | Clonidine HCl | Clonidine HCl Tab 0.3 MG |
| 61392051990 | Clonidine HCl | Clonidine HCl Tab 0.3 MG |
| 61392051991 | Clonidine HCl | Clonidine HCl Tab 0.3 MG |
| 62584033933 | Clonidine HCl | Clonidine HCl Tab 0.2 MG |
| 57801030322 | Clonidine HCl | Clonidine HCl Tab 0.1 MG |
| 57801030422 | Clonidine HCl | Clonidine HCl Tab 0.2 MG |
| 57801030522 | Clonidine HCl | Clonidine HCl Tab 0.3 MG |
| 57362041084 | Clonidine HCl | Clonidine HCl Tab 0.2 MG |
| 57480030901 | Clonidine HCl | Clonidine HCl Tab 0.1 MG |
| 57480030906 | Clonidine HCl | Clonidine HCl Tab 0.1 MG |
| 57480031001 | Clonidine HCl | Clonidine HCl Tab 0.2 MG |
| 57480031006 | Clonidine HCl | Clonidine HCl Tab 0.2 MG |
| 57480031101 | Clonidine HCl | Clonidine HCl Tab 0.3 MG |
| 57480031106 | Clonidine HCl | Clonidine HCl Tab 0.3 MG |
| 58016051800 | Clonidine HCl | Clonidine HCl Tab 0.2 MG |
| 58016051810 | Clonidine HCl | Clonidine HCl Tab 0.2 MG |
| 58016051812 | Clonidine HCl | Clonidine HCl Tab 0.2 MG |
| 58016051814 | Clonidine HCl | Clonidine HCl Tab 0.2 MG |
| 58016051820 | Clonidine HCl | Clonidine HCl Tab 0.2 MG |
| 58016051821 | Clonidine HCl | Clonidine HCl Tab 0.2 MG |
| 58016051824 | Clonidine HCl | Clonidine HCl Tab 0.2 MG |
| 58016051828 | Clonidine HCl | Clonidine HCl Tab 0.2 MG |
| 58016051830 | Clonidine HCl | Clonidine HCl Tab 0.2 MG |
| 58016051840 | Clonidine HCl | Clonidine HCl Tab 0.2 MG |
| 58016051860 | Clonidine HCl | Clonidine HCl Tab 0.2 MG |
| 58016060500 | Clonidine HCl | Clonidine HCl Tab 0.3 MG |
| 58016060512 | Clonidine HCl | Clonidine HCl Tab 0.3 MG |
| 58016060515 | Clonidine HCl | Clonidine HCl Tab 0.3 MG |
| 58016060520 | Clonidine HCl | Clonidine HCl Tab 0.3 MG |
| 58016060530 | Clonidine HCl | Clonidine HCl Tab 0.3 MG |
| 58016060560 | Clonidine HCl | Clonidine HCl Tab 0.3 MG |
| 58016501201 | Clonidine HCl | Clonidine HCl TD Patch Weekly 0.1 MG/24HR |
| 54569104000 | Clonidine HCl | Clonidine HCl TD Patch Weekly 0.3 MG/24HR |
| 54569185302 | Clonidine HCl | Clonidine HCl Tab 0.2 MG |
| 54569185303 | Clonidine HCl | Clonidine HCl Tab 0.2 MG |
| 54569700900 | Clonidine HCl | Clonidine HCl Tab 0.1 MG |
| 54807011101 | Clonidine HCl | Clonidine HCl Tab 0.1 MG |
| 54807011201 | Clonidine HCl | Clonidine HCl Tab 0.2 MG |
| 54807011301 | Clonidine HCl | Clonidine HCl Tab 0.3 MG |
| 54977006030 | Clonidine HCl | Clonidine HCl Tab 0.1 MG |
| 54977006099 | Clonidine HCl | Clonidine HCl Tab 0.1 MG |
| 54977006130 | Clonidine HCl | Clonidine HCl Tab 0.2 MG |
| 55081012600 | Clonidine HCl | Clonidine HCl Tab 0.1 MG |
| 55081012601 | Clonidine HCl | Clonidine HCl Tab 0.1 MG |
| 55081012602 | Clonidine HCl | Clonidine HCl Tab 0.1 MG |
| 55081012700 | Clonidine HCl | Clonidine HCl Tab 0.2 MG |
| 55081012701 | Clonidine HCl | Clonidine HCl Tab 0.2 MG |
| 55081012702 | Clonidine HCl | Clonidine HCl Tab 0.2 MG |
| 55081060300 | Clonidine HCl | Clonidine HCl Tab 0.3 MG |
| 55829021110 | Clonidine HCl | Clonidine HCl Tab 0.1 MG |
| 55829021210 | Clonidine HCl | Clonidine HCl Tab 0.2 MG |
| 55829021310 | Clonidine HCl | Clonidine HCl Tab 0.3 MG |
| 55175445001 | Clonidine HCl | Clonidine HCl Tab 0.1 MG |
| 55175445003 | Clonidine HCl | Clonidine HCl Tab 0.1 MG |
| 55175445006 | Clonidine HCl | Clonidine HCl Tab 0.1 MG |
| 55175445106 | Clonidine HCl | Clonidine HCl Tab 0.2 MG |
| 55175445201 | Clonidine HCl | Clonidine HCl Tab 0.3 MG |
| 55947040930 | Clonidine HCl | Clonidine HCl Tab 0.1 MG |
| 55947041030 | Clonidine HCl | Clonidine HCl Tab 0.2 MG |
| 57362040913 | Clonidine HCl | Clonidine HCl Tab 0.1 MG |
| 57362040919 | Clonidine HCl | Clonidine HCl Tab 0.1 MG |
| 57362040984 | Clonidine HCl | Clonidine HCl Tab 0.1 MG |
| 57362041013 | Clonidine HCl | Clonidine HCl Tab 0.2 MG |
| 57362041019 | Clonidine HCl | Clonidine HCl Tab 0.2 MG |
| 52555033001 | Clonidine HCl | Clonidine HCl Tab 0.3 MG |
| 53258019101 | Clonidine HCl | Clonidine HCl Tab 0.1 MG |
| 53258019113 | Clonidine HCl | Clonidine HCl Tab 0.1 MG |
| 53258022101 | Clonidine HCl | Clonidine HCl Tab 0.2 MG |
| 53258022113 | Clonidine HCl | Clonidine HCl Tab 0.2 MG |
| 53445176700 | Clonidine HCl | Clonidine HCl Tab 0.1 MG |
| 53445176800 | Clonidine HCl | Clonidine HCl Tab 0.2 MG |
| 53445176901 | Clonidine HCl | Clonidine HCl Tab 0.3 MG |
| 53746004501 | Clonidine HCl | Clonidine HCl Tab 0.1 MG |
| 53746004510 | Clonidine HCl | Clonidine HCl Tab 0.1 MG |
| 53746004601 | Clonidine HCl | Clonidine HCl Tab 0.2 MG |
| 53746004610 | Clonidine HCl | Clonidine HCl Tab 0.2 MG |
| 53746004701 | Clonidine HCl | Clonidine HCl Tab 0.3 MG |
| 53746004710 | Clonidine HCl | Clonidine HCl Tab 0.3 MG |
| 54124076930 | Clonidine HCl | Clonidine HCl Tab 0.2 MG |
| 54274005110 | Clonidine HCl | Clonidine HCl Tab 0.2 MG |
| 54274005150 | Clonidine HCl | Clonidine HCl Tab 0.2 MG |
| 54274066610 | Clonidine HCl | Clonidine HCl Tab 0.1 MG |
| 54274066650 | Clonidine HCl | Clonidine HCl Tab 0.1 MG |
| 54274066710 | Clonidine HCl | Clonidine HCl Tab 0.2 MG |
| 54274066750 | Clonidine HCl | Clonidine HCl Tab 0.2 MG |
| 54274066810 | Clonidine HCl | Clonidine HCl Tab 0.3 MG |
| 54274066830 | Clonidine HCl | Clonidine HCl Tab 0.3 MG |
| 54274020810 | Clonidine HCl | Clonidine HCl Tab 0.1 MG |
| 54274020850 | Clonidine HCl | Clonidine HCl Tab 0.1 MG |
| 54274020910 | Clonidine HCl | Clonidine HCl Tab 0.2 MG |
| 54274020930 | Clonidine HCl | Clonidine HCl Tab 0.2 MG |
| 54274020950 | Clonidine HCl | Clonidine HCl Tab 0.2 MG |
| 54274021010 | Clonidine HCl | Clonidine HCl Tab 0.3 MG |
| 54274021030 | Clonidine HCl | Clonidine HCl Tab 0.3 MG |
| 54274021050 | Clonidine HCl | Clonidine HCl Tab 0.3 MG |
| 54569047803 | Clonidine HCl | Clonidine HCl Tab 0.1 MG |
| 54569047805 | Clonidine HCl | Clonidine HCl Tab 0.1 MG |
| 54569047806 | Clonidine HCl | Clonidine HCl Tab 0.1 MG |
| 54569052600 | Clonidine HCl | Clonidine HCl Tab 0.3 MG |
| 54569052700 | Clonidine HCl | Clonidine HCl Tab 0.1 MG |
| 54569052701 | Clonidine HCl | Clonidine HCl Tab 0.1 MG |
| 54569052800 | Clonidine HCl | Clonidine HCl Tab 0.2 MG |
| 54569052900 | Clonidine HCl | Clonidine HCl TD Patch Weekly 0.2 MG/24HR |
| 52246060312 | Clonidine HCl | Clonidine HCl Tab 0.1 MG |
| 52246060360 | Clonidine HCl | Clonidine HCl Tab 0.1 MG |
| 52246061060 | Clonidine HCl | Clonidine HCl Tab 0.2 MG |
| 52246061090 | Clonidine HCl | Clonidine HCl Tab 0.2 MG |
| 52246068290 | Clonidine HCl | Clonidine HCl Tab 0.3 MG |
| 52446012121 | Clonidine HCl | Clonidine HCl Tab 0.1 MG |
| 52446012132 | Clonidine HCl | Clonidine HCl Tab 0.1 MG |
| 52446012221 | Clonidine HCl | Clonidine HCl Tab 0.2 MG |
| 52446012232 | Clonidine HCl | Clonidine HCl Tab 0.2 MG |
| 52446012521 | Clonidine HCl | Clonidine HCl Tab 0.3 MG |
| 52446012528 | Clonidine HCl | Clonidine HCl Tab 0.3 MG |
| 52555001101 | Clonidine HCl | Clonidine HCl Tab 0.2 MG |
| 52555001105 | Clonidine HCl | Clonidine HCl Tab 0.2 MG |
| 52555001110 | Clonidine HCl | Clonidine HCl Tab 0.2 MG |
| 52555011001 | Clonidine HCl | Clonidine HCl Tab 0.1 MG |
| 52555011005 | Clonidine HCl | Clonidine HCl Tab 0.1 MG |
| 52555011010 | Clonidine HCl | Clonidine HCl Tab 0.1 MG |
| 52555011201 | Clonidine HCl | Clonidine HCl Tab 0.3 MG |
| 52555011205 | Clonidine HCl | Clonidine HCl Tab 0.3 MG |
| 52493060601 | Clonidine HCl | Clonidine HCl Tab 0.1 MG |
| 52493060602 | Clonidine HCl | Clonidine HCl Tab 0.1 MG |
| 52493060630 | Clonidine HCl | Clonidine HCl Tab 0.1 MG |
| 52493060660 | Clonidine HCl | Clonidine HCl Tab 0.1 MG |
| 52493060701 | Clonidine HCl | Clonidine HCl Tab 0.2 MG |
| 52493060730 | Clonidine HCl | Clonidine HCl Tab 0.2 MG |
| 52493060760 | Clonidine HCl | Clonidine HCl Tab 0.2 MG |
| 52555032801 | Clonidine HCl | Clonidine HCl Tab 0.1 MG |
| 52555032810 | Clonidine HCl | Clonidine HCl Tab 0.1 MG |
| 52555032901 | Clonidine HCl | Clonidine HCl Tab 0.2 MG |
| 52555032910 | Clonidine HCl | Clonidine HCl Tab 0.2 MG |
| 49884011001 | Clonidine HCl | Clonidine HCl Tab 0.1 MG |
| 49884011005 | Clonidine HCl | Clonidine HCl Tab 0.1 MG |
| 49884011010 | Clonidine HCl | Clonidine HCl Tab 0.1 MG |
| 49884011101 | Clonidine HCl | Clonidine HCl Tab 0.2 MG |
| 49884011105 | Clonidine HCl | Clonidine HCl Tab 0.2 MG |
| 49884011110 | Clonidine HCl | Clonidine HCl Tab 0.2 MG |
| 49884011205 | Clonidine HCl | Clonidine HCl Tab 0.3 MG |
| 49884011210 | Clonidine HCl | Clonidine HCl Tab 0.3 MG |
| 50349014801 | Clonidine HCl | Clonidine HCl Tab 0.1 MG |
| 50349014805 | Clonidine HCl | Clonidine HCl Tab 0.1 MG |
| 50349014810 | Clonidine HCl | Clonidine HCl Tab 0.1 MG |
| 50349014901 | Clonidine HCl | Clonidine HCl Tab 0.2 MG |
| 50349014905 | Clonidine HCl | Clonidine HCl Tab 0.2 MG |
| 50349014910 | Clonidine HCl | Clonidine HCl Tab 0.2 MG |
| 50349015001 | Clonidine HCl | Clonidine HCl Tab 0.3 MG |
| 50349015005 | Clonidine HCl | Clonidine HCl Tab 0.3 MG |
| 50349015010 | Clonidine HCl | Clonidine HCl Tab 0.3 MG |
| 51285021102 | Clonidine HCl | Clonidine HCl Tab 0.1 MG |
| 51285021105 | Clonidine HCl | Clonidine HCl Tab 0.1 MG |
| 51285021202 | Clonidine HCl | Clonidine HCl Tab 0.2 MG |
| 51285021205 | Clonidine HCl | Clonidine HCl Tab 0.2 MG |
| 51285021302 | Clonidine HCl | Clonidine HCl Tab 0.3 MG |
| 51309078391 | Clonidine HCl | Clonidine HCl Tab 0.1 MG |
| 51309078591 | Clonidine HCl | Clonidine HCl Tab 0.3 MG |
| 51432008703 | Clonidine HCl | Clonidine HCl Tab 0.1 MG |
| 51432008706 | Clonidine HCl | Clonidine HCl Tab 0.1 MG |
| 51432008903 | Clonidine HCl | Clonidine HCl Tab 0.2 MG |
| 51432008906 | Clonidine HCl | Clonidine HCl Tab 0.2 MG |
| 51432009103 | Clonidine HCl | Clonidine HCl Tab 0.3 MG |
| 51432009106 | Clonidine HCl | Clonidine HCl Tab 0.3 MG |
| 11845019201 | Clonidine HCl | Clonidine HCl Tab 0.2 MG |
| 11845019204 | Clonidine HCl | Clonidine HCl Tab 0.2 MG |
| 11845019301 | Clonidine HCl | Clonidine HCl Tab 0.3 MG |
| 17236059201 | Clonidine HCl | Clonidine HCl Tab 0.1 MG |
| 17236059210 | Clonidine HCl | Clonidine HCl Tab 0.1 MG |
| 17236059301 | Clonidine HCl | Clonidine HCl Tab 0.2 MG |
| 17236059310 | Clonidine HCl | Clonidine HCl Tab 0.2 MG |
| 17236059401 | Clonidine HCl | Clonidine HCl Tab 0.3 MG |
| 17236059405 | Clonidine HCl | Clonidine HCl Tab 0.3 MG |
| 24208011501 | Clonidine HCl | Clonidine HCl Tab 0.1 MG |
| 24208011505 | Clonidine HCl | Clonidine HCl Tab 0.1 MG |
| 24208011510 | Clonidine HCl | Clonidine HCl Tab 0.1 MG |
| 24208011511 | Clonidine HCl | Clonidine HCl Tab 0.1 MG |
| 24208011601 | Clonidine HCl | Clonidine HCl Tab 0.2 MG |
| 24208011605 | Clonidine HCl | Clonidine HCl Tab 0.2 MG |
| 24208011610 | Clonidine HCl | Clonidine HCl Tab 0.2 MG |
| 24208011611 | Clonidine HCl | Clonidine HCl Tab 0.2 MG |
| 24208011701 | Clonidine HCl | Clonidine HCl Tab 0.3 MG |
| 24208011710 | Clonidine HCl | Clonidine HCl Tab 0.3 MG |
| 24208011711 | Clonidine HCl | Clonidine HCl Tab 0.3 MG |
| 38779056111 | Clonidine HCl | Clonidine HCl Powder |
| 38779056115 | Clonidine HCl | Clonidine HCl Powder |
| 40893060601 | Clonidine HCl | Clonidine HCl Tab 0.1 MG |
| 40893060602 | Clonidine HCl | Clonidine HCl Tab 0.1 MG |
| 40893060630 | Clonidine HCl | Clonidine HCl Tab 0.1 MG |
| 40893060660 | Clonidine HCl | Clonidine HCl Tab 0.1 MG |
| 40893060701 | Clonidine HCl | Clonidine HCl Tab 0.2 MG |
| 40893060730 | Clonidine HCl | Clonidine HCl Tab 0.2 MG |
| 40893060760 | Clonidine HCl | Clonidine HCl Tab 0.2 MG |
| 47202265101 | Clonidine HCl | Clonidine HCl Tab 0.1 MG |
| 47202265103 | Clonidine HCl | Clonidine HCl Tab 0.1 MG |
| 47202265201 | Clonidine HCl | Clonidine HCl Tab 0.2 MG |
| 47202265203 | Clonidine HCl | Clonidine HCl Tab 0.2 MG |
| 47202265301 | Clonidine HCl | Clonidine HCl Tab 0.3 MG |
| 47202265303 | Clonidine HCl | Clonidine HCl Tab 0.3 MG |
| 47202273501 | Clonidine HCl | Clonidine HCl Tab 0.3 MG |
| 47202273503 | Clonidine HCl | Clonidine HCl Tab 0.3 MG |
| 47202274801 | Clonidine HCl | Clonidine HCl Tab 0.1 MG |
| 47202274803 | Clonidine HCl | Clonidine HCl Tab 0.1 MG |
| 47202274901 | Clonidine HCl | Clonidine HCl Tab 0.2 MG |
| 47202274903 | Clonidine HCl | Clonidine HCl Tab 0.2 MG |
| 47679080801 | Clonidine HCl | Clonidine HCl Tab 0.1 MG |
| 47679080835 | Clonidine HCl | Clonidine HCl Tab 0.1 MG |
| 47679080901 | Clonidine HCl | Clonidine HCl Tab 0.2 MG |
| 47679080935 | Clonidine HCl | Clonidine HCl Tab 0.2 MG |
| 47679082701 | Clonidine HCl | Clonidine HCl Tab 0.3 MG |
| 47679082735 | Clonidine HCl | Clonidine HCl Tab 0.3 MG |
| 00725007901 | Clonidine HCl | Clonidine HCl Tab 0.1 MG |
| 00725007905 | Clonidine HCl | Clonidine HCl Tab 0.1 MG |
| 00725007910 | Clonidine HCl | Clonidine HCl Tab 0.1 MG |
| 00725008001 | Clonidine HCl | Clonidine HCl Tab 0.2 MG |
| 00725008005 | Clonidine HCl | Clonidine HCl Tab 0.2 MG |
| 00725008010 | Clonidine HCl | Clonidine HCl Tab 0.2 MG |
| 00725008101 | Clonidine HCl | Clonidine HCl Tab 0.3 MG |
| 00725008105 | Clonidine HCl | Clonidine HCl Tab 0.3 MG |
| 00725008110 | Clonidine HCl | Clonidine HCl Tab 0.3 MG |
| 00781147101 | Clonidine HCl | Clonidine HCl Tab 0.1 MG |
| 00781147110 | Clonidine HCl | Clonidine HCl Tab 0.1 MG |
| 00781147113 | Clonidine HCl | Clonidine HCl Tab 0.1 MG |
| 00781147201 | Clonidine HCl | Clonidine HCl Tab 0.2 MG |
| 00781147210 | Clonidine HCl | Clonidine HCl Tab 0.2 MG |
| 00781147213 | Clonidine HCl | Clonidine HCl Tab 0.2 MG |
| 00781147301 | Clonidine HCl | Clonidine HCl Tab 0.3 MG |
| 00814173014 | Clonidine HCl | Clonidine HCl Tab 0.1 MG |
| 00814173030 | Clonidine HCl | Clonidine HCl Tab 0.1 MG |
| 00814173114 | Clonidine HCl | Clonidine HCl Tab 0.2 MG |
| 00814173130 | Clonidine HCl | Clonidine HCl Tab 0.2 MG |
| 00814173214 | Clonidine HCl | Clonidine HCl Tab 0.3 MG |
| 00839718206 | Clonidine HCl | Clonidine HCl Tab 0.1 MG |
| 00839718212 | Clonidine HCl | Clonidine HCl Tab 0.1 MG |
| 00839718216 | Clonidine HCl | Clonidine HCl Tab 0.1 MG |
| 00839718306 | Clonidine HCl | Clonidine HCl Tab 0.2 MG |
| 00839718312 | Clonidine HCl | Clonidine HCl Tab 0.2 MG |
| 00839718316 | Clonidine HCl | Clonidine HCl Tab 0.2 MG |
| 00839718406 | Clonidine HCl | Clonidine HCl Tab 0.3 MG |
| 00839718412 | Clonidine HCl | Clonidine HCl Tab 0.3 MG |
| 00839718416 | Clonidine HCl | Clonidine HCl Tab 0.3 MG |
| 00904102260 | Clonidine HCl | Clonidine HCl Tab 0.1 MG |
| 00904102560 | Clonidine HCl | Clonidine HCl Tab 0.1 MG |
| 00904102561 | Clonidine HCl | Clonidine HCl Tab 0.1 MG |
| 00904102580 | Clonidine HCl | Clonidine HCl Tab 0.1 MG |
| 00904102640 | Clonidine HCl | Clonidine HCl Tab 0.2 MG |
| 00904102660 | Clonidine HCl | Clonidine HCl Tab 0.2 MG |
| 00904102680 | Clonidine HCl | Clonidine HCl Tab 0.2 MG |
| 00904102740 | Clonidine HCl | Clonidine HCl Tab 0.3 MG |
| 00904102760 | Clonidine HCl | Clonidine HCl Tab 0.3 MG |
| 00904102761 | Clonidine HCl | Clonidine HCl Tab 0.3 MG |
| 11845019101 | Clonidine HCl | Clonidine HCl Tab 0.1 MG |
| 11845019104 | Clonidine HCl | Clonidine HCl Tab 0.1 MG |
| 00615257201 | Clonidine HCl | Clonidine HCl Tab 0.1 MG |
| 00615257210 | Clonidine HCl | Clonidine HCl Tab 0.1 MG |
| 00615257213 | Clonidine HCl | Clonidine HCl Tab 0.1 MG |
| 00615257217 | Clonidine HCl | Clonidine HCl Tab 0.1 MG |
| 00615257229 | Clonidine HCl | Clonidine HCl Tab 0.1 MG |
| 00615257239 | Clonidine HCl | Clonidine HCl Tab 0.1 MG |
| 00615257265 | Clonidine HCl | Clonidine HCl Tab 0.1 MG |
| 00615257280 | Clonidine HCl | Clonidine HCl Tab 0.1 MG |
| 00615257301 | Clonidine HCl | Clonidine HCl Tab 0.2 MG |
| 00615257310 | Clonidine HCl | Clonidine HCl Tab 0.2 MG |
| 00615257313 | Clonidine HCl | Clonidine HCl Tab 0.2 MG |
| 00615257329 | Clonidine HCl | Clonidine HCl Tab 0.2 MG |
| 00615257332 | Clonidine HCl | Clonidine HCl Tab 0.2 MG |
| 00615257339 | Clonidine HCl | Clonidine HCl Tab 0.2 MG |
| 00615257343 | Clonidine HCl | Clonidine HCl Tab 0.2 MG |
| 00615257401 | Clonidine HCl | Clonidine HCl Tab 0.3 MG |
| 00615257410 | Clonidine HCl | Clonidine HCl Tab 0.3 MG |
| 00615257413 | Clonidine HCl | Clonidine HCl Tab 0.3 MG |
| 00615257439 | Clonidine HCl | Clonidine HCl Tab 0.3 MG |
| 00677107701 | Clonidine HCl | Clonidine HCl Tab 0.1 MG |
| 00677107710 | Clonidine HCl | Clonidine HCl Tab 0.1 MG |
| 00677107801 | Clonidine HCl | Clonidine HCl Tab 0.2 MG |
| 00677107810 | Clonidine HCl | Clonidine HCl Tab 0.2 MG |
| 00677111401 | Clonidine HCl | Clonidine HCl Tab 0.3 MG |
| 00677111410 | Clonidine HCl | Clonidine HCl Tab 0.3 MG |
| 00719125310 | Clonidine HCl | Clonidine HCl Tab 0.1 MG |
| 00719125312 | Clonidine HCl | Clonidine HCl Tab 0.1 MG |
| 00719125313 | Clonidine HCl | Clonidine HCl Tab 0.1 MG |
| 00719125410 | Clonidine HCl | Clonidine HCl Tab 0.2 MG |
| 00719125412 | Clonidine HCl | Clonidine HCl Tab 0.2 MG |
| 00719125413 | Clonidine HCl | Clonidine HCl Tab 0.2 MG |
| 00719125510 | Clonidine HCl | Clonidine HCl Tab 0.3 MG |
| 00719123010 | Clonidine HCl | Clonidine HCl Tab 0.1 MG |
| 00719123012 | Clonidine HCl | Clonidine HCl Tab 0.1 MG |
| 00719123013 | Clonidine HCl | Clonidine HCl Tab 0.1 MG |
| 00719123110 | Clonidine HCl | Clonidine HCl Tab 0.2 MG |
| 00719123112 | Clonidine HCl | Clonidine HCl Tab 0.2 MG |
| 00719123113 | Clonidine HCl | Clonidine HCl Tab 0.2 MG |
| 00719123210 | Clonidine HCl | Clonidine HCl Tab 0.3 MG |
| 00536352605 | Clonidine HCl | Clonidine HCl Tab 0.3 MG |
| 00536562101 | Clonidine HCl | Clonidine HCl Tab 0.1 MG |
| 00536562105 | Clonidine HCl | Clonidine HCl Tab 0.1 MG |
| 00536562201 | Clonidine HCl | Clonidine HCl Tab 0.2 MG |
| 00536562205 | Clonidine HCl | Clonidine HCl Tab 0.2 MG |
| 00536562301 | Clonidine HCl | Clonidine HCl Tab 0.3 MG |
| 00536497301 | Clonidine HCl | Clonidine HCl Tab 0.1 MG |
| 00536497305 | Clonidine HCl | Clonidine HCl Tab 0.1 MG |
| 00536497310 | Clonidine HCl | Clonidine HCl Tab 0.1 MG |
| 00536497401 | Clonidine HCl | Clonidine HCl Tab 0.2 MG |
| 00536497405 | Clonidine HCl | Clonidine HCl Tab 0.2 MG |
| 00536497410 | Clonidine HCl | Clonidine HCl Tab 0.2 MG |
| 00536497501 | Clonidine HCl | Clonidine HCl Tab 0.3 MG |
| 00536497505 | Clonidine HCl | Clonidine HCl Tab 0.3 MG |
| 00555039702 | Clonidine HCl | Clonidine HCl Tab 0.1 MG |
| 00555039705 | Clonidine HCl | Clonidine HCl Tab 0.1 MG |
| 00555039802 | Clonidine HCl | Clonidine HCl Tab 0.2 MG |
| 00555039805 | Clonidine HCl | Clonidine HCl Tab 0.2 MG |
| 00555039902 | Clonidine HCl | Clonidine HCl Tab 0.3 MG |
| 00591560901 | Clonidine HCl | Clonidine HCl Tab 0.1 MG |
| 00591560904 | Clonidine HCl | Clonidine HCl Tab 0.1 MG |
| 00591561201 | Clonidine HCl | Clonidine HCl Tab 0.2 MG |
| 00591561204 | Clonidine HCl | Clonidine HCl Tab 0.2 MG |
| 00591561301 | Clonidine HCl | Clonidine HCl Tab 0.3 MG |
| 00591561304 | Clonidine HCl | Clonidine HCl Tab 0.3 MG |
| 00597000610 | Clonidine HCl | Clonidine HCl Tab 0.1 MG |
| 00597000661 | Clonidine HCl | Clonidine HCl Tab 0.1 MG |
| 00597000710 | Clonidine HCl | Clonidine HCl Tab 0.2 MG |
| 00597000761 | Clonidine HCl | Clonidine HCl Tab 0.2 MG |
| 00580137001 | Clonidine HCl | Clonidine HCl Tab 0.1 MG |
| 00580137101 | Clonidine HCl | Clonidine HCl Tab 0.2 MG |
| 00580137201 | Clonidine HCl | Clonidine HCl Tab 0.3 MG |
| 00603295421 | Clonidine HCl | Clonidine HCl Tab 0.1 MG |
| 00603295428 | Clonidine HCl | Clonidine HCl Tab 0.1 MG |
| 00603295432 | Clonidine HCl | Clonidine HCl Tab 0.1 MG |
| 00603295521 | Clonidine HCl | Clonidine HCl Tab 0.2 MG |
| 00603295528 | Clonidine HCl | Clonidine HCl Tab 0.2 MG |
| 00603295532 | Clonidine HCl | Clonidine HCl Tab 0.2 MG |
| 00603295621 | Clonidine HCl | Clonidine HCl Tab 0.3 MG |
| 00603295628 | Clonidine HCl | Clonidine HCl Tab 0.3 MG |
| 00364082001 | Clonidine HCl | Clonidine HCl Tab 0.1 MG |
| 00364082002 | Clonidine HCl | Clonidine HCl Tab 0.1 MG |
| 00364082090 | Clonidine HCl | Clonidine HCl Tab 0.1 MG |
| 00364082101 | Clonidine HCl | Clonidine HCl Tab 0.2 MG |
| 00364082102 | Clonidine HCl | Clonidine HCl Tab 0.2 MG |
| 00364082190 | Clonidine HCl | Clonidine HCl Tab 0.2 MG |
| 00364082401 | Clonidine HCl | Clonidine HCl Tab 0.3 MG |
| 00364082402 | Clonidine HCl | Clonidine HCl Tab 0.3 MG |
| 00364082490 | Clonidine HCl | Clonidine HCl Tab 0.3 MG |
| 00349892101 | Clonidine HCl | Clonidine HCl Tab 0.1 MG |
| 00349892105 | Clonidine HCl | Clonidine HCl Tab 0.1 MG |
| 00349892201 | Clonidine HCl | Clonidine HCl Tab 0.2 MG |
| 00349892205 | Clonidine HCl | Clonidine HCl Tab 0.2 MG |
| 00349892301 | Clonidine HCl | Clonidine HCl Tab 0.3 MG |
| 00367212710 | Clonidine HCl | Clonidine HCl Tab 0.1 MG |
| 00367212810 | Clonidine HCl | Clonidine HCl Tab 0.2 MG |
| 00367212850 | Clonidine HCl | Clonidine HCl Tab 0.2 MG |
| 00367212910 | Clonidine HCl | Clonidine HCl Tab 0.3 MG |
| 00367212950 | Clonidine HCl | Clonidine HCl Tab 0.3 MG |
| 00405424101 | Clonidine HCl | Clonidine HCl Tab 0.1 MG |
| 00405424102 | Clonidine HCl | Clonidine HCl Tab 0.1 MG |
| 00405424103 | Clonidine HCl | Clonidine HCl Tab 0.1 MG |
| 00405424201 | Clonidine HCl | Clonidine HCl Tab 0.2 MG |
| 00405424202 | Clonidine HCl | Clonidine HCl Tab 0.2 MG |
| 00405424203 | Clonidine HCl | Clonidine HCl Tab 0.2 MG |
| 00405424301 | Clonidine HCl | Clonidine HCl Tab 0.3 MG |
| 00536352301 | Clonidine HCl | Clonidine HCl Tab 0.1 MG |
| 00536352305 | Clonidine HCl | Clonidine HCl Tab 0.1 MG |
| 00536352310 | Clonidine HCl | Clonidine HCl Tab 0.1 MG |
| 00536352401 | Clonidine HCl | Clonidine HCl Tab 0.2 MG |
| 00536352405 | Clonidine HCl | Clonidine HCl Tab 0.2 MG |
| 00536352410 | Clonidine HCl | Clonidine HCl Tab 0.2 MG |
| 00536352601 | Clonidine HCl | Clonidine HCl Tab 0.3 MG |
| 00182827389 | Clonidine HCl | Clonidine HCl Tab 0.2 MG |
| 00223066001 | Clonidine HCl | Clonidine HCl Tab 0.1 MG |
| 00223066002 | Clonidine HCl | Clonidine HCl Tab 0.1 MG |
| 00223066005 | Clonidine HCl | Clonidine HCl Tab 0.1 MG |
| 00223066101 | Clonidine HCl | Clonidine HCl Tab 0.2 MG |
| 00223066102 | Clonidine HCl | Clonidine HCl Tab 0.2 MG |
| 00223066105 | Clonidine HCl | Clonidine HCl Tab 0.2 MG |
| 00223066201 | Clonidine HCl | Clonidine HCl Tab 0.3 MG |
| 00223066202 | Clonidine HCl | Clonidine HCl Tab 0.3 MG |
| 00223066205 | Clonidine HCl | Clonidine HCl Tab 0.3 MG |
| 00228212796 | Clonidine HCl | Clonidine HCl Tab 0.1 MG |
| 00228212896 | Clonidine HCl | Clonidine HCl Tab 0.2 MG |
| 00228212950 | Clonidine HCl | Clonidine HCl Tab 0.3 MG |
| 00228212996 | Clonidine HCl | Clonidine HCl Tab 0.3 MG |
| 00302110001 | Clonidine HCl | Clonidine HCl Tab 0.1 MG |
| 00302110010 | Clonidine HCl | Clonidine HCl Tab 0.1 MG |
| 00302110101 | Clonidine HCl | Clonidine HCl Tab 0.2 MG |
| 00302110110 | Clonidine HCl | Clonidine HCl Tab 0.2 MG |
| 00302110201 | Clonidine HCl | Clonidine HCl Tab 0.3 MG |
| 00304168500 | Clonidine HCl | Clonidine HCl Tab 0.1 MG |
| 00304168501 | Clonidine HCl | Clonidine HCl Tab 0.1 MG |
| 00304168511 | Clonidine HCl | Clonidine HCl Tab 0.1 MG |
| 00304168600 | Clonidine HCl | Clonidine HCl Tab 0.2 MG |
| 00304168601 | Clonidine HCl | Clonidine HCl Tab 0.2 MG |
| 00304168611 | Clonidine HCl | Clonidine HCl Tab 0.2 MG |
| 00304168700 | Clonidine HCl | Clonidine HCl Tab 0.3 MG |
| 00304168701 | Clonidine HCl | Clonidine HCl Tab 0.3 MG |
| 00339544512 | Clonidine HCl | Clonidine HCl Tab 0.1 MG |
| 00339544712 | Clonidine HCl | Clonidine HCl Tab 0.2 MG |
| 00332220009 | Clonidine HCl | Clonidine HCl Tab 0.1 MG |
| 00332220015 | Clonidine HCl | Clonidine HCl Tab 0.1 MG |
| 00332220109 | Clonidine HCl | Clonidine HCl Tab 0.2 MG |
| 00332220115 | Clonidine HCl | Clonidine HCl Tab 0.2 MG |
| 00332220209 | Clonidine HCl | Clonidine HCl Tab 0.3 MG |
| 00304212701 | Clonidine HCl | Clonidine HCl Tab 0.1 MG |
| 00304212801 | Clonidine HCl | Clonidine HCl Tab 0.2 MG |
| 00304212901 | Clonidine HCl | Clonidine HCl Tab 0.3 MG |
| 00304568500 | Clonidine HCl | Clonidine HCl Tab 0.1 MG |
| 00304568501 | Clonidine HCl | Clonidine HCl Tab 0.1 MG |
| 00304568600 | Clonidine HCl | Clonidine HCl Tab 0.2 MG |
| 00304568601 | Clonidine HCl | Clonidine HCl Tab 0.2 MG |
| 00304568701 | Clonidine HCl | Clonidine HCl Tab 0.3 MG |
| 00349852501 | Clonidine HCl | Clonidine HCl Tab 0.1 MG |
| 00349852510 | Clonidine HCl | Clonidine HCl Tab 0.1 MG |
| 00349852600 | Clonidine HCl | Clonidine HCl Tab 0.2 MG |
| 00349852601 | Clonidine HCl | Clonidine HCl Tab 0.2 MG |
| 00349852610 | Clonidine HCl | Clonidine HCl Tab 0.2 MG |
| 00349852701 | Clonidine HCl | Clonidine HCl Tab 0.3 MG |
| 00102341501 | Clonidine HCl | Clonidine HCl Tab 0.1 MG |
| 00102342001 | Clonidine HCl | Clonidine HCl Tab 0.2 MG |
| 00102342501 | Clonidine HCl | Clonidine HCl Tab 0.3 MG |
| 00143118625 | Clonidine HCl | Clonidine HCl Tab 0.1 MG |
| 00143118725 | Clonidine HCl | Clonidine HCl Tab 0.2 MG |
| 00157022701 | Clonidine HCl | Clonidine HCl Tab 0.1 MG |
| 00157022705 | Clonidine HCl | Clonidine HCl Tab 0.1 MG |
| 00157022710 | Clonidine HCl | Clonidine HCl Tab 0.1 MG |
| 00157022801 | Clonidine HCl | Clonidine HCl Tab 0.2 MG |
| 00157022805 | Clonidine HCl | Clonidine HCl Tab 0.2 MG |
| 00157022810 | Clonidine HCl | Clonidine HCl Tab 0.2 MG |
| 00157022901 | Clonidine HCl | Clonidine HCl Tab 0.3 MG |
| 00157022905 | Clonidine HCl | Clonidine HCl Tab 0.3 MG |
| 00182113301 | Clonidine HCl | Clonidine HCl Tab 0.1 MG |
| 00182113305 | Clonidine HCl | Clonidine HCl Tab 0.1 MG |
| 00182113310 | Clonidine HCl | Clonidine HCl Tab 0.1 MG |
| 00182113326 | Clonidine HCl | Clonidine HCl Tab 0.1 MG |
| 00182113401 | Clonidine HCl | Clonidine HCl Tab 0.2 MG |
| 00182113405 | Clonidine HCl | Clonidine HCl Tab 0.2 MG |
| 00182113410 | Clonidine HCl | Clonidine HCl Tab 0.2 MG |
| 00182113426 | Clonidine HCl | Clonidine HCl Tab 0.2 MG |
| 00182113501 | Clonidine HCl | Clonidine HCl Tab 0.3 MG |
| 00182125001 | Clonidine HCl | Clonidine HCl Tab 0.1 MG |
| 00182125005 | Clonidine HCl | Clonidine HCl Tab 0.1 MG |
| 00182125089 | Clonidine HCl | Clonidine HCl Tab 0.1 MG |
| 00182125101 | Clonidine HCl | Clonidine HCl Tab 0.2 MG |
| 00182125105 | Clonidine HCl | Clonidine HCl Tab 0.2 MG |
| 00182125189 | Clonidine HCl | Clonidine HCl Tab 0.2 MG |
| 00182125201 | Clonidine HCl | Clonidine HCl Tab 0.3 MG |
| 00182125289 | Clonidine HCl | Clonidine HCl Tab 0.3 MG |
| 00182176701 | Clonidine HCl | Clonidine HCl Tab 0.1 MG |
| 00182176705 | Clonidine HCl | Clonidine HCl Tab 0.1 MG |
| 00182176710 | Clonidine HCl | Clonidine HCl Tab 0.1 MG |
| 00182176726 | Clonidine HCl | Clonidine HCl Tab 0.1 MG |
| 00182176729 | Clonidine HCl | Clonidine HCl Tab 0.1 MG |
| 00182176789 | Clonidine HCl | Clonidine HCl Tab 0.1 MG |
| 00182176801 | Clonidine HCl | Clonidine HCl Tab 0.2 MG |
| 00182176805 | Clonidine HCl | Clonidine HCl Tab 0.2 MG |
| 00182176810 | Clonidine HCl | Clonidine HCl Tab 0.2 MG |
| 00182176826 | Clonidine HCl | Clonidine HCl Tab 0.2 MG |
| 00182176829 | Clonidine HCl | Clonidine HCl Tab 0.2 MG |
| 00182176889 | Clonidine HCl | Clonidine HCl Tab 0.2 MG |
| 00182176901 | Clonidine HCl | Clonidine HCl Tab 0.3 MG |
| 00182176989 | Clonidine HCl | Clonidine HCl Tab 0.3 MG |
| 00182118489 | Clonidine HCl | Clonidine HCl Tab 0.1 MG |
| 00182118589 | Clonidine HCl | Clonidine HCl Tab 0.2 MG |
| 00182827289 | Clonidine HCl | Clonidine HCl Tab 0.1 MG |
| 67253026411 | Clonidine HCl | Clonidine HCl Tab 0.2 MG |
| 67253026510 | Clonidine HCl | Clonidine HCl Tab 0.3 MG |
| 66336078612 | Clonidine HCl | Clonidine HCl Tab 0.1 MG |
| 66336078630 | Clonidine HCl | Clonidine HCl Tab 0.1 MG |
| 66336078660 | Clonidine HCl | Clonidine HCl Tab 0.1 MG |
| 66336078690 | Clonidine HCl | Clonidine HCl Tab 0.1 MG |
| 66336078730 | Clonidine HCl | Clonidine HCl Tab 0.2 MG |
| 66336078760 | Clonidine HCl | Clonidine HCl Tab 0.2 MG |
| 66336078790 | Clonidine HCl | Clonidine HCl Tab 0.2 MG |
| 66336091960 | Clonidine HCl | Clonidine HCl Tab 0.3 MG |
| 66336091990 | Clonidine HCl | Clonidine HCl Tab 0.3 MG |
| 68115008700 | Clonidine HCl | Clonidine HCl Tab 0.1 MG |
| 68115008701 | Clonidine HCl | Clonidine HCl Tab 0.1 MG |
| 68115008800 | Clonidine HCl | Clonidine HCl Tab 0.2 MG |
| 68258106401 | Clonidine HCl | Clonidine HCl Tab 0.2 MG |
| 68258107601 | Clonidine HCl | Clonidine HCl Tab 0.1 MG |
| 55887047501 | Clonidine HCl | Clonidine HCl Tab 0.2 MG |
| 67544077630 | Clonidine HCl | Clonidine HCl Tab 0.3 MG |
| 21695056704 | Clonidine HCl | Clonidine HCl TD Patch Weekly 0.1 MG/24HR |
| 00003052050 | Clonidine HCl | Clonidine HCl Tab 0.2 MG |
| 00003053450 | Clonidine HCl | Clonidine HCl Tab 0.3 MG |
| 00003028950 | Clonidine HCl | Clonidine HCl Tab 0.1 MG |
| 00047044324 | Clonidine HCl | Clonidine HCl Tab 0.1 MG |
| 00047044330 | Clonidine HCl | Clonidine HCl Tab 0.1 MG |
| 00047044424 | Clonidine HCl | Clonidine HCl Tab 0.2 MG |
| 00047044430 | Clonidine HCl | Clonidine HCl Tab 0.2 MG |
| 00047044524 | Clonidine HCl | Clonidine HCl Tab 0.3 MG |
| 00093049401 | Clonidine HCl | Clonidine HCl Tab 0.1 MG |
| 00093049410 | Clonidine HCl | Clonidine HCl Tab 0.1 MG |
| 00093049501 | Clonidine HCl | Clonidine HCl Tab 0.2 MG |
| 00093049510 | Clonidine HCl | Clonidine HCl Tab 0.2 MG |
| 00093049601 | Clonidine HCl | Clonidine HCl Tab 0.3 MG |
| 23490535203 | Clonidine HCl | Clonidine HCl Tab 0.2 MG |
| 23490535301 | Clonidine HCl | Clonidine HCl Tab 0.3 MG |
| 21695037130 | Clonidine HCl | Clonidine HCl Tab 0.1 MG |
| 49999012700 | Clonidine HCl | Clonidine HCl Tab 0.1 MG |
| 49999025800 | Clonidine HCl | Clonidine HCl Tab 0.2 MG |
| 49999025830 | Clonidine HCl | Clonidine HCl Tab 0.2 MG |
| 49999043830 | Clonidine HCl | Clonidine HCl Tab 0.3 MG |
| 51079029962 | Clonidine HCl | Clonidine HCl Tab 0.1 MG |
| 51079029963 | Clonidine HCl | Clonidine HCl Tab 0.1 MG |
| 51079030062 | Clonidine HCl | Clonidine HCl Tab 0.2 MG |
| 51079030063 | Clonidine HCl | Clonidine HCl Tab 0.2 MG |
| 51079030162 | Clonidine HCl | Clonidine HCl Tab 0.3 MG |
| 51079030163 | Clonidine HCl | Clonidine HCl Tab 0.3 MG |
| 52959078830 | Clonidine HCl | Clonidine HCl Tab 0.2 MG |
| 54868053300 | Clonidine HCl | Clonidine HCl TD Patch Weekly 0.3 MG/24HR |
| 54868004809 | Clonidine HCl | Clonidine HCl Tab 0.1 MG |
| 54738090701 | Clonidine HCl | Clonidine HCl Tab 0.1 MG |
| 54738090703 | Clonidine HCl | Clonidine HCl Tab 0.1 MG |
| 54738090801 | Clonidine HCl | Clonidine HCl Tab 0.2 MG |
| 54738090803 | Clonidine HCl | Clonidine HCl Tab 0.2 MG |
| 54738090901 | Clonidine HCl | Clonidine HCl Tab 0.3 MG |
| 55045116706 | Clonidine HCl | Clonidine HCl Tab 0.1 MG |
| 58016051799 | Clonidine HCl | Clonidine HCl Tab 0.1 MG |
| 58864063328 | Clonidine HCl | Clonidine HCl Tab 0.1 MG |
| 63739006010 | Clonidine HCl | Clonidine HCl Tab 0.1 MG |
| 63739006015 | Clonidine HCl | Clonidine HCl Tab 0.1 MG |
| 63739006110 | Clonidine HCl | Clonidine HCl Tab 0.2 MG |
| 63739006115 | Clonidine HCl | Clonidine HCl Tab 0.2 MG |
| 63739006210 | Clonidine HCl | Clonidine HCl Tab 0.3 MG |
| 63739006215 | Clonidine HCl | Clonidine HCl Tab 0.3 MG |
| 64181003300 | Clonidine HCl | Clonidine HCl Powder |
| 62584033911 | Clonidine HCl | Clonidine HCl Tab 0.2 MG |
| 62584065711 | Clonidine HCl | Clonidine HCl Tab 0.1 MG |
| 62584065911 | Clonidine HCl | Clonidine HCl Tab 0.3 MG |
| 67253026310 | Clonidine HCl | Clonidine HCl Tab 0.1 MG |
| 67253026311 | Clonidine HCl | Clonidine HCl Tab 0.1 MG |
| 67253026410 | Clonidine HCl | Clonidine HCl Tab 0.2 MG |
| 63874047515 | Clonidine HCl | Clonidine HCl Tab 0.1 MG |
| 63874047520 | Clonidine HCl | Clonidine HCl Tab 0.1 MG |
| 63874047528 | Clonidine HCl | Clonidine HCl Tab 0.1 MG |
| 63874047530 | Clonidine HCl | Clonidine HCl Tab 0.1 MG |
| 63874047560 | Clonidine HCl | Clonidine HCl Tab 0.1 MG |
| 63874047581 | Clonidine HCl | Clonidine HCl Tab 0.1 MG |
| 63874101403 | Clonidine HCl | Clonidine HCl Tab 0.3 MG |
| 63874101406 | Clonidine HCl | Clonidine HCl Tab 0.3 MG |
| 68115008712 | Clonidine HCl | Clonidine HCl Tab 0.1 MG |
| 68115008730 | Clonidine HCl | Clonidine HCl Tab 0.1 MG |
| 68115008760 | Clonidine HCl | Clonidine HCl Tab 0.1 MG |
| 68115008790 | Clonidine HCl | Clonidine HCl Tab 0.1 MG |
| 68115008830 | Clonidine HCl | Clonidine HCl Tab 0.2 MG |
| 68115008860 | Clonidine HCl | Clonidine HCl Tab 0.2 MG |
| 68115008890 | Clonidine HCl | Clonidine HCl Tab 0.2 MG |
| 68115008930 | Clonidine HCl | Clonidine HCl Tab 0.3 MG |
| 00597003134 | Clonidine HCl | Clonidine HCl TD Patch Weekly 0.1 MG/24HR |
| 00603295721 | Clonidine HCl | Clonidine HCl Tab 0.1 MG |
| 00603295728 | Clonidine HCl | Clonidine HCl Tab 0.1 MG |
| 00603295732 | Clonidine HCl | Clonidine HCl Tab 0.1 MG |
| 00603295821 | Clonidine HCl | Clonidine HCl Tab 0.2 MG |
| 00603295828 | Clonidine HCl | Clonidine HCl Tab 0.2 MG |
| 00603295832 | Clonidine HCl | Clonidine HCl Tab 0.2 MG |
| 00603295921 | Clonidine HCl | Clonidine HCl Tab 0.3 MG |
| 00603295928 | Clonidine HCl | Clonidine HCl Tab 0.3 MG |
| 12280030130 | Clonidine HCl | Clonidine HCl Tab 0.3 MG |
| 12280030160 | Clonidine HCl | Clonidine HCl Tab 0.3 MG |
| 16590026630 | Clonidine HCl | Clonidine HCl Tab 0.1 MG |
| 16590026660 | Clonidine HCl | Clonidine HCl Tab 0.1 MG |
| 16590026690 | Clonidine HCl | Clonidine HCl Tab 0.1 MG |
| 23490535101 | Clonidine HCl | Clonidine HCl Tab 0.1 MG |
| 23490535102 | Clonidine HCl | Clonidine HCl Tab 0.1 MG |
| 23490535103 | Clonidine HCl | Clonidine HCl Tab 0.1 MG |
| 23490535104 | Clonidine HCl | Clonidine HCl Tab 0.1 MG |
| 23490535201 | Clonidine HCl | Clonidine HCl Tab 0.2 MG |
| 23490535202 | Clonidine HCl | Clonidine HCl Tab 0.2 MG |
| 62584033901 | Clonidine HCl | Clonidine HCl Tab 0.2 MG |
| 62584065701 | Clonidine HCl | Clonidine HCl Tab 0.1 MG |
| 62584065901 | Clonidine HCl | Clonidine HCl Tab 0.3 MG |
| 62991142201 | Clonidine HCl | Clonidine HCl Powder |
| 62991142202 | Clonidine HCl | Clonidine HCl Powder |
| 63275997401 | Clonidine HCl | Clonidine HCl Powder |
| 63275997402 | Clonidine HCl | Clonidine HCl Powder |
| 63275997403 | Clonidine HCl | Clonidine HCl Powder |
| 63370005210 | Clonidine HCl | Clonidine HCl Powder |
| 63370005215 | Clonidine HCl | Clonidine HCl Powder |
| 63370005225 | Clonidine HCl | Clonidine HCl Powder |
| 63739006001 | Clonidine HCl | Clonidine HCl Tab 0.1 MG |
| 63739006002 | Clonidine HCl | Clonidine HCl Tab 0.1 MG |
| 63739006003 | Clonidine HCl | Clonidine HCl Tab 0.1 MG |
| 63739006101 | Clonidine HCl | Clonidine HCl Tab 0.2 MG |
| 63739006102 | Clonidine HCl | Clonidine HCl Tab 0.2 MG |
| 63739006103 | Clonidine HCl | Clonidine HCl Tab 0.2 MG |
| 63874047402 | Clonidine HCl | Clonidine HCl Tab 0.2 MG |
| 63874047410 | Clonidine HCl | Clonidine HCl Tab 0.2 MG |
| 63874047412 | Clonidine HCl | Clonidine HCl Tab 0.2 MG |
| 63874047414 | Clonidine HCl | Clonidine HCl Tab 0.2 MG |
| 63874047415 | Clonidine HCl | Clonidine HCl Tab 0.2 MG |
| 63874047420 | Clonidine HCl | Clonidine HCl Tab 0.2 MG |
| 63874047421 | Clonidine HCl | Clonidine HCl Tab 0.2 MG |
| 63874047424 | Clonidine HCl | Clonidine HCl Tab 0.2 MG |
| 63874047428 | Clonidine HCl | Clonidine HCl Tab 0.2 MG |
| 63874047430 | Clonidine HCl | Clonidine HCl Tab 0.2 MG |
| 63874047440 | Clonidine HCl | Clonidine HCl Tab 0.2 MG |
| 63874047460 | Clonidine HCl | Clonidine HCl Tab 0.2 MG |
| 63874047481 | Clonidine HCl | Clonidine HCl Tab 0.2 MG |
| 63874047501 | Clonidine HCl | Clonidine HCl Tab 0.1 MG |
| 63874047502 | Clonidine HCl | Clonidine HCl Tab 0.1 MG |
| 63874047510 | Clonidine HCl | Clonidine HCl Tab 0.1 MG |
| 63874047512 | Clonidine HCl | Clonidine HCl Tab 0.1 MG |
| 57866352601 | Clonidine HCl | Clonidine HCl Tab 0.3 MG |
| 57866022301 | Clonidine HCl | Clonidine HCl Tab 0.1 MG |
| 57866981102 | Clonidine HCl | Clonidine HCl Tab 0.1 MG |
| 58016051700 | Clonidine HCl | Clonidine HCl Tab 0.1 MG |
| 58016051702 | Clonidine HCl | Clonidine HCl Tab 0.1 MG |
| 58016051708 | Clonidine HCl | Clonidine HCl Tab 0.1 MG |
| 58016051712 | Clonidine HCl | Clonidine HCl Tab 0.1 MG |
| 58016051730 | Clonidine HCl | Clonidine HCl Tab 0.1 MG |
| 58016051760 | Clonidine HCl | Clonidine HCl Tab 0.1 MG |
| 58016051790 | Clonidine HCl | Clonidine HCl Tab 0.1 MG |
| 58016051815 | Clonidine HCl | Clonidine HCl Tab 0.2 MG |
| 58864011028 | Clonidine HCl | Clonidine HCl Tab 0.1 MG |
| 58864011030 | Clonidine HCl | Clonidine HCl Tab 0.1 MG |
| 58864011060 | Clonidine HCl | Clonidine HCl Tab 0.1 MG |
| 58864011090 | Clonidine HCl | Clonidine HCl Tab 0.1 MG |
| 58864011130 | Clonidine HCl | Clonidine HCl Tab 0.2 MG |
| 58864011160 | Clonidine HCl | Clonidine HCl Tab 0.2 MG |
| 58864011190 | Clonidine HCl | Clonidine HCl Tab 0.2 MG |
| 60346078612 | Clonidine HCl | Clonidine HCl Tab 0.1 MG |
| 60346078625 | Clonidine HCl | Clonidine HCl Tab 0.1 MG |
| 60346078630 | Clonidine HCl | Clonidine HCl Tab 0.1 MG |
| 60346078660 | Clonidine HCl | Clonidine HCl Tab 0.1 MG |
| 60346078662 | Clonidine HCl | Clonidine HCl Tab 0.1 MG |
| 60346078690 | Clonidine HCl | Clonidine HCl Tab 0.1 MG |
| 60346078725 | Clonidine HCl | Clonidine HCl Tab 0.2 MG |
| 60346078730 | Clonidine HCl | Clonidine HCl Tab 0.2 MG |
| 60346078759 | Clonidine HCl | Clonidine HCl Tab 0.2 MG |
| 60346078760 | Clonidine HCl | Clonidine HCl Tab 0.2 MG |
| 60346078762 | Clonidine HCl | Clonidine HCl Tab 0.2 MG |
| 60346078790 | Clonidine HCl | Clonidine HCl Tab 0.2 MG |
| 60346078794 | Clonidine HCl | Clonidine HCl Tab 0.2 MG |
| 60346091990 | Clonidine HCl | Clonidine HCl Tab 0.3 MG |
| 55045165601 | Clonidine HCl | Clonidine HCl Tab 0.2 MG |
| 55045177001 | Clonidine HCl | Clonidine HCl Tab 0.1 MG |
| 55289007301 | Clonidine HCl | Clonidine HCl Tab 0.1 MG |
| 55289007308 | Clonidine HCl | Clonidine HCl Tab 0.1 MG |
| 55289007330 | Clonidine HCl | Clonidine HCl Tab 0.1 MG |
| 55289007360 | Clonidine HCl | Clonidine HCl Tab 0.1 MG |
| 55289007390 | Clonidine HCl | Clonidine HCl Tab 0.1 MG |
| 55289007397 | Clonidine HCl | Clonidine HCl Tab 0.1 MG |
| 55289007430 | Clonidine HCl | Clonidine HCl Tab 0.2 MG |
| 55289007460 | Clonidine HCl | Clonidine HCl Tab 0.2 MG |
| 55887047530 | Clonidine HCl | Clonidine HCl Tab 0.2 MG |
| 55887047560 | Clonidine HCl | Clonidine HCl Tab 0.2 MG |
| 55887047590 | Clonidine HCl | Clonidine HCl Tab 0.2 MG |
| 55887026930 | Clonidine HCl | Clonidine HCl Tab 0.3 MG |
| 55887026960 | Clonidine HCl | Clonidine HCl Tab 0.3 MG |
| 55887026982 | Clonidine HCl | Clonidine HCl Tab 0.3 MG |
| 55887026990 | Clonidine HCl | Clonidine HCl Tab 0.3 MG |
| 55887073830 | Clonidine HCl | Clonidine HCl Tab 0.1 MG |
| 55887073860 | Clonidine HCl | Clonidine HCl Tab 0.1 MG |
| 55887073890 | Clonidine HCl | Clonidine HCl Tab 0.1 MG |
| 55887048804 | Clonidine HCl | Clonidine HCl TD Patch Weekly 0.3 MG/24HR |
| 57866352301 | Clonidine HCl | Clonidine HCl Tab 0.1 MG |
| 57866352303 | Clonidine HCl | Clonidine HCl Tab 0.1 MG |
| 57866352401 | Clonidine HCl | Clonidine HCl Tab 0.2 MG |
| 54569047804 | Clonidine HCl | Clonidine HCl Tab 0.1 MG |
| 54569143700 | Clonidine HCl | Clonidine HCl TD Patch Weekly 0.1 MG/24HR |
| 54569280100 | Clonidine HCl | Clonidine HCl Tab 0.3 MG |
| 54868004800 | Clonidine HCl | Clonidine HCl Tab 0.1 MG |
| 54868004801 | Clonidine HCl | Clonidine HCl Tab 0.1 MG |
| 54868004802 | Clonidine HCl | Clonidine HCl Tab 0.1 MG |
| 54868004803 | Clonidine HCl | Clonidine HCl Tab 0.1 MG |
| 54868004805 | Clonidine HCl | Clonidine HCl Tab 0.1 MG |
| 54868004806 | Clonidine HCl | Clonidine HCl Tab 0.1 MG |
| 54868004807 | Clonidine HCl | Clonidine HCl Tab 0.1 MG |
| 54868004808 | Clonidine HCl | Clonidine HCl Tab 0.1 MG |
| 54868004900 | Clonidine HCl | Clonidine HCl Tab 0.2 MG |
| 54868004901 | Clonidine HCl | Clonidine HCl Tab 0.2 MG |
| 54868004902 | Clonidine HCl | Clonidine HCl Tab 0.2 MG |
| 54868004903 | Clonidine HCl | Clonidine HCl Tab 0.2 MG |
| 54868004905 | Clonidine HCl | Clonidine HCl Tab 0.2 MG |
| 54868053201 | Clonidine HCl | Clonidine HCl TD Patch Weekly 0.2 MG/24HR |
| 54868053500 | Clonidine HCl | Clonidine HCl Tab 0.1 MG |
| 54868053701 | Clonidine HCl | Clonidine HCl TD Patch Weekly 0.1 MG/24HR |
| 54868093100 | Clonidine HCl | Clonidine HCl Tab 0.2 MG |
| 54868196700 | Clonidine HCl | Clonidine HCl Tab 0.3 MG |
| 54868196702 | Clonidine HCl | Clonidine HCl Tab 0.3 MG |
| 54868196703 | Clonidine HCl | Clonidine HCl Tab 0.3 MG |
| 49999012704 | Clonidine HCl | Clonidine HCl Tab 0.1 MG |
| 49999012712 | Clonidine HCl | Clonidine HCl Tab 0.1 MG |
| 49999012720 | Clonidine HCl | Clonidine HCl Tab 0.1 MG |
| 49999012730 | Clonidine HCl | Clonidine HCl Tab 0.1 MG |
| 49999043800 | Clonidine HCl | Clonidine HCl Tab 0.3 MG |
| 51079029901 | Clonidine HCl | Clonidine HCl Tab 0.1 MG |
| 51079029917 | Clonidine HCl | Clonidine HCl Tab 0.1 MG |
| 51079029919 | Clonidine HCl | Clonidine HCl Tab 0.1 MG |
| 51079029920 | Clonidine HCl | Clonidine HCl Tab 0.1 MG |
| 51079030001 | Clonidine HCl | Clonidine HCl Tab 0.2 MG |
| 51079030017 | Clonidine HCl | Clonidine HCl Tab 0.2 MG |
| 51079030019 | Clonidine HCl | Clonidine HCl Tab 0.2 MG |
| 51079030020 | Clonidine HCl | Clonidine HCl Tab 0.2 MG |
| 51079030101 | Clonidine HCl | Clonidine HCl Tab 0.3 MG |
| 51079030120 | Clonidine HCl | Clonidine HCl Tab 0.3 MG |
| 51552048001 | Clonidine HCl | Clonidine HCl Powder |
| 51552048002 | Clonidine HCl | Clonidine HCl Powder |
| 51552048004 | Clonidine HCl | Clonidine HCl Powder |
| 51552048005 | Clonidine HCl | Clonidine HCl Powder |
| 51655028824 | Clonidine HCl | Clonidine HCl Tab 0.1 MG |
| 51655035353 | Clonidine HCl | Clonidine HCl Tab 0.1 MG |
| 51655036225 | Clonidine HCl | Clonidine HCl Tab 0.2 MG |
| 51927237900 | Clonidine HCl | Clonidine HCl Powder |
| 52959071800 | Clonidine HCl | Clonidine HCl Tab 0.1 MG |
| 52959071805 | Clonidine HCl | Clonidine HCl Tab 0.1 MG |
| 52959071812 | Clonidine HCl | Clonidine HCl Tab 0.1 MG |
| 52959071830 | Clonidine HCl | Clonidine HCl Tab 0.1 MG |
| 52959071840 | Clonidine HCl | Clonidine HCl Tab 0.1 MG |
| 52959071860 | Clonidine HCl | Clonidine HCl Tab 0.1 MG |
| 52959071890 | Clonidine HCl | Clonidine HCl Tab 0.1 MG |
| 53489021501 | Clonidine HCl | Clonidine HCl Tab 0.1 MG |
| 53489021510 | Clonidine HCl | Clonidine HCl Tab 0.1 MG |
| 53489021601 | Clonidine HCl | Clonidine HCl Tab 0.2 MG |
| 53489021610 | Clonidine HCl | Clonidine HCl Tab 0.2 MG |
| 53489021701 | Clonidine HCl | Clonidine HCl Tab 0.3 MG |
| 52959067912 | Clonidine HCl | Clonidine HCl TD Patch Weekly 0.2 MG/24HR |
| 54569185300 | Clonidine HCl | Clonidine HCl Tab 0.2 MG |
| 54569185301 | Clonidine HCl | Clonidine HCl Tab 0.2 MG |
| 54569047800 | Clonidine HCl | Clonidine HCl Tab 0.1 MG |
| 54569047801 | Clonidine HCl | Clonidine HCl Tab 0.1 MG |
| 54569047802 | Clonidine HCl | Clonidine HCl Tab 0.1 MG |
| 00615257353 | Clonidine HCl | Clonidine HCl Tab 0.2 MG |
| 00615257363 | Clonidine HCl | Clonidine HCl Tab 0.2 MG |
| 00677192201 | Clonidine HCl | Clonidine HCl Tab 0.1 MG |
| 00677192210 | Clonidine HCl | Clonidine HCl Tab 0.1 MG |
| 00677192301 | Clonidine HCl | Clonidine HCl Tab 0.2 MG |
| 00677192310 | Clonidine HCl | Clonidine HCl Tab 0.2 MG |
| 00677192401 | Clonidine HCl | Clonidine HCl Tab 0.3 MG |
| 00904102661 | Clonidine HCl | Clonidine HCl Tab 0.2 MG |
| 00904565661 | Clonidine HCl | Clonidine HCl Tab 0.1 MG |
| 00904565761 | Clonidine HCl | Clonidine HCl Tab 0.2 MG |
| 00904565861 | Clonidine HCl | Clonidine HCl Tab 0.3 MG |
| 38779056101 | Clonidine HCl | Clonidine HCl Powder |
| 38779056103 | Clonidine HCl | Clonidine HCl Powder |
| 38779056104 | Clonidine HCl | Clonidine HCl Powder |
| 38779056106 | Clonidine HCl | Clonidine HCl Powder |
| 49452214701 | Clonidine HCl | Clonidine HCl Powder |
| 49452214702 | Clonidine HCl | Clonidine HCl Powder |
| 49452214703 | Clonidine HCl | Clonidine HCl Powder |
| 49452214704 | Clonidine HCl | Clonidine HCl Powder |
| 49999025890 | Clonidine HCl | Clonidine HCl Tab 0.2 MG |
| 00228212710 | Clonidine HCl | Clonidine HCl Tab 0.1 MG |
| 00228212750 | Clonidine HCl | Clonidine HCl Tab 0.1 MG |
| 00228212810 | Clonidine HCl | Clonidine HCl Tab 0.2 MG |
| 00228212850 | Clonidine HCl | Clonidine HCl Tab 0.2 MG |
| 00228212910 | Clonidine HCl | Clonidine HCl Tab 0.3 MG |
| 00247110330 | Clonidine HCl | Clonidine HCl Tab 0.1 MG |
| 00247110360 | Clonidine HCl | Clonidine HCl Tab 0.1 MG |
| 00247110430 | Clonidine HCl | Clonidine HCl Tab 0.2 MG |
| 00247110460 | Clonidine HCl | Clonidine HCl Tab 0.2 MG |
| 00378015201 | Clonidine HCl | Clonidine HCl Tab 0.1 MG |
| 00378015210 | Clonidine HCl | Clonidine HCl Tab 0.1 MG |
| 00378018601 | Clonidine HCl | Clonidine HCl Tab 0.2 MG |
| 00378018610 | Clonidine HCl | Clonidine HCl Tab 0.2 MG |
| 00378019901 | Clonidine HCl | Clonidine HCl Tab 0.3 MG |
| 00440732510 | Clonidine HCl | Clonidine HCl Tab 0.1 MG |
| 00440732530 | Clonidine HCl | Clonidine HCl Tab 0.1 MG |
| 00440732560 | Clonidine HCl | Clonidine HCl Tab 0.1 MG |
| 00440732590 | Clonidine HCl | Clonidine HCl Tab 0.1 MG |
| 00440732592 | Clonidine HCl | Clonidine HCl Tab 0.1 MG |
| 00440732594 | Clonidine HCl | Clonidine HCl Tab 0.1 MG |
| 00440732660 | Clonidine HCl | Clonidine HCl Tab 0.2 MG |
| 00440732690 | Clonidine HCl | Clonidine HCl Tab 0.2 MG |
| 00440732692 | Clonidine HCl | Clonidine HCl Tab 0.2 MG |
| 00440732694 | Clonidine HCl | Clonidine HCl Tab 0.2 MG |
| 00597000601 | Clonidine HCl | Clonidine HCl Tab 0.1 MG |
| 00597000701 | Clonidine HCl | Clonidine HCl Tab 0.2 MG |
| 00597001101 | Clonidine HCl | Clonidine HCl Tab 0.3 MG |
| 00597003112 | Clonidine HCl | Clonidine HCl TD Patch Weekly 0.1 MG/24HR |
| 00597003212 | Clonidine HCl | Clonidine HCl TD Patch Weekly 0.2 MG/24HR |
| 00597003334 | Clonidine HCl | Clonidine HCl TD Patch Weekly 0.3 MG/24HR |
| 00615257243 | Clonidine HCl | Clonidine HCl Tab 0.1 MG |
| 00615257253 | Clonidine HCl | Clonidine HCl Tab 0.1 MG |
| 00615257263 | Clonidine HCl | Clonidine HCl Tab 0.1 MG |
| 00005318023 | Clonidine HCl | Clonidine HCl Tab 0.1 MG |
| 00005318034 | Clonidine HCl | Clonidine HCl Tab 0.1 MG |
| 00005318123 | Clonidine HCl | Clonidine HCl Tab 0.2 MG |
| 00005318134 | Clonidine HCl | Clonidine HCl Tab 0.2 MG |
| 00005318223 | Clonidine HCl | Clonidine HCl Tab 0.3 MG |
| 00074692201 | Deserpidine & Hydrochlorothiazide | Deserpidine & Hydrochlorothiazide Tab 0.125-25 MG |
| 00074693101 | Deserpidine & Hydrochlorothiazide | Deserpidine & Hydrochlorothiazide Tab 0.125-50 MG |
| 00074692701 | Deserpidine & Hydrochlorothiazide | Deserpidine & Hydrochlorothiazide Tab 0.25-25 MG |
| 00677095101 | Deserpidine & Methyclothiazide | Deserpidine & Methyclothiazide Tab 0.5-5 MG |
| 00719156610 | Deserpidine & Methyclothiazide | Deserpidine & Methyclothiazide Tab 0.25-5 MG |
| 00719156612 | Deserpidine & Methyclothiazide | Deserpidine & Methyclothiazide Tab 0.25-5 MG |
| 00719156710 | Deserpidine & Methyclothiazide | Deserpidine & Methyclothiazide Tab 0.5-5 MG |
| 00781100201 | Deserpidine & Methyclothiazide | Deserpidine & Methyclothiazide Tab 0.25-5 MG |
| 00781100601 | Deserpidine & Methyclothiazide | Deserpidine & Methyclothiazide Tab 0.5-5 MG |
| 00725015701 | Deserpidine & Methyclothiazide | Deserpidine & Methyclothiazide Tab 0.5-5 MG |
| 00725015705 | Deserpidine & Methyclothiazide | Deserpidine & Methyclothiazide Tab 0.5-5 MG |
| 00725015801 | Deserpidine & Methyclothiazide | Deserpidine & Methyclothiazide Tab 0.25-5 MG |
| 00725015805 | Deserpidine & Methyclothiazide | Deserpidine & Methyclothiazide Tab 0.25-5 MG |
| 00725015810 | Deserpidine & Methyclothiazide | Deserpidine & Methyclothiazide Tab 0.25-5 MG |
| 00814479514 | Deserpidine & Methyclothiazide | Deserpidine & Methyclothiazide Tab 0.25-5 MG |
| 00839679406 | Deserpidine & Methyclothiazide | Deserpidine & Methyclothiazide Tab 0.25-5 MG |
| 00839679506 | Deserpidine & Methyclothiazide | Deserpidine & Methyclothiazide Tab 0.5-5 MG |
| 00904195060 | Deserpidine & Methyclothiazide | Deserpidine & Methyclothiazide Tab 0.25-5 MG |
| 00904195070 | Deserpidine & Methyclothiazide | Deserpidine & Methyclothiazide Tab 0.25-5 MG |
| 00904195160 | Deserpidine & Methyclothiazide | Deserpidine & Methyclothiazide Tab 0.5-5 MG |
| 00904195170 | Deserpidine & Methyclothiazide | Deserpidine & Methyclothiazide Tab 0.5-5 MG |
| 11845023101 | Deserpidine & Methyclothiazide | Deserpidine & Methyclothiazide Tab 0.5-5 MG |
| 11845023103 | Deserpidine & Methyclothiazide | Deserpidine & Methyclothiazide Tab 0.5-5 MG |
| 17236049301 | Deserpidine & Methyclothiazide | Deserpidine & Methyclothiazide Tab 0.25-5 MG |
| 17236065101 | Deserpidine & Methyclothiazide | Deserpidine & Methyclothiazide Tab 0.5-5 MG |
| 47202250101 | Deserpidine & Methyclothiazide | Deserpidine & Methyclothiazide Tab 0.25-5 MG |
| 47202250201 | Deserpidine & Methyclothiazide | Deserpidine & Methyclothiazide Tab 0.5-5 MG |
| 52446029421 | Deserpidine & Methyclothiazide | Deserpidine & Methyclothiazide Tab 0.5-5 MG |
| 52446029521 | Deserpidine & Methyclothiazide | Deserpidine & Methyclothiazide Tab 0.25-5 MG |
| 54274001010 | Deserpidine & Methyclothiazide | Deserpidine & Methyclothiazide Tab 0.25-5 MG |
| 54274002510 | Deserpidine & Methyclothiazide | Deserpidine & Methyclothiazide Tab 0.5-5 MG |
| 54274002530 | Deserpidine & Methyclothiazide | Deserpidine & Methyclothiazide Tab 0.5-5 MG |
| 54569209300 | Deserpidine & Methyclothiazide | Deserpidine & Methyclothiazide Tab 0.25-5 MG |
| 00074683802 | Deserpidine & Methyclothiazide | Deserpidine & Methyclothiazide Tab 0.25-5 MG |
| 00074683806 | Deserpidine & Methyclothiazide | Deserpidine & Methyclothiazide Tab 0.25-5 MG |
| 00074685402 | Deserpidine & Methyclothiazide | Deserpidine & Methyclothiazide Tab 0.5-5 MG |
| 00102317501 | Deserpidine & Methyclothiazide | Deserpidine & Methyclothiazide Tab 0.25-5 MG |
| 00102318001 | Deserpidine & Methyclothiazide | Deserpidine & Methyclothiazide Tab 0.5-5 MG |
| 00182167101 | Deserpidine & Methyclothiazide | Deserpidine & Methyclothiazide Tab 0.25-5 MG |
| 00182167105 | Deserpidine & Methyclothiazide | Deserpidine & Methyclothiazide Tab 0.25-5 MG |
| 00182167201 | Deserpidine & Methyclothiazide | Deserpidine & Methyclothiazide Tab 0.5-5 MG |
| 00302408001 | Deserpidine & Methyclothiazide | Deserpidine & Methyclothiazide Tab 0.25-5 MG |
| 00302408101 | Deserpidine & Methyclothiazide | Deserpidine & Methyclothiazide Tab 0.5-5 MG |
| 00304131001 | Deserpidine & Methyclothiazide | Deserpidine & Methyclothiazide Tab 0.25-5 MG |
| 00304131101 | Deserpidine & Methyclothiazide | Deserpidine & Methyclothiazide Tab 0.5-5 MG |
| 00349804501 | Deserpidine & Methyclothiazide | Deserpidine & Methyclothiazide Tab 0.25-5 MG |
| 00349840401 | Deserpidine & Methyclothiazide | Deserpidine & Methyclothiazide Tab 0.25-5 MG |
| 00349840405 | Deserpidine & Methyclothiazide | Deserpidine & Methyclothiazide Tab 0.25-5 MG |
| 00349840501 | Deserpidine & Methyclothiazide | Deserpidine & Methyclothiazide Tab 0.25-5 MG |
| 00349840505 | Deserpidine & Methyclothiazide | Deserpidine & Methyclothiazide Tab 0.25-5 MG |
| 00349840510 | Deserpidine & Methyclothiazide | Deserpidine & Methyclothiazide Tab 0.25-5 MG |
| 00364085001 | Deserpidine & Methyclothiazide | Deserpidine & Methyclothiazide Tab 0.5-5 MG |
| 00364069801 | Deserpidine & Methyclothiazide | Deserpidine & Methyclothiazide Tab 0.25-5 MG |
| 00536400701 | Deserpidine & Methyclothiazide | Deserpidine & Methyclothiazide Tab 0.25-5 MG |
| 00074683801 | Deserpidine & Methyclothiazide | Deserpidine & Methyclothiazide Tab 0.25-5 MG |
| 00677095001 | Deserpidine & Methyclothiazide | Deserpidine & Methyclothiazide Tab 0.25-5 MG |
| 00074685401 | Deserpidine & Methyclothiazide | Deserpidine & Methyclothiazide Tab 0.5-5 MG |
| 00536400801 | Deserpidine & Methyclothiazide | Deserpidine & Methyclothiazide Tab 0.5-5 MG |
| 00580149501 | Deserpidine & Methyclothiazide | Deserpidine & Methyclothiazide Tab 0.25-5 MG |
| 38779032408 | Diazoxide (Antihypertensive) | Diazoxide Powder |
| 49452254103 | Diazoxide (Antihypertensive) | Diazoxide Powder |
| 49452254105 | Diazoxide (Antihypertensive) | Diazoxide Powder |
| 37803041702 | Diazoxide (Antihypertensive) | Diazoxide Powder |
| 62991285701 | Diazoxide (Antihypertensive) | Diazoxide Powder |
| 62991285702 | Diazoxide (Antihypertensive) | Diazoxide Powder |
| 62991285703 | Diazoxide (Antihypertensive) | Diazoxide Powder |
| 62991285704 | Diazoxide (Antihypertensive) | Diazoxide Powder |
| 38779032411 | Diazoxide (Antihypertensive) | Diazoxide Powder |
| 38779032415 | Diazoxide (Antihypertensive) | Diazoxide Powder |
| 38779032425 | Diazoxide (Antihypertensive) | Diazoxide Powder |
| 51309061610 | Diazoxide (Antihypertensive) | Diazoxide Inj 15 MG/ML |
| 51309061620 | Diazoxide (Antihypertensive) | Diazoxide Inj 15 MG/ML |
| 54569328300 | Diazoxide (Antihypertensive) | Diazoxide Inj 15 MG/ML |
| 00469279040 | Diazoxide (Antihypertensive) | Diazoxide Inj 15 MG/ML |
| 51927274200 | Diazoxide (Antihypertensive) | Diazoxide Powder |
| 00085020105 | Diazoxide (Antihypertensive) | Diazoxide Inj 15 MG/ML |
| 38779032401 | Diazoxide (Antihypertensive) | Diazoxide Powder |
| 38779032403 | Diazoxide (Antihypertensive) | Diazoxide Powder |
| 38779032404 | Diazoxide (Antihypertensive) | Diazoxide Powder |
| 38779032406 | Diazoxide (Antihypertensive) | Diazoxide Powder |
| 49452254101 | Diazoxide (Antihypertensive) | Diazoxide Powder |
| 49452254102 | Diazoxide (Antihypertensive) | Diazoxide Powder |
| 49452254104 | Diazoxide (Antihypertensive) | Diazoxide Powder |
| 43353074060 | Doxazosin Mesylate | Doxazosin Mesylate Tab 4 MG |
| 54868480200 | Doxazosin Mesylate | Doxazosin Mesylate Tab 4 MG |
| 51138002430 | Doxazosin Mesylate | Doxazosin Mesylate Tab 1 MG |
| 51138002530 | Doxazosin Mesylate | Doxazosin Mesylate Tab 2 MG |
| 51138002630 | Doxazosin Mesylate | Doxazosin Mesylate Tab 4 MG |
| 51138002730 | Doxazosin Mesylate | Doxazosin Mesylate Tab 8 MG |
| 60429095301 | Doxazosin Mesylate | Doxazosin Mesylate Tab 1 MG |
| 60429095310 | Doxazosin Mesylate | Doxazosin Mesylate Tab 1 MG |
| 60429095401 | Doxazosin Mesylate | Doxazosin Mesylate Tab 2 MG |
| 60429095410 | Doxazosin Mesylate | Doxazosin Mesylate Tab 2 MG |
| 60429095501 | Doxazosin Mesylate | Doxazosin Mesylate Tab 4 MG |
| 60429095510 | Doxazosin Mesylate | Doxazosin Mesylate Tab 4 MG |
| 60429095601 | Doxazosin Mesylate | Doxazosin Mesylate Tab 8 MG |
| 60429095610 | Doxazosin Mesylate | Doxazosin Mesylate Tab 8 MG |
| 00093812219 | Doxazosin Mesylate | Doxazosin Mesylate Tab 4 MG |
| 00093812293 | Doxazosin Mesylate | Doxazosin Mesylate Tab 4 MG |
| 23490547509 | Doxazosin Mesylate | Doxazosin Mesylate Tab 4 MG |
| 23490547609 | Doxazosin Mesylate | Doxazosin Mesylate Tab 8 MG |
| 60429095390 | Doxazosin Mesylate | Doxazosin Mesylate Tab 1 MG |
| 42254007530 | Doxazosin Mesylate | Doxazosin Mesylate Tab 2 MG |
| 54868480103 | Doxazosin Mesylate | Doxazosin Mesylate Tab 2 MG |
| 60429005690 | Doxazosin Mesylate | Doxazosin Mesylate Tab 8 MG |
| 60429005390 | Doxazosin Mesylate | Doxazosin Mesylate Tab 1 MG |
| 60429005490 | Doxazosin Mesylate | Doxazosin Mesylate Tab 2 MG |
| 60429005590 | Doxazosin Mesylate | Doxazosin Mesylate Tab 4 MG |
| 60429055690 | Doxazosin Mesylate | Doxazosin Mesylate Tab 8 MG |
| 55289002290 | Doxazosin Mesylate | Doxazosin Mesylate Tab 2 MG |
| 55289060090 | Doxazosin Mesylate | Doxazosin Mesylate Tab 4 MG |
| 00093812119 | Doxazosin Mesylate | Doxazosin Mesylate Tab 2 MG |
| 00093812193 | Doxazosin Mesylate | Doxazosin Mesylate Tab 2 MG |
| 54458095310 | Doxazosin Mesylate | Doxazosin Mesylate Tab 4 MG |
| 54458095410 | Doxazosin Mesylate | Doxazosin Mesylate Tab 2 MG |
| 54458095510 | Doxazosin Mesylate | Doxazosin Mesylate Tab 1 MG |
| 63629130901 | Doxazosin Mesylate | Doxazosin Mesylate Tab 8 MG |
| 63629135501 | Doxazosin Mesylate | Doxazosin Mesylate Tab 4 MG |
| 68645017054 | Doxazosin Mesylate | Doxazosin Mesylate Tab 8 MG |
| 60429005301 | Doxazosin Mesylate | Doxazosin Mesylate Tab 1 MG |
| 60429005310 | Doxazosin Mesylate | Doxazosin Mesylate Tab 1 MG |
| 60429005401 | Doxazosin Mesylate | Doxazosin Mesylate Tab 2 MG |
| 60429005410 | Doxazosin Mesylate | Doxazosin Mesylate Tab 2 MG |
| 60429005501 | Doxazosin Mesylate | Doxazosin Mesylate Tab 4 MG |
| 60429005510 | Doxazosin Mesylate | Doxazosin Mesylate Tab 4 MG |
| 60429005601 | Doxazosin Mesylate | Doxazosin Mesylate Tab 8 MG |
| 60429005610 | Doxazosin Mesylate | Doxazosin Mesylate Tab 8 MG |
| 43353051260 | Doxazosin Mesylate | Doxazosin Mesylate Tab 2 MG |
| 43353052260 | Doxazosin Mesylate | Doxazosin Mesylate Tab 4 MG |
| 43353061260 | Doxazosin Mesylate | Doxazosin Mesylate Tab 4 MG |
| 43353061360 | Doxazosin Mesylate | Doxazosin Mesylate Tab 8 MG |
| 66336068230 | Doxazosin Mesylate | Doxazosin Mesylate Tab 2 MG |
| 00904614060 | Doxazosin Mesylate | Doxazosin Mesylate Tab 1 MG |
| 00904614160 | Doxazosin Mesylate | Doxazosin Mesylate Tab 2 MG |
| 00904614260 | Doxazosin Mesylate | Doxazosin Mesylate Tab 4 MG |
| 00904614360 | Doxazosin Mesylate | Doxazosin Mesylate Tab 8 MG |
| 16590027430 | Doxazosin Mesylate | Doxazosin Mesylate Tab 4 MG |
| 00093812019 | Doxazosin Mesylate | Doxazosin Mesylate Tab 1 MG |
| 00093812093 | Doxazosin Mesylate | Doxazosin Mesylate Tab 1 MG |
| 00904614080 | Doxazosin Mesylate | Doxazosin Mesylate Tab 1 MG |
| 00904614180 | Doxazosin Mesylate | Doxazosin Mesylate Tab 2 MG |
| 00904614280 | Doxazosin Mesylate | Doxazosin Mesylate Tab 4 MG |
| 00904614380 | Doxazosin Mesylate | Doxazosin Mesylate Tab 8 MG |
| 43353064460 | Doxazosin Mesylate | Doxazosin Mesylate Tab 2 MG |
| 43353064560 | Doxazosin Mesylate | Doxazosin Mesylate Tab 4 MG |
| 43353064660 | Doxazosin Mesylate | Doxazosin Mesylate Tab 8 MG |
| 43353064760 | Doxazosin Mesylate | Doxazosin Mesylate Tab 2 MG |
| 43353064860 | Doxazosin Mesylate | Doxazosin Mesylate Tab 4 MG |
| 55045305300 | Doxazosin Mesylate | Doxazosin Mesylate Tab 8 MG |
| 63629135502 | Doxazosin Mesylate | Doxazosin Mesylate Tab 4 MG |
| 63629135503 | Doxazosin Mesylate | Doxazosin Mesylate Tab 4 MG |
| 63629135504 | Doxazosin Mesylate | Doxazosin Mesylate Tab 4 MG |
| 63629403701 | Doxazosin Mesylate | Doxazosin Mesylate Tab 2 MG |
| 63629403702 | Doxazosin Mesylate | Doxazosin Mesylate Tab 2 MG |
| 63629403703 | Doxazosin Mesylate | Doxazosin Mesylate Tab 2 MG |
| 68258600803 | Doxazosin Mesylate | Doxazosin Mesylate Tab 8 MG |
| 68258699603 | Doxazosin Mesylate | Doxazosin Mesylate Tab 4 MG |
| 23490547403 | Doxazosin Mesylate | Doxazosin Mesylate Tab 2 MG |
| 23490547603 | Doxazosin Mesylate | Doxazosin Mesylate Tab 8 MG |
| 43353073960 | Doxazosin Mesylate | Doxazosin Mesylate Tab 2 MG |
| 00781500110 | Doxazosin Mesylate | Doxazosin Mesylate Tab 1 MG |
| 00781500210 | Doxazosin Mesylate | Doxazosin Mesylate Tab 2 MG |
| 00781500310 | Doxazosin Mesylate | Doxazosin Mesylate Tab 4 MG |
| 00781500410 | Doxazosin Mesylate | Doxazosin Mesylate Tab 8 MG |
| 54569329700 | Doxazosin Mesylate | Doxazosin Mesylate Tab 8 MG |
| 58016013030 | Doxazosin Mesylate | Doxazosin Mesylate Tab 4 MG |
| 67544107945 | Doxazosin Mesylate | Doxazosin Mesylate Tab 8 MG |
| 67544107960 | Doxazosin Mesylate | Doxazosin Mesylate Tab 8 MG |
| 33358011630 | Doxazosin Mesylate | Doxazosin Mesylate Tab 2 MG |
| 00440747030 | Doxazosin Mesylate | Doxazosin Mesylate Tab 1 MG |
| 00440747060 | Doxazosin Mesylate | Doxazosin Mesylate Tab 1 MG |
| 00440747090 | Doxazosin Mesylate | Doxazosin Mesylate Tab 1 MG |
| 00440747091 | Doxazosin Mesylate | Doxazosin Mesylate Tab 1 MG |
| 00440747130 | Doxazosin Mesylate | Doxazosin Mesylate Tab 2 MG |
| 00440747160 | Doxazosin Mesylate | Doxazosin Mesylate Tab 2 MG |
| 00440747190 | Doxazosin Mesylate | Doxazosin Mesylate Tab 2 MG |
| 00440747191 | Doxazosin Mesylate | Doxazosin Mesylate Tab 2 MG |
| 00440747230 | Doxazosin Mesylate | Doxazosin Mesylate Tab 4 MG |
| 00440747260 | Doxazosin Mesylate | Doxazosin Mesylate Tab 4 MG |
| 00440747290 | Doxazosin Mesylate | Doxazosin Mesylate Tab 4 MG |
| 00440747291 | Doxazosin Mesylate | Doxazosin Mesylate Tab 4 MG |
| 00440747330 | Doxazosin Mesylate | Doxazosin Mesylate Tab 8 MG |
| 00440747360 | Doxazosin Mesylate | Doxazosin Mesylate Tab 8 MG |
| 00440747390 | Doxazosin Mesylate | Doxazosin Mesylate Tab 8 MG |
| 00440747391 | Doxazosin Mesylate | Doxazosin Mesylate Tab 8 MG |
| 68115012130 | Doxazosin Mesylate | Doxazosin Mesylate Tab 2 MG |
| 68115012230 | Doxazosin Mesylate | Doxazosin Mesylate Tab 4 MG |
| 68115012330 | Doxazosin Mesylate | Doxazosin Mesylate Tab 8 MG |
| 23490547503 | Doxazosin Mesylate | Doxazosin Mesylate Tab 4 MG |
| 23490547601 | Doxazosin Mesylate | Doxazosin Mesylate Tab 8 MG |
| 54868476702 | Doxazosin Mesylate | Doxazosin Mesylate Tab 8 MG |
| 54868480204 | Doxazosin Mesylate | Doxazosin Mesylate Tab 4 MG |
| 54868176802 | Doxazosin Mesylate | Doxazosin Mesylate Tab 1 MG |
| 55045313008 | Doxazosin Mesylate | Doxazosin Mesylate Tab 2 MG |
| 55045316108 | Doxazosin Mesylate | Doxazosin Mesylate Tab 1 MG |
| 58016099700 | Doxazosin Mesylate | Doxazosin Mesylate Tab 2 MG |
| 58016099702 | Doxazosin Mesylate | Doxazosin Mesylate Tab 2 MG |
| 58016099730 | Doxazosin Mesylate | Doxazosin Mesylate Tab 2 MG |
| 58016099760 | Doxazosin Mesylate | Doxazosin Mesylate Tab 2 MG |
| 58016099790 | Doxazosin Mesylate | Doxazosin Mesylate Tab 2 MG |
| 60505009408 | Doxazosin Mesylate | Doxazosin Mesylate Tab 2 MG |
| 60505009505 | Doxazosin Mesylate | Doxazosin Mesylate Tab 4 MG |
| 60505009508 | Doxazosin Mesylate | Doxazosin Mesylate Tab 4 MG |
| 60505009608 | Doxazosin Mesylate | Doxazosin Mesylate Tab 8 MG |
| 58016062500 | Doxazosin Mesylate | Doxazosin Mesylate Tab 4 MG |
| 58016062502 | Doxazosin Mesylate | Doxazosin Mesylate Tab 4 MG |
| 58016062530 | Doxazosin Mesylate | Doxazosin Mesylate Tab 4 MG |
| 58016062560 | Doxazosin Mesylate | Doxazosin Mesylate Tab 4 MG |
| 58016062590 | Doxazosin Mesylate | Doxazosin Mesylate Tab 4 MG |
| 58016064402 | Doxazosin Mesylate | Doxazosin Mesylate Tab 8 MG |
| 58016064430 | Doxazosin Mesylate | Doxazosin Mesylate Tab 8 MG |
| 58016064460 | Doxazosin Mesylate | Doxazosin Mesylate Tab 8 MG |
| 58016064490 | Doxazosin Mesylate | Doxazosin Mesylate Tab 8 MG |
| 67544031445 | Doxazosin Mesylate | Doxazosin Mesylate Tab 8 MG |
| 67544031460 | Doxazosin Mesylate | Doxazosin Mesylate Tab 8 MG |
| 67544032545 | Doxazosin Mesylate | Doxazosin Mesylate Tab 8 MG |
| 67544032560 | Doxazosin Mesylate | Doxazosin Mesylate Tab 8 MG |
| 67544027345 | Doxazosin Mesylate | Doxazosin Mesylate Tab 8 MG |
| 67544027360 | Doxazosin Mesylate | Doxazosin Mesylate Tab 8 MG |
| 67544035945 | Doxazosin Mesylate | Doxazosin Mesylate Tab 8 MG |
| 67544035960 | Doxazosin Mesylate | Doxazosin Mesylate Tab 8 MG |
| 67544068345 | Doxazosin Mesylate | Doxazosin Mesylate Tab 8 MG |
| 67544068360 | Doxazosin Mesylate | Doxazosin Mesylate Tab 8 MG |
| 54868480102 | Doxazosin Mesylate | Doxazosin Mesylate Tab 2 MG |
| 57866631002 | Doxazosin Mesylate | Doxazosin Mesylate Tab 1 MG |
| 57866631003 | Doxazosin Mesylate | Doxazosin Mesylate Tab 1 MG |
| 57866631004 | Doxazosin Mesylate | Doxazosin Mesylate Tab 1 MG |
| 57866631101 | Doxazosin Mesylate | Doxazosin Mesylate Tab 2 MG |
| 57866631102 | Doxazosin Mesylate | Doxazosin Mesylate Tab 2 MG |
| 57866631103 | Doxazosin Mesylate | Doxazosin Mesylate Tab 2 MG |
| 57866631104 | Doxazosin Mesylate | Doxazosin Mesylate Tab 2 MG |
| 57866631201 | Doxazosin Mesylate | Doxazosin Mesylate Tab 4 MG |
| 57866631202 | Doxazosin Mesylate | Doxazosin Mesylate Tab 4 MG |
| 57866631203 | Doxazosin Mesylate | Doxazosin Mesylate Tab 4 MG |
| 57866631204 | Doxazosin Mesylate | Doxazosin Mesylate Tab 4 MG |
| 57866661901 | Doxazosin Mesylate | Doxazosin Mesylate Tab 8 MG |
| 57866661902 | Doxazosin Mesylate | Doxazosin Mesylate Tab 8 MG |
| 57866661903 | Doxazosin Mesylate | Doxazosin Mesylate Tab 8 MG |
| 57866661904 | Doxazosin Mesylate | Doxazosin Mesylate Tab 8 MG |
| 58016064400 | Doxazosin Mesylate | Doxazosin Mesylate Tab 8 MG |
| 58177026604 | Doxazosin Mesylate | Doxazosin Mesylate Tab 1 MG |
| 58177026608 | Doxazosin Mesylate | Doxazosin Mesylate Tab 1 MG |
| 58177026622 | Doxazosin Mesylate | Doxazosin Mesylate Tab 1 MG |
| 58177026704 | Doxazosin Mesylate | Doxazosin Mesylate Tab 2 MG |
| 58177026708 | Doxazosin Mesylate | Doxazosin Mesylate Tab 2 MG |
| 58177026722 | Doxazosin Mesylate | Doxazosin Mesylate Tab 2 MG |
| 58177026804 | Doxazosin Mesylate | Doxazosin Mesylate Tab 4 MG |
| 58177026808 | Doxazosin Mesylate | Doxazosin Mesylate Tab 4 MG |
| 58177026822 | Doxazosin Mesylate | Doxazosin Mesylate Tab 4 MG |
| 58177026904 | Doxazosin Mesylate | Doxazosin Mesylate Tab 8 MG |
| 58177026908 | Doxazosin Mesylate | Doxazosin Mesylate Tab 8 MG |
| 58864080990 | Doxazosin Mesylate | Doxazosin Mesylate Tab 2 MG |
| 58864085630 | Doxazosin Mesylate | Doxazosin Mesylate Tab 8 MG |
| 58864086030 | Doxazosin Mesylate | Doxazosin Mesylate Tab 4 MG |
| 60505009300 | Doxazosin Mesylate | Doxazosin Mesylate Tab 1 MG |
| 60505009301 | Doxazosin Mesylate | Doxazosin Mesylate Tab 1 MG |
| 60505009400 | Doxazosin Mesylate | Doxazosin Mesylate Tab 2 MG |
| 60505009401 | Doxazosin Mesylate | Doxazosin Mesylate Tab 2 MG |
| 60505009500 | Doxazosin Mesylate | Doxazosin Mesylate Tab 4 MG |
| 60505009501 | Doxazosin Mesylate | Doxazosin Mesylate Tab 4 MG |
| 60505009600 | Doxazosin Mesylate | Doxazosin Mesylate Tab 8 MG |
| 60505009601 | Doxazosin Mesylate | Doxazosin Mesylate Tab 8 MG |
| 66116043430 | Doxazosin Mesylate | Doxazosin Mesylate Tab 2 MG |
| 66116018930 | Doxazosin Mesylate | Doxazosin Mesylate Tab 4 MG |
| 67253038010 | Doxazosin Mesylate | Doxazosin Mesylate Tab 1 MG |
| 67253038011 | Doxazosin Mesylate | Doxazosin Mesylate Tab 1 MG |
| 67253038015 | Doxazosin Mesylate | Doxazosin Mesylate Tab 1 MG |
| 67253038110 | Doxazosin Mesylate | Doxazosin Mesylate Tab 2 MG |
| 67253038111 | Doxazosin Mesylate | Doxazosin Mesylate Tab 2 MG |
| 67253038115 | Doxazosin Mesylate | Doxazosin Mesylate Tab 2 MG |
| 67253038210 | Doxazosin Mesylate | Doxazosin Mesylate Tab 4 MG |
| 67253038211 | Doxazosin Mesylate | Doxazosin Mesylate Tab 4 MG |
| 67253038215 | Doxazosin Mesylate | Doxazosin Mesylate Tab 4 MG |
| 67253038310 | Doxazosin Mesylate | Doxazosin Mesylate Tab 8 MG |
| 67253038311 | Doxazosin Mesylate | Doxazosin Mesylate Tab 8 MG |
| 67253038315 | Doxazosin Mesylate | Doxazosin Mesylate Tab 8 MG |
| 67253038410 | Doxazosin Mesylate | Doxazosin Mesylate Tab 1 MG |
| 67253038411 | Doxazosin Mesylate | Doxazosin Mesylate Tab 1 MG |
| 67253038415 | Doxazosin Mesylate | Doxazosin Mesylate Tab 1 MG |
| 67253038510 | Doxazosin Mesylate | Doxazosin Mesylate Tab 2 MG |
| 67253038511 | Doxazosin Mesylate | Doxazosin Mesylate Tab 2 MG |
| 67253038515 | Doxazosin Mesylate | Doxazosin Mesylate Tab 2 MG |
| 67253038610 | Doxazosin Mesylate | Doxazosin Mesylate Tab 4 MG |
| 67253038611 | Doxazosin Mesylate | Doxazosin Mesylate Tab 4 MG |
| 67253038615 | Doxazosin Mesylate | Doxazosin Mesylate Tab 4 MG |
| 67253038710 | Doxazosin Mesylate | Doxazosin Mesylate Tab 8 MG |
| 67253038711 | Doxazosin Mesylate | Doxazosin Mesylate Tab 8 MG |
| 67253038715 | Doxazosin Mesylate | Doxazosin Mesylate Tab 8 MG |
| 49884055401 | Doxazosin Mesylate | Doxazosin Mesylate Tab 4 MG |
| 49884055410 | Doxazosin Mesylate | Doxazosin Mesylate Tab 4 MG |
| 49884055501 | Doxazosin Mesylate | Doxazosin Mesylate Tab 8 MG |
| 49884055510 | Doxazosin Mesylate | Doxazosin Mesylate Tab 8 MG |
| 49999020630 | Doxazosin Mesylate | Doxazosin Mesylate Tab 2 MG |
| 50111065101 | Doxazosin Mesylate | Doxazosin Mesylate Tab 1 MG |
| 50111065102 | Doxazosin Mesylate | Doxazosin Mesylate Tab 1 MG |
| 50111065201 | Doxazosin Mesylate | Doxazosin Mesylate Tab 2 MG |
| 50111065202 | Doxazosin Mesylate | Doxazosin Mesylate Tab 2 MG |
| 50111065203 | Doxazosin Mesylate | Doxazosin Mesylate Tab 2 MG |
| 50111065301 | Doxazosin Mesylate | Doxazosin Mesylate Tab 4 MG |
| 50111065302 | Doxazosin Mesylate | Doxazosin Mesylate Tab 4 MG |
| 50111065303 | Doxazosin Mesylate | Doxazosin Mesylate Tab 4 MG |
| 50111065401 | Doxazosin Mesylate | Doxazosin Mesylate Tab 8 MG |
| 50111065402 | Doxazosin Mesylate | Doxazosin Mesylate Tab 8 MG |
| 51079095701 | Doxazosin Mesylate | Doxazosin Mesylate Tab 1 MG |
| 51079095720 | Doxazosin Mesylate | Doxazosin Mesylate Tab 1 MG |
| 51079095801 | Doxazosin Mesylate | Doxazosin Mesylate Tab 2 MG |
| 51079095820 | Doxazosin Mesylate | Doxazosin Mesylate Tab 2 MG |
| 51079095901 | Doxazosin Mesylate | Doxazosin Mesylate Tab 4 MG |
| 51079095920 | Doxazosin Mesylate | Doxazosin Mesylate Tab 4 MG |
| 52544063901 | Doxazosin Mesylate | Doxazosin Mesylate Tab 1 MG |
| 52544063905 | Doxazosin Mesylate | Doxazosin Mesylate Tab 1 MG |
| 52544064001 | Doxazosin Mesylate | Doxazosin Mesylate Tab 2 MG |
| 52544064005 | Doxazosin Mesylate | Doxazosin Mesylate Tab 2 MG |
| 52544064101 | Doxazosin Mesylate | Doxazosin Mesylate Tab 4 MG |
| 52544064105 | Doxazosin Mesylate | Doxazosin Mesylate Tab 4 MG |
| 52544064201 | Doxazosin Mesylate | Doxazosin Mesylate Tab 8 MG |
| 52544064205 | Doxazosin Mesylate | Doxazosin Mesylate Tab 8 MG |
| 54569325000 | Doxazosin Mesylate | Doxazosin Mesylate Tab 4 MG |
| 54569329701 | Doxazosin Mesylate | Doxazosin Mesylate Tab 8 MG |
| 54569486400 | Doxazosin Mesylate | Doxazosin Mesylate Tab 1 MG |
| 54569486500 | Doxazosin Mesylate | Doxazosin Mesylate Tab 2 MG |
| 54569516800 | Doxazosin Mesylate | Doxazosin Mesylate Tab 1 MG |
| 54569516900 | Doxazosin Mesylate | Doxazosin Mesylate Tab 2 MG |
| 54569517000 | Doxazosin Mesylate | Doxazosin Mesylate Tab 4 MG |
| 54569524500 | Doxazosin Mesylate | Doxazosin Mesylate Tab 8 MG |
| 54868176801 | Doxazosin Mesylate | Doxazosin Mesylate Tab 1 MG |
| 54868215100 | Doxazosin Mesylate | Doxazosin Mesylate Tab 2 MG |
| 54868215101 | Doxazosin Mesylate | Doxazosin Mesylate Tab 2 MG |
| 54868215102 | Doxazosin Mesylate | Doxazosin Mesylate Tab 2 MG |
| 54868264001 | Doxazosin Mesylate | Doxazosin Mesylate Tab 4 MG |
| 54868341900 | Doxazosin Mesylate | Doxazosin Mesylate Tab 8 MG |
| 54868341901 | Doxazosin Mesylate | Doxazosin Mesylate Tab 8 MG |
| 54868489500 | Doxazosin Mesylate | Doxazosin Mesylate Tab 1 MG |
| 54868489501 | Doxazosin Mesylate | Doxazosin Mesylate Tab 1 MG |
| 54868476700 | Doxazosin Mesylate | Doxazosin Mesylate Tab 8 MG |
| 54868476701 | Doxazosin Mesylate | Doxazosin Mesylate Tab 8 MG |
| 54868480100 | Doxazosin Mesylate | Doxazosin Mesylate Tab 2 MG |
| 54868480101 | Doxazosin Mesylate | Doxazosin Mesylate Tab 2 MG |
| 54868480201 | Doxazosin Mesylate | Doxazosin Mesylate Tab 4 MG |
| 54868480202 | Doxazosin Mesylate | Doxazosin Mesylate Tab 4 MG |
| 54868480203 | Doxazosin Mesylate | Doxazosin Mesylate Tab 4 MG |
| 55289058430 | Doxazosin Mesylate | Doxazosin Mesylate Tab 8 MG |
| 55289060030 | Doxazosin Mesylate | Doxazosin Mesylate Tab 4 MG |
| 55887050630 | Doxazosin Mesylate | Doxazosin Mesylate Tab 2 MG |
| 55887050660 | Doxazosin Mesylate | Doxazosin Mesylate Tab 2 MG |
| 55887050682 | Doxazosin Mesylate | Doxazosin Mesylate Tab 2 MG |
| 55887050690 | Doxazosin Mesylate | Doxazosin Mesylate Tab 2 MG |
| 55887050730 | Doxazosin Mesylate | Doxazosin Mesylate Tab 4 MG |
| 55887050760 | Doxazosin Mesylate | Doxazosin Mesylate Tab 4 MG |
| 55887050790 | Doxazosin Mesylate | Doxazosin Mesylate Tab 4 MG |
| 57866631001 | Doxazosin Mesylate | Doxazosin Mesylate Tab 1 MG |
| 00049275041 | Doxazosin Mesylate | Doxazosin Mesylate Tab 1 MG |
| 00049275066 | Doxazosin Mesylate | Doxazosin Mesylate Tab 1 MG |
| 00049276041 | Doxazosin Mesylate | Doxazosin Mesylate Tab 2 MG |
| 00049276066 | Doxazosin Mesylate | Doxazosin Mesylate Tab 2 MG |
| 00049277041 | Doxazosin Mesylate | Doxazosin Mesylate Tab 4 MG |
| 00049277066 | Doxazosin Mesylate | Doxazosin Mesylate Tab 4 MG |
| 00049278041 | Doxazosin Mesylate | Doxazosin Mesylate Tab 8 MG |
| 00049278066 | Doxazosin Mesylate | Doxazosin Mesylate Tab 8 MG |
| 00093812001 | Doxazosin Mesylate | Doxazosin Mesylate Tab 1 MG |
| 00093812101 | Doxazosin Mesylate | Doxazosin Mesylate Tab 2 MG |
| 00093812201 | Doxazosin Mesylate | Doxazosin Mesylate Tab 4 MG |
| 00093812301 | Doxazosin Mesylate | Doxazosin Mesylate Tab 8 MG |
| 00172368560 | Doxazosin Mesylate | Doxazosin Mesylate Tab 1 MG |
| 00172368570 | Doxazosin Mesylate | Doxazosin Mesylate Tab 1 MG |
| 00172368660 | Doxazosin Mesylate | Doxazosin Mesylate Tab 2 MG |
| 00172368670 | Doxazosin Mesylate | Doxazosin Mesylate Tab 2 MG |
| 00172368680 | Doxazosin Mesylate | Doxazosin Mesylate Tab 2 MG |
| 00172368760 | Doxazosin Mesylate | Doxazosin Mesylate Tab 4 MG |
| 00172368770 | Doxazosin Mesylate | Doxazosin Mesylate Tab 4 MG |
| 00172368780 | Doxazosin Mesylate | Doxazosin Mesylate Tab 4 MG |
| 00172368860 | Doxazosin Mesylate | Doxazosin Mesylate Tab 8 MG |
| 00172368870 | Doxazosin Mesylate | Doxazosin Mesylate Tab 8 MG |
| 00228264211 | Doxazosin Mesylate | Doxazosin Mesylate Tab 1 MG |
| 00228264250 | Doxazosin Mesylate | Doxazosin Mesylate Tab 1 MG |
| 00228264311 | Doxazosin Mesylate | Doxazosin Mesylate Tab 2 MG |
| 00228264350 | Doxazosin Mesylate | Doxazosin Mesylate Tab 2 MG |
| 00228264411 | Doxazosin Mesylate | Doxazosin Mesylate Tab 4 MG |
| 00228264450 | Doxazosin Mesylate | Doxazosin Mesylate Tab 4 MG |
| 00228264511 | Doxazosin Mesylate | Doxazosin Mesylate Tab 8 MG |
| 00228264550 | Doxazosin Mesylate | Doxazosin Mesylate Tab 8 MG |
| 00378402101 | Doxazosin Mesylate | Doxazosin Mesylate Tab 1 MG |
| 00378402201 | Doxazosin Mesylate | Doxazosin Mesylate Tab 2 MG |
| 00378402401 | Doxazosin Mesylate | Doxazosin Mesylate Tab 4 MG |
| 00378402801 | Doxazosin Mesylate | Doxazosin Mesylate Tab 8 MG |
| 00591063901 | Doxazosin Mesylate | Doxazosin Mesylate Tab 1 MG |
| 00591064001 | Doxazosin Mesylate | Doxazosin Mesylate Tab 2 MG |
| 00591064101 | Doxazosin Mesylate | Doxazosin Mesylate Tab 4 MG |
| 00591064201 | Doxazosin Mesylate | Doxazosin Mesylate Tab 8 MG |
| 00781500101 | Doxazosin Mesylate | Doxazosin Mesylate Tab 1 MG |
| 00781500201 | Doxazosin Mesylate | Doxazosin Mesylate Tab 2 MG |
| 00781500301 | Doxazosin Mesylate | Doxazosin Mesylate Tab 4 MG |
| 00781500401 | Doxazosin Mesylate | Doxazosin Mesylate Tab 8 MG |
| 00904552260 | Doxazosin Mesylate | Doxazosin Mesylate Tab 1 MG |
| 00904552261 | Doxazosin Mesylate | Doxazosin Mesylate Tab 1 MG |
| 00904552360 | Doxazosin Mesylate | Doxazosin Mesylate Tab 2 MG |
| 00904552361 | Doxazosin Mesylate | Doxazosin Mesylate Tab 2 MG |
| 00904552460 | Doxazosin Mesylate | Doxazosin Mesylate Tab 4 MG |
| 00904552461 | Doxazosin Mesylate | Doxazosin Mesylate Tab 4 MG |
| 00904552560 | Doxazosin Mesylate | Doxazosin Mesylate Tab 8 MG |
| 12280021300 | Doxazosin Mesylate | Doxazosin Mesylate Tab 2 MG |
| 49884055201 | Doxazosin Mesylate | Doxazosin Mesylate Tab 1 MG |
| 49884055210 | Doxazosin Mesylate | Doxazosin Mesylate Tab 1 MG |
| 49884055301 | Doxazosin Mesylate | Doxazosin Mesylate Tab 2 MG |
| 49884055310 | Doxazosin Mesylate | Doxazosin Mesylate Tab 2 MG |
| 63629152603 | Enalapril Maleate | Enalapril Maleate Tab 10 MG |
| 21695048930 | Enalapril Maleate | Enalapril Maleate Tab 20 MG |
| 47463022130 | Enalapril Maleate | Enalapril Maleate Tab 10 MG |
| 47463022230 | Enalapril Maleate | Enalapril Maleate Tab 20 MG |
| 47463022330 | Enalapril Maleate | Enalapril Maleate Tab 5 MG |
| 47463022030 | Enalapril Maleate | Enalapril Maleate Tab 2.5 MG |
| 21695048890 | Enalapril Maleate | Enalapril Maleate Tab 10 MG |
| 54868435703 | Enalapril Maleate | Enalapril Maleate Tab 5 MG |
| 51138003330 | Enalapril Maleate | Enalapril Maleate Tab 2.5 MG |
| 51138003430 | Enalapril Maleate | Enalapril Maleate Tab 5 MG |
| 51138003530 | Enalapril Maleate | Enalapril Maleate Tab 10 MG |
| 51138003630 | Enalapril Maleate | Enalapril Maleate Tab 20 MG |
| 66336038990 | Enalapril Maleate | Enalapril Maleate Tab 10 MG |
| 66336039190 | Enalapril Maleate | Enalapril Maleate Tab 5 MG |
| 51138033330 | Enalapril Maleate | Enalapril Maleate Tab 2.5 MG |
| 51138033430 | Enalapril Maleate | Enalapril Maleate Tab 5 MG |
| 51138033530 | Enalapril Maleate | Enalapril Maleate Tab 10 MG |
| 51138033630 | Enalapril Maleate | Enalapril Maleate Tab 20 MG |
| 55048022030 | Enalapril Maleate | Enalapril Maleate Tab 2.5 MG |
| 55048022130 | Enalapril Maleate | Enalapril Maleate Tab 10 MG |
| 55048022230 | Enalapril Maleate | Enalapril Maleate Tab 20 MG |
| 55048022330 | Enalapril Maleate | Enalapril Maleate Tab 5 MG |
| 66336039390 | Enalapril Maleate | Enalapril Maleate Tab 20 MG |
| 55048027730 | Enalapril Maleate | Enalapril Maleate Tab 10 MG |
| 55048027830 | Enalapril Maleate | Enalapril Maleate Tab 20 MG |
| 54868433103 | Enalapril Maleate | Enalapril Maleate Tab 20 MG |
| 35356054130 | Enalapril Maleate | Enalapril Maleate Tab 2.5 MG |
| 43353052160 | Enalapril Maleate | Enalapril Maleate Tab 20 MG |
| 43353052180 | Enalapril Maleate | Enalapril Maleate Tab 20 MG |
| 68084039001 | Enalapril Maleate | Enalapril Maleate Tab 5 MG |
| 68084039011 | Enalapril Maleate | Enalapril Maleate Tab 5 MG |
| 68084039101 | Enalapril Maleate | Enalapril Maleate Tab 10 MG |
| 68084039111 | Enalapril Maleate | Enalapril Maleate Tab 10 MG |
| 68084039201 | Enalapril Maleate | Enalapril Maleate Tab 20 MG |
| 68084039211 | Enalapril Maleate | Enalapril Maleate Tab 20 MG |
| 67544016060 | Enalapril Maleate | Enalapril Maleate Tab 10 MG |
| 67544016560 | Enalapril Maleate | Enalapril Maleate Tab 5 MG |
| 66267032330 | Enalapril Maleate | Enalapril Maleate Tab 10 MG |
| 66267032360 | Enalapril Maleate | Enalapril Maleate Tab 10 MG |
| 66267032390 | Enalapril Maleate | Enalapril Maleate Tab 10 MG |
| 66267032391 | Enalapril Maleate | Enalapril Maleate Tab 10 MG |
| 66267038030 | Enalapril Maleate | Enalapril Maleate Tab 20 MG |
| 66267038060 | Enalapril Maleate | Enalapril Maleate Tab 20 MG |
| 66267038090 | Enalapril Maleate | Enalapril Maleate Tab 20 MG |
| 66267038091 | Enalapril Maleate | Enalapril Maleate Tab 20 MG |
| 66267041330 | Enalapril Maleate | Enalapril Maleate Tab 5 MG |
| 66267041360 | Enalapril Maleate | Enalapril Maleate Tab 5 MG |
| 66267041390 | Enalapril Maleate | Enalapril Maleate Tab 5 MG |
| 66267041392 | Enalapril Maleate | Enalapril Maleate Tab 5 MG |
| 55045282700 | Enalapril Maleate | Enalapril Maleate Tab 10 MG |
| 55045283200 | Enalapril Maleate | Enalapril Maleate Tab 20 MG |
| 60429007427 | Enalapril Maleate | Enalapril Maleate Tab 20 MG |
| 60429007430 | Enalapril Maleate | Enalapril Maleate Tab 20 MG |
| 60429007460 | Enalapril Maleate | Enalapril Maleate Tab 20 MG |
| 60429007490 | Enalapril Maleate | Enalapril Maleate Tab 20 MG |
| 60429007101 | Enalapril Maleate | Enalapril Maleate Tab 2.5 MG |
| 60429007105 | Enalapril Maleate | Enalapril Maleate Tab 2.5 MG |
| 60429007110 | Enalapril Maleate | Enalapril Maleate Tab 2.5 MG |
| 60429007127 | Enalapril Maleate | Enalapril Maleate Tab 2.5 MG |
| 60429007130 | Enalapril Maleate | Enalapril Maleate Tab 2.5 MG |
| 60429007160 | Enalapril Maleate | Enalapril Maleate Tab 2.5 MG |
| 60429007190 | Enalapril Maleate | Enalapril Maleate Tab 2.5 MG |
| 60429007201 | Enalapril Maleate | Enalapril Maleate Tab 5 MG |
| 60429007205 | Enalapril Maleate | Enalapril Maleate Tab 5 MG |
| 60429007210 | Enalapril Maleate | Enalapril Maleate Tab 5 MG |
| 60429007227 | Enalapril Maleate | Enalapril Maleate Tab 5 MG |
| 60429007230 | Enalapril Maleate | Enalapril Maleate Tab 5 MG |
| 60429007260 | Enalapril Maleate | Enalapril Maleate Tab 5 MG |
| 60429007290 | Enalapril Maleate | Enalapril Maleate Tab 5 MG |
| 60429007301 | Enalapril Maleate | Enalapril Maleate Tab 10 MG |
| 60429007305 | Enalapril Maleate | Enalapril Maleate Tab 10 MG |
| 60429007310 | Enalapril Maleate | Enalapril Maleate Tab 10 MG |
| 60429007327 | Enalapril Maleate | Enalapril Maleate Tab 10 MG |
| 60429007330 | Enalapril Maleate | Enalapril Maleate Tab 10 MG |
| 60429007360 | Enalapril Maleate | Enalapril Maleate Tab 10 MG |
| 60429007390 | Enalapril Maleate | Enalapril Maleate Tab 10 MG |
| 60429007401 | Enalapril Maleate | Enalapril Maleate Tab 20 MG |
| 60429007405 | Enalapril Maleate | Enalapril Maleate Tab 20 MG |
| 60429007410 | Enalapril Maleate | Enalapril Maleate Tab 20 MG |
| 63629152201 | Enalapril Maleate | Enalapril Maleate Tab 5 MG |
| 63629152202 | Enalapril Maleate | Enalapril Maleate Tab 5 MG |
| 63629152204 | Enalapril Maleate | Enalapril Maleate Tab 5 MG |
| 00440748790 | Enalapril Maleate | Enalapril Maleate Tab 10 MG |
| 00440748890 | Enalapril Maleate | Enalapril Maleate Tab 20 MG |
| 64679092309 | Enalapril Maleate | Enalapril Maleate Tab 2.5 MG |
| 64679092409 | Enalapril Maleate | Enalapril Maleate Tab 5 MG |
| 64679092509 | Enalapril Maleate | Enalapril Maleate Tab 10 MG |
| 64679092609 | Enalapril Maleate | Enalapril Maleate Tab 20 MG |
| 49158050001 | Enalapril Maleate | Enalapril Maleate Tab 2.5 MG |
| 49158050010 | Enalapril Maleate | Enalapril Maleate Tab 2.5 MG |
| 49158050101 | Enalapril Maleate | Enalapril Maleate Tab 5 MG |
| 49158050110 | Enalapril Maleate | Enalapril Maleate Tab 5 MG |
| 49158050201 | Enalapril Maleate | Enalapril Maleate Tab 10 MG |
| 49158050210 | Enalapril Maleate | Enalapril Maleate Tab 10 MG |
| 49158050301 | Enalapril Maleate | Enalapril Maleate Tab 20 MG |
| 49158050310 | Enalapril Maleate | Enalapril Maleate Tab 20 MG |
| 54868435803 | Enalapril Maleate | Enalapril Maleate Tab 10 MG |
| 55887009930 | Enalapril Maleate | Enalapril Maleate Tab 2.5 MG |
| 16590027730 | Enalapril Maleate | Enalapril Maleate Tab 10 MG |
| 16590027760 | Enalapril Maleate | Enalapril Maleate Tab 10 MG |
| 16590027790 | Enalapril Maleate | Enalapril Maleate Tab 10 MG |
| 16590039230 | Enalapril Maleate | Enalapril Maleate Tab 20 MG |
| 16590039260 | Enalapril Maleate | Enalapril Maleate Tab 20 MG |
| 16590039290 | Enalapril Maleate | Enalapril Maleate Tab 20 MG |
| 00440748590 | Enalapril Maleate | Enalapril Maleate Tab 2.5 MG |
| 00440748630 | Enalapril Maleate | Enalapril Maleate Tab 5 MG |
| 00440748730 | Enalapril Maleate | Enalapril Maleate Tab 10 MG |
| 21695048730 | Enalapril Maleate | Enalapril Maleate Tab 5 MG |
| 21695048830 | Enalapril Maleate | Enalapril Maleate Tab 10 MG |
| 15338020030 | Enalapril Maleate | Enalapril Maleate Tab 2.5 MG |
| 15338021130 | Enalapril Maleate | Enalapril Maleate Tab 5 MG |
| 15338022230 | Enalapril Maleate | Enalapril Maleate Tab 10 MG |
| 15338023330 | Enalapril Maleate | Enalapril Maleate Tab 20 MG |
| 52959094230 | Enalapril Maleate | Enalapril Maleate Tab 20 MG |
| 15338022030 | Enalapril Maleate | Enalapril Maleate Tab 10 MG |
| 00093002750 | Enalapril Maleate | Enalapril Maleate Tab 5 MG |
| 00093002850 | Enalapril Maleate | Enalapril Maleate Tab 10 MG |
| 00093002950 | Enalapril Maleate | Enalapril Maleate Tab 20 MG |
| 21695048990 | Enalapril Maleate | Enalapril Maleate Tab 20 MG |
| 63629152203 | Enalapril Maleate | Enalapril Maleate Tab 5 MG |
| 63629152501 | Enalapril Maleate | Enalapril Maleate Tab 20 MG |
| 63629152502 | Enalapril Maleate | Enalapril Maleate Tab 20 MG |
| 63629152503 | Enalapril Maleate | Enalapril Maleate Tab 20 MG |
| 63629152601 | Enalapril Maleate | Enalapril Maleate Tab 10 MG |
| 63629152602 | Enalapril Maleate | Enalapril Maleate Tab 10 MG |
| 21695048860 | Enalapril Maleate | Enalapril Maleate Tab 10 MG |
| 43353004580 | Enalapril Maleate | Enalapril Maleate Tab 10 MG |
| 00364273401 | Enalapril Maleate | Enalapril Maleate Tab 20 MG |
| 40893069660 | Enalapril Maleate | Enalapril Maleate Tab 5 MG |
| 52246065930 | Enalapril Maleate | Enalapril Maleate Tab 5 MG |
| 52246065990 | Enalapril Maleate | Enalapril Maleate Tab 5 MG |
| 52246066630 | Enalapril Maleate | Enalapril Maleate Tab 10 MG |
| 52493069660 | Enalapril Maleate | Enalapril Maleate Tab 5 MG |
| 53506075230 | Enalapril Maleate | Enalapril Maleate Tab 5 MG |
| 53506075330 | Enalapril Maleate | Enalapril Maleate Tab 10 MG |
| 54124038930 | Enalapril Maleate | Enalapril Maleate Tab 20 MG |
| 54124071230 | Enalapril Maleate | Enalapril Maleate Tab 5 MG |
| 54124071260 | Enalapril Maleate | Enalapril Maleate Tab 5 MG |
| 54124071330 | Enalapril Maleate | Enalapril Maleate Tab 10 MG |
| 54124071360 | Enalapril Maleate | Enalapril Maleate Tab 10 MG |
| 54569060602 | Enalapril Maleate | Enalapril Maleate Tab 5 MG |
| 54569060702 | Enalapril Maleate | Enalapril Maleate Tab 10 MG |
| 54569060703 | Enalapril Maleate | Enalapril Maleate Tab 10 MG |
| 54569061201 | Enalapril Maleate | Enalapril Maleate Tab 20 MG |
| 54569712500 | Enalapril Maleate | Enalapril Maleate Tab 5 MG |
| 54569325800 | Enalapril Maleate | Enalapril Maleate Tab 2.5 MG |
| 54868228001 | Enalapril Maleate | Enalapril Maleate Tab 2.5 MG |
| 54569802000 | Enalapril Maleate | Enalapril Maleate Tab 5 MG |
| 55175395400 | Enalapril Maleate | Enalapril Maleate Tab 5 MG |
| 55175395405 | Enalapril Maleate | Enalapril Maleate Tab 5 MG |
| 55175395503 | Enalapril Maleate | Enalapril Maleate Tab 10 MG |
| 55289098430 | Enalapril Maleate | Enalapril Maleate Tab 20 MG |
| 55370092207 | Enalapril Maleate | Enalapril Maleate Tab 2.5 MG |
| 55370092209 | Enalapril Maleate | Enalapril Maleate Tab 2.5 MG |
| 55370092307 | Enalapril Maleate | Enalapril Maleate Tab 5 MG |
| 55370092309 | Enalapril Maleate | Enalapril Maleate Tab 5 MG |
| 55370092407 | Enalapril Maleate | Enalapril Maleate Tab 10 MG |
| 55370092409 | Enalapril Maleate | Enalapril Maleate Tab 10 MG |
| 55370092507 | Enalapril Maleate | Enalapril Maleate Tab 20 MG |
| 55370092509 | Enalapril Maleate | Enalapril Maleate Tab 20 MG |
| 57362040411 | Enalapril Maleate | Enalapril Maleate Tab 10 MG |
| 57362040413 | Enalapril Maleate | Enalapril Maleate Tab 10 MG |
| 57362040419 | Enalapril Maleate | Enalapril Maleate Tab 10 MG |
| 57362040484 | Enalapril Maleate | Enalapril Maleate Tab 10 MG |
| 57362040511 | Enalapril Maleate | Enalapril Maleate Tab 20 MG |
| 57362040519 | Enalapril Maleate | Enalapril Maleate Tab 20 MG |
| 59772554101 | Enalapril Maleate | Enalapril Maleate Tab 2.5 MG |
| 59772554103 | Enalapril Maleate | Enalapril Maleate Tab 2.5 MG |
| 59772554201 | Enalapril Maleate | Enalapril Maleate Tab 5 MG |
| 59772554203 | Enalapril Maleate | Enalapril Maleate Tab 5 MG |
| 59772554301 | Enalapril Maleate | Enalapril Maleate Tab 10 MG |
| 59772554401 | Enalapril Maleate | Enalapril Maleate Tab 20 MG |
| 59772554403 | Enalapril Maleate | Enalapril Maleate Tab 20 MG |
| 55887022730 | Enalapril Maleate | Enalapril Maleate Tab 20 MG |
| 55887022760 | Enalapril Maleate | Enalapril Maleate Tab 20 MG |
| 33358012630 | Enalapril Maleate | Enalapril Maleate Tab 5 MG |
| 33358012730 | Enalapril Maleate | Enalapril Maleate Tab 10 MG |
| 33358012760 | Enalapril Maleate | Enalapril Maleate Tab 10 MG |
| 33358012830 | Enalapril Maleate | Enalapril Maleate Tab 20 MG |
| 33358012860 | Enalapril Maleate | Enalapril Maleate Tab 20 MG |
| 00440748610 | Enalapril Maleate | Enalapril Maleate Tab 5 MG |
| 00440748660 | Enalapril Maleate | Enalapril Maleate Tab 5 MG |
| 00440748690 | Enalapril Maleate | Enalapril Maleate Tab 5 MG |
| 00440748691 | Enalapril Maleate | Enalapril Maleate Tab 5 MG |
| 23490549401 | Enalapril Maleate | Enalapril Maleate Tab 5 MG |
| 23490549402 | Enalapril Maleate | Enalapril Maleate Tab 5 MG |
| 49999023960 | Enalapril Maleate | Enalapril Maleate Tab 5 MG |
| 52959018030 | Enalapril Maleate | Enalapril Maleate Tab 10 MG |
| 52959018060 | Enalapril Maleate | Enalapril Maleate Tab 10 MG |
| 54868062002 | Enalapril Maleate | Enalapril Maleate Tab 10 MG |
| 54868433101 | Enalapril Maleate | Enalapril Maleate Tab 20 MG |
| 55289059190 | Enalapril Maleate | Enalapril Maleate Tab 10 MG |
| 55289069490 | Enalapril Maleate | Enalapril Maleate Tab 5 MG |
| 64455014030 | Enalapril Maleate | Enalapril Maleate Tab 2.5 MG |
| 64455014130 | Enalapril Maleate | Enalapril Maleate Tab 5 MG |
| 64455014230 | Enalapril Maleate | Enalapril Maleate Tab 10 MG |
| 64455014330 | Enalapril Maleate | Enalapril Maleate Tab 20 MG |
| 63739030210 | Enalapril Maleate | Enalapril Maleate Tab 10 MG |
| 63739032210 | Enalapril Maleate | Enalapril Maleate Tab 2.5 MG |
| 63739032310 | Enalapril Maleate | Enalapril Maleate Tab 5 MG |
| 66336038930 | Enalapril Maleate | Enalapril Maleate Tab 10 MG |
| 66336038960 | Enalapril Maleate | Enalapril Maleate Tab 10 MG |
| 66336039130 | Enalapril Maleate | Enalapril Maleate Tab 5 MG |
| 66336039160 | Enalapril Maleate | Enalapril Maleate Tab 5 MG |
| 66336039330 | Enalapril Maleate | Enalapril Maleate Tab 20 MG |
| 66336039360 | Enalapril Maleate | Enalapril Maleate Tab 20 MG |
| 66267100900 | Enalapril Maleate | Enalapril Maleate Tab 10 MG |
| 67544031580 | Enalapril Maleate | Enalapril Maleate Tab 10 MG |
| 67544035080 | Enalapril Maleate | Enalapril Maleate Tab 2.5 MG |
| 67544016080 | Enalapril Maleate | Enalapril Maleate Tab 10 MG |
| 67544016580 | Enalapril Maleate | Enalapril Maleate Tab 5 MG |
| 67544016680 | Enalapril Maleate | Enalapril Maleate Tab 2.5 MG |
| 67544017580 | Enalapril Maleate | Enalapril Maleate Tab 5 MG |
| 67544021680 | Enalapril Maleate | Enalapril Maleate Tab 10 MG |
| 67544028680 | Enalapril Maleate | Enalapril Maleate Tab 10 MG |
| 68115012700 | Enalapril Maleate | Enalapril Maleate Tab 10 MG |
| 60505004907 | Enalapril Maleate | Enalapril Maleate Tab 2.5 MG |
| 60505004909 | Enalapril Maleate | Enalapril Maleate Tab 2.5 MG |
| 60505005007 | Enalapril Maleate | Enalapril Maleate Tab 5 MG |
| 60505005009 | Enalapril Maleate | Enalapril Maleate Tab 5 MG |
| 60505005107 | Enalapril Maleate | Enalapril Maleate Tab 10 MG |
| 60505005109 | Enalapril Maleate | Enalapril Maleate Tab 10 MG |
| 60505005207 | Enalapril Maleate | Enalapril Maleate Tab 20 MG |
| 60505005209 | Enalapril Maleate | Enalapril Maleate Tab 20 MG |
| 55887098430 | Enalapril Maleate | Enalapril Maleate Tab 2.5 MG |
| 00006001498 | Enalapril Maleate | Enalapril Maleate Tab 2.5 MG |
| 00006071281 | Enalapril Maleate | Enalapril Maleate Tab 5 MG |
| 00006071298 | Enalapril Maleate | Enalapril Maleate Tab 5 MG |
| 00006071381 | Enalapril Maleate | Enalapril Maleate Tab 10 MG |
| 00006071398 | Enalapril Maleate | Enalapril Maleate Tab 10 MG |
| 00364269801 | Enalapril Maleate | Enalapril Maleate Tab 2.5 MG |
| 00364270101 | Enalapril Maleate | Enalapril Maleate Tab 5 MG |
| 00364272701 | Enalapril Maleate | Enalapril Maleate Tab 10 MG |
| 64455014190 | Enalapril Maleate | Enalapril Maleate Tab 5 MG |
| 64455014210 | Enalapril Maleate | Enalapril Maleate Tab 10 MG |
| 64455014211 | Enalapril Maleate | Enalapril Maleate Tab 10 MG |
| 64455014290 | Enalapril Maleate | Enalapril Maleate Tab 10 MG |
| 64455014310 | Enalapril Maleate | Enalapril Maleate Tab 20 MG |
| 64455014311 | Enalapril Maleate | Enalapril Maleate Tab 20 MG |
| 64455014390 | Enalapril Maleate | Enalapril Maleate Tab 20 MG |
| 64679092302 | Enalapril Maleate | Enalapril Maleate Tab 2.5 MG |
| 64679092303 | Enalapril Maleate | Enalapril Maleate Tab 2.5 MG |
| 64679092402 | Enalapril Maleate | Enalapril Maleate Tab 5 MG |
| 64679092403 | Enalapril Maleate | Enalapril Maleate Tab 5 MG |
| 64679092502 | Enalapril Maleate | Enalapril Maleate Tab 10 MG |
| 64679092503 | Enalapril Maleate | Enalapril Maleate Tab 10 MG |
| 64679092602 | Enalapril Maleate | Enalapril Maleate Tab 20 MG |
| 64679092603 | Enalapril Maleate | Enalapril Maleate Tab 20 MG |
| 66116043530 | Enalapril Maleate | Enalapril Maleate Tab 10 MG |
| 66116043630 | Enalapril Maleate | Enalapril Maleate Tab 5 MG |
| 66267025330 | Enalapril Maleate | Enalapril Maleate Tab 5 MG |
| 66685030100 | Enalapril Maleate | Enalapril Maleate Tab 2.5 MG |
| 66685030102 | Enalapril Maleate | Enalapril Maleate Tab 2.5 MG |
| 66685030200 | Enalapril Maleate | Enalapril Maleate Tab 5 MG |
| 66685030202 | Enalapril Maleate | Enalapril Maleate Tab 5 MG |
| 66685030300 | Enalapril Maleate | Enalapril Maleate Tab 10 MG |
| 66685030302 | Enalapril Maleate | Enalapril Maleate Tab 10 MG |
| 66685030400 | Enalapril Maleate | Enalapril Maleate Tab 20 MG |
| 66685030402 | Enalapril Maleate | Enalapril Maleate Tab 20 MG |
| 68115012715 | Enalapril Maleate | Enalapril Maleate Tab 10 MG |
| 68115012730 | Enalapril Maleate | Enalapril Maleate Tab 10 MG |
| 68115012760 | Enalapril Maleate | Enalapril Maleate Tab 10 MG |
| 68115012800 | Enalapril Maleate | Enalapril Maleate Tab 20 MG |
| 68115012820 | Enalapril Maleate | Enalapril Maleate Tab 20 MG |
| 68115012830 | Enalapril Maleate | Enalapril Maleate Tab 20 MG |
| 68115012860 | Enalapril Maleate | Enalapril Maleate Tab 20 MG |
| 68115012930 | Enalapril Maleate | Enalapril Maleate Tab 5 MG |
| 68115012960 | Enalapril Maleate | Enalapril Maleate Tab 5 MG |
| 00781544101 | Enalapril Maleate | Enalapril Maleate Tab 2.5 MG |
| 00781544110 | Enalapril Maleate | Enalapril Maleate Tab 2.5 MG |
| 00781544201 | Enalapril Maleate | Enalapril Maleate Tab 5 MG |
| 00781544210 | Enalapril Maleate | Enalapril Maleate Tab 5 MG |
| 00781544301 | Enalapril Maleate | Enalapril Maleate Tab 10 MG |
| 00781544310 | Enalapril Maleate | Enalapril Maleate Tab 10 MG |
| 00781544401 | Enalapril Maleate | Enalapril Maleate Tab 20 MG |
| 00781544410 | Enalapril Maleate | Enalapril Maleate Tab 20 MG |
| 23490549101 | Enalapril Maleate | Enalapril Maleate Tab 10 MG |
| 23490549102 | Enalapril Maleate | Enalapril Maleate Tab 10 MG |
| 23490549108 | Enalapril Maleate | Enalapril Maleate Tab 10 MG |
| 23490549109 | Enalapril Maleate | Enalapril Maleate Tab 10 MG |
| 23490549201 | Enalapril Maleate | Enalapril Maleate Tab 20 MG |
| 23490549202 | Enalapril Maleate | Enalapril Maleate Tab 20 MG |
| 58016058120 | Enalapril Maleate | Enalapril Maleate Tab 20 MG |
| 58016058130 | Enalapril Maleate | Enalapril Maleate Tab 20 MG |
| 58016058160 | Enalapril Maleate | Enalapril Maleate Tab 20 MG |
| 58864075430 | Enalapril Maleate | Enalapril Maleate Tab 5 MG |
| 58864075530 | Enalapril Maleate | Enalapril Maleate Tab 10 MG |
| 58864076230 | Enalapril Maleate | Enalapril Maleate Tab 20 MG |
| 59772554303 | Enalapril Maleate | Enalapril Maleate Tab 10 MG |
| 60346061230 | Enalapril Maleate | Enalapril Maleate Tab 5 MG |
| 60346061290 | Enalapril Maleate | Enalapril Maleate Tab 5 MG |
| 60346090130 | Enalapril Maleate | Enalapril Maleate Tab 10 MG |
| 60346053430 | Enalapril Maleate | Enalapril Maleate Tab 20 MG |
| 62584016406 | Enalapril Maleate | Enalapril Maleate Tab 2.5 MG |
| 62584016506 | Enalapril Maleate | Enalapril Maleate Tab 5 MG |
| 62584016606 | Enalapril Maleate | Enalapril Maleate Tab 10 MG |
| 62584016706 | Enalapril Maleate | Enalapril Maleate Tab 20 MG |
| 63304052201 | Enalapril Maleate | Enalapril Maleate Tab 2.5 MG |
| 63304052210 | Enalapril Maleate | Enalapril Maleate Tab 2.5 MG |
| 63304052301 | Enalapril Maleate | Enalapril Maleate Tab 5 MG |
| 63304052310 | Enalapril Maleate | Enalapril Maleate Tab 5 MG |
| 63304052401 | Enalapril Maleate | Enalapril Maleate Tab 10 MG |
| 63304052410 | Enalapril Maleate | Enalapril Maleate Tab 10 MG |
| 63304052501 | Enalapril Maleate | Enalapril Maleate Tab 20 MG |
| 63304052510 | Enalapril Maleate | Enalapril Maleate Tab 20 MG |
| 63304083401 | Enalapril Maleate | Enalapril Maleate Tab 2.5 MG |
| 63304083410 | Enalapril Maleate | Enalapril Maleate Tab 2.5 MG |
| 63304083501 | Enalapril Maleate | Enalapril Maleate Tab 5 MG |
| 63304083510 | Enalapril Maleate | Enalapril Maleate Tab 5 MG |
| 63304083601 | Enalapril Maleate | Enalapril Maleate Tab 10 MG |
| 63304083610 | Enalapril Maleate | Enalapril Maleate Tab 10 MG |
| 63304083701 | Enalapril Maleate | Enalapril Maleate Tab 20 MG |
| 63304083710 | Enalapril Maleate | Enalapril Maleate Tab 20 MG |
| 63739030215 | Enalapril Maleate | Enalapril Maleate Tab 10 MG |
| 63739032215 | Enalapril Maleate | Enalapril Maleate Tab 2.5 MG |
| 63739032315 | Enalapril Maleate | Enalapril Maleate Tab 5 MG |
| 63874065501 | Enalapril Maleate | Enalapril Maleate Tab 10 MG |
| 63874065510 | Enalapril Maleate | Enalapril Maleate Tab 10 MG |
| 63874065514 | Enalapril Maleate | Enalapril Maleate Tab 10 MG |
| 63874065520 | Enalapril Maleate | Enalapril Maleate Tab 10 MG |
| 63874065530 | Enalapril Maleate | Enalapril Maleate Tab 10 MG |
| 63874065560 | Enalapril Maleate | Enalapril Maleate Tab 10 MG |
| 63874042301 | Enalapril Maleate | Enalapril Maleate Tab 5 MG |
| 63874042307 | Enalapril Maleate | Enalapril Maleate Tab 5 MG |
| 63874042310 | Enalapril Maleate | Enalapril Maleate Tab 5 MG |
| 63874042314 | Enalapril Maleate | Enalapril Maleate Tab 5 MG |
| 63874042320 | Enalapril Maleate | Enalapril Maleate Tab 5 MG |
| 63874042330 | Enalapril Maleate | Enalapril Maleate Tab 5 MG |
| 63874042360 | Enalapril Maleate | Enalapril Maleate Tab 5 MG |
| 63874098701 | Enalapril Maleate | Enalapril Maleate Tab 20 MG |
| 63874098710 | Enalapril Maleate | Enalapril Maleate Tab 20 MG |
| 63874098714 | Enalapril Maleate | Enalapril Maleate Tab 20 MG |
| 63874098720 | Enalapril Maleate | Enalapril Maleate Tab 20 MG |
| 63874098730 | Enalapril Maleate | Enalapril Maleate Tab 20 MG |
| 63874098760 | Enalapril Maleate | Enalapril Maleate Tab 20 MG |
| 64455014010 | Enalapril Maleate | Enalapril Maleate Tab 2.5 MG |
| 64455014011 | Enalapril Maleate | Enalapril Maleate Tab 2.5 MG |
| 64455014090 | Enalapril Maleate | Enalapril Maleate Tab 2.5 MG |
| 64455014110 | Enalapril Maleate | Enalapril Maleate Tab 5 MG |
| 64455014111 | Enalapril Maleate | Enalapril Maleate Tab 5 MG |
| 54868435800 | Enalapril Maleate | Enalapril Maleate Tab 10 MG |
| 54868435801 | Enalapril Maleate | Enalapril Maleate Tab 10 MG |
| 54868435802 | Enalapril Maleate | Enalapril Maleate Tab 10 MG |
| 55045182008 | Enalapril Maleate | Enalapril Maleate Tab 10 MG |
| 55045279900 | Enalapril Maleate | Enalapril Maleate Tab 5 MG |
| 55045282708 | Enalapril Maleate | Enalapril Maleate Tab 10 MG |
| 55045283208 | Enalapril Maleate | Enalapril Maleate Tab 20 MG |
| 55289059130 | Enalapril Maleate | Enalapril Maleate Tab 10 MG |
| 55289062203 | Enalapril Maleate | Enalapril Maleate Tab 5 MG |
| 55289062230 | Enalapril Maleate | Enalapril Maleate Tab 5 MG |
| 55289069410 | Enalapril Maleate | Enalapril Maleate Tab 5 MG |
| 55289069430 | Enalapril Maleate | Enalapril Maleate Tab 5 MG |
| 55289048330 | Enalapril Maleate | Enalapril Maleate Tab 10 MG |
| 55887055830 | Enalapril Maleate | Enalapril Maleate Tab 20 MG |
| 55887055860 | Enalapril Maleate | Enalapril Maleate Tab 20 MG |
| 55887055882 | Enalapril Maleate | Enalapril Maleate Tab 20 MG |
| 55887055890 | Enalapril Maleate | Enalapril Maleate Tab 20 MG |
| 55887059630 | Enalapril Maleate | Enalapril Maleate Tab 5 MG |
| 55887059660 | Enalapril Maleate | Enalapril Maleate Tab 5 MG |
| 55887059682 | Enalapril Maleate | Enalapril Maleate Tab 5 MG |
| 55887059690 | Enalapril Maleate | Enalapril Maleate Tab 5 MG |
| 55887061230 | Enalapril Maleate | Enalapril Maleate Tab 10 MG |
| 55887061260 | Enalapril Maleate | Enalapril Maleate Tab 10 MG |
| 55887061282 | Enalapril Maleate | Enalapril Maleate Tab 10 MG |
| 55887061290 | Enalapril Maleate | Enalapril Maleate Tab 10 MG |
| 57866685901 | Enalapril Maleate | Enalapril Maleate Tab 5 MG |
| 57866685902 | Enalapril Maleate | Enalapril Maleate Tab 5 MG |
| 57866685903 | Enalapril Maleate | Enalapril Maleate Tab 5 MG |
| 57866685904 | Enalapril Maleate | Enalapril Maleate Tab 5 MG |
| 58016057100 | Enalapril Maleate | Enalapril Maleate Tab 20 MG |
| 58016057130 | Enalapril Maleate | Enalapril Maleate Tab 20 MG |
| 58016057160 | Enalapril Maleate | Enalapril Maleate Tab 20 MG |
| 58016057190 | Enalapril Maleate | Enalapril Maleate Tab 20 MG |
| 58016057900 | Enalapril Maleate | Enalapril Maleate Tab 5 MG |
| 58016057920 | Enalapril Maleate | Enalapril Maleate Tab 5 MG |
| 58016057930 | Enalapril Maleate | Enalapril Maleate Tab 5 MG |
| 58016057960 | Enalapril Maleate | Enalapril Maleate Tab 5 MG |
| 58016058000 | Enalapril Maleate | Enalapril Maleate Tab 10 MG |
| 58016058020 | Enalapril Maleate | Enalapril Maleate Tab 10 MG |
| 58016058030 | Enalapril Maleate | Enalapril Maleate Tab 10 MG |
| 58016058060 | Enalapril Maleate | Enalapril Maleate Tab 10 MG |
| 58016058100 | Enalapril Maleate | Enalapril Maleate Tab 20 MG |
| 50111089403 | Enalapril Maleate | Enalapril Maleate Tab 20 MG |
| 51079095001 | Enalapril Maleate | Enalapril Maleate Tab 2.5 MG |
| 51079095020 | Enalapril Maleate | Enalapril Maleate Tab 2.5 MG |
| 51079095101 | Enalapril Maleate | Enalapril Maleate Tab 5 MG |
| 51079095120 | Enalapril Maleate | Enalapril Maleate Tab 5 MG |
| 51079095201 | Enalapril Maleate | Enalapril Maleate Tab 10 MG |
| 51079095220 | Enalapril Maleate | Enalapril Maleate Tab 10 MG |
| 51079095301 | Enalapril Maleate | Enalapril Maleate Tab 20 MG |
| 51079095320 | Enalapril Maleate | Enalapril Maleate Tab 20 MG |
| 51655028624 | Enalapril Maleate | Enalapril Maleate Tab 5 MG |
| 51655028724 | Enalapril Maleate | Enalapril Maleate Tab 10 MG |
| 51672403701 | Enalapril Maleate | Enalapril Maleate Tab 2.5 MG |
| 51672403703 | Enalapril Maleate | Enalapril Maleate Tab 2.5 MG |
| 51672403801 | Enalapril Maleate | Enalapril Maleate Tab 5 MG |
| 51672403803 | Enalapril Maleate | Enalapril Maleate Tab 5 MG |
| 51672403901 | Enalapril Maleate | Enalapril Maleate Tab 10 MG |
| 51672403903 | Enalapril Maleate | Enalapril Maleate Tab 10 MG |
| 51672404001 | Enalapril Maleate | Enalapril Maleate Tab 20 MG |
| 51672404003 | Enalapril Maleate | Enalapril Maleate Tab 20 MG |
| 52544066801 | Enalapril Maleate | Enalapril Maleate Tab 2.5 MG |
| 52544066805 | Enalapril Maleate | Enalapril Maleate Tab 2.5 MG |
| 52544066901 | Enalapril Maleate | Enalapril Maleate Tab 5 MG |
| 52544066905 | Enalapril Maleate | Enalapril Maleate Tab 5 MG |
| 52544067001 | Enalapril Maleate | Enalapril Maleate Tab 10 MG |
| 52544067005 | Enalapril Maleate | Enalapril Maleate Tab 10 MG |
| 52544067101 | Enalapril Maleate | Enalapril Maleate Tab 20 MG |
| 54569060600 | Enalapril Maleate | Enalapril Maleate Tab 5 MG |
| 54569060601 | Enalapril Maleate | Enalapril Maleate Tab 5 MG |
| 54569060700 | Enalapril Maleate | Enalapril Maleate Tab 10 MG |
| 54569060701 | Enalapril Maleate | Enalapril Maleate Tab 10 MG |
| 54569061200 | Enalapril Maleate | Enalapril Maleate Tab 20 MG |
| 54569325801 | Enalapril Maleate | Enalapril Maleate Tab 2.5 MG |
| 54569513200 | Enalapril Maleate | Enalapril Maleate Tab 2.5 MG |
| 54569513300 | Enalapril Maleate | Enalapril Maleate Tab 5 MG |
| 54569513301 | Enalapril Maleate | Enalapril Maleate Tab 5 MG |
| 54569513400 | Enalapril Maleate | Enalapril Maleate Tab 10 MG |
| 54569513401 | Enalapril Maleate | Enalapril Maleate Tab 10 MG |
| 54569513402 | Enalapril Maleate | Enalapril Maleate Tab 10 MG |
| 54569513500 | Enalapril Maleate | Enalapril Maleate Tab 20 MG |
| 54569513501 | Enalapril Maleate | Enalapril Maleate Tab 20 MG |
| 54868054100 | Enalapril Maleate | Enalapril Maleate Tab 20 MG |
| 54868054101 | Enalapril Maleate | Enalapril Maleate Tab 20 MG |
| 54868054103 | Enalapril Maleate | Enalapril Maleate Tab 20 MG |
| 54868062001 | Enalapril Maleate | Enalapril Maleate Tab 10 MG |
| 54868062003 | Enalapril Maleate | Enalapril Maleate Tab 10 MG |
| 54868062005 | Enalapril Maleate | Enalapril Maleate Tab 10 MG |
| 54868228000 | Enalapril Maleate | Enalapril Maleate Tab 2.5 MG |
| 54868228002 | Enalapril Maleate | Enalapril Maleate Tab 2.5 MG |
| 54868109001 | Enalapril Maleate | Enalapril Maleate Tab 5 MG |
| 54868109005 | Enalapril Maleate | Enalapril Maleate Tab 5 MG |
| 54868109006 | Enalapril Maleate | Enalapril Maleate Tab 5 MG |
| 54868433100 | Enalapril Maleate | Enalapril Maleate Tab 20 MG |
| 54868433102 | Enalapril Maleate | Enalapril Maleate Tab 20 MG |
| 54868433200 | Enalapril Maleate | Enalapril Maleate Tab 2.5 MG |
| 54868433201 | Enalapril Maleate | Enalapril Maleate Tab 2.5 MG |
| 54868433202 | Enalapril Maleate | Enalapril Maleate Tab 2.5 MG |
| 54868435700 | Enalapril Maleate | Enalapril Maleate Tab 5 MG |
| 54868435701 | Enalapril Maleate | Enalapril Maleate Tab 5 MG |
| 54868435702 | Enalapril Maleate | Enalapril Maleate Tab 5 MG |
| 00781122910 | Enalapril Maleate | Enalapril Maleate Tab 2.5 MG |
| 00781122913 | Enalapril Maleate | Enalapril Maleate Tab 2.5 MG |
| 00781123101 | Enalapril Maleate | Enalapril Maleate Tab 5 MG |
| 00781123110 | Enalapril Maleate | Enalapril Maleate Tab 5 MG |
| 00781123113 | Enalapril Maleate | Enalapril Maleate Tab 5 MG |
| 00781123201 | Enalapril Maleate | Enalapril Maleate Tab 10 MG |
| 00781123210 | Enalapril Maleate | Enalapril Maleate Tab 10 MG |
| 00781123213 | Enalapril Maleate | Enalapril Maleate Tab 10 MG |
| 00781123301 | Enalapril Maleate | Enalapril Maleate Tab 20 MG |
| 00781123310 | Enalapril Maleate | Enalapril Maleate Tab 20 MG |
| 00904550160 | Enalapril Maleate | Enalapril Maleate Tab 2.5 MG |
| 00904550260 | Enalapril Maleate | Enalapril Maleate Tab 5 MG |
| 00904550261 | Enalapril Maleate | Enalapril Maleate Tab 5 MG |
| 00904550280 | Enalapril Maleate | Enalapril Maleate Tab 5 MG |
| 00904550360 | Enalapril Maleate | Enalapril Maleate Tab 10 MG |
| 00904550380 | Enalapril Maleate | Enalapril Maleate Tab 10 MG |
| 00904550460 | Enalapril Maleate | Enalapril Maleate Tab 20 MG |
| 00904550480 | Enalapril Maleate | Enalapril Maleate Tab 20 MG |
| 00904560960 | Enalapril Maleate | Enalapril Maleate Tab 2.5 MG |
| 00904560961 | Enalapril Maleate | Enalapril Maleate Tab 2.5 MG |
| 00904561060 | Enalapril Maleate | Enalapril Maleate Tab 10 MG |
| 00904561061 | Enalapril Maleate | Enalapril Maleate Tab 10 MG |
| 00904561080 | Enalapril Maleate | Enalapril Maleate Tab 10 MG |
| 00904561160 | Enalapril Maleate | Enalapril Maleate Tab 20 MG |
| 00904561161 | Enalapril Maleate | Enalapril Maleate Tab 20 MG |
| 00904561180 | Enalapril Maleate | Enalapril Maleate Tab 20 MG |
| 00904570161 | Enalapril Maleate | Enalapril Maleate Tab 5 MG |
| 17236099704 | Enalapril Maleate | Enalapril Maleate Tab 5 MG |
| 17236099730 | Enalapril Maleate | Enalapril Maleate Tab 5 MG |
| 48866030100 | Enalapril Maleate | Enalapril Maleate Tab 2.5 MG |
| 48866030102 | Enalapril Maleate | Enalapril Maleate Tab 2.5 MG |
| 48866030200 | Enalapril Maleate | Enalapril Maleate Tab 5 MG |
| 48866030202 | Enalapril Maleate | Enalapril Maleate Tab 5 MG |
| 48866030300 | Enalapril Maleate | Enalapril Maleate Tab 10 MG |
| 48866030302 | Enalapril Maleate | Enalapril Maleate Tab 10 MG |
| 48866030400 | Enalapril Maleate | Enalapril Maleate Tab 20 MG |
| 48866030402 | Enalapril Maleate | Enalapril Maleate Tab 20 MG |
| 49999023900 | Enalapril Maleate | Enalapril Maleate Tab 5 MG |
| 49999023930 | Enalapril Maleate | Enalapril Maleate Tab 5 MG |
| 49999024000 | Enalapril Maleate | Enalapril Maleate Tab 10 MG |
| 49999024030 | Enalapril Maleate | Enalapril Maleate Tab 10 MG |
| 49884059101 | Enalapril Maleate | Enalapril Maleate Tab 2.5 MG |
| 49884059110 | Enalapril Maleate | Enalapril Maleate Tab 2.5 MG |
| 49884059201 | Enalapril Maleate | Enalapril Maleate Tab 5 MG |
| 49884059210 | Enalapril Maleate | Enalapril Maleate Tab 5 MG |
| 49884059301 | Enalapril Maleate | Enalapril Maleate Tab 10 MG |
| 49884059310 | Enalapril Maleate | Enalapril Maleate Tab 10 MG |
| 49884059401 | Enalapril Maleate | Enalapril Maleate Tab 20 MG |
| 49884059410 | Enalapril Maleate | Enalapril Maleate Tab 20 MG |
| 49999034500 | Enalapril Maleate | Enalapril Maleate Tab 20 MG |
| 49999034530 | Enalapril Maleate | Enalapril Maleate Tab 20 MG |
| 50111089101 | Enalapril Maleate | Enalapril Maleate Tab 2.5 MG |
| 50111089103 | Enalapril Maleate | Enalapril Maleate Tab 2.5 MG |
| 50111089201 | Enalapril Maleate | Enalapril Maleate Tab 5 MG |
| 50111089203 | Enalapril Maleate | Enalapril Maleate Tab 5 MG |
| 50111089301 | Enalapril Maleate | Enalapril Maleate Tab 10 MG |
| 50111089303 | Enalapril Maleate | Enalapril Maleate Tab 10 MG |
| 50111089401 | Enalapril Maleate | Enalapril Maleate Tab 20 MG |
| 00172419780 | Enalapril Maleate | Enalapril Maleate Tab 10 MG |
| 00172419785 | Enalapril Maleate | Enalapril Maleate Tab 10 MG |
| 00172419793 | Enalapril Maleate | Enalapril Maleate Tab 10 MG |
| 00172419810 | Enalapril Maleate | Enalapril Maleate Tab 20 MG |
| 00172419860 | Enalapril Maleate | Enalapril Maleate Tab 20 MG |
| 00172419864 | Enalapril Maleate | Enalapril Maleate Tab 20 MG |
| 00172419870 | Enalapril Maleate | Enalapril Maleate Tab 20 MG |
| 00172419880 | Enalapril Maleate | Enalapril Maleate Tab 20 MG |
| 00172419893 | Enalapril Maleate | Enalapril Maleate Tab 20 MG |
| 00185014701 | Enalapril Maleate | Enalapril Maleate Tab 10 MG |
| 00185014710 | Enalapril Maleate | Enalapril Maleate Tab 10 MG |
| 00185014750 | Enalapril Maleate | Enalapril Maleate Tab 10 MG |
| 00185021401 | Enalapril Maleate | Enalapril Maleate Tab 20 MG |
| 00185021410 | Enalapril Maleate | Enalapril Maleate Tab 20 MG |
| 00185021450 | Enalapril Maleate | Enalapril Maleate Tab 20 MG |
| 00185011401 | Enalapril Maleate | Enalapril Maleate Tab 2.5 MG |
| 00185011410 | Enalapril Maleate | Enalapril Maleate Tab 2.5 MG |
| 00185011450 | Enalapril Maleate | Enalapril Maleate Tab 2.5 MG |
| 00185012701 | Enalapril Maleate | Enalapril Maleate Tab 5 MG |
| 00185012710 | Enalapril Maleate | Enalapril Maleate Tab 5 MG |
| 00185012750 | Enalapril Maleate | Enalapril Maleate Tab 5 MG |
| 00228265811 | Enalapril Maleate | Enalapril Maleate Tab 2.5 MG |
| 00228265896 | Enalapril Maleate | Enalapril Maleate Tab 2.5 MG |
| 00228265911 | Enalapril Maleate | Enalapril Maleate Tab 5 MG |
| 00228265996 | Enalapril Maleate | Enalapril Maleate Tab 5 MG |
| 00228266011 | Enalapril Maleate | Enalapril Maleate Tab 10 MG |
| 00228266096 | Enalapril Maleate | Enalapril Maleate Tab 10 MG |
| 00228266111 | Enalapril Maleate | Enalapril Maleate Tab 20 MG |
| 00228266196 | Enalapril Maleate | Enalapril Maleate Tab 20 MG |
| 00378105101 | Enalapril Maleate | Enalapril Maleate Tab 2.5 MG |
| 00378105105 | Enalapril Maleate | Enalapril Maleate Tab 2.5 MG |
| 00378105201 | Enalapril Maleate | Enalapril Maleate Tab 5 MG |
| 00378105210 | Enalapril Maleate | Enalapril Maleate Tab 5 MG |
| 00378105301 | Enalapril Maleate | Enalapril Maleate Tab 10 MG |
| 00378105310 | Enalapril Maleate | Enalapril Maleate Tab 10 MG |
| 00378105401 | Enalapril Maleate | Enalapril Maleate Tab 20 MG |
| 00378105405 | Enalapril Maleate | Enalapril Maleate Tab 20 MG |
| 00364269802 | Enalapril Maleate | Enalapril Maleate Tab 2.5 MG |
| 00364270102 | Enalapril Maleate | Enalapril Maleate Tab 5 MG |
| 00364272702 | Enalapril Maleate | Enalapril Maleate Tab 10 MG |
| 00364273402 | Enalapril Maleate | Enalapril Maleate Tab 20 MG |
| 00591066801 | Enalapril Maleate | Enalapril Maleate Tab 2.5 MG |
| 00591066805 | Enalapril Maleate | Enalapril Maleate Tab 2.5 MG |
| 00591066901 | Enalapril Maleate | Enalapril Maleate Tab 5 MG |
| 00591066905 | Enalapril Maleate | Enalapril Maleate Tab 5 MG |
| 00591067001 | Enalapril Maleate | Enalapril Maleate Tab 10 MG |
| 00591067005 | Enalapril Maleate | Enalapril Maleate Tab 10 MG |
| 00591067101 | Enalapril Maleate | Enalapril Maleate Tab 20 MG |
| 00591067105 | Enalapril Maleate | Enalapril Maleate Tab 20 MG |
| 00615459053 | Enalapril Maleate | Enalapril Maleate Tab 5 MG |
| 00615459063 | Enalapril Maleate | Enalapril Maleate Tab 5 MG |
| 00615459153 | Enalapril Maleate | Enalapril Maleate Tab 10 MG |
| 00615459163 | Enalapril Maleate | Enalapril Maleate Tab 10 MG |
| 00781122901 | Enalapril Maleate | Enalapril Maleate Tab 2.5 MG |
| 00006001428 | Enalapril Maleate | Enalapril Maleate Tab 2.5 MG |
| 00006001468 | Enalapril Maleate | Enalapril Maleate Tab 2.5 MG |
| 00006001482 | Enalapril Maleate | Enalapril Maleate Tab 2.5 MG |
| 00006001487 | Enalapril Maleate | Enalapril Maleate Tab 2.5 MG |
| 00006001494 | Enalapril Maleate | Enalapril Maleate Tab 2.5 MG |
| 00006071228 | Enalapril Maleate | Enalapril Maleate Tab 5 MG |
| 00006071268 | Enalapril Maleate | Enalapril Maleate Tab 5 MG |
| 00006071282 | Enalapril Maleate | Enalapril Maleate Tab 5 MG |
| 00006071287 | Enalapril Maleate | Enalapril Maleate Tab 5 MG |
| 00006071294 | Enalapril Maleate | Enalapril Maleate Tab 5 MG |
| 00006071328 | Enalapril Maleate | Enalapril Maleate Tab 10 MG |
| 00006071368 | Enalapril Maleate | Enalapril Maleate Tab 10 MG |
| 00006071382 | Enalapril Maleate | Enalapril Maleate Tab 10 MG |
| 00006071387 | Enalapril Maleate | Enalapril Maleate Tab 10 MG |
| 00006071394 | Enalapril Maleate | Enalapril Maleate Tab 10 MG |
| 00006071428 | Enalapril Maleate | Enalapril Maleate Tab 20 MG |
| 00006071468 | Enalapril Maleate | Enalapril Maleate Tab 20 MG |
| 00006071482 | Enalapril Maleate | Enalapril Maleate Tab 20 MG |
| 00006071487 | Enalapril Maleate | Enalapril Maleate Tab 20 MG |
| 00006071494 | Enalapril Maleate | Enalapril Maleate Tab 20 MG |
| 00093002601 | Enalapril Maleate | Enalapril Maleate Tab 2.5 MG |
| 00093002610 | Enalapril Maleate | Enalapril Maleate Tab 2.5 MG |
| 00093002701 | Enalapril Maleate | Enalapril Maleate Tab 5 MG |
| 00093002710 | Enalapril Maleate | Enalapril Maleate Tab 5 MG |
| 00093002801 | Enalapril Maleate | Enalapril Maleate Tab 10 MG |
| 00093002810 | Enalapril Maleate | Enalapril Maleate Tab 10 MG |
| 00093002901 | Enalapril Maleate | Enalapril Maleate Tab 20 MG |
| 00093002910 | Enalapril Maleate | Enalapril Maleate Tab 20 MG |
| 00172419510 | Enalapril Maleate | Enalapril Maleate Tab 2.5 MG |
| 00172419560 | Enalapril Maleate | Enalapril Maleate Tab 2.5 MG |
| 00172419564 | Enalapril Maleate | Enalapril Maleate Tab 2.5 MG |
| 00172419580 | Enalapril Maleate | Enalapril Maleate Tab 2.5 MG |
| 00172419585 | Enalapril Maleate | Enalapril Maleate Tab 2.5 MG |
| 00172419593 | Enalapril Maleate | Enalapril Maleate Tab 2.5 MG |
| 00172419610 | Enalapril Maleate | Enalapril Maleate Tab 5 MG |
| 00172419660 | Enalapril Maleate | Enalapril Maleate Tab 5 MG |
| 00172419664 | Enalapril Maleate | Enalapril Maleate Tab 5 MG |
| 00172419680 | Enalapril Maleate | Enalapril Maleate Tab 5 MG |
| 00172419685 | Enalapril Maleate | Enalapril Maleate Tab 5 MG |
| 00172419693 | Enalapril Maleate | Enalapril Maleate Tab 5 MG |
| 00172419710 | Enalapril Maleate | Enalapril Maleate Tab 10 MG |
| 00172419760 | Enalapril Maleate | Enalapril Maleate Tab 10 MG |
| 00172419764 | Enalapril Maleate | Enalapril Maleate Tab 10 MG |
| 51672404601 | Enalapril Maleate & Hydrochlorothiazide | Enalapril Maleate & Hydrochlorothiazide Tab 10-25 MG |
| 49884068601 | Enalapril Maleate & Hydrochlorothiazide | Enalapril Maleate & Hydrochlorothiazide Tab 5-12.5 MG |
| 49884068701 | Enalapril Maleate & Hydrochlorothiazide | Enalapril Maleate & Hydrochlorothiazide Tab 10-25 MG |
| 00185015101 | Enalapril Maleate & Hydrochlorothiazide | Enalapril Maleate & Hydrochlorothiazide Tab 5-12.5 MG |
| 00185017201 | Enalapril Maleate & Hydrochlorothiazide | Enalapril Maleate & Hydrochlorothiazide Tab 10-25 MG |
| 00185017210 | Enalapril Maleate & Hydrochlorothiazide | Enalapril Maleate & Hydrochlorothiazide Tab 10-25 MG |
| 00378071201 | Enalapril Maleate & Hydrochlorothiazide | Enalapril Maleate & Hydrochlorothiazide Tab 5-12.5 MG |
| 00378072301 | Enalapril Maleate & Hydrochlorothiazide | Enalapril Maleate & Hydrochlorothiazide Tab 10-25 MG |
| 00006017368 | Enalapril Maleate & Hydrochlorothiazide | Enalapril Maleate & Hydrochlorothiazide Tab 5-12.5 MG |
| 00006072068 | Enalapril Maleate & Hydrochlorothiazide | Enalapril Maleate & Hydrochlorothiazide Tab 10-25 MG |
| 00093104401 | Enalapril Maleate & Hydrochlorothiazide | Enalapril Maleate & Hydrochlorothiazide Tab 5-12.5 MG |
| 00093105201 | Enalapril Maleate & Hydrochlorothiazide | Enalapril Maleate & Hydrochlorothiazide Tab 10-25 MG |
| 00093105210 | Enalapril Maleate & Hydrochlorothiazide | Enalapril Maleate & Hydrochlorothiazide Tab 10-25 MG |
| 63629321701 | Enalapril Maleate & Hydrochlorothiazide | Enalapril Maleate & Hydrochlorothiazide Tab 10-25 MG |
| 54868510001 | Enalapril Maleate & Hydrochlorothiazide | Enalapril Maleate & Hydrochlorothiazide Tab 10-25 MG |
| 51138044330 | Enalapril Maleate & Hydrochlorothiazide | Enalapril Maleate & Hydrochlorothiazide Tab 5-12.5 MG |
| 51138044430 | Enalapril Maleate & Hydrochlorothiazide | Enalapril Maleate & Hydrochlorothiazide Tab 10-25 MG |
| 51138032730 | Enalapril Maleate & Hydrochlorothiazide | Enalapril Maleate & Hydrochlorothiazide Tab 5-12.5 MG |
| 51138032830 | Enalapril Maleate & Hydrochlorothiazide | Enalapril Maleate & Hydrochlorothiazide Tab 10-25 MG |
| 00187014601 | Enalapril Maleate & Hydrochlorothiazide | Enalapril Maleate & Hydrochlorothiazide Tab 10-25 MG |
| 54868510002 | Enalapril Maleate & Hydrochlorothiazide | Enalapril Maleate & Hydrochlorothiazide Tab 10-25 MG |
| 21695078030 | Enalapril Maleate & Hydrochlorothiazide | Enalapril Maleate & Hydrochlorothiazide Tab 10-25 MG |
| 54124072030 | Enalapril Maleate & Hydrochlorothiazide | Enalapril Maleate & Hydrochlorothiazide Tab 10-25 MG |
| 54569060400 | Enalapril Maleate & Hydrochlorothiazide | Enalapril Maleate & Hydrochlorothiazide Tab 10-25 MG |
| 54569060401 | Enalapril Maleate & Hydrochlorothiazide | Enalapril Maleate & Hydrochlorothiazide Tab 10-25 MG |
| 54569859500 | Enalapril Maleate & Hydrochlorothiazide | Enalapril Maleate & Hydrochlorothiazide Tab 10-25 MG |
| 49999082300 | Enalapril Maleate & Hydrochlorothiazide | Enalapril Maleate & Hydrochlorothiazide Tab 10-25 MG |
| 54868550300 | Enalapril Maleate & Hydrochlorothiazide | Enalapril Maleate & Hydrochlorothiazide Tab 5-12.5 MG |
| 54868550301 | Enalapril Maleate & Hydrochlorothiazide | Enalapril Maleate & Hydrochlorothiazide Tab 5-12.5 MG |
| 60505020801 | Enalapril Maleate & Hydrochlorothiazide | Enalapril Maleate & Hydrochlorothiazide Tab 5-12.5 MG |
| 60505020901 | Enalapril Maleate & Hydrochlorothiazide | Enalapril Maleate & Hydrochlorothiazide Tab 10-25 MG |
| 64455014501 | Enalapril Maleate & Hydrochlorothiazide | Enalapril Maleate & Hydrochlorothiazide Tab 5-12.5 MG |
| 64455014601 | Enalapril Maleate & Hydrochlorothiazide | Enalapril Maleate & Hydrochlorothiazide Tab 10-25 MG |
| 00490006700 | Enalapril Maleate & Hydrochlorothiazide | Enalapril Maleate & Hydrochlorothiazide Tab 10-25 MG |
| 00490006730 | Enalapril Maleate & Hydrochlorothiazide | Enalapril Maleate & Hydrochlorothiazide Tab 10-25 MG |
| 00490006760 | Enalapril Maleate & Hydrochlorothiazide | Enalapril Maleate & Hydrochlorothiazide Tab 10-25 MG |
| 00490006790 | Enalapril Maleate & Hydrochlorothiazide | Enalapril Maleate & Hydrochlorothiazide Tab 10-25 MG |
| 00490703000 | Enalapril Maleate & Hydrochlorothiazide | Enalapril Maleate & Hydrochlorothiazide Tab 5-12.5 MG |
| 00490703030 | Enalapril Maleate & Hydrochlorothiazide | Enalapril Maleate & Hydrochlorothiazide Tab 5-12.5 MG |
| 00490703060 | Enalapril Maleate & Hydrochlorothiazide | Enalapril Maleate & Hydrochlorothiazide Tab 5-12.5 MG |
| 00490703090 | Enalapril Maleate & Hydrochlorothiazide | Enalapril Maleate & Hydrochlorothiazide Tab 5-12.5 MG |
| 54868510000 | Enalapril Maleate & Hydrochlorothiazide | Enalapril Maleate & Hydrochlorothiazide Tab 10-25 MG |
| 55111013301 | Enalapril Maleate & Hydrochlorothiazide | Enalapril Maleate & Hydrochlorothiazide Tab 5-12.5 MG |
| 55111013401 | Enalapril Maleate & Hydrochlorothiazide | Enalapril Maleate & Hydrochlorothiazide Tab 10-25 MG |
| 55045337308 | Enalapril Maleate & Hydrochlorothiazide | Enalapril Maleate & Hydrochlorothiazide Tab 5-12.5 MG |
| 55289048430 | Enalapril Maleate & Hydrochlorothiazide | Enalapril Maleate & Hydrochlorothiazide Tab 10-25 MG |
| 55887059430 | Enalapril Maleate & Hydrochlorothiazide | Enalapril Maleate & Hydrochlorothiazide Tab 5-12.5 MG |
| 55887059460 | Enalapril Maleate & Hydrochlorothiazide | Enalapril Maleate & Hydrochlorothiazide Tab 5-12.5 MG |
| 55887059482 | Enalapril Maleate & Hydrochlorothiazide | Enalapril Maleate & Hydrochlorothiazide Tab 5-12.5 MG |
| 55887059490 | Enalapril Maleate & Hydrochlorothiazide | Enalapril Maleate & Hydrochlorothiazide Tab 5-12.5 MG |
| 55887059530 | Enalapril Maleate & Hydrochlorothiazide | Enalapril Maleate & Hydrochlorothiazide Tab 10-25 MG |
| 55887059560 | Enalapril Maleate & Hydrochlorothiazide | Enalapril Maleate & Hydrochlorothiazide Tab 10-25 MG |
| 55887059582 | Enalapril Maleate & Hydrochlorothiazide | Enalapril Maleate & Hydrochlorothiazide Tab 10-25 MG |
| 55887059590 | Enalapril Maleate & Hydrochlorothiazide | Enalapril Maleate & Hydrochlorothiazide Tab 10-25 MG |
| 51079097720 | Enalapril Maleate & Hydrochlorothiazide | Enalapril Maleate & Hydrochlorothiazide Tab 10-25 MG |
| 51672404501 | Enalapril Maleate & Hydrochlorothiazide | Enalapril Maleate & Hydrochlorothiazide Tab 5-12.5 MG |
| 00088176547 | Enalapril Maleate-Diltiazem Malate | Enalapril Maleate-Diltiazem Malate Tab SR 24HR 5-180 MG |
| 00186000168 | Enalapril Maleate-Felodipine | Enalapril Maleate-Felodipine Tab CR 5-5 MG |
| 00186000231 | Enalapril Maleate-Felodipine | Enalapril Maleate-Felodipine Tab CR 5-2.5 MG |
| 61113000128 | Enalapril Maleate-Felodipine | Enalapril Maleate-Felodipine Tab CR 5-5 MG |
| 00186000228 | Enalapril Maleate-Felodipine | Enalapril Maleate-Felodipine Tab CR 5-2.5 MG |
| 61113000168 | Enalapril Maleate-Felodipine | Enalapril Maleate-Felodipine Tab CR 5-5 MG |
| 00186000128 | Enalapril Maleate-Felodipine | Enalapril Maleate-Felodipine Tab CR 5-5 MG |
| 00186000131 | Enalapril Maleate-Felodipine | Enalapril Maleate-Felodipine Tab CR 5-5 MG |
| 61113000131 | Enalapril Maleate-Felodipine | Enalapril Maleate-Felodipine Tab CR 5-5 MG |
| 10019009503 | Enalaprilat | Enalaprilat IV Inj 1.25 MG/ML |
| 10019009504 | Enalaprilat | Enalaprilat IV Inj 1.25 MG/ML |
| 00409212213 | Enalaprilat | Enalaprilat IV Inj 1.25 MG/ML |
| 00409212288 | Enalaprilat | Enalaprilat IV Inj 1.25 MG/ML |
| 00006350801 | Enalaprilat | Enalaprilat IV Inj 1.25 MG/ML |
| 00006350804 | Enalaprilat | Enalaprilat IV Inj 1.25 MG/ML |
| 00006382401 | Enalaprilat | Enalaprilat IV Inj 1.25 MG/ML |
| 00006382404 | Enalaprilat | Enalaprilat IV Inj 1.25 MG/ML |
| 00074210931 | Enalaprilat | Enalaprilat IV Inj 1.25 MG/ML |
| 00074212201 | Enalaprilat | Enalaprilat IV Inj 1.25 MG/ML |
| 00074212202 | Enalaprilat | Enalaprilat IV Inj 1.25 MG/ML |
| 00703840104 | Enalaprilat | Enalaprilat IV Inj 1.25 MG/ML |
| 00703841104 | Enalaprilat | Enalaprilat IV Inj 1.25 MG/ML |
| 00143978610 | Enalaprilat | Enalaprilat IV Inj 1.25 MG/ML |
| 00143978710 | Enalaprilat | Enalaprilat IV Inj 1.25 MG/ML |
| 00409212201 | Enalaprilat | Enalaprilat IV Inj 1.25 MG/ML |
| 00409212202 | Enalaprilat | Enalaprilat IV Inj 1.25 MG/ML |
| 00703840101 | Enalaprilat | Enalaprilat IV Inj 1.25 MG/ML |
| 00703841101 | Enalaprilat | Enalaprilat IV Inj 1.25 MG/ML |
| 61703023716 | Enalaprilat | Enalaprilat IV Inj 1.25 MG/ML |
| 61703023744 | Enalaprilat | Enalaprilat IV Inj 1.25 MG/ML |
| 55390001010 | Enalaprilat | Enalaprilat IV Inj 1.25 MG/ML |
| 55390001110 | Enalaprilat | Enalaprilat IV Inj 1.25 MG/ML |
| 10019009203 | Enalaprilat | Enalaprilat IV Inj 1.25 MG/ML |
| 10019009204 | Enalaprilat | Enalaprilat IV Inj 1.25 MG/ML |
| 59762171003 | Eplerenone | Eplerenone Tab 25 MG |
| 54868606500 | Eplerenone | Eplerenone Tab 25 MG |
| 59762171001 | Eplerenone | Eplerenone Tab 25 MG |
| 00025172001 | Eplerenone | Eplerenone Tab 50 MG |
| 54868505100 | Eplerenone | Eplerenone Tab 50 MG |
| 00025171001 | Eplerenone | Eplerenone Tab 25 MG |
| 21695089190 | Eplerenone | Eplerenone Tab 50 MG |
| 00025172003 | Eplerenone | Eplerenone Tab 50 MG |
| 68115068530 | Eplerenone | Eplerenone Tab 50 MG |
| 54868606501 | Eplerenone | Eplerenone Tab 25 MG |
| 54569612000 | Eplerenone | Eplerenone Tab 50 MG |
| 00025171002 | Eplerenone | Eplerenone Tab 25 MG |
| 00025171003 | Eplerenone | Eplerenone Tab 25 MG |
| 59762172001 | Eplerenone | Eplerenone Tab 50 MG |
| 59762172002 | Eplerenone | Eplerenone Tab 50 MG |
| 60505265103 | Eplerenone | Eplerenone Tab 25 MG |
| 60505265105 | Eplerenone | Eplerenone Tab 25 MG |
| 60505265109 | Eplerenone | Eplerenone Tab 25 MG |
| 60505265203 | Eplerenone | Eplerenone Tab 50 MG |
| 60505265209 | Eplerenone | Eplerenone Tab 50 MG |
| 42254001190 | Eplerenone | Eplerenone Tab 50 MG |
| 00185536809 | Eplerenone | Eplerenone Tab 25 MG |
| 00185536830 | Eplerenone | Eplerenone Tab 25 MG |
| 00185536909 | Eplerenone | Eplerenone Tab 50 MG |
| 00185536930 | Eplerenone | Eplerenone Tab 50 MG |
| 54569583500 | Eplerenone | Eplerenone Tab 25 MG |
| 21695079790 | Eplerenone | Eplerenone Tab 25 MG |
| 59762171002 | Eplerenone | Eplerenone Tab 25 MG |
| 54868472003 | Eprosartan Mesylate | Eprosartan Mesylate Tab 400 MG |
| 54868472002 | Eprosartan Mesylate | Eprosartan Mesylate Tab 400 MG |
| 54868472001 | Eprosartan Mesylate | Eprosartan Mesylate Tab 400 MG |
| 54868472000 | Eprosartan Mesylate | Eprosartan Mesylate Tab 400 MG |
| 64455013101 | Eprosartan Mesylate | Eprosartan Mesylate Tab 600 MG |
| 64455013001 | Eprosartan Mesylate | Eprosartan Mesylate Tab 400 MG |
| 60598010101 | Eprosartan Mesylate | Eprosartan Mesylate Tab 600 MG |
| 60598010001 | Eprosartan Mesylate | Eprosartan Mesylate Tab 400 MG |
| 00051504642 | Eprosartan Mesylate | Eprosartan Mesylate Tab 600 MG |
| 54868546600 | Eprosartan Mesylate | Eprosartan Mesylate Tab 600 MG |
| 00074302511 | Eprosartan Mesylate | Eprosartan Mesylate Tab 400 MG |
| 00074304011 | Eprosartan Mesylate | Eprosartan Mesylate Tab 600 MG |
| 00378662993 | Eprosartan Mesylate | Eprosartan Mesylate Tab 600 MG |
| 54868546601 | Eprosartan Mesylate | Eprosartan Mesylate Tab 600 MG |
| 00051504601 | Eprosartan Mesylate | Eprosartan Mesylate Tab 600 MG |
| 00051504442 | Eprosartan Mesylate | Eprosartan Mesylate Tab 400 MG |
| 00051504401 | Eprosartan Mesylate | Eprosartan Mesylate Tab 400 MG |
| 54868000901 | Eprosartan Mesylate-Hydrochlorothiazide | Eprosartan Mesylate-Hydrochlorothiazide Tab 600-25 MG |
| 64455013201 | Eprosartan Mesylate-Hydrochlorothiazide | Eprosartan Mesylate-Hydrochlorothiazide Tab 600-12.5 MG |
| 60598008101 | Eprosartan Mesylate-Hydrochlorothiazide | Eprosartan Mesylate-Hydrochlorothiazide Tab 600-25 MG |
| 60598008001 | Eprosartan Mesylate-Hydrochlorothiazide | Eprosartan Mesylate-Hydrochlorothiazide Tab 600-12.5 MG |
| 64455013301 | Eprosartan Mesylate-Hydrochlorothiazide | Eprosartan Mesylate-Hydrochlorothiazide Tab 600-25 MG |
| 00074302011 | Eprosartan Mesylate-Hydrochlorothiazide | Eprosartan Mesylate-Hydrochlorothiazide Tab 600-25 MG |
| 54868000900 | Eprosartan Mesylate-Hydrochlorothiazide | Eprosartan Mesylate-Hydrochlorothiazide Tab 600-25 MG |
| 54868528101 | Eprosartan Mesylate-Hydrochlorothiazide | Eprosartan Mesylate-Hydrochlorothiazide Tab 600-12.5 MG |
| 54868528100 | Eprosartan Mesylate-Hydrochlorothiazide | Eprosartan Mesylate-Hydrochlorothiazide Tab 600-12.5 MG |
| 00074301511 | Eprosartan Mesylate-Hydrochlorothiazide | Eprosartan Mesylate-Hydrochlorothiazide Tab 600-12.5 MG |
| 00409337301 | Fenoldopam Mesylate | Fenoldopam Mesylate IV Inj 10 MG/ML (Base Equiv) |
| 00409337302 | Fenoldopam Mesylate | Fenoldopam Mesylate IV Inj 20 MG/2ML (Base Equiv) |
| 00409230401 | Fenoldopam Mesylate | Fenoldopam Mesylate IV Inj 10 MG/ML (Base Equiv) |
| 00781300571 | Fenoldopam Mesylate | Fenoldopam Mesylate IV Inj 10 MG/ML (Base Equiv) |
| 00781300592 | Fenoldopam Mesylate | Fenoldopam Mesylate IV Inj 20 MG/2ML (Base Equiv) |
| 00074230401 | Fenoldopam Mesylate | Fenoldopam Mesylate IV Inj 10 MG/ML (Base Equiv) |
| 62860000401 | Fenoldopam Mesylate | Fenoldopam Mesylate IV Inj 10 MG/ML (Base Equiv) |
| 00409230402 | Fenoldopam Mesylate | Fenoldopam Mesylate IV Inj 20 MG/2ML (Base Equiv) |
| 10019014201 | Fenoldopam Mesylate | Fenoldopam Mesylate IV Inj 10 MG/ML (Base Equiv) |
| 10019014202 | Fenoldopam Mesylate | Fenoldopam Mesylate IV Inj 20 MG/2ML (Base Equiv) |
| 55390007101 | Fenoldopam Mesylate | Fenoldopam Mesylate IV Inj 10 MG/ML (Base Equiv) |
| 55390007201 | Fenoldopam Mesylate | Fenoldopam Mesylate IV Inj 20 MG/2ML (Base Equiv) |
| 62860000202 | Fenoldopam Mesylate | Fenoldopam Mesylate IV Inj 10 MG/ML (Base Equiv) |
| 00074230402 | Fenoldopam Mesylate | Fenoldopam Mesylate IV Inj 20 MG/2ML (Base Equiv) |
| 67544110545 | Fosinopril Sodium | Fosinopril Sodium Tab 10 MG |
| 67544110530 | Fosinopril Sodium | Fosinopril Sodium Tab 10 MG |
| 23629002110 | Fosinopril Sodium | Fosinopril Sodium Tab 10 MG |
| 00440755290 | Fosinopril Sodium | Fosinopril Sodium Tab 40 MG |
| 00440755292 | Fosinopril Sodium | Fosinopril Sodium Tab 40 MG |
| 54569609800 | Fosinopril Sodium | Fosinopril Sodium Tab 40 MG |
| 60429075510 | Fosinopril Sodium | Fosinopril Sodium Tab 10 MG |
| 60429075545 | Fosinopril Sodium | Fosinopril Sodium Tab 10 MG |
| 60429075590 | Fosinopril Sodium | Fosinopril Sodium Tab 10 MG |
| 60429075610 | Fosinopril Sodium | Fosinopril Sodium Tab 20 MG |
| 60429075618 | Fosinopril Sodium | Fosinopril Sodium Tab 20 MG |
| 60429075645 | Fosinopril Sodium | Fosinopril Sodium Tab 20 MG |
| 60429075690 | Fosinopril Sodium | Fosinopril Sodium Tab 20 MG |
| 60429075710 | Fosinopril Sodium | Fosinopril Sodium Tab 40 MG |
| 60429075718 | Fosinopril Sodium | Fosinopril Sodium Tab 40 MG |
| 60429075745 | Fosinopril Sodium | Fosinopril Sodium Tab 40 MG |
| 60429075790 | Fosinopril Sodium | Fosinopril Sodium Tab 40 MG |
| 31722020010 | Fosinopril Sodium | Fosinopril Sodium Tab 10 MG |
| 31722020090 | Fosinopril Sodium | Fosinopril Sodium Tab 10 MG |
| 31722020110 | Fosinopril Sodium | Fosinopril Sodium Tab 20 MG |
| 31722020190 | Fosinopril Sodium | Fosinopril Sodium Tab 20 MG |
| 31722020210 | Fosinopril Sodium | Fosinopril Sodium Tab 40 MG |
| 31722020290 | Fosinopril Sodium | Fosinopril Sodium Tab 40 MG |
| 51138052030 | Fosinopril Sodium | Fosinopril Sodium Tab 10 MG |
| 51138052130 | Fosinopril Sodium | Fosinopril Sodium Tab 20 MG |
| 51138052230 | Fosinopril Sodium | Fosinopril Sodium Tab 40 MG |
| 65862047190 | Fosinopril Sodium | Fosinopril Sodium Tab 10 MG |
| 65862047290 | Fosinopril Sodium | Fosinopril Sodium Tab 20 MG |
| 65862047390 | Fosinopril Sodium | Fosinopril Sodium Tab 40 MG |
| 76282020010 | Fosinopril Sodium | Fosinopril Sodium Tab 10 MG |
| 76282020090 | Fosinopril Sodium | Fosinopril Sodium Tab 10 MG |
| 76282020110 | Fosinopril Sodium | Fosinopril Sodium Tab 20 MG |
| 76282020190 | Fosinopril Sodium | Fosinopril Sodium Tab 20 MG |
| 76282020210 | Fosinopril Sodium | Fosinopril Sodium Tab 40 MG |
| 76282020290 | Fosinopril Sodium | Fosinopril Sodium Tab 40 MG |
| 55048028330 | Fosinopril Sodium | Fosinopril Sodium Tab 40 MG |
| 55048029130 | Fosinopril Sodium | Fosinopril Sodium Tab 20 MG |
| 67544020280 | Fosinopril Sodium | Fosinopril Sodium Tab 10 MG |
| 67544043130 | Fosinopril Sodium | Fosinopril Sodium Tab 20 MG |
| 68552022110 | Fosinopril Sodium | Fosinopril Sodium Tab 10 MG |
| 68552022190 | Fosinopril Sodium | Fosinopril Sodium Tab 10 MG |
| 68552022210 | Fosinopril Sodium | Fosinopril Sodium Tab 20 MG |
| 68552022290 | Fosinopril Sodium | Fosinopril Sodium Tab 20 MG |
| 68552022310 | Fosinopril Sodium | Fosinopril Sodium Tab 40 MG |
| 68552022390 | Fosinopril Sodium | Fosinopril Sodium Tab 40 MG |
| 67544043115 | Fosinopril Sodium | Fosinopril Sodium Tab 20 MG |
| 67544043145 | Fosinopril Sodium | Fosinopril Sodium Tab 20 MG |
| 67544043153 | Fosinopril Sodium | Fosinopril Sodium Tab 20 MG |
| 67544043160 | Fosinopril Sodium | Fosinopril Sodium Tab 20 MG |
| 67544043170 | Fosinopril Sodium | Fosinopril Sodium Tab 20 MG |
| 67544043173 | Fosinopril Sodium | Fosinopril Sodium Tab 20 MG |
| 67544043180 | Fosinopril Sodium | Fosinopril Sodium Tab 20 MG |
| 67544043192 | Fosinopril Sodium | Fosinopril Sodium Tab 20 MG |
| 67544043194 | Fosinopril Sodium | Fosinopril Sodium Tab 20 MG |
| 67544045415 | Fosinopril Sodium | Fosinopril Sodium Tab 40 MG |
| 67544045430 | Fosinopril Sodium | Fosinopril Sodium Tab 40 MG |
| 67544045440 | Fosinopril Sodium | Fosinopril Sodium Tab 40 MG |
| 67544045445 | Fosinopril Sodium | Fosinopril Sodium Tab 40 MG |
| 67544045460 | Fosinopril Sodium | Fosinopril Sodium Tab 40 MG |
| 67544048915 | Fosinopril Sodium | Fosinopril Sodium Tab 10 MG |
| 67544048930 | Fosinopril Sodium | Fosinopril Sodium Tab 10 MG |
| 67544048960 | Fosinopril Sodium | Fosinopril Sodium Tab 10 MG |
| 67544038030 | Fosinopril Sodium | Fosinopril Sodium Tab 10 MG |
| 67544038060 | Fosinopril Sodium | Fosinopril Sodium Tab 10 MG |
| 67544038115 | Fosinopril Sodium | Fosinopril Sodium Tab 20 MG |
| 67544038130 | Fosinopril Sodium | Fosinopril Sodium Tab 20 MG |
| 67544038145 | Fosinopril Sodium | Fosinopril Sodium Tab 20 MG |
| 67544038153 | Fosinopril Sodium | Fosinopril Sodium Tab 20 MG |
| 67544038160 | Fosinopril Sodium | Fosinopril Sodium Tab 20 MG |
| 67544038170 | Fosinopril Sodium | Fosinopril Sodium Tab 20 MG |
| 67544038173 | Fosinopril Sodium | Fosinopril Sodium Tab 20 MG |
| 67544038180 | Fosinopril Sodium | Fosinopril Sodium Tab 20 MG |
| 67544038192 | Fosinopril Sodium | Fosinopril Sodium Tab 20 MG |
| 67544038194 | Fosinopril Sodium | Fosinopril Sodium Tab 20 MG |
| 54868518202 | Fosinopril Sodium | Fosinopril Sodium Tab 40 MG |
| 67544020215 | Fosinopril Sodium | Fosinopril Sodium Tab 10 MG |
| 67544030015 | Fosinopril Sodium | Fosinopril Sodium Tab 40 MG |
| 67544103830 | Fosinopril Sodium | Fosinopril Sodium Tab 40 MG |
| 67544103845 | Fosinopril Sodium | Fosinopril Sodium Tab 40 MG |
| 00087060941 | Fosinopril Sodium | Fosinopril Sodium Tab 20 MG |
| 00087060950 | Fosinopril Sodium | Fosinopril Sodium Tab 20 MG |
| 00087060951 | Fosinopril Sodium | Fosinopril Sodium Tab 20 MG |
| 00087120212 | Fosinopril Sodium | Fosinopril Sodium Tab 40 MG |
| 00087015822 | Fosinopril Sodium | Fosinopril Sodium Tab 10 MG |
| 00087015845 | Fosinopril Sodium | Fosinopril Sodium Tab 10 MG |
| 00087015850 | Fosinopril Sodium | Fosinopril Sodium Tab 10 MG |
| 00087015851 | Fosinopril Sodium | Fosinopril Sodium Tab 10 MG |
| 54569380801 | Fosinopril Sodium | Fosinopril Sodium Tab 10 MG |
| 54569859200 | Fosinopril Sodium | Fosinopril Sodium Tab 10 MG |
| 67544104260 | Fosinopril Sodium | Fosinopril Sodium Tab 40 MG |
| 57866027401 | Fosinopril Sodium | Fosinopril Sodium Tab 40 MG |
| 57866027502 | Fosinopril Sodium | Fosinopril Sodium Tab 10 MG |
| 00440755045 | Fosinopril Sodium | Fosinopril Sodium Tab 10 MG |
| 00440755090 | Fosinopril Sodium | Fosinopril Sodium Tab 10 MG |
| 00440755145 | Fosinopril Sodium | Fosinopril Sodium Tab 20 MG |
| 00440755190 | Fosinopril Sodium | Fosinopril Sodium Tab 20 MG |
| 00440755245 | Fosinopril Sodium | Fosinopril Sodium Tab 40 MG |
| 67544104245 | Fosinopril Sodium | Fosinopril Sodium Tab 40 MG |
| 67544108245 | Fosinopril Sodium | Fosinopril Sodium Tab 20 MG |
| 63304077690 | Fosinopril Sodium | Fosinopril Sodium Tab 20 MG |
| 63304077710 | Fosinopril Sodium | Fosinopril Sodium Tab 40 MG |
| 63304077790 | Fosinopril Sodium | Fosinopril Sodium Tab 40 MG |
| 68462036710 | Fosinopril Sodium | Fosinopril Sodium Tab 10 MG |
| 68462036790 | Fosinopril Sodium | Fosinopril Sodium Tab 10 MG |
| 68462036810 | Fosinopril Sodium | Fosinopril Sodium Tab 20 MG |
| 68462036890 | Fosinopril Sodium | Fosinopril Sodium Tab 20 MG |
| 68462036910 | Fosinopril Sodium | Fosinopril Sodium Tab 40 MG |
| 68462036990 | Fosinopril Sodium | Fosinopril Sodium Tab 40 MG |
| 24658010010 | Fosinopril Sodium | Fosinopril Sodium Tab 10 MG |
| 24658010090 | Fosinopril Sodium | Fosinopril Sodium Tab 10 MG |
| 24658010110 | Fosinopril Sodium | Fosinopril Sodium Tab 20 MG |
| 24658010190 | Fosinopril Sodium | Fosinopril Sodium Tab 20 MG |
| 24658010210 | Fosinopril Sodium | Fosinopril Sodium Tab 40 MG |
| 24658010290 | Fosinopril Sodium | Fosinopril Sodium Tab 40 MG |
| 62037093590 | Fosinopril Sodium | Fosinopril Sodium Tab 10 MG |
| 62037093690 | Fosinopril Sodium | Fosinopril Sodium Tab 20 MG |
| 62037093790 | Fosinopril Sodium | Fosinopril Sodium Tab 40 MG |
| 60505251004 | Fosinopril Sodium | Fosinopril Sodium Tab 10 MG |
| 60505251104 | Fosinopril Sodium | Fosinopril Sodium Tab 20 MG |
| 60505251208 | Fosinopril Sodium | Fosinopril Sodium Tab 40 MG |
| 67544030030 | Fosinopril Sodium | Fosinopril Sodium Tab 40 MG |
| 67544030045 | Fosinopril Sodium | Fosinopril Sodium Tab 40 MG |
| 67544030630 | Fosinopril Sodium | Fosinopril Sodium Tab 40 MG |
| 67544030640 | Fosinopril Sodium | Fosinopril Sodium Tab 40 MG |
| 67544030645 | Fosinopril Sodium | Fosinopril Sodium Tab 40 MG |
| 67544030660 | Fosinopril Sodium | Fosinopril Sodium Tab 40 MG |
| 67544031130 | Fosinopril Sodium | Fosinopril Sodium Tab 40 MG |
| 67544031145 | Fosinopril Sodium | Fosinopril Sodium Tab 40 MG |
| 67544032115 | Fosinopril Sodium | Fosinopril Sodium Tab 10 MG |
| 67544032130 | Fosinopril Sodium | Fosinopril Sodium Tab 10 MG |
| 67544032160 | Fosinopril Sodium | Fosinopril Sodium Tab 10 MG |
| 67544032215 | Fosinopril Sodium | Fosinopril Sodium Tab 20 MG |
| 67544032230 | Fosinopril Sodium | Fosinopril Sodium Tab 20 MG |
| 67544032245 | Fosinopril Sodium | Fosinopril Sodium Tab 20 MG |
| 67544032253 | Fosinopril Sodium | Fosinopril Sodium Tab 20 MG |
| 67544032260 | Fosinopril Sodium | Fosinopril Sodium Tab 20 MG |
| 67544032270 | Fosinopril Sodium | Fosinopril Sodium Tab 20 MG |
| 67544032273 | Fosinopril Sodium | Fosinopril Sodium Tab 20 MG |
| 67544032280 | Fosinopril Sodium | Fosinopril Sodium Tab 20 MG |
| 67544032292 | Fosinopril Sodium | Fosinopril Sodium Tab 20 MG |
| 67544032294 | Fosinopril Sodium | Fosinopril Sodium Tab 20 MG |
| 67544004215 | Fosinopril Sodium | Fosinopril Sodium Tab 20 MG |
| 67544004230 | Fosinopril Sodium | Fosinopril Sodium Tab 20 MG |
| 67544004245 | Fosinopril Sodium | Fosinopril Sodium Tab 20 MG |
| 67544004253 | Fosinopril Sodium | Fosinopril Sodium Tab 20 MG |
| 67544004260 | Fosinopril Sodium | Fosinopril Sodium Tab 20 MG |
| 67544004270 | Fosinopril Sodium | Fosinopril Sodium Tab 20 MG |
| 67544004273 | Fosinopril Sodium | Fosinopril Sodium Tab 20 MG |
| 67544004280 | Fosinopril Sodium | Fosinopril Sodium Tab 20 MG |
| 67544004292 | Fosinopril Sodium | Fosinopril Sodium Tab 20 MG |
| 67544004294 | Fosinopril Sodium | Fosinopril Sodium Tab 20 MG |
| 67544020145 | Fosinopril Sodium | Fosinopril Sodium Tab 40 MG |
| 67544020230 | Fosinopril Sodium | Fosinopril Sodium Tab 10 MG |
| 67544020245 | Fosinopril Sodium | Fosinopril Sodium Tab 10 MG |
| 00087060942 | Fosinopril Sodium | Fosinopril Sodium Tab 20 MG |
| 00087060945 | Fosinopril Sodium | Fosinopril Sodium Tab 20 MG |
| 00087060985 | Fosinopril Sodium | Fosinopril Sodium Tab 20 MG |
| 00093722210 | Fosinopril Sodium | Fosinopril Sodium Tab 10 MG |
| 00093722298 | Fosinopril Sodium | Fosinopril Sodium Tab 10 MG |
| 00093722310 | Fosinopril Sodium | Fosinopril Sodium Tab 20 MG |
| 00093722398 | Fosinopril Sodium | Fosinopril Sodium Tab 20 MG |
| 00093722410 | Fosinopril Sodium | Fosinopril Sodium Tab 40 MG |
| 00093722498 | Fosinopril Sodium | Fosinopril Sodium Tab 40 MG |
| 00087120213 | Fosinopril Sodium | Fosinopril Sodium Tab 40 MG |
| 00087015846 | Fosinopril Sodium | Fosinopril Sodium Tab 10 MG |
| 00087015885 | Fosinopril Sodium | Fosinopril Sodium Tab 10 MG |
| 00185004109 | Fosinopril Sodium | Fosinopril Sodium Tab 10 MG |
| 00185004110 | Fosinopril Sodium | Fosinopril Sodium Tab 10 MG |
| 00185004209 | Fosinopril Sodium | Fosinopril Sodium Tab 20 MG |
| 00185004210 | Fosinopril Sodium | Fosinopril Sodium Tab 20 MG |
| 00185004709 | Fosinopril Sodium | Fosinopril Sodium Tab 40 MG |
| 00185004710 | Fosinopril Sodium | Fosinopril Sodium Tab 40 MG |
| 00781508310 | Fosinopril Sodium | Fosinopril Sodium Tab 10 MG |
| 00781508392 | Fosinopril Sodium | Fosinopril Sodium Tab 10 MG |
| 00781508410 | Fosinopril Sodium | Fosinopril Sodium Tab 20 MG |
| 00781508492 | Fosinopril Sodium | Fosinopril Sodium Tab 20 MG |
| 00781508592 | Fosinopril Sodium | Fosinopril Sodium Tab 40 MG |
| 54569380800 | Fosinopril Sodium | Fosinopril Sodium Tab 10 MG |
| 54569380900 | Fosinopril Sodium | Fosinopril Sodium Tab 20 MG |
| 54569562100 | Fosinopril Sodium | Fosinopril Sodium Tab 10 MG |
| 54569494800 | Fosinopril Sodium | Fosinopril Sodium Tab 40 MG |
| 54868236800 | Fosinopril Sodium | Fosinopril Sodium Tab 10 MG |
| 54868236801 | Fosinopril Sodium | Fosinopril Sodium Tab 10 MG |
| 54868236802 | Fosinopril Sodium | Fosinopril Sodium Tab 10 MG |
| 54868327900 | Fosinopril Sodium | Fosinopril Sodium Tab 20 MG |
| 54868327902 | Fosinopril Sodium | Fosinopril Sodium Tab 20 MG |
| 54868327903 | Fosinopril Sodium | Fosinopril Sodium Tab 20 MG |
| 54868420900 | Fosinopril Sodium | Fosinopril Sodium Tab 40 MG |
| 54868505500 | Fosinopril Sodium | Fosinopril Sodium Tab 20 MG |
| 54868506400 | Fosinopril Sodium | Fosinopril Sodium Tab 10 MG |
| 54868506401 | Fosinopril Sodium | Fosinopril Sodium Tab 10 MG |
| 54868518200 | Fosinopril Sodium | Fosinopril Sodium Tab 40 MG |
| 54868518201 | Fosinopril Sodium | Fosinopril Sodium Tab 40 MG |
| 57866313801 | Fosinopril Sodium | Fosinopril Sodium Tab 20 MG |
| 58864010430 | Fosinopril Sodium | Fosinopril Sodium Tab 10 MG |
| 58864051015 | Fosinopril Sodium | Fosinopril Sodium Tab 40 MG |
| 58864066730 | Fosinopril Sodium | Fosinopril Sodium Tab 20 MG |
| 58864077430 | Fosinopril Sodium | Fosinopril Sodium Tab 10 MG |
| 58864077490 | Fosinopril Sodium | Fosinopril Sodium Tab 10 MG |
| 60505251002 | Fosinopril Sodium | Fosinopril Sodium Tab 10 MG |
| 60505251102 | Fosinopril Sodium | Fosinopril Sodium Tab 20 MG |
| 60505251202 | Fosinopril Sodium | Fosinopril Sodium Tab 40 MG |
| 63304077510 | Fosinopril Sodium | Fosinopril Sodium Tab 10 MG |
| 63304077590 | Fosinopril Sodium | Fosinopril Sodium Tab 10 MG |
| 63304077610 | Fosinopril Sodium | Fosinopril Sodium Tab 20 MG |
| 65862030801 | Fosinopril Sodium & Hydrochlorothiazide | Fosinopril Sodium & Hydrochlorothiazide Tab 10-12.5 MG |
| 63304040401 | Fosinopril Sodium & Hydrochlorothiazide | Fosinopril Sodium & Hydrochlorothiazide Tab 20-12.5 MG |
| 68462055401 | Fosinopril Sodium & Hydrochlorothiazide | Fosinopril Sodium & Hydrochlorothiazide Tab 10-12.5 MG |
| 68462055501 | Fosinopril Sodium & Hydrochlorothiazide | Fosinopril Sodium & Hydrochlorothiazide Tab 20-12.5 MG |
| 59762525001 | Fosinopril Sodium & Hydrochlorothiazide | Fosinopril Sodium & Hydrochlorothiazide Tab 10-12.5 MG |
| 59762525004 | Fosinopril Sodium & Hydrochlorothiazide | Fosinopril Sodium & Hydrochlorothiazide Tab 10-12.5 MG |
| 59762525101 | Fosinopril Sodium & Hydrochlorothiazide | Fosinopril Sodium & Hydrochlorothiazide Tab 20-12.5 MG |
| 59762525104 | Fosinopril Sodium & Hydrochlorothiazide | Fosinopril Sodium & Hydrochlorothiazide Tab 20-12.5 MG |
| 23155006001 | Fosinopril Sodium & Hydrochlorothiazide | Fosinopril Sodium & Hydrochlorothiazide Tab 10-12.5 MG |
| 23155006101 | Fosinopril Sodium & Hydrochlorothiazide | Fosinopril Sodium & Hydrochlorothiazide Tab 20-12.5 MG |
| 00185034101 | Fosinopril Sodium & Hydrochlorothiazide | Fosinopril Sodium & Hydrochlorothiazide Tab 10-12.5 MG |
| 00185034201 | Fosinopril Sodium & Hydrochlorothiazide | Fosinopril Sodium & Hydrochlorothiazide Tab 20-12.5 MG |
| 54868534600 | Fosinopril Sodium & Hydrochlorothiazide | Fosinopril Sodium & Hydrochlorothiazide Tab 10-12.5 MG |
| 54868534601 | Fosinopril Sodium & Hydrochlorothiazide | Fosinopril Sodium & Hydrochlorothiazide Tab 10-12.5 MG |
| 54868546900 | Fosinopril Sodium & Hydrochlorothiazide | Fosinopril Sodium & Hydrochlorothiazide Tab 20-12.5 MG |
| 54868546901 | Fosinopril Sodium & Hydrochlorothiazide | Fosinopril Sodium & Hydrochlorothiazide Tab 20-12.5 MG |
| 00087149201 | Fosinopril Sodium & Hydrochlorothiazide | Fosinopril Sodium & Hydrochlorothiazide Tab 10-12.5 MG |
| 00087149301 | Fosinopril Sodium & Hydrochlorothiazide | Fosinopril Sodium & Hydrochlorothiazide Tab 20-12.5 MG |
| 63304040301 | Fosinopril Sodium & Hydrochlorothiazide | Fosinopril Sodium & Hydrochlorothiazide Tab 10-12.5 MG |
| 65862030901 | Fosinopril Sodium & Hydrochlorothiazide | Fosinopril Sodium & Hydrochlorothiazide Tab 20-12.5 MG |
| 00536568701 | Guanabenz Acetate | Guanabenz Acetate Tab 4 MG |
| 00182195201 | Guanabenz Acetate | Guanabenz Acetate Tab 8 MG |
| 00536568801 | Guanabenz Acetate | Guanabenz Acetate Tab 8 MG |
| 00182195101 | Guanabenz Acetate | Guanabenz Acetate Tab 4 MG |
| 00172422770 | Guanabenz Acetate | Guanabenz Acetate Tab 8 MG |
| 00172422670 | Guanabenz Acetate | Guanabenz Acetate Tab 4 MG |
| 00047056124 | Guanabenz Acetate | Guanabenz Acetate Tab 8 MG |
| 00047056024 | Guanabenz Acetate | Guanabenz Acetate Tab 4 MG |
| 00008009201 | Guanabenz Acetate | Guanabenz Acetate Tab 16 MG |
| 00008007305 | Guanabenz Acetate | Guanabenz Acetate Tab 4 MG |
| 00008007304 | Guanabenz Acetate | Guanabenz Acetate Tab 4 MG |
| 00008007303 | Guanabenz Acetate | Guanabenz Acetate Tab 4 MG |
| 00008007301 | Guanabenz Acetate | Guanabenz Acetate Tab 4 MG |
| 52544045201 | Guanabenz Acetate | Guanabenz Acetate Tab 8 MG |
| 00603377921 | Guanabenz Acetate | Guanabenz Acetate Tab 4 MG |
| 00603378021 | Guanabenz Acetate | Guanabenz Acetate Tab 8 MG |
| 00839793206 | Guanabenz Acetate | Guanabenz Acetate Tab 4 MG |
| 00839793306 | Guanabenz Acetate | Guanabenz Acetate Tab 8 MG |
| 38245071110 | Guanabenz Acetate | Guanabenz Acetate Tab 8 MG |
| 38245071710 | Guanabenz Acetate | Guanabenz Acetate Tab 4 MG |
| 38245071750 | Guanabenz Acetate | Guanabenz Acetate Tab 4 MG |
| 52555055501 | Guanabenz Acetate | Guanabenz Acetate Tab 4 MG |
| 52555055601 | Guanabenz Acetate | Guanabenz Acetate Tab 8 MG |
| 54124094360 | Guanabenz Acetate | Guanabenz Acetate Tab 4 MG |
| 54124094760 | Guanabenz Acetate | Guanabenz Acetate Tab 8 MG |
| 54569061000 | Guanabenz Acetate | Guanabenz Acetate Tab 4 MG |
| 54569061100 | Guanabenz Acetate | Guanabenz Acetate Tab 8 MG |
| 00008007401 | Guanabenz Acetate | Guanabenz Acetate Tab 8 MG |
| 00172422660 | Guanabenz Acetate | Guanabenz Acetate Tab 4 MG |
| 00172422760 | Guanabenz Acetate | Guanabenz Acetate Tab 8 MG |
| 52544045101 | Guanabenz Acetate | Guanabenz Acetate Tab 4 MG |
| 00018078771 | Guanadrel Sulfate | Guanadrel Sulfate Tab 10 MG |
| 54569171000 | Guanadrel Sulfate | Guanadrel Sulfate Tab 25 MG |
| 53014078871 | Guanadrel Sulfate | Guanadrel Sulfate Tab 25 MG |
| 54569062201 | Guanadrel Sulfate | Guanadrel Sulfate Tab 10 MG |
| 54569062200 | Guanadrel Sulfate | Guanadrel Sulfate Tab 10 MG |
| 00585078771 | Guanadrel Sulfate | Guanadrel Sulfate Tab 10 MG |
| 54124053260 | Guanadrel Sulfate | Guanadrel Sulfate Tab 10 MG |
| 00018078871 | Guanadrel Sulfate | Guanadrel Sulfate Tab 25 MG |
| 53014078771 | Guanadrel Sulfate | Guanadrel Sulfate Tab 10 MG |
| 54124082960 | Guanadrel Sulfate | Guanadrel Sulfate Tab 25 MG |
| 00585078871 | Guanadrel Sulfate | Guanadrel Sulfate Tab 25 MG |
| 54124053290 | Guanadrel Sulfate | Guanadrel Sulfate Tab 10 MG |
| 00536387201 | Guanethidine Monosulfate | Guanethidine Monosulfate Tab 10 MG |
| 00536387301 | Guanethidine Monosulfate | Guanethidine Monosulfate Tab 25 MG |
| 00719136810 | Guanethidine Monosulfate | Guanethidine Monosulfate Tab 10 MG |
| 00719136910 | Guanethidine Monosulfate | Guanethidine Monosulfate Tab 25 MG |
| 00725013501 | Guanethidine Monosulfate | Guanethidine Monosulfate Tab 10 MG |
| 00725013505 | Guanethidine Monosulfate | Guanethidine Monosulfate Tab 10 MG |
| 00725013601 | Guanethidine Monosulfate | Guanethidine Monosulfate Tab 25 MG |
| 00725013605 | Guanethidine Monosulfate | Guanethidine Monosulfate Tab 25 MG |
| 00814360014 | Guanethidine Monosulfate | Guanethidine Monosulfate Tab 10 MG |
| 00814360114 | Guanethidine Monosulfate | Guanethidine Monosulfate Tab 25 MG |
| 00839708906 | Guanethidine Monosulfate | Guanethidine Monosulfate Tab 10 MG |
| 00839709006 | Guanethidine Monosulfate | Guanethidine Monosulfate Tab 25 MG |
| 00904249460 | Guanethidine Monosulfate | Guanethidine Monosulfate Tab 25 MG |
| 00904249560 | Guanethidine Monosulfate | Guanethidine Monosulfate Tab 10 MG |
| 17236057701 | Guanethidine Monosulfate | Guanethidine Monosulfate Tab 10 MG |
| 17236057801 | Guanethidine Monosulfate | Guanethidine Monosulfate Tab 25 MG |
| 47202253201 | Guanethidine Monosulfate | Guanethidine Monosulfate Tab 10 MG |
| 47202253301 | Guanethidine Monosulfate | Guanethidine Monosulfate Tab 25 MG |
| 51432019203 | Guanethidine Monosulfate | Guanethidine Monosulfate Tab 25 MG |
| 51432019303 | Guanethidine Monosulfate | Guanethidine Monosulfate Tab 10 MG |
| 55175246100 | Guanethidine Monosulfate | Guanethidine Monosulfate Tab 10 MG |
| 55175247500 | Guanethidine Monosulfate | Guanethidine Monosulfate Tab 25 MG |
| 00083004930 | Guanethidine Monosulfate | Guanethidine Monosulfate Tab 10 MG |
| 00083010330 | Guanethidine Monosulfate | Guanethidine Monosulfate Tab 25 MG |
| 00302322001 | Guanethidine Monosulfate | Guanethidine Monosulfate Tab 10 MG |
| 00302322201 | Guanethidine Monosulfate | Guanethidine Monosulfate Tab 25 MG |
| 00304189001 | Guanethidine Monosulfate | Guanethidine Monosulfate Tab 10 MG |
| 00304140801 | Guanethidine Monosulfate | Guanethidine Monosulfate Tab 25 MG |
| 00364074101 | Guanethidine Monosulfate | Guanethidine Monosulfate Tab 10 MG |
| 00364074201 | Guanethidine Monosulfate | Guanethidine Monosulfate Tab 25 MG |
| 35356016190 | Guanfacine HCl | Guanfacine HCl Tab 1 MG |
| 00182264201 | Guanfacine HCl | Guanfacine HCl Tab 2 MG |
| 65162071110 | Guanfacine HCl | Guanfacine HCl Tab 1 MG |
| 65162071310 | Guanfacine HCl | Guanfacine HCl Tab 2 MG |
| 00904618360 | Guanfacine HCl | Guanfacine HCl Tab 1 MG |
| 00904618460 | Guanfacine HCl | Guanfacine HCl Tab 2 MG |
| 00536575601 | Guanfacine HCl | Guanfacine HCl Tab 1 MG |
| 00536575701 | Guanfacine HCl | Guanfacine HCl Tab 2 MG |
| 00781136601 | Guanfacine HCl | Guanfacine HCl Tab 1 MG |
| 00781137301 | Guanfacine HCl | Guanfacine HCl Tab 2 MG |
| 00839804606 | Guanfacine HCl | Guanfacine HCl Tab 1 MG |
| 00839804706 | Guanfacine HCl | Guanfacine HCl Tab 2 MG |
| 40893068130 | Guanfacine HCl | Guanfacine HCl Tab 1 MG |
| 52493068130 | Guanfacine HCl | Guanfacine HCl Tab 1 MG |
| 53506072230 | Guanfacine HCl | Guanfacine HCl Tab 1 MG |
| 54124059830 | Guanfacine HCl | Guanfacine HCl Tab 1 MG |
| 54569060200 | Guanfacine HCl | Guanfacine HCl Tab 1 MG |
| 54569060201 | Guanfacine HCl | Guanfacine HCl Tab 1 MG |
| 54569060202 | Guanfacine HCl | Guanfacine HCl Tab 1 MG |
| 54569352600 | Guanfacine HCl | Guanfacine HCl Tab 2 MG |
| 54569856300 | Guanfacine HCl | Guanfacine HCl Tab 1 MG |
| 23490795203 | Guanfacine HCl | Guanfacine HCl Tab 1 MG |
| 23629002510 | Guanfacine HCl | Guanfacine HCl Tab 1 MG |
| 35356016130 | Guanfacine HCl | Guanfacine HCl Tab 1 MG |
| 00031890163 | Guanfacine HCl | Guanfacine HCl Tab 1 MG |
| 00031890170 | Guanfacine HCl | Guanfacine HCl Tab 1 MG |
| 00031890363 | Guanfacine HCl | Guanfacine HCl Tab 2 MG |
| 00378116001 | Guanfacine HCl | Guanfacine HCl Tab 1 MG |
| 00378119001 | Guanfacine HCl | Guanfacine HCl Tab 2 MG |
| 00591044401 | Guanfacine HCl | Guanfacine HCl Tab 1 MG |
| 00591045301 | Guanfacine HCl | Guanfacine HCl Tab 2 MG |
| 00603377421 | Guanfacine HCl | Guanfacine HCl Tab 1 MG |
| 00603377521 | Guanfacine HCl | Guanfacine HCl Tab 2 MG |
| 00904557960 | Guanfacine HCl | Guanfacine HCl Tab 1 MG |
| 00904558060 | Guanfacine HCl | Guanfacine HCl Tab 2 MG |
| 00904513360 | Guanfacine HCl | Guanfacine HCl Tab 1 MG |
| 00904513460 | Guanfacine HCl | Guanfacine HCl Tab 2 MG |
| 49884057201 | Guanfacine HCl | Guanfacine HCl Tab 1 MG |
| 49884057301 | Guanfacine HCl | Guanfacine HCl Tab 2 MG |
| 52544044401 | Guanfacine HCl | Guanfacine HCl Tab 1 MG |
| 52544045301 | Guanfacine HCl | Guanfacine HCl Tab 2 MG |
| 52152011802 | Guanfacine HCl | Guanfacine HCl Tab 1 MG |
| 52152011804 | Guanfacine HCl | Guanfacine HCl Tab 1 MG |
| 52152011902 | Guanfacine HCl | Guanfacine HCl Tab 2 MG |
| 52152011904 | Guanfacine HCl | Guanfacine HCl Tab 2 MG |
| 54868487600 | Guanfacine HCl | Guanfacine HCl Tab 2 MG |
| 54868487601 | Guanfacine HCl | Guanfacine HCl Tab 2 MG |
| 54868487602 | Guanfacine HCl | Guanfacine HCl Tab 2 MG |
| 59911584001 | Guanfacine HCl | Guanfacine HCl Tab 1 MG |
| 59911584101 | Guanfacine HCl | Guanfacine HCl Tab 2 MG |
| 67286890101 | Guanfacine HCl | Guanfacine HCl Tab 1 MG |
| 67286890105 | Guanfacine HCl | Guanfacine HCl Tab 1 MG |
| 67286890301 | Guanfacine HCl | Guanfacine HCl Tab 2 MG |
| 67857070501 | Guanfacine HCl | Guanfacine HCl Tab 1 MG |
| 67857070505 | Guanfacine HCl | Guanfacine HCl Tab 1 MG |
| 67857070601 | Guanfacine HCl | Guanfacine HCl Tab 2 MG |
| 00031890164 | Guanfacine HCl | Guanfacine HCl Tab 1 MG |
| 00047031224 | Guanfacine HCl | Guanfacine HCl Tab 1 MG |
| 00047031324 | Guanfacine HCl | Guanfacine HCl Tab 2 MG |
| 00182264101 | Guanfacine HCl | Guanfacine HCl Tab 1 MG |
| 52959094500 | Guanfacine HCl | Guanfacine HCl Tab 1 MG |
| 00228219810 | Hydralazine & Hydrochlorothiazide | Hydralazine & Hydrochlorothiazide Cap 50-50 MG |
| 00302328801 | Hydralazine & Hydrochlorothiazide | Hydralazine & Hydrochlorothiazide Cap 25-25 MG |
| 00302328805 | Hydralazine & Hydrochlorothiazide | Hydralazine & Hydrochlorothiazide Cap 25-25 MG |
| 00302329001 | Hydralazine & Hydrochlorothiazide | Hydralazine & Hydrochlorothiazide Cap 50-50 MG |
| 00304003801 | Hydralazine & Hydrochlorothiazide | Hydralazine & Hydrochlorothiazide Tab 25-15 MG |
| 00047086824 | Hydralazine & Hydrochlorothiazide | Hydralazine & Hydrochlorothiazide Cap 25-25 MG |
| 00047087124 | Hydralazine & Hydrochlorothiazide | Hydralazine & Hydrochlorothiazide Cap 50-50 MG |
| 00083012930 | Hydralazine & Hydrochlorothiazide | Hydralazine & Hydrochlorothiazide Tab 25-15 MG |
| 00083013930 | Hydralazine & Hydrochlorothiazide | Hydralazine & Hydrochlorothiazide Cap 25-25 MG |
| 00083014930 | Hydralazine & Hydrochlorothiazide | Hydralazine & Hydrochlorothiazide Cap 50-50 MG |
| 00083015930 | Hydralazine & Hydrochlorothiazide | Hydralazine & Hydrochlorothiazide Cap 100-50 MG |
| 00102300501 | Hydralazine & Hydrochlorothiazide | Hydralazine & Hydrochlorothiazide Cap 25-25 MG |
| 00102301001 | Hydralazine & Hydrochlorothiazide | Hydralazine & Hydrochlorothiazide Cap 50-50 MG |
| 49884014301 | Hydralazine & Hydrochlorothiazide | Hydralazine & Hydrochlorothiazide Cap 25-25 MG |
| 49884014305 | Hydralazine & Hydrochlorothiazide | Hydralazine & Hydrochlorothiazide Cap 25-25 MG |
| 49884014401 | Hydralazine & Hydrochlorothiazide | Hydralazine & Hydrochlorothiazide Cap 50-50 MG |
| 49884014405 | Hydralazine & Hydrochlorothiazide | Hydralazine & Hydrochlorothiazide Cap 50-50 MG |
| 49884014501 | Hydralazine & Hydrochlorothiazide | Hydralazine & Hydrochlorothiazide Cap 100-50 MG |
| 00536388501 | Hydralazine & Hydrochlorothiazide | Hydralazine & Hydrochlorothiazide Cap 25-25 MG |
| 00536388505 | Hydralazine & Hydrochlorothiazide | Hydralazine & Hydrochlorothiazide Cap 25-25 MG |
| 00536388601 | Hydralazine & Hydrochlorothiazide | Hydralazine & Hydrochlorothiazide Cap 50-50 MG |
| 00536388605 | Hydralazine & Hydrochlorothiazide | Hydralazine & Hydrochlorothiazide Cap 50-50 MG |
| 00536388701 | Hydralazine & Hydrochlorothiazide | Hydralazine & Hydrochlorothiazide Cap 100-50 MG |
| 00536327901 | Hydralazine & Hydrochlorothiazide | Hydralazine & Hydrochlorothiazide Tab 25-15 MG |
| 00536327910 | Hydralazine & Hydrochlorothiazide | Hydralazine & Hydrochlorothiazide Tab 25-15 MG |
| 00537622901 | Hydralazine & Hydrochlorothiazide | Hydralazine & Hydrochlorothiazide Tab 25-15 MG |
| 00537622910 | Hydralazine & Hydrochlorothiazide | Hydralazine & Hydrochlorothiazide Tab 25-15 MG |
| 00580135101 | Hydralazine & Hydrochlorothiazide | Hydralazine & Hydrochlorothiazide Cap 25-25 MG |
| 00580135201 | Hydralazine & Hydrochlorothiazide | Hydralazine & Hydrochlorothiazide Cap 50-50 MG |
| 00580135301 | Hydralazine & Hydrochlorothiazide | Hydralazine & Hydrochlorothiazide Cap 100-50 MG |
| 00304081301 | Hydralazine & Hydrochlorothiazide | Hydralazine & Hydrochlorothiazide Cap 25-25 MG |
| 00304081305 | Hydralazine & Hydrochlorothiazide | Hydralazine & Hydrochlorothiazide Cap 25-25 MG |
| 00304081401 | Hydralazine & Hydrochlorothiazide | Hydralazine & Hydrochlorothiazide Cap 50-50 MG |
| 00349802501 | Hydralazine & Hydrochlorothiazide | Hydralazine & Hydrochlorothiazide Cap 25-25 MG |
| 00349802505 | Hydralazine & Hydrochlorothiazide | Hydralazine & Hydrochlorothiazide Cap 25-25 MG |
| 00349802601 | Hydralazine & Hydrochlorothiazide | Hydralazine & Hydrochlorothiazide Cap 50-50 MG |
| 00349802605 | Hydralazine & Hydrochlorothiazide | Hydralazine & Hydrochlorothiazide Cap 50-50 MG |
| 00364035801 | Hydralazine & Hydrochlorothiazide | Hydralazine & Hydrochlorothiazide Tab 25-15 MG |
| 00364061601 | Hydralazine & Hydrochlorothiazide | Hydralazine & Hydrochlorothiazide Cap 25-25 MG |
| 00364061701 | Hydralazine & Hydrochlorothiazide | Hydralazine & Hydrochlorothiazide Cap 50-50 MG |
| 00405447701 | Hydralazine & Hydrochlorothiazide | Hydralazine & Hydrochlorothiazide Cap 25-25 MG |
| 00405447801 | Hydralazine & Hydrochlorothiazide | Hydralazine & Hydrochlorothiazide Cap 50-50 MG |
| 00182097210 | Hydralazine & Hydrochlorothiazide | Hydralazine & Hydrochlorothiazide Tab 25-15 MG |
| 00182148301 | Hydralazine & Hydrochlorothiazide | Hydralazine & Hydrochlorothiazide Cap 25-25 MG |
| 00182148401 | Hydralazine & Hydrochlorothiazide | Hydralazine & Hydrochlorothiazide Cap 50-50 MG |
| 00182150901 | Hydralazine & Hydrochlorothiazide | Hydralazine & Hydrochlorothiazide Cap 25-25 MG |
| 00182151001 | Hydralazine & Hydrochlorothiazide | Hydralazine & Hydrochlorothiazide Cap 50-50 MG |
| 00223104701 | Hydralazine & Hydrochlorothiazide | Hydralazine & Hydrochlorothiazide Tab 50-50 MG |
| 00223104705 | Hydralazine & Hydrochlorothiazide | Hydralazine & Hydrochlorothiazide Tab 50-50 MG |
| 00223104801 | Hydralazine & Hydrochlorothiazide | Hydralazine & Hydrochlorothiazide Tab 100-50 MG |
| 00223104805 | Hydralazine & Hydrochlorothiazide | Hydralazine & Hydrochlorothiazide Tab 100-50 MG |
| 00228219610 | Hydralazine & Hydrochlorothiazide | Hydralazine & Hydrochlorothiazide Cap 25-25 MG |
| 47202237501 | Hydralazine & Hydrochlorothiazide | Hydralazine & Hydrochlorothiazide Cap 25-25 MG |
| 47202237502 | Hydralazine & Hydrochlorothiazide | Hydralazine & Hydrochlorothiazide Cap 25-25 MG |
| 47202237601 | Hydralazine & Hydrochlorothiazide | Hydralazine & Hydrochlorothiazide Cap 50-50 MG |
| 47202237602 | Hydralazine & Hydrochlorothiazide | Hydralazine & Hydrochlorothiazide Cap 50-50 MG |
| 47202237701 | Hydralazine & Hydrochlorothiazide | Hydralazine & Hydrochlorothiazide Cap 100-50 MG |
| 47202277501 | Hydralazine & Hydrochlorothiazide | Hydralazine & Hydrochlorothiazide Cap 25-25 MG |
| 47202277601 | Hydralazine & Hydrochlorothiazide | Hydralazine & Hydrochlorothiazide Cap 50-50 MG |
| 00725006505 | Hydralazine & Hydrochlorothiazide | Hydralazine & Hydrochlorothiazide Cap 25-25 MG |
| 00725006601 | Hydralazine & Hydrochlorothiazide | Hydralazine & Hydrochlorothiazide Cap 50-50 MG |
| 00725006605 | Hydralazine & Hydrochlorothiazide | Hydralazine & Hydrochlorothiazide Cap 50-50 MG |
| 00725008301 | Hydralazine & Hydrochlorothiazide | Hydralazine & Hydrochlorothiazide Tab 25-15 MG |
| 00725008310 | Hydralazine & Hydrochlorothiazide | Hydralazine & Hydrochlorothiazide Tab 25-15 MG |
| 00781261001 | Hydralazine & Hydrochlorothiazide | Hydralazine & Hydrochlorothiazide Cap 25-25 MG |
| 00781261201 | Hydralazine & Hydrochlorothiazide | Hydralazine & Hydrochlorothiazide Cap 50-50 MG |
| 00814371514 | Hydralazine & Hydrochlorothiazide | Hydralazine & Hydrochlorothiazide Cap 25-25 MG |
| 00814371528 | Hydralazine & Hydrochlorothiazide | Hydralazine & Hydrochlorothiazide Cap 25-25 MG |
| 00839658206 | Hydralazine & Hydrochlorothiazide | Hydralazine & Hydrochlorothiazide Cap 25-25 MG |
| 00839658216 | Hydralazine & Hydrochlorothiazide | Hydralazine & Hydrochlorothiazide Cap 25-25 MG |
| 00839658306 | Hydralazine & Hydrochlorothiazide | Hydralazine & Hydrochlorothiazide Cap 50-50 MG |
| 00839677406 | Hydralazine & Hydrochlorothiazide | Hydralazine & Hydrochlorothiazide Tab 25-15 MG |
| 00603383421 | Hydralazine & Hydrochlorothiazide | Hydralazine & Hydrochlorothiazide Cap 25-25 MG |
| 00603383521 | Hydralazine & Hydrochlorothiazide | Hydralazine & Hydrochlorothiazide Cap 50-50 MG |
| 00603383621 | Hydralazine & Hydrochlorothiazide | Hydralazine & Hydrochlorothiazide Cap 100-50 MG |
| 00665441006 | Hydralazine & Hydrochlorothiazide | Hydralazine & Hydrochlorothiazide Cap 25-25 MG |
| 00665442006 | Hydralazine & Hydrochlorothiazide | Hydralazine & Hydrochlorothiazide Cap 50-50 MG |
| 00677077301 | Hydralazine & Hydrochlorothiazide | Hydralazine & Hydrochlorothiazide Cap 25-25 MG |
| 00677077401 | Hydralazine & Hydrochlorothiazide | Hydralazine & Hydrochlorothiazide Cap 50-50 MG |
| 00719144510 | Hydralazine & Hydrochlorothiazide | Hydralazine & Hydrochlorothiazide Tab 25-15 MG |
| 00719145510 | Hydralazine & Hydrochlorothiazide | Hydralazine & Hydrochlorothiazide Cap 25-25 MG |
| 00719145610 | Hydralazine & Hydrochlorothiazide | Hydralazine & Hydrochlorothiazide Cap 50-50 MG |
| 00725006401 | Hydralazine & Hydrochlorothiazide | Hydralazine & Hydrochlorothiazide Cap 100-50 MG |
| 00725006405 | Hydralazine & Hydrochlorothiazide | Hydralazine & Hydrochlorothiazide Cap 100-50 MG |
| 00725006501 | Hydralazine & Hydrochlorothiazide | Hydralazine & Hydrochlorothiazide Cap 25-25 MG |
| 52446021021 | Hydralazine & Hydrochlorothiazide | Hydralazine & Hydrochlorothiazide Cap 100-50 MG |
| 52446021321 | Hydralazine & Hydrochlorothiazide | Hydralazine & Hydrochlorothiazide Tab 25-25 MG |
| 52555014301 | Hydralazine & Hydrochlorothiazide | Hydralazine & Hydrochlorothiazide Cap 25-25 MG |
| 52555014401 | Hydralazine & Hydrochlorothiazide | Hydralazine & Hydrochlorothiazide Cap 50-50 MG |
| 54274000310 | Hydralazine & Hydrochlorothiazide | Hydralazine & Hydrochlorothiazide Cap 25-25 MG |
| 54274000330 | Hydralazine & Hydrochlorothiazide | Hydralazine & Hydrochlorothiazide Cap 25-25 MG |
| 54274004510 | Hydralazine & Hydrochlorothiazide | Hydralazine & Hydrochlorothiazide Cap 50-50 MG |
| 49884014310 | Hydralazine & Hydrochlorothiazide | Hydralazine & Hydrochlorothiazide Cap 25-25 MG |
| 49884014410 | Hydralazine & Hydrochlorothiazide | Hydralazine & Hydrochlorothiazide Cap 50-50 MG |
| 49884014505 | Hydralazine & Hydrochlorothiazide | Hydralazine & Hydrochlorothiazide Cap 100-50 MG |
| 49884014510 | Hydralazine & Hydrochlorothiazide | Hydralazine & Hydrochlorothiazide Cap 100-50 MG |
| 49727001802 | Hydralazine & Hydrochlorothiazide | Hydralazine & Hydrochlorothiazide Tab 25-25 MG |
| 49727001804 | Hydralazine & Hydrochlorothiazide | Hydralazine & Hydrochlorothiazide Tab 25-25 MG |
| 51432020303 | Hydralazine & Hydrochlorothiazide | Hydralazine & Hydrochlorothiazide Cap 25-25 MG |
| 51432091303 | Hydralazine & Hydrochlorothiazide | Hydralazine & Hydrochlorothiazide Cap 100-50 MG |
| 51432091403 | Hydralazine & Hydrochlorothiazide | Hydralazine & Hydrochlorothiazide Cap 50-50 MG |
| 51728002101 | Hydralazine & Hydrochlorothiazide | Hydralazine & Hydrochlorothiazide Cap 25-25 MG |
| 51728002105 | Hydralazine & Hydrochlorothiazide | Hydralazine & Hydrochlorothiazide Cap 25-25 MG |
| 51728002201 | Hydralazine & Hydrochlorothiazide | Hydralazine & Hydrochlorothiazide Cap 50-50 MG |
| 51728002205 | Hydralazine & Hydrochlorothiazide | Hydralazine & Hydrochlorothiazide Cap 50-50 MG |
| 51728002301 | Hydralazine & Hydrochlorothiazide | Hydralazine & Hydrochlorothiazide Cap 100-50 MG |
| 52446020821 | Hydralazine & Hydrochlorothiazide | Hydralazine & Hydrochlorothiazide Cap 25-25 MG |
| 52446020921 | Hydralazine & Hydrochlorothiazide | Hydralazine & Hydrochlorothiazide Cap 50-50 MG |
| 00904285260 | Hydralazine & Hydrochlorothiazide | Hydralazine & Hydrochlorothiazide Cap 25-25 MG |
| 00904285360 | Hydralazine & Hydrochlorothiazide | Hydralazine & Hydrochlorothiazide Cap 50-50 MG |
| 00904285560 | Hydralazine & Hydrochlorothiazide | Hydralazine & Hydrochlorothiazide Cap 25-25 MG |
| 00904285570 | Hydralazine & Hydrochlorothiazide | Hydralazine & Hydrochlorothiazide Cap 25-25 MG |
| 00904285660 | Hydralazine & Hydrochlorothiazide | Hydralazine & Hydrochlorothiazide Cap 50-50 MG |
| 00904285670 | Hydralazine & Hydrochlorothiazide | Hydralazine & Hydrochlorothiazide Cap 50-50 MG |
| 00904285760 | Hydralazine & Hydrochlorothiazide | Hydralazine & Hydrochlorothiazide Cap 100-50 MG |
| 00904266960 | Hydralazine & Hydrochlorothiazide | Hydralazine & Hydrochlorothiazide Tab 25-15 MG |
| 00904266970 | Hydralazine & Hydrochlorothiazide | Hydralazine & Hydrochlorothiazide Tab 25-15 MG |
| 17236047801 | Hydralazine & Hydrochlorothiazide | Hydralazine & Hydrochlorothiazide Cap 50-50 MG |
| 00814687030 | Hydralazine & Reserpine & Hydrochlorothiazide | Hydralazine-Reserpine-Hydrochlorothiazide Tab 25-0.1-15 MG |
| 00839128206 | Hydralazine & Reserpine & Hydrochlorothiazide | Hydralazine-Reserpine-Hydrochlorothiazide Tab 25-0.1-15 MG |
| 00839128216 | Hydralazine & Reserpine & Hydrochlorothiazide | Hydralazine-Reserpine-Hydrochlorothiazide Tab 25-0.1-15 MG |
| 00580035201 | Hydralazine & Reserpine & Hydrochlorothiazide | Hydralazine-Reserpine-Hydrochlorothiazide Tab 25-0.1-15 MG |
| 00580035210 | Hydralazine & Reserpine & Hydrochlorothiazide | Hydralazine-Reserpine-Hydrochlorothiazide Tab 25-0.1-15 MG |
| 00591542801 | Hydralazine & Reserpine & Hydrochlorothiazide | Hydralazine-Reserpine-Hydrochlorothiazide Tab 25-0.1-15 MG |
| 00591542804 | Hydralazine & Reserpine & Hydrochlorothiazide | Hydralazine-Reserpine-Hydrochlorothiazide Tab 25-0.1-15 MG |
| 00603380721 | Hydralazine & Reserpine & Hydrochlorothiazide | Hydralazine-Reserpine-Hydrochlorothiazide Tab 25-0.1-15 MG |
| 00603380732 | Hydralazine & Reserpine & Hydrochlorothiazide | Hydralazine-Reserpine-Hydrochlorothiazide Tab 25-0.1-15 MG |
| 00615057601 | Hydralazine & Reserpine & Hydrochlorothiazide | Hydralazine-Reserpine-Hydrochlorothiazide Tab 25-0.1-15 MG |
| 00615057610 | Hydralazine & Reserpine & Hydrochlorothiazide | Hydralazine-Reserpine-Hydrochlorothiazide Tab 25-0.1-15 MG |
| 00615251501 | Hydralazine & Reserpine & Hydrochlorothiazide | Hydralazine-Reserpine-Hydrochlorothiazide Tab 25-0.1-15 MG |
| 00615251510 | Hydralazine & Reserpine & Hydrochlorothiazide | Hydralazine-Reserpine-Hydrochlorothiazide Tab 25-0.1-15 MG |
| 00665139306 | Hydralazine & Reserpine & Hydrochlorothiazide | Hydralazine-Reserpine-Hydrochlorothiazide Tab 25-0.1-15 MG |
| 00665139309 | Hydralazine & Reserpine & Hydrochlorothiazide | Hydralazine-Reserpine-Hydrochlorothiazide Tab 25-0.1-15 MG |
| 00682180001 | Hydralazine & Reserpine & Hydrochlorothiazide | Hydralazine-Reserpine-Hydrochlorothiazide Tab 25-0.1-15 MG |
| 00719143410 | Hydralazine & Reserpine & Hydrochlorothiazide | Hydralazine-Reserpine-Hydrochlorothiazide Tab 25-0.1-15 MG |
| 00719143413 | Hydralazine & Reserpine & Hydrochlorothiazide | Hydralazine-Reserpine-Hydrochlorothiazide Tab 25-0.1-15 MG |
| 00719143510 | Hydralazine & Reserpine & Hydrochlorothiazide | Hydralazine-Reserpine-Hydrochlorothiazide Tab 25-0.1-15 MG |
| 00525050001 | Hydralazine & Reserpine & Hydrochlorothiazide | Hydralazine-Reserpine-Hydrochlorothiazide Tab 25-0.1-15 MG |
| 54977029030 | Hydralazine & Reserpine & Hydrochlorothiazide | Hydralazine-Reserpine-Hydrochlorothiazide Tab 25-0.1-15 MG |
| 54977029099 | Hydralazine & Reserpine & Hydrochlorothiazide | Hydralazine-Reserpine-Hydrochlorothiazide Tab 25-0.1-15 MG |
| 55081049601 | Hydralazine & Reserpine & Hydrochlorothiazide | Hydralazine-Reserpine-Hydrochlorothiazide Tab 25-0.1-15 MG |
| 55175076300 | Hydralazine & Reserpine & Hydrochlorothiazide | Hydralazine-Reserpine-Hydrochlorothiazide Tab 25-0.1-15 MG |
| 52446043121 | Hydralazine & Reserpine & Hydrochlorothiazide | Hydralazine-Reserpine-Hydrochlorothiazide Tab 25-0.1-15 MG |
| 52446043132 | Hydralazine & Reserpine & Hydrochlorothiazide | Hydralazine-Reserpine-Hydrochlorothiazide Tab 25-0.1-15 MG |
| 52493063301 | Hydralazine & Reserpine & Hydrochlorothiazide | Hydralazine-Reserpine-Hydrochlorothiazide Tab 25-0.1-15 MG |
| 52765110800 | Hydralazine & Reserpine & Hydrochlorothiazide | Hydralazine-Reserpine-Hydrochlorothiazide Tab 25-0.1-15 MG |
| 53489031901 | Hydralazine & Reserpine & Hydrochlorothiazide | Hydralazine-Reserpine-Hydrochlorothiazide Tab 25-0.1-15 MG |
| 53489031910 | Hydralazine & Reserpine & Hydrochlorothiazide | Hydralazine-Reserpine-Hydrochlorothiazide Tab 25-0.1-15 MG |
| 54274006510 | Hydralazine & Reserpine & Hydrochlorothiazide | Hydralazine-Reserpine-Hydrochlorothiazide Tab 25-0.1-15 MG |
| 54274006550 | Hydralazine & Reserpine & Hydrochlorothiazide | Hydralazine-Reserpine-Hydrochlorothiazide Tab 25-0.1-15 MG |
| 54569059500 | Hydralazine & Reserpine & Hydrochlorothiazide | Hydralazine-Reserpine-Hydrochlorothiazide Tab 25-0.1-15 MG |
| 54569059501 | Hydralazine & Reserpine & Hydrochlorothiazide | Hydralazine-Reserpine-Hydrochlorothiazide Tab 25-0.1-15 MG |
| 54569353700 | Hydralazine & Reserpine & Hydrochlorothiazide | Hydralazine-Reserpine-Hydrochlorothiazide Tab 25-0.1-15 MG |
| 51079016720 | Hydralazine & Reserpine & Hydrochlorothiazide | Hydralazine-Reserpine-Hydrochlorothiazide Tab 25-0.1-15 MG |
| 51079016740 | Hydralazine & Reserpine & Hydrochlorothiazide | Hydralazine-Reserpine-Hydrochlorothiazide Tab 25-0.1-15 MG |
| 51432022000 | Hydralazine & Reserpine & Hydrochlorothiazide | Hydralazine-Reserpine-Hydrochlorothiazide Tab 25-0.1-15 MG |
| 51432022006 | Hydralazine & Reserpine & Hydrochlorothiazide | Hydralazine-Reserpine-Hydrochlorothiazide Tab 25-0.1-15 MG |
| 51728042110 | Hydralazine & Reserpine & Hydrochlorothiazide | Hydralazine-Reserpine-Hydrochlorothiazide Tab 25-0.1-15 MG |
| 52246067590 | Hydralazine & Reserpine & Hydrochlorothiazide | Hydralazine-Reserpine-Hydrochlorothiazide Tab 25-0.1-15 MG |
| 00904233460 | Hydralazine & Reserpine & Hydrochlorothiazide | Hydralazine-Reserpine-Hydrochlorothiazide Tab 25-0.1-15 MG |
| 00904233480 | Hydralazine & Reserpine & Hydrochlorothiazide | Hydralazine-Reserpine-Hydrochlorothiazide Tab 25-0.1-15 MG |
| 00904233560 | Hydralazine & Reserpine & Hydrochlorothiazide | Hydralazine-Reserpine-Hydrochlorothiazide Tab 25-0.1-15 MG |
| 00904233561 | Hydralazine & Reserpine & Hydrochlorothiazide | Hydralazine-Reserpine-Hydrochlorothiazide Tab 25-0.1-15 MG |
| 00904233570 | Hydralazine & Reserpine & Hydrochlorothiazide | Hydralazine-Reserpine-Hydrochlorothiazide Tab 25-0.1-15 MG |
| 00904233580 | Hydralazine & Reserpine & Hydrochlorothiazide | Hydralazine-Reserpine-Hydrochlorothiazide Tab 25-0.1-15 MG |
| 17236015001 | Hydralazine & Reserpine & Hydrochlorothiazide | Hydralazine-Reserpine-Hydrochlorothiazide Tab 25-0.1-15 MG |
| 17236015010 | Hydralazine & Reserpine & Hydrochlorothiazide | Hydralazine-Reserpine-Hydrochlorothiazide Tab 25-0.1-15 MG |
| 40893063301 | Hydralazine & Reserpine & Hydrochlorothiazide | Hydralazine-Reserpine-Hydrochlorothiazide Tab 25-0.1-15 MG |
| 47202206501 | Hydralazine & Reserpine & Hydrochlorothiazide | Hydralazine-Reserpine-Hydrochlorothiazide Tab 25-0.1-15 MG |
| 00228222710 | Hydralazine & Reserpine & Hydrochlorothiazide | Hydralazine-Reserpine-Hydrochlorothiazide Tab 25-0.1-15 MG |
| 00228222796 | Hydralazine & Reserpine & Hydrochlorothiazide | Hydralazine-Reserpine-Hydrochlorothiazide Tab 25-0.1-15 MG |
| 00005378323 | Hydralazine & Reserpine & Hydrochlorothiazide | Hydralazine-Reserpine-Hydrochlorothiazide Tab 25-0.1-15 MG |
| 00005378331 | Hydralazine & Reserpine & Hydrochlorothiazide | Hydralazine-Reserpine-Hydrochlorothiazide Tab 25-0.1-15 MG |
| 00032113201 | Hydralazine & Reserpine & Hydrochlorothiazide | Hydralazine-Reserpine-Hydrochlorothiazide Tab 25-0.1-15 MG |
| 00063113206 | Hydralazine & Reserpine & Hydrochlorothiazide | Hydralazine-Reserpine-Hydrochlorothiazide Tab 25-0.1-15 MG |
| 00063113209 | Hydralazine & Reserpine & Hydrochlorothiazide | Hydralazine-Reserpine-Hydrochlorothiazide Tab 25-0.1-15 MG |
| 00083007130 | Hydralazine & Reserpine & Hydrochlorothiazide | Hydralazine-Reserpine-Hydrochlorothiazide Tab 25-0.1-15 MG |
| 00083007132 | Hydralazine & Reserpine & Hydrochlorothiazide | Hydralazine-Reserpine-Hydrochlorothiazide Tab 25-0.1-15 MG |
| 00083007140 | Hydralazine & Reserpine & Hydrochlorothiazide | Hydralazine-Reserpine-Hydrochlorothiazide Tab 25-0.1-15 MG |
| 00083007199 | Hydralazine & Reserpine & Hydrochlorothiazide | Hydralazine-Reserpine-Hydrochlorothiazide Tab 25-0.1-15 MG |
| 00102112501 | Hydralazine & Reserpine & Hydrochlorothiazide | Hydralazine-Reserpine-Hydrochlorothiazide Tab 25-0.1-15 MG |
| 00093055801 | Hydralazine & Reserpine & Hydrochlorothiazide | Hydralazine-Reserpine-Hydrochlorothiazide Tab 25-0.1-15 MG |
| 00093055810 | Hydralazine & Reserpine & Hydrochlorothiazide | Hydralazine-Reserpine-Hydrochlorothiazide Tab 25-0.1-15 MG |
| 00144156303 | Hydralazine & Reserpine & Hydrochlorothiazide | Hydralazine-Reserpine-Hydrochlorothiazide Tab 25-0.1-15 MG |
| 00157040801 | Hydralazine & Reserpine & Hydrochlorothiazide | Hydralazine-Reserpine-Hydrochlorothiazide Tab 25-0.1-15 MG |
| 00157040810 | Hydralazine & Reserpine & Hydrochlorothiazide | Hydralazine-Reserpine-Hydrochlorothiazide Tab 25-0.1-15 MG |
| 00147012410 | Hydralazine & Reserpine & Hydrochlorothiazide | Hydralazine-Reserpine-Hydrochlorothiazide Tab 25-0.1-15 MG |
| 00147012420 | Hydralazine & Reserpine & Hydrochlorothiazide | Hydralazine-Reserpine-Hydrochlorothiazide Tab 25-0.1-15 MG |
| 55289036101 | Hydralazine & Reserpine & Hydrochlorothiazide | Hydralazine-Reserpine-Hydrochlorothiazide Tab 25-0.1-15 MG |
| 60346079490 | Hydralazine & Reserpine & Hydrochlorothiazide | Hydralazine-Reserpine-Hydrochlorothiazide Tab 25-0.1-15 MG |
| 00677041501 | Hydralazine & Reserpine & Hydrochlorothiazide | Hydralazine-Reserpine-Hydrochlorothiazide Tab 25-0.1-15 MG |
| 00677041510 | Hydralazine & Reserpine & Hydrochlorothiazide | Hydralazine-Reserpine-Hydrochlorothiazide Tab 25-0.1-15 MG |
| 00527105801 | Hydralazine & Reserpine & Hydrochlorothiazide | Hydralazine-Reserpine-Hydrochlorothiazide Tab 25-0.1-15 MG |
| 00527105810 | Hydralazine & Reserpine & Hydrochlorothiazide | Hydralazine-Reserpine-Hydrochlorothiazide Tab 25-0.1-15 MG |
| 00536472101 | Hydralazine & Reserpine & Hydrochlorothiazide | Hydralazine-Reserpine-Hydrochlorothiazide Tab 25-0.1-15 MG |
| 00536472110 | Hydralazine & Reserpine & Hydrochlorothiazide | Hydralazine-Reserpine-Hydrochlorothiazide Tab 25-0.1-15 MG |
| 00536490901 | Hydralazine & Reserpine & Hydrochlorothiazide | Hydralazine-Reserpine-Hydrochlorothiazide Tab 25-0.1-15 MG |
| 00536490910 | Hydralazine & Reserpine & Hydrochlorothiazide | Hydralazine-Reserpine-Hydrochlorothiazide Tab 25-0.1-15 MG |
| 00537252301 | Hydralazine & Reserpine & Hydrochlorothiazide | Hydralazine-Reserpine-Hydrochlorothiazide Tab 25-0.1-15 MG |
| 00537252310 | Hydralazine & Reserpine & Hydrochlorothiazide | Hydralazine-Reserpine-Hydrochlorothiazide Tab 25-0.1-15 MG |
| 00555011502 | Hydralazine & Reserpine & Hydrochlorothiazide | Hydralazine-Reserpine-Hydrochlorothiazide Tab 25-0.1-15 MG |
| 00555011505 | Hydralazine & Reserpine & Hydrochlorothiazide | Hydralazine-Reserpine-Hydrochlorothiazide Tab 25-0.1-15 MG |
| 00556013701 | Hydralazine & Reserpine & Hydrochlorothiazide | Hydralazine-Reserpine-Hydrochlorothiazide Tab 25-0.1-15 MG |
| 00556013710 | Hydralazine & Reserpine & Hydrochlorothiazide | Hydralazine-Reserpine-Hydrochlorothiazide Tab 25-0.1-15 MG |
| 00302631501 | Hydralazine & Reserpine & Hydrochlorothiazide | Hydralazine-Reserpine-Hydrochlorothiazide Tab 25-0.1-15 MG |
| 00302631510 | Hydralazine & Reserpine & Hydrochlorothiazide | Hydralazine-Reserpine-Hydrochlorothiazide Tab 25-0.1-15 MG |
| 00304014800 | Hydralazine & Reserpine & Hydrochlorothiazide | Hydralazine-Reserpine-Hydrochlorothiazide Tab 25-0.1-15 MG |
| 00304014801 | Hydralazine & Reserpine & Hydrochlorothiazide | Hydralazine-Reserpine-Hydrochlorothiazide Tab 25-0.1-15 MG |
| 00349207601 | Hydralazine & Reserpine & Hydrochlorothiazide | Hydralazine-Reserpine-Hydrochlorothiazide Tab 25-0.1-15 MG |
| 00349207610 | Hydralazine & Reserpine & Hydrochlorothiazide | Hydralazine-Reserpine-Hydrochlorothiazide Tab 25-0.1-15 MG |
| 00364036101 | Hydralazine & Reserpine & Hydrochlorothiazide | Hydralazine-Reserpine-Hydrochlorothiazide Tab 25-0.1-15 MG |
| 00364036102 | Hydralazine & Reserpine & Hydrochlorothiazide | Hydralazine-Reserpine-Hydrochlorothiazide Tab 25-0.1-15 MG |
| 00385205801 | Hydralazine & Reserpine & Hydrochlorothiazide | Hydralazine-Reserpine-Hydrochlorothiazide Tab 25-0.1-15 MG |
| 00385205810 | Hydralazine & Reserpine & Hydrochlorothiazide | Hydralazine-Reserpine-Hydrochlorothiazide Tab 25-0.1-15 MG |
| 00405448401 | Hydralazine & Reserpine & Hydrochlorothiazide | Hydralazine-Reserpine-Hydrochlorothiazide Tab 25-0.1-15 MG |
| 00405448403 | Hydralazine & Reserpine & Hydrochlorothiazide | Hydralazine-Reserpine-Hydrochlorothiazide Tab 25-0.1-15 MG |
| 00403190401 | Hydralazine & Reserpine & Hydrochlorothiazide | Hydralazine-Reserpine-Hydrochlorothiazide Tab 25-0.1-15 MG |
| 00485004010 | Hydralazine & Reserpine & Hydrochlorothiazide | Hydralazine-Reserpine-Hydrochlorothiazide Tab 25-0.1-15 MG |
| 00182182001 | Hydralazine & Reserpine & Hydrochlorothiazide | Hydralazine-Reserpine-Hydrochlorothiazide Tab 25-0.1-15 MG |
| 00182182010 | Hydralazine & Reserpine & Hydrochlorothiazide | Hydralazine-Reserpine-Hydrochlorothiazide Tab 25-0.1-15 MG |
| 00223107301 | Hydralazine & Reserpine & Hydrochlorothiazide | Hydralazine-Reserpine-Hydrochlorothiazide Tab 25-0.1-15 MG |
| 00223107302 | Hydralazine & Reserpine & Hydrochlorothiazide | Hydralazine-Reserpine-Hydrochlorothiazide Tab 25-0.1-15 MG |
| 47202206503 | Hydralazine & Reserpine & Hydrochlorothiazide | Hydralazine-Reserpine-Hydrochlorothiazide Tab 25-0.1-15 MG |
| 43797039506 | Hydralazine & Reserpine & Hydrochlorothiazide | Hydralazine-Reserpine-Hydrochlorothiazide Tab 25-0.1-15 MG |
| 46703003810 | Hydralazine & Reserpine & Hydrochlorothiazide | Hydralazine-Reserpine-Hydrochlorothiazide Tab 25-0.1-15 MG |
| 00725204901 | Hydralazine & Reserpine & Hydrochlorothiazide | Hydralazine-Reserpine-Hydrochlorothiazide Tab 25-0.1-15 MG |
| 00725204904 | Hydralazine & Reserpine & Hydrochlorothiazide | Hydralazine-Reserpine-Hydrochlorothiazide Tab 25-0.1-15 MG |
| 00725204910 | Hydralazine & Reserpine & Hydrochlorothiazide | Hydralazine-Reserpine-Hydrochlorothiazide Tab 25-0.1-15 MG |
| 00779058325 | Hydralazine & Reserpine & Hydrochlorothiazide | Hydralazine-Reserpine-Hydrochlorothiazide Tab 25-0.1-15 MG |
| 00779058330 | Hydralazine & Reserpine & Hydrochlorothiazide | Hydralazine-Reserpine-Hydrochlorothiazide Tab 25-0.1-15 MG |
| 00781134301 | Hydralazine & Reserpine & Hydrochlorothiazide | Hydralazine-Reserpine-Hydrochlorothiazide Tab 25-0.1-15 MG |
| 00781134310 | Hydralazine & Reserpine & Hydrochlorothiazide | Hydralazine-Reserpine-Hydrochlorothiazide Tab 25-0.1-15 MG |
| 00814687014 | Hydralazine & Reserpine & Hydrochlorothiazide | Hydralazine-Reserpine-Hydrochlorothiazide Tab 25-0.1-15 MG |
| 55048035330 | Hydralazine HCl | Hydralazine HCl Tab 25 MG |
| 55048035430 | Hydralazine HCl | Hydralazine HCl Tab 50 MG |
| 43353075680 | Hydralazine HCl | Hydralazine HCl Tab 25 MG |
| 54868289303 | Hydralazine HCl | Hydralazine HCl Tab 50 MG |
| 17478093415 | Hydralazine HCl | Hydralazine HCl Inj 20 MG/ML |
| 63739012610 | Hydralazine HCl | Hydralazine HCl Tab 25 MG |
| 63739012710 | Hydralazine HCl | Hydralazine HCl Tab 50 MG |
| 68462034101 | Hydralazine HCl | Hydralazine HCl Tab 10 MG |
| 68462034201 | Hydralazine HCl | Hydralazine HCl Tab 25 MG |
| 68462034301 | Hydralazine HCl | Hydralazine HCl Tab 50 MG |
| 68462034401 | Hydralazine HCl | Hydralazine HCl Tab 100 MG |
| 23155000101 | Hydralazine HCl | Hydralazine HCl Tab 10 MG |
| 23155000110 | Hydralazine HCl | Hydralazine HCl Tab 10 MG |
| 23155000401 | Hydralazine HCl | Hydralazine HCl Tab 100 MG |
| 60429006401 | Hydralazine HCl | Hydralazine HCl Tab 10 MG |
| 60429006410 | Hydralazine HCl | Hydralazine HCl Tab 10 MG |
| 60429006501 | Hydralazine HCl | Hydralazine HCl Tab 25 MG |
| 60429006510 | Hydralazine HCl | Hydralazine HCl Tab 25 MG |
| 60429006601 | Hydralazine HCl | Hydralazine HCl Tab 50 MG |
| 60429006610 | Hydralazine HCl | Hydralazine HCl Tab 50 MG |
| 60429006701 | Hydralazine HCl | Hydralazine HCl Tab 100 MG |
| 60429006710 | Hydralazine HCl | Hydralazine HCl Tab 100 MG |
| 60429006705 | Hydralazine HCl | Hydralazine HCl Tab 100 MG |
| 43353063660 | Hydralazine HCl | Hydralazine HCl Tab 50 MG |
| 21695067990 | Hydralazine HCl | Hydralazine HCl Tab 10 MG |
| 21695068090 | Hydralazine HCl | Hydralazine HCl Tab 25 MG |
| 21695069490 | Hydralazine HCl | Hydralazine HCl Tab 50 MG |
| 21695069590 | Hydralazine HCl | Hydralazine HCl Tab 100 MG |
| 68084044701 | Hydralazine HCl | Hydralazine HCl Tab 10 MG |
| 68084044711 | Hydralazine HCl | Hydralazine HCl Tab 10 MG |
| 68462034105 | Hydralazine HCl | Hydralazine HCl Tab 10 MG |
| 68462034205 | Hydralazine HCl | Hydralazine HCl Tab 25 MG |
| 68462034305 | Hydralazine HCl | Hydralazine HCl Tab 50 MG |
| 68462034405 | Hydralazine HCl | Hydralazine HCl Tab 100 MG |
| 47463035230 | Hydralazine HCl | Hydralazine HCl Tab 100 MG |
| 47463035330 | Hydralazine HCl | Hydralazine HCl Tab 25 MG |
| 47463035430 | Hydralazine HCl | Hydralazine HCl Tab 50 MG |
| 67544065760 | Hydralazine HCl | Hydralazine HCl Tab 25 MG |
| 43353067460 | Hydralazine HCl | Hydralazine HCl Tab 50 MG |
| 43353067480 | Hydralazine HCl | Hydralazine HCl Tab 50 MG |
| 43353067492 | Hydralazine HCl | Hydralazine HCl Tab 50 MG |
| 43353067494 | Hydralazine HCl | Hydralazine HCl Tab 50 MG |
| 43353067496 | Hydralazine HCl | Hydralazine HCl Tab 50 MG |
| 43353067498 | Hydralazine HCl | Hydralazine HCl Tab 50 MG |
| 43353074260 | Hydralazine HCl | Hydralazine HCl Tab 50 MG |
| 43353074280 | Hydralazine HCl | Hydralazine HCl Tab 50 MG |
| 43353074292 | Hydralazine HCl | Hydralazine HCl Tab 50 MG |
| 43353074294 | Hydralazine HCl | Hydralazine HCl Tab 50 MG |
| 43353074296 | Hydralazine HCl | Hydralazine HCl Tab 50 MG |
| 43353074298 | Hydralazine HCl | Hydralazine HCl Tab 50 MG |
| 51138034230 | Hydralazine HCl | Hydralazine HCl Tab 10 MG |
| 51138034330 | Hydralazine HCl | Hydralazine HCl Tab 25 MG |
| 51138034430 | Hydralazine HCl | Hydralazine HCl Tab 50 MG |
| 51138034530 | Hydralazine HCl | Hydralazine HCl Tab 100 MG |
| 51138035230 | Hydralazine HCl | Hydralazine HCl Tab 10 MG |
| 51138035330 | Hydralazine HCl | Hydralazine HCl Tab 25 MG |
| 51138035430 | Hydralazine HCl | Hydralazine HCl Tab 50 MG |
| 51138035530 | Hydralazine HCl | Hydralazine HCl Tab 100 MG |
| 68084052011 | Hydralazine HCl | Hydralazine HCl Tab 100 MG |
| 68084052021 | Hydralazine HCl | Hydralazine HCl Tab 100 MG |
| 43353075660 | Hydralazine HCl | Hydralazine HCl Tab 25 MG |
| 43353069460 | Hydralazine HCl | Hydralazine HCl Tab 25 MG |
| 43353069480 | Hydralazine HCl | Hydralazine HCl Tab 25 MG |
| 43353069492 | Hydralazine HCl | Hydralazine HCl Tab 25 MG |
| 21695068030 | Hydralazine HCl | Hydralazine HCl Tab 25 MG |
| 43353063692 | Hydralazine HCl | Hydralazine HCl Tab 50 MG |
| 43353063698 | Hydralazine HCl | Hydralazine HCl Tab 50 MG |
| 43353075692 | Hydralazine HCl | Hydralazine HCl Tab 25 MG |
| 55048035230 | Hydralazine HCl | Hydralazine HCl Tab 100 MG |
| 55081077201 | Hydralazine HCl | Hydralazine HCl Tab 10 MG |
| 55081095100 | Hydralazine HCl | Hydralazine HCl Tab 50 MG |
| 55081028100 | Hydralazine HCl | Hydralazine HCl Tab 25 MG |
| 55081028101 | Hydralazine HCl | Hydralazine HCl Tab 25 MG |
| 55081028102 | Hydralazine HCl | Hydralazine HCl Tab 25 MG |
| 55829030010 | Hydralazine HCl | Hydralazine HCl Tab 10 MG |
| 55829030110 | Hydralazine HCl | Hydralazine HCl Tab 25 MG |
| 55829030210 | Hydralazine HCl | Hydralazine HCl Tab 50 MG |
| 55829030310 | Hydralazine HCl | Hydralazine HCl Tab 100 MG |
| 55175445301 | Hydralazine HCl | Hydralazine HCl Tab 25 MG |
| 55175445401 | Hydralazine HCl | Hydralazine HCl Tab 50 MG |
| 55175445406 | Hydralazine HCl | Hydralazine HCl Tab 50 MG |
| 57783664001 | Hydralazine HCl | Hydralazine HCl Tab 10 MG |
| 57783664003 | Hydralazine HCl | Hydralazine HCl Tab 10 MG |
| 57783665001 | Hydralazine HCl | Hydralazine HCl Tab 25 MG |
| 57783665003 | Hydralazine HCl | Hydralazine HCl Tab 25 MG |
| 57783666001 | Hydralazine HCl | Hydralazine HCl Tab 50 MG |
| 57783666003 | Hydralazine HCl | Hydralazine HCl Tab 50 MG |
| 57480033101 | Hydralazine HCl | Hydralazine HCl Tab 10 MG |
| 57480033106 | Hydralazine HCl | Hydralazine HCl Tab 10 MG |
| 57480033201 | Hydralazine HCl | Hydralazine HCl Tab 25 MG |
| 57480033206 | Hydralazine HCl | Hydralazine HCl Tab 25 MG |
| 57480033301 | Hydralazine HCl | Hydralazine HCl Tab 50 MG |
| 57480033306 | Hydralazine HCl | Hydralazine HCl Tab 50 MG |
| 61392004330 | Hydralazine HCl | Hydralazine HCl Tab 25 MG |
| 61392004331 | Hydralazine HCl | Hydralazine HCl Tab 25 MG |
| 61392004332 | Hydralazine HCl | Hydralazine HCl Tab 25 MG |
| 61392004334 | Hydralazine HCl | Hydralazine HCl Tab 25 MG |
| 61392004339 | Hydralazine HCl | Hydralazine HCl Tab 25 MG |
| 61392004345 | Hydralazine HCl | Hydralazine HCl Tab 25 MG |
| 61392004351 | Hydralazine HCl | Hydralazine HCl Tab 25 MG |
| 61392004354 | Hydralazine HCl | Hydralazine HCl Tab 25 MG |
| 61392004356 | Hydralazine HCl | Hydralazine HCl Tab 25 MG |
| 61392004360 | Hydralazine HCl | Hydralazine HCl Tab 25 MG |
| 61392004390 | Hydralazine HCl | Hydralazine HCl Tab 25 MG |
| 61392004391 | Hydralazine HCl | Hydralazine HCl Tab 25 MG |
| 54868053602 | Hydralazine HCl | Hydralazine HCl Tab 100 MG |
| 54868194902 | Hydralazine HCl | Hydralazine HCl Tab 25 MG |
| 54868289302 | Hydralazine HCl | Hydralazine HCl Tab 50 MG |
| 54868578801 | Hydralazine HCl | Hydralazine HCl Tab 10 MG |
| 00182055400 | Hydralazine HCl | Hydralazine HCl Tab 25 MG |
| 00182055500 | Hydralazine HCl | Hydralazine HCl Tab 50 MG |
| 00182090500 | Hydralazine HCl | Hydralazine HCl Tab 10 MG |
| 51079007401 | Hydralazine HCl | Hydralazine HCl Tab 10 MG |
| 51079007501 | Hydralazine HCl | Hydralazine HCl Tab 25 MG |
| 51079007601 | Hydralazine HCl | Hydralazine HCl Tab 50 MG |
| 51079007517 | Hydralazine HCl | Hydralazine HCl Tab 25 MG |
| 51079007617 | Hydralazine HCl | Hydralazine HCl Tab 50 MG |
| 62584073301 | Hydralazine HCl | Hydralazine HCl Tab 25 MG |
| 62584073311 | Hydralazine HCl | Hydralazine HCl Tab 25 MG |
| 62584073401 | Hydralazine HCl | Hydralazine HCl Tab 50 MG |
| 62584073411 | Hydralazine HCl | Hydralazine HCl Tab 50 MG |
| 31722051901 | Hydralazine HCl | Hydralazine HCl Tab 10 MG |
| 31722052001 | Hydralazine HCl | Hydralazine HCl Tab 25 MG |
| 31722052010 | Hydralazine HCl | Hydralazine HCl Tab 25 MG |
| 31722052101 | Hydralazine HCl | Hydralazine HCl Tab 50 MG |
| 31722052110 | Hydralazine HCl | Hydralazine HCl Tab 50 MG |
| 31722052201 | Hydralazine HCl | Hydralazine HCl Tab 100 MG |
| 17478093401 | Hydralazine HCl | Hydralazine HCl Inj 20 MG/ML |
| 54868194903 | Hydralazine HCl | Hydralazine HCl Tab 25 MG |
| 52446022021 | Hydralazine HCl | Hydralazine HCl Tab 100 MG |
| 52555002601 | Hydralazine HCl | Hydralazine HCl Tab 100 MG |
| 52555002701 | Hydralazine HCl | Hydralazine HCl Tab 25 MG |
| 52555002710 | Hydralazine HCl | Hydralazine HCl Tab 25 MG |
| 52555002801 | Hydralazine HCl | Hydralazine HCl Tab 50 MG |
| 52555002810 | Hydralazine HCl | Hydralazine HCl Tab 50 MG |
| 52555002901 | Hydralazine HCl | Hydralazine HCl Tab 10 MG |
| 52555002910 | Hydralazine HCl | Hydralazine HCl Tab 10 MG |
| 52493060401 | Hydralazine HCl | Hydralazine HCl Tab 25 MG |
| 52493060501 | Hydralazine HCl | Hydralazine HCl Tab 50 MG |
| 53258017601 | Hydralazine HCl | Hydralazine HCl Tab 25 MG |
| 53258017613 | Hydralazine HCl | Hydralazine HCl Tab 25 MG |
| 53489012301 | Hydralazine HCl | Hydralazine HCl Tab 10 MG |
| 53489012310 | Hydralazine HCl | Hydralazine HCl Tab 10 MG |
| 53489012401 | Hydralazine HCl | Hydralazine HCl Tab 25 MG |
| 53489012410 | Hydralazine HCl | Hydralazine HCl Tab 25 MG |
| 53489012501 | Hydralazine HCl | Hydralazine HCl Tab 50 MG |
| 53489012510 | Hydralazine HCl | Hydralazine HCl Tab 50 MG |
| 53489031001 | Hydralazine HCl | Hydralazine HCl Tab 100 MG |
| 53506075940 | Hydralazine HCl | Hydralazine HCl Tab 10 MG |
| 54124087202 | Hydralazine HCl | Hydralazine HCl Tab 50 MG |
| 54274035150 | Hydralazine HCl | Hydralazine HCl Tab 10 MG |
| 54274035210 | Hydralazine HCl | Hydralazine HCl Tab 25 MG |
| 54274035250 | Hydralazine HCl | Hydralazine HCl Tab 25 MG |
| 54274035310 | Hydralazine HCl | Hydralazine HCl Tab 50 MG |
| 54274035350 | Hydralazine HCl | Hydralazine HCl Tab 50 MG |
| 54569051400 | Hydralazine HCl | Hydralazine HCl Tab 25 MG |
| 54569051502 | Hydralazine HCl | Hydralazine HCl Tab 25 MG |
| 54569051503 | Hydralazine HCl | Hydralazine HCl Tab 25 MG |
| 54569051600 | Hydralazine HCl | Hydralazine HCl Tab 50 MG |
| 54569051700 | Hydralazine HCl | Hydralazine HCl Tab 50 MG |
| 54569051800 | Hydralazine HCl | Hydralazine HCl Tab 100 MG |
| 54569207500 | Hydralazine HCl | Hydralazine HCl Inj 20 MG/ML |
| 54569706800 | Hydralazine HCl | Hydralazine HCl Tab 10 MG |
| 54569714600 | Hydralazine HCl | Hydralazine HCl Tab 25 MG |
| 54569714700 | Hydralazine HCl | Hydralazine HCl Tab 50 MG |
| 54807006001 | Hydralazine HCl | Hydralazine HCl Tab 10 MG |
| 54807006101 | Hydralazine HCl | Hydralazine HCl Tab 25 MG |
| 54807006201 | Hydralazine HCl | Hydralazine HCl Tab 50 MG |
| 54977002130 | Hydralazine HCl | Hydralazine HCl Tab 10 MG |
| 54977002199 | Hydralazine HCl | Hydralazine HCl Tab 10 MG |
| 54977002230 | Hydralazine HCl | Hydralazine HCl Tab 25 MG |
| 54977002299 | Hydralazine HCl | Hydralazine HCl Tab 25 MG |
| 54977002330 | Hydralazine HCl | Hydralazine HCl Tab 50 MG |
| 54977002399 | Hydralazine HCl | Hydralazine HCl Tab 50 MG |
| 55081077200 | Hydralazine HCl | Hydralazine HCl Tab 10 MG |
| 47679074535 | Hydralazine HCl | Hydralazine HCl Tab 100 MG |
| 50111039703 | Hydralazine HCl | Hydralazine HCl Tab 100 MG |
| 51079007419 | Hydralazine HCl | Hydralazine HCl Tab 10 MG |
| 51079007420 | Hydralazine HCl | Hydralazine HCl Tab 10 MG |
| 51079007440 | Hydralazine HCl | Hydralazine HCl Tab 10 MG |
| 51079007460 | Hydralazine HCl | Hydralazine HCl Tab 10 MG |
| 51079007519 | Hydralazine HCl | Hydralazine HCl Tab 25 MG |
| 51079007520 | Hydralazine HCl | Hydralazine HCl Tab 25 MG |
| 51079007524 | Hydralazine HCl | Hydralazine HCl Tab 25 MG |
| 51079007540 | Hydralazine HCl | Hydralazine HCl Tab 25 MG |
| 51079007560 | Hydralazine HCl | Hydralazine HCl Tab 25 MG |
| 51079007619 | Hydralazine HCl | Hydralazine HCl Tab 50 MG |
| 51079007620 | Hydralazine HCl | Hydralazine HCl Tab 50 MG |
| 51079007640 | Hydralazine HCl | Hydralazine HCl Tab 50 MG |
| 51079007660 | Hydralazine HCl | Hydralazine HCl Tab 50 MG |
| 51079018320 | Hydralazine HCl | Hydralazine HCl Tab 100 MG |
| 51079018340 | Hydralazine HCl | Hydralazine HCl Tab 100 MG |
| 51079018360 | Hydralazine HCl | Hydralazine HCl Tab 100 MG |
| 51432020200 | Hydralazine HCl | Hydralazine HCl Tab 50 MG |
| 51432020206 | Hydralazine HCl | Hydralazine HCl Tab 50 MG |
| 51432020503 | Hydralazine HCl | Hydralazine HCl Tab 100 MG |
| 51432019803 | Hydralazine HCl | Hydralazine HCl Tab 10 MG |
| 51432019806 | Hydralazine HCl | Hydralazine HCl Tab 10 MG |
| 51432020000 | Hydralazine HCl | Hydralazine HCl Tab 25 MG |
| 51432020003 | Hydralazine HCl | Hydralazine HCl Tab 25 MG |
| 51432020006 | Hydralazine HCl | Hydralazine HCl Tab 25 MG |
| 51728002410 | Hydralazine HCl | Hydralazine HCl Tab 10 MG |
| 51728002501 | Hydralazine HCl | Hydralazine HCl Tab 25 MG |
| 51728002510 | Hydralazine HCl | Hydralazine HCl Tab 25 MG |
| 51728002601 | Hydralazine HCl | Hydralazine HCl Tab 50 MG |
| 51728002610 | Hydralazine HCl | Hydralazine HCl Tab 50 MG |
| 52152002802 | Hydralazine HCl | Hydralazine HCl Tab 25 MG |
| 52152002805 | Hydralazine HCl | Hydralazine HCl Tab 25 MG |
| 52152002902 | Hydralazine HCl | Hydralazine HCl Tab 50 MG |
| 52152002905 | Hydralazine HCl | Hydralazine HCl Tab 50 MG |
| 51728064701 | Hydralazine HCl | Hydralazine HCl Tab 100 MG |
| 52446020321 | Hydralazine HCl | Hydralazine HCl Tab 10 MG |
| 52446020332 | Hydralazine HCl | Hydralazine HCl Tab 10 MG |
| 52446020421 | Hydralazine HCl | Hydralazine HCl Tab 25 MG |
| 52446020432 | Hydralazine HCl | Hydralazine HCl Tab 25 MG |
| 52446020521 | Hydralazine HCl | Hydralazine HCl Tab 50 MG |
| 52446020532 | Hydralazine HCl | Hydralazine HCl Tab 50 MG |
| 00879053810 | Hydralazine HCl | Hydralazine HCl Tab 100 MG |
| 00904516980 | Hydralazine HCl | Hydralazine HCl Tab 10 MG |
| 00904517260 | Hydralazine HCl | Hydralazine HCl Tab 100 MG |
| 00904233860 | Hydralazine HCl | Hydralazine HCl Tab 10 MG |
| 00904233861 | Hydralazine HCl | Hydralazine HCl Tab 10 MG |
| 00904233980 | Hydralazine HCl | Hydralazine HCl Tab 25 MG |
| 00904234160 | Hydralazine HCl | Hydralazine HCl Tab 100 MG |
| 00904333980 | Hydralazine HCl | Hydralazine HCl Tab 25 MG |
| 00904334080 | Hydralazine HCl | Hydralazine HCl Tab 50 MG |
| 17236016301 | Hydralazine HCl | Hydralazine HCl Tab 10 MG |
| 17236016310 | Hydralazine HCl | Hydralazine HCl Tab 10 MG |
| 17236016401 | Hydralazine HCl | Hydralazine HCl Tab 25 MG |
| 17236016410 | Hydralazine HCl | Hydralazine HCl Tab 25 MG |
| 17236016601 | Hydralazine HCl | Hydralazine HCl Tab 50 MG |
| 17236016610 | Hydralazine HCl | Hydralazine HCl Tab 50 MG |
| 17236024901 | Hydralazine HCl | Hydralazine HCl Tab 100 MG |
| 17236024910 | Hydralazine HCl | Hydralazine HCl Tab 100 MG |
| 39769002101 | Hydralazine HCl | Hydralazine HCl Inj 20 MG/ML |
| 39769002102 | Hydralazine HCl | Hydralazine HCl Inj 20 MG/ML |
| 40893060401 | Hydralazine HCl | Hydralazine HCl Tab 25 MG |
| 40893060501 | Hydralazine HCl | Hydralazine HCl Tab 50 MG |
| 47202206603 | Hydralazine HCl | Hydralazine HCl Tab 25 MG |
| 47202206703 | Hydralazine HCl | Hydralazine HCl Tab 50 MG |
| 47202226503 | Hydralazine HCl | Hydralazine HCl Tab 10 MG |
| 47679014101 | Hydralazine HCl | Hydralazine HCl Tab 25 MG |
| 47679014104 | Hydralazine HCl | Hydralazine HCl Tab 25 MG |
| 47679014135 | Hydralazine HCl | Hydralazine HCl Tab 25 MG |
| 47679014201 | Hydralazine HCl | Hydralazine HCl Tab 50 MG |
| 47679014204 | Hydralazine HCl | Hydralazine HCl Tab 50 MG |
| 47679014235 | Hydralazine HCl | Hydralazine HCl Tab 50 MG |
| 47202248603 | Hydralazine HCl | Hydralazine HCl Tab 100 MG |
| 47679074401 | Hydralazine HCl | Hydralazine HCl Tab 10 MG |
| 47679074435 | Hydralazine HCl | Hydralazine HCl Tab 10 MG |
| 47679074501 | Hydralazine HCl | Hydralazine HCl Tab 100 MG |
| 00779056025 | Hydralazine HCl | Hydralazine HCl Tab 25 MG |
| 00779056030 | Hydralazine HCl | Hydralazine HCl Tab 25 MG |
| 00781173201 | Hydralazine HCl | Hydralazine HCl Tab 10 MG |
| 00781173210 | Hydralazine HCl | Hydralazine HCl Tab 10 MG |
| 00781173301 | Hydralazine HCl | Hydralazine HCl Tab 25 MG |
| 00781173310 | Hydralazine HCl | Hydralazine HCl Tab 25 MG |
| 00781173313 | Hydralazine HCl | Hydralazine HCl Tab 25 MG |
| 00781173401 | Hydralazine HCl | Hydralazine HCl Tab 50 MG |
| 00781173410 | Hydralazine HCl | Hydralazine HCl Tab 50 MG |
| 00781173413 | Hydralazine HCl | Hydralazine HCl Tab 50 MG |
| 00783090109 | Hydralazine HCl | Hydralazine HCl Tab 25 MG |
| 00783090112 | Hydralazine HCl | Hydralazine HCl Tab 25 MG |
| 00783090209 | Hydralazine HCl | Hydralazine HCl Tab 50 MG |
| 00783090212 | Hydralazine HCl | Hydralazine HCl Tab 50 MG |
| 00783150109 | Hydralazine HCl | Hydralazine HCl Tab 10 MG |
| 00783150112 | Hydralazine HCl | Hydralazine HCl Tab 10 MG |
| 00814370714 | Hydralazine HCl | Hydralazine HCl Tab 10 MG |
| 00814370730 | Hydralazine HCl | Hydralazine HCl Tab 10 MG |
| 00814370914 | Hydralazine HCl | Hydralazine HCl Tab 25 MG |
| 00814370930 | Hydralazine HCl | Hydralazine HCl Tab 25 MG |
| 00814371014 | Hydralazine HCl | Hydralazine HCl Tab 50 MG |
| 00814371030 | Hydralazine HCl | Hydralazine HCl Tab 50 MG |
| 00814371214 | Hydralazine HCl | Hydralazine HCl Tab 100 MG |
| 00814371230 | Hydralazine HCl | Hydralazine HCl Tab 100 MG |
| 00832060013 | Hydralazine HCl | Hydralazine HCl Tab 25 MG |
| 00832060113 | Hydralazine HCl | Hydralazine HCl Tab 50 MG |
| 00839611406 | Hydralazine HCl | Hydralazine HCl Tab 10 MG |
| 00839611416 | Hydralazine HCl | Hydralazine HCl Tab 10 MG |
| 00839136106 | Hydralazine HCl | Hydralazine HCl Tab 25 MG |
| 00839136116 | Hydralazine HCl | Hydralazine HCl Tab 25 MG |
| 00839136306 | Hydralazine HCl | Hydralazine HCl Tab 50 MG |
| 00839136316 | Hydralazine HCl | Hydralazine HCl Tab 50 MG |
| 00839676106 | Hydralazine HCl | Hydralazine HCl Tab 100 MG |
| 00879053501 | Hydralazine HCl | Hydralazine HCl Tab 10 MG |
| 00879053510 | Hydralazine HCl | Hydralazine HCl Tab 10 MG |
| 00879053601 | Hydralazine HCl | Hydralazine HCl Tab 25 MG |
| 00879053610 | Hydralazine HCl | Hydralazine HCl Tab 25 MG |
| 00879053701 | Hydralazine HCl | Hydralazine HCl Tab 50 MG |
| 00879053710 | Hydralazine HCl | Hydralazine HCl Tab 50 MG |
| 00879053801 | Hydralazine HCl | Hydralazine HCl Tab 100 MG |
| 00580017401 | Hydralazine HCl | Hydralazine HCl Tab 25 MG |
| 00580017410 | Hydralazine HCl | Hydralazine HCl Tab 25 MG |
| 00591505001 | Hydralazine HCl | Hydralazine HCl Tab 25 MG |
| 00591505004 | Hydralazine HCl | Hydralazine HCl Tab 25 MG |
| 00591505501 | Hydralazine HCl | Hydralazine HCl Tab 50 MG |
| 00591505504 | Hydralazine HCl | Hydralazine HCl Tab 50 MG |
| 00615051601 | Hydralazine HCl | Hydralazine HCl Tab 10 MG |
| 00615051610 | Hydralazine HCl | Hydralazine HCl Tab 10 MG |
| 00615051613 | Hydralazine HCl | Hydralazine HCl Tab 10 MG |
| 00615051632 | Hydralazine HCl | Hydralazine HCl Tab 10 MG |
| 00615051639 | Hydralazine HCl | Hydralazine HCl Tab 10 MG |
| 00615053101 | Hydralazine HCl | Hydralazine HCl Tab 25 MG |
| 00615053110 | Hydralazine HCl | Hydralazine HCl Tab 25 MG |
| 00615053113 | Hydralazine HCl | Hydralazine HCl Tab 25 MG |
| 00615053132 | Hydralazine HCl | Hydralazine HCl Tab 25 MG |
| 00615053139 | Hydralazine HCl | Hydralazine HCl Tab 25 MG |
| 00615053201 | Hydralazine HCl | Hydralazine HCl Tab 50 MG |
| 00615053210 | Hydralazine HCl | Hydralazine HCl Tab 50 MG |
| 00615053213 | Hydralazine HCl | Hydralazine HCl Tab 50 MG |
| 00615053229 | Hydralazine HCl | Hydralazine HCl Tab 50 MG |
| 00677044701 | Hydralazine HCl | Hydralazine HCl Tab 25 MG |
| 00677044710 | Hydralazine HCl | Hydralazine HCl Tab 25 MG |
| 00677045101 | Hydralazine HCl | Hydralazine HCl Tab 50 MG |
| 00677045110 | Hydralazine HCl | Hydralazine HCl Tab 50 MG |
| 00677065001 | Hydralazine HCl | Hydralazine HCl Tab 10 MG |
| 00677065010 | Hydralazine HCl | Hydralazine HCl Tab 10 MG |
| 00677092201 | Hydralazine HCl | Hydralazine HCl Tab 100 MG |
| 00677092210 | Hydralazine HCl | Hydralazine HCl Tab 100 MG |
| 00719140010 | Hydralazine HCl | Hydralazine HCl Tab 10 MG |
| 00719140013 | Hydralazine HCl | Hydralazine HCl Tab 10 MG |
| 00719140110 | Hydralazine HCl | Hydralazine HCl Tab 25 MG |
| 00719140113 | Hydralazine HCl | Hydralazine HCl Tab 25 MG |
| 00719140210 | Hydralazine HCl | Hydralazine HCl Tab 50 MG |
| 00719140213 | Hydralazine HCl | Hydralazine HCl Tab 50 MG |
| 00719140310 | Hydralazine HCl | Hydralazine HCl Tab 100 MG |
| 00527115701 | Hydralazine HCl | Hydralazine HCl Tab 50 MG |
| 00527115710 | Hydralazine HCl | Hydralazine HCl Tab 50 MG |
| 00527118701 | Hydralazine HCl | Hydralazine HCl Tab 25 MG |
| 00527118710 | Hydralazine HCl | Hydralazine HCl Tab 25 MG |
| 00527122301 | Hydralazine HCl | Hydralazine HCl Tab 10 MG |
| 00527122310 | Hydralazine HCl | Hydralazine HCl Tab 10 MG |
| 00536386201 | Hydralazine HCl | Hydralazine HCl Tab 25 MG |
| 00536386210 | Hydralazine HCl | Hydralazine HCl Tab 25 MG |
| 00536386301 | Hydralazine HCl | Hydralazine HCl Tab 50 MG |
| 00536386310 | Hydralazine HCl | Hydralazine HCl Tab 50 MG |
| 00536389001 | Hydralazine HCl | Hydralazine HCl Tab 10 MG |
| 00536389010 | Hydralazine HCl | Hydralazine HCl Tab 10 MG |
| 00536389101 | Hydralazine HCl | Hydralazine HCl Tab 100 MG |
| 00536389110 | Hydralazine HCl | Hydralazine HCl Tab 100 MG |
| 00536391101 | Hydralazine HCl | Hydralazine HCl Tab 50 MG |
| 00536391110 | Hydralazine HCl | Hydralazine HCl Tab 50 MG |
| 00536391701 | Hydralazine HCl | Hydralazine HCl Tab 25 MG |
| 00536391710 | Hydralazine HCl | Hydralazine HCl Tab 25 MG |
| 00537209910 | Hydralazine HCl | Hydralazine HCl Tab 25 MG |
| 00537210001 | Hydralazine HCl | Hydralazine HCl Tab 10 MG |
| 00537210010 | Hydralazine HCl | Hydralazine HCl Tab 10 MG |
| 00537224310 | Hydralazine HCl | Hydralazine HCl Tab 50 MG |
| 00555006402 | Hydralazine HCl | Hydralazine HCl Tab 25 MG |
| 00555006405 | Hydralazine HCl | Hydralazine HCl Tab 25 MG |
| 00555006502 | Hydralazine HCl | Hydralazine HCl Tab 50 MG |
| 00555006505 | Hydralazine HCl | Hydralazine HCl Tab 50 MG |
| 00555038802 | Hydralazine HCl | Hydralazine HCl Tab 10 MG |
| 00555038805 | Hydralazine HCl | Hydralazine HCl Tab 10 MG |
| 00555038902 | Hydralazine HCl | Hydralazine HCl Tab 100 MG |
| 00556016201 | Hydralazine HCl | Hydralazine HCl Tab 10 MG |
| 00556016210 | Hydralazine HCl | Hydralazine HCl Tab 10 MG |
| 00556016301 | Hydralazine HCl | Hydralazine HCl Tab 25 MG |
| 00556016310 | Hydralazine HCl | Hydralazine HCl Tab 25 MG |
| 00556016401 | Hydralazine HCl | Hydralazine HCl Tab 50 MG |
| 00556016410 | Hydralazine HCl | Hydralazine HCl Tab 50 MG |
| 00580017201 | Hydralazine HCl | Hydralazine HCl Tab 10 MG |
| 00580017210 | Hydralazine HCl | Hydralazine HCl Tab 10 MG |
| 00580017301 | Hydralazine HCl | Hydralazine HCl Tab 50 MG |
| 00580017310 | Hydralazine HCl | Hydralazine HCl Tab 50 MG |
| 00304013600 | Hydralazine HCl | Hydralazine HCl Tab 10 MG |
| 00304013601 | Hydralazine HCl | Hydralazine HCl Tab 10 MG |
| 00304013700 | Hydralazine HCl | Hydralazine HCl Tab 25 MG |
| 00304013701 | Hydralazine HCl | Hydralazine HCl Tab 25 MG |
| 00304013800 | Hydralazine HCl | Hydralazine HCl Tab 50 MG |
| 00304013801 | Hydralazine HCl | Hydralazine HCl Tab 50 MG |
| 00304121400 | Hydralazine HCl | Hydralazine HCl Tab 100 MG |
| 00304121401 | Hydralazine HCl | Hydralazine HCl Tab 100 MG |
| 00339514512 | Hydralazine HCl | Hydralazine HCl Tab 25 MG |
| 00339514712 | Hydralazine HCl | Hydralazine HCl Tab 50 MG |
| 00349207801 | Hydralazine HCl | Hydralazine HCl Tab 25 MG |
| 00349207810 | Hydralazine HCl | Hydralazine HCl Tab 25 MG |
| 00349231201 | Hydralazine HCl | Hydralazine HCl Tab 50 MG |
| 00349231210 | Hydralazine HCl | Hydralazine HCl Tab 50 MG |
| 00349233801 | Hydralazine HCl | Hydralazine HCl Tab 10 MG |
| 00349233810 | Hydralazine HCl | Hydralazine HCl Tab 10 MG |
| 00364064701 | Hydralazine HCl | Hydralazine HCl Tab 10 MG |
| 00364064702 | Hydralazine HCl | Hydralazine HCl Tab 10 MG |
| 00364069601 | Hydralazine HCl | Hydralazine HCl Tab 100 MG |
| 00364014401 | Hydralazine HCl | Hydralazine HCl Tab 25 MG |
| 00364014402 | Hydralazine HCl | Hydralazine HCl Tab 25 MG |
| 00364014501 | Hydralazine HCl | Hydralazine HCl Tab 50 MG |
| 00364014502 | Hydralazine HCl | Hydralazine HCl Tab 50 MG |
| 00367221996 | Hydralazine HCl | Hydralazine HCl Tab 25 MG |
| 00385205501 | Hydralazine HCl | Hydralazine HCl Tab 10 MG |
| 00385205510 | Hydralazine HCl | Hydralazine HCl Tab 10 MG |
| 00385205601 | Hydralazine HCl | Hydralazine HCl Tab 25 MG |
| 00385205610 | Hydralazine HCl | Hydralazine HCl Tab 25 MG |
| 00385205701 | Hydralazine HCl | Hydralazine HCl Tab 50 MG |
| 00385205710 | Hydralazine HCl | Hydralazine HCl Tab 50 MG |
| 00405446901 | Hydralazine HCl | Hydralazine HCl Tab 10 MG |
| 00405446903 | Hydralazine HCl | Hydralazine HCl Tab 10 MG |
| 00405447001 | Hydralazine HCl | Hydralazine HCl Tab 25 MG |
| 00405447003 | Hydralazine HCl | Hydralazine HCl Tab 25 MG |
| 00405447101 | Hydralazine HCl | Hydralazine HCl Tab 50 MG |
| 00405447103 | Hydralazine HCl | Hydralazine HCl Tab 50 MG |
| 00405447201 | Hydralazine HCl | Hydralazine HCl Tab 100 MG |
| 00469249000 | Hydralazine HCl | Hydralazine HCl Inj 20 MG/ML |
| 00157048110 | Hydralazine HCl | Hydralazine HCl Tab 50 MG |
| 00147025510 | Hydralazine HCl | Hydralazine HCl Tab 10 MG |
| 00147025520 | Hydralazine HCl | Hydralazine HCl Tab 10 MG |
| 00147025610 | Hydralazine HCl | Hydralazine HCl Tab 25 MG |
| 00147025620 | Hydralazine HCl | Hydralazine HCl Tab 25 MG |
| 00147025710 | Hydralazine HCl | Hydralazine HCl Tab 50 MG |
| 00147025720 | Hydralazine HCl | Hydralazine HCl Tab 50 MG |
| 00147025810 | Hydralazine HCl | Hydralazine HCl Tab 100 MG |
| 00147025820 | Hydralazine HCl | Hydralazine HCl Tab 100 MG |
| 00172233860 | Hydralazine HCl | Hydralazine HCl Tab 10 MG |
| 00172233880 | Hydralazine HCl | Hydralazine HCl Tab 10 MG |
| 00172233960 | Hydralazine HCl | Hydralazine HCl Tab 25 MG |
| 00172233980 | Hydralazine HCl | Hydralazine HCl Tab 25 MG |
| 00172249360 | Hydralazine HCl | Hydralazine HCl Tab 50 MG |
| 00172249380 | Hydralazine HCl | Hydralazine HCl Tab 50 MG |
| 00182055401 | Hydralazine HCl | Hydralazine HCl Tab 25 MG |
| 00182055407 | Hydralazine HCl | Hydralazine HCl Tab 25 MG |
| 00182055410 | Hydralazine HCl | Hydralazine HCl Tab 25 MG |
| 00182055429 | Hydralazine HCl | Hydralazine HCl Tab 25 MG |
| 00182055501 | Hydralazine HCl | Hydralazine HCl Tab 50 MG |
| 00182055510 | Hydralazine HCl | Hydralazine HCl Tab 50 MG |
| 00182090501 | Hydralazine HCl | Hydralazine HCl Tab 10 MG |
| 00182090510 | Hydralazine HCl | Hydralazine HCl Tab 10 MG |
| 00182155301 | Hydralazine HCl | Hydralazine HCl Tab 100 MG |
| 00223106001 | Hydralazine HCl | Hydralazine HCl Tab 10 MG |
| 00223106002 | Hydralazine HCl | Hydralazine HCl Tab 10 MG |
| 00223106100 | Hydralazine HCl | Hydralazine HCl Tab 25 MG |
| 00223106101 | Hydralazine HCl | Hydralazine HCl Tab 25 MG |
| 00223106102 | Hydralazine HCl | Hydralazine HCl Tab 25 MG |
| 00223106200 | Hydralazine HCl | Hydralazine HCl Tab 50 MG |
| 00223106201 | Hydralazine HCl | Hydralazine HCl Tab 50 MG |
| 00223106202 | Hydralazine HCl | Hydralazine HCl Tab 50 MG |
| 00228221910 | Hydralazine HCl | Hydralazine HCl Tab 25 MG |
| 00228221996 | Hydralazine HCl | Hydralazine HCl Tab 25 MG |
| 00228222010 | Hydralazine HCl | Hydralazine HCl Tab 50 MG |
| 00228222096 | Hydralazine HCl | Hydralazine HCl Tab 50 MG |
| 00302328001 | Hydralazine HCl | Hydralazine HCl Tab 10 MG |
| 00302328010 | Hydralazine HCl | Hydralazine HCl Tab 10 MG |
| 00302328201 | Hydralazine HCl | Hydralazine HCl Tab 25 MG |
| 00302328210 | Hydralazine HCl | Hydralazine HCl Tab 25 MG |
| 00302328401 | Hydralazine HCl | Hydralazine HCl Tab 50 MG |
| 00302328410 | Hydralazine HCl | Hydralazine HCl Tab 50 MG |
| 00302328601 | Hydralazine HCl | Hydralazine HCl Tab 100 MG |
| 68258908501 | Hydralazine HCl | Hydralazine HCl Tab 10 MG |
| 68258903301 | Hydralazine HCl | Hydralazine HCl Tab 100 MG |
| 54868053601 | Hydralazine HCl | Hydralazine HCl Tab 100 MG |
| 54868578800 | Hydralazine HCl | Hydralazine HCl Tab 10 MG |
| 00005356434 | Hydralazine HCl | Hydralazine HCl Tab 25 MG |
| 00005356462 | Hydralazine HCl | Hydralazine HCl Tab 25 MG |
| 00005356534 | Hydralazine HCl | Hydralazine HCl Tab 50 MG |
| 00005356562 | Hydralazine HCl | Hydralazine HCl Tab 50 MG |
| 00083003730 | Hydralazine HCl | Hydralazine HCl Tab 10 MG |
| 00083003740 | Hydralazine HCl | Hydralazine HCl Tab 10 MG |
| 00083003765 | Hydralazine HCl | Hydralazine HCl Tab 10 MG |
| 00083003930 | Hydralazine HCl | Hydralazine HCl Tab 25 MG |
| 00083003932 | Hydralazine HCl | Hydralazine HCl Tab 25 MG |
| 00083003940 | Hydralazine HCl | Hydralazine HCl Tab 25 MG |
| 00083003965 | Hydralazine HCl | Hydralazine HCl Tab 25 MG |
| 00083007330 | Hydralazine HCl | Hydralazine HCl Tab 50 MG |
| 00083007332 | Hydralazine HCl | Hydralazine HCl Tab 50 MG |
| 00083007340 | Hydralazine HCl | Hydralazine HCl Tab 50 MG |
| 00083007365 | Hydralazine HCl | Hydralazine HCl Tab 50 MG |
| 00083010130 | Hydralazine HCl | Hydralazine HCl Tab 100 MG |
| 00083262605 | Hydralazine HCl | Hydralazine HCl Inj 20 MG/ML |
| 00102160705 | Hydralazine HCl | Hydralazine HCl Tab 25 MG |
| 00102160805 | Hydralazine HCl | Hydralazine HCl Tab 50 MG |
| 00102343001 | Hydralazine HCl | Hydralazine HCl Tab 10 MG |
| 00143126925 | Hydralazine HCl | Hydralazine HCl Tab 25 MG |
| 00143127125 | Hydralazine HCl | Hydralazine HCl Tab 50 MG |
| 00115366001 | Hydralazine HCl | Hydralazine HCl Tab 25 MG |
| 00115366002 | Hydralazine HCl | Hydralazine HCl Tab 25 MG |
| 00115366003 | Hydralazine HCl | Hydralazine HCl Tab 25 MG |
| 00115366201 | Hydralazine HCl | Hydralazine HCl Tab 50 MG |
| 00115366202 | Hydralazine HCl | Hydralazine HCl Tab 50 MG |
| 00115366203 | Hydralazine HCl | Hydralazine HCl Tab 50 MG |
| 00144114503 | Hydralazine HCl | Hydralazine HCl Tab 25 MG |
| 00144114603 | Hydralazine HCl | Hydralazine HCl Tab 50 MG |
| 00157047901 | Hydralazine HCl | Hydralazine HCl Tab 10 MG |
| 00157047910 | Hydralazine HCl | Hydralazine HCl Tab 10 MG |
| 00157048001 | Hydralazine HCl | Hydralazine HCl Tab 25 MG |
| 00157048010 | Hydralazine HCl | Hydralazine HCl Tab 25 MG |
| 00157048101 | Hydralazine HCl | Hydralazine HCl Tab 50 MG |
| 54868336900 | Hydralazine HCl | Hydralazine HCl Tab 50 MG |
| 55289013301 | Hydralazine HCl | Hydralazine HCl Tab 25 MG |
| 55289013390 | Hydralazine HCl | Hydralazine HCl Tab 25 MG |
| 55289013397 | Hydralazine HCl | Hydralazine HCl Tab 25 MG |
| 55289013490 | Hydralazine HCl | Hydralazine HCl Tab 50 MG |
| 55289032501 | Hydralazine HCl | Hydralazine HCl Tab 10 MG |
| 55887056830 | Hydralazine HCl | Hydralazine HCl Tab 10 MG |
| 55887056860 | Hydralazine HCl | Hydralazine HCl Tab 10 MG |
| 55887056890 | Hydralazine HCl | Hydralazine HCl Tab 10 MG |
| 55887059230 | Hydralazine HCl | Hydralazine HCl Tab 25 MG |
| 55887059260 | Hydralazine HCl | Hydralazine HCl Tab 25 MG |
| 55887059290 | Hydralazine HCl | Hydralazine HCl Tab 25 MG |
| 55887059330 | Hydralazine HCl | Hydralazine HCl Tab 50 MG |
| 55887059360 | Hydralazine HCl | Hydralazine HCl Tab 50 MG |
| 55887059390 | Hydralazine HCl | Hydralazine HCl Tab 50 MG |
| 58016050500 | Hydralazine HCl | Hydralazine HCl Tab 10 MG |
| 58016050512 | Hydralazine HCl | Hydralazine HCl Tab 10 MG |
| 58016050515 | Hydralazine HCl | Hydralazine HCl Tab 10 MG |
| 58016050520 | Hydralazine HCl | Hydralazine HCl Tab 10 MG |
| 58016050530 | Hydralazine HCl | Hydralazine HCl Tab 10 MG |
| 58016050600 | Hydralazine HCl | Hydralazine HCl Tab 25 MG |
| 58016050612 | Hydralazine HCl | Hydralazine HCl Tab 25 MG |
| 58016050615 | Hydralazine HCl | Hydralazine HCl Tab 25 MG |
| 58016050620 | Hydralazine HCl | Hydralazine HCl Tab 25 MG |
| 58016050630 | Hydralazine HCl | Hydralazine HCl Tab 25 MG |
| 58016050700 | Hydralazine HCl | Hydralazine HCl Tab 50 MG |
| 58016050712 | Hydralazine HCl | Hydralazine HCl Tab 50 MG |
| 58016050715 | Hydralazine HCl | Hydralazine HCl Tab 50 MG |
| 58016050720 | Hydralazine HCl | Hydralazine HCl Tab 50 MG |
| 58016050730 | Hydralazine HCl | Hydralazine HCl Tab 50 MG |
| 58864013301 | Hydralazine HCl | Hydralazine HCl Tab 25 MG |
| 58864013390 | Hydralazine HCl | Hydralazine HCl Tab 25 MG |
| 58864013490 | Hydralazine HCl | Hydralazine HCl Tab 50 MG |
| 58864032501 | Hydralazine HCl | Hydralazine HCl Tab 10 MG |
| 58864064128 | Hydralazine HCl | Hydralazine HCl Tab 10 MG |
| 60346082330 | Hydralazine HCl | Hydralazine HCl Tab 10 MG |
| 60346082360 | Hydralazine HCl | Hydralazine HCl Tab 10 MG |
| 60346082430 | Hydralazine HCl | Hydralazine HCl Tab 25 MG |
| 60346082460 | Hydralazine HCl | Hydralazine HCl Tab 25 MG |
| 60346082560 | Hydralazine HCl | Hydralazine HCl Tab 50 MG |
| 60346082590 | Hydralazine HCl | Hydralazine HCl Tab 50 MG |
| 63323061401 | Hydralazine HCl | Hydralazine HCl Inj 20 MG/ML |
| 12280030930 | Hydralazine HCl | Hydralazine HCl Tab 50 MG |
| 12280030960 | Hydralazine HCl | Hydralazine HCl Tab 50 MG |
| 12280031030 | Hydralazine HCl | Hydralazine HCl Tab 100 MG |
| 12280031060 | Hydralazine HCl | Hydralazine HCl Tab 100 MG |
| 23155000201 | Hydralazine HCl | Hydralazine HCl Tab 25 MG |
| 23155000210 | Hydralazine HCl | Hydralazine HCl Tab 25 MG |
| 23155000301 | Hydralazine HCl | Hydralazine HCl Tab 50 MG |
| 23155000310 | Hydralazine HCl | Hydralazine HCl Tab 50 MG |
| 49999077700 | Hydralazine HCl | Hydralazine HCl Tab 50 MG |
| 54868053600 | Hydralazine HCl | Hydralazine HCl Tab 100 MG |
| 54868194901 | Hydralazine HCl | Hydralazine HCl Tab 25 MG |
| 54868289301 | Hydralazine HCl | Hydralazine HCl Tab 50 MG |
| 55045379301 | Hydralazine HCl | Hydralazine HCl Tab 50 MG |
| 58016003600 | Hydralazine HCl | Hydralazine HCl Tab 50 MG |
| 58016003630 | Hydralazine HCl | Hydralazine HCl Tab 50 MG |
| 58016003660 | Hydralazine HCl | Hydralazine HCl Tab 50 MG |
| 58016003690 | Hydralazine HCl | Hydralazine HCl Tab 50 MG |
| 63323061455 | Hydralazine HCl | Hydralazine HCl Inj 20 MG/ML |
| 68258905301 | Hydralazine HCl | Hydralazine HCl Tab 25 MG |
| 68258905401 | Hydralazine HCl | Hydralazine HCl Tab 50 MG |
| 00182055489 | Hydralazine HCl | Hydralazine HCl Tab 25 MG |
| 00182055589 | Hydralazine HCl | Hydralazine HCl Tab 50 MG |
| 00182090589 | Hydralazine HCl | Hydralazine HCl Tab 10 MG |
| 00440758030 | Hydralazine HCl | Hydralazine HCl Tab 10 MG |
| 00440758090 | Hydralazine HCl | Hydralazine HCl Tab 10 MG |
| 00440758130 | Hydralazine HCl | Hydralazine HCl Tab 25 MG |
| 00440758190 | Hydralazine HCl | Hydralazine HCl Tab 25 MG |
| 00440758292 | Hydralazine HCl | Hydralazine HCl Tab 50 MG |
| 00517090125 | Hydralazine HCl | Hydralazine HCl Inj 20 MG/ML |
| 00517091125 | Hydralazine HCl | Hydralazine HCl Inj 20 MG/ML |
| 00603383021 | Hydralazine HCl | Hydralazine HCl Tab 10 MG |
| 00603383032 | Hydralazine HCl | Hydralazine HCl Tab 10 MG |
| 00603383121 | Hydralazine HCl | Hydralazine HCl Tab 25 MG |
| 00603383132 | Hydralazine HCl | Hydralazine HCl Tab 25 MG |
| 00603383221 | Hydralazine HCl | Hydralazine HCl Tab 50 MG |
| 00603383232 | Hydralazine HCl | Hydralazine HCl Tab 50 MG |
| 00603383321 | Hydralazine HCl | Hydralazine HCl Tab 100 MG |
| 00703820101 | Hydralazine HCl | Hydralazine HCl Inj 20 MG/ML |
| 00703820104 | Hydralazine HCl | Hydralazine HCl Inj 20 MG/ML |
| 00703820194 | Hydralazine HCl | Hydralazine HCl Inj 20 MG/ML |
| 00904233880 | Hydralazine HCl | Hydralazine HCl Tab 10 MG |
| 00904233960 | Hydralazine HCl | Hydralazine HCl Tab 25 MG |
| 00904233961 | Hydralazine HCl | Hydralazine HCl Tab 25 MG |
| 00904234060 | Hydralazine HCl | Hydralazine HCl Tab 50 MG |
| 00904234061 | Hydralazine HCl | Hydralazine HCl Tab 50 MG |
| 00904234080 | Hydralazine HCl | Hydralazine HCl Tab 50 MG |
| 00904516960 | Hydralazine HCl | Hydralazine HCl Tab 10 MG |
| 00904517060 | Hydralazine HCl | Hydralazine HCl Tab 25 MG |
| 00904517080 | Hydralazine HCl | Hydralazine HCl Tab 25 MG |
| 00904517160 | Hydralazine HCl | Hydralazine HCl Tab 50 MG |
| 00904517180 | Hydralazine HCl | Hydralazine HCl Tab 50 MG |
| 49884012101 | Hydralazine HCl | Hydralazine HCl Tab 100 MG |
| 49884012110 | Hydralazine HCl | Hydralazine HCl Tab 100 MG |
| 49884002701 | Hydralazine HCl | Hydralazine HCl Tab 25 MG |
| 49884002710 | Hydralazine HCl | Hydralazine HCl Tab 25 MG |
| 49884002801 | Hydralazine HCl | Hydralazine HCl Tab 50 MG |
| 49884002810 | Hydralazine HCl | Hydralazine HCl Tab 50 MG |
| 49884002901 | Hydralazine HCl | Hydralazine HCl Tab 10 MG |
| 49884002910 | Hydralazine HCl | Hydralazine HCl Tab 10 MG |
| 50111032701 | Hydralazine HCl | Hydralazine HCl Tab 25 MG |
| 50111032703 | Hydralazine HCl | Hydralazine HCl Tab 25 MG |
| 50111032801 | Hydralazine HCl | Hydralazine HCl Tab 50 MG |
| 50111032803 | Hydralazine HCl | Hydralazine HCl Tab 50 MG |
| 50111039701 | Hydralazine HCl | Hydralazine HCl Tab 100 MG |
| 50111039801 | Hydralazine HCl | Hydralazine HCl Tab 10 MG |
| 50111039803 | Hydralazine HCl | Hydralazine HCl Tab 10 MG |
| 54569051500 | Hydralazine HCl | Hydralazine HCl Tab 25 MG |
| 54569051501 | Hydralazine HCl | Hydralazine HCl Tab 25 MG |
| 54569051701 | Hydralazine HCl | Hydralazine HCl Tab 50 MG |
| 54738032701 | Hydralazine HCl | Hydralazine HCl Tab 25 MG |
| 54738032703 | Hydralazine HCl | Hydralazine HCl Tab 25 MG |
| 54738032801 | Hydralazine HCl | Hydralazine HCl Tab 50 MG |
| 54738032803 | Hydralazine HCl | Hydralazine HCl Tab 50 MG |
| 54738039801 | Hydralazine HCl | Hydralazine HCl Tab 10 MG |
| 54738039803 | Hydralazine HCl | Hydralazine HCl Tab 10 MG |
| 54868194900 | Hydralazine HCl | Hydralazine HCl Tab 25 MG |
| 54868289300 | Hydralazine HCl | Hydralazine HCl Tab 50 MG |
| 35356042830 | Irbesartan | Irbesartan Tab 150 MG |
| 54868419902 | Irbesartan | Irbesartan Tab 150 MG |
| 63629337301 | Irbesartan | Irbesartan Tab 150 MG |
| 63629337302 | Irbesartan | Irbesartan Tab 150 MG |
| 63629337303 | Irbesartan | Irbesartan Tab 150 MG |
| 63629337304 | Irbesartan | Irbesartan Tab 150 MG |
| 47463037530 | Irbesartan | Irbesartan Tab 150 MG |
| 47463037630 | Irbesartan | Irbesartan Tab 300 MG |
| 54868441401 | Irbesartan | Irbesartan Tab 300 MG |
| 23490010703 | Irbesartan | Irbesartan Tab 75 MG |
| 23490010803 | Irbesartan | Irbesartan Tab 150 MG |
| 23490010909 | Irbesartan | Irbesartan Tab 300 MG |
| 51138021730 | Irbesartan | Irbesartan Tab 75 MG |
| 51138021760 | Irbesartan | Irbesartan Tab 75 MG |
| 51138021830 | Irbesartan | Irbesartan Tab 150 MG |
| 51138021860 | Irbesartan | Irbesartan Tab 150 MG |
| 51138021930 | Irbesartan | Irbesartan Tab 300 MG |
| 51138021960 | Irbesartan | Irbesartan Tab 300 MG |
| 55048037530 | Irbesartan | Irbesartan Tab 150 MG |
| 55048037630 | Irbesartan | Irbesartan Tab 300 MG |
| 00093746456 | Irbesartan | Irbesartan Tab 75 MG |
| 00093746498 | Irbesartan | Irbesartan Tab 75 MG |
| 00093746505 | Irbesartan | Irbesartan Tab 150 MG |
| 00093746556 | Irbesartan | Irbesartan Tab 150 MG |
| 00093746598 | Irbesartan | Irbesartan Tab 150 MG |
| 00093746605 | Irbesartan | Irbesartan Tab 300 MG |
| 00093746656 | Irbesartan | Irbesartan Tab 300 MG |
| 00093746698 | Irbesartan | Irbesartan Tab 300 MG |
| 00955104030 | Irbesartan | Irbesartan Tab 75 MG |
| 00955104090 | Irbesartan | Irbesartan Tab 75 MG |
| 00955104130 | Irbesartan | Irbesartan Tab 150 MG |
| 00955104190 | Irbesartan | Irbesartan Tab 150 MG |
| 00955104230 | Irbesartan | Irbesartan Tab 300 MG |
| 00955104290 | Irbesartan | Irbesartan Tab 300 MG |
| 68258606603 | Irbesartan | Irbesartan Tab 150 MG |
| 68258606606 | Irbesartan | Irbesartan Tab 150 MG |
| 54569630900 | Irbesartan | Irbesartan Tab 150 MG |
| 00087277131 | Irbesartan | Irbesartan Tab 75 MG |
| 00087277132 | Irbesartan | Irbesartan Tab 75 MG |
| 00087277215 | Irbesartan | Irbesartan Tab 150 MG |
| 00087277231 | Irbesartan | Irbesartan Tab 150 MG |
| 00087277232 | Irbesartan | Irbesartan Tab 150 MG |
| 00087277235 | Irbesartan | Irbesartan Tab 150 MG |
| 00087277315 | Irbesartan | Irbesartan Tab 300 MG |
| 00087277331 | Irbesartan | Irbesartan Tab 300 MG |
| 00087277332 | Irbesartan | Irbesartan Tab 300 MG |
| 12280006390 | Irbesartan | Irbesartan Tab 150 MG |
| 54569489500 | Irbesartan | Irbesartan Tab 300 MG |
| 54569457200 | Irbesartan | Irbesartan Tab 150 MG |
| 54868419900 | Irbesartan | Irbesartan Tab 150 MG |
| 54868419901 | Irbesartan | Irbesartan Tab 150 MG |
| 54868441400 | Irbesartan | Irbesartan Tab 300 MG |
| 58864081790 | Irbesartan | Irbesartan Tab 150 MG |
| 58864072615 | Irbesartan | Irbesartan Tab 150 MG |
| 58864072630 | Irbesartan | Irbesartan Tab 150 MG |
| 58864077115 | Irbesartan | Irbesartan Tab 300 MG |
| 12280006330 | Irbesartan | Irbesartan Tab 150 MG |
| 13411010601 | Irbesartan | Irbesartan Tab 150 MG |
| 13411010603 | Irbesartan | Irbesartan Tab 150 MG |
| 13411010606 | Irbesartan | Irbesartan Tab 150 MG |
| 13411010609 | Irbesartan | Irbesartan Tab 150 MG |
| 13411010615 | Irbesartan | Irbesartan Tab 150 MG |
| 13411010701 | Irbesartan | Irbesartan Tab 300 MG |
| 13411010703 | Irbesartan | Irbesartan Tab 300 MG |
| 13411010706 | Irbesartan | Irbesartan Tab 300 MG |
| 13411010709 | Irbesartan | Irbesartan Tab 300 MG |
| 13411010715 | Irbesartan | Irbesartan Tab 300 MG |
| 12280036930 | Irbesartan | Irbesartan Tab 300 MG |
| 12280036990 | Irbesartan | Irbesartan Tab 300 MG |
| 54569586700 | Irbesartan | Irbesartan Tab 75 MG |
| 66105050301 | Irbesartan | Irbesartan Tab 150 MG |
| 66105050303 | Irbesartan | Irbesartan Tab 150 MG |
| 66105050306 | Irbesartan | Irbesartan Tab 150 MG |
| 66105050309 | Irbesartan | Irbesartan Tab 150 MG |
| 66105050315 | Irbesartan | Irbesartan Tab 150 MG |
| 66105050401 | Irbesartan | Irbesartan Tab 300 MG |
| 66105050403 | Irbesartan | Irbesartan Tab 300 MG |
| 66105050406 | Irbesartan | Irbesartan Tab 300 MG |
| 66105050409 | Irbesartan | Irbesartan Tab 300 MG |
| 66105050415 | Irbesartan | Irbesartan Tab 300 MG |
| 67544040045 | Irbesartan | Irbesartan Tab 300 MG |
| 00087277115 | Irbesartan | Irbesartan Tab 75 MG |
| 00087277135 | Irbesartan | Irbesartan Tab 75 MG |
| 00087277335 | Irbesartan | Irbesartan Tab 300 MG |
| 55175535503 | Irbesartan | Irbesartan Tab 150 MG |
| 35356013130 | Irbesartan | Irbesartan Tab 300 MG |
| 35356037430 | Irbesartan-Hydrochlorothiazide | Irbesartan-Hydrochlorothiazide Tab 150-12.5 MG |
| 35356040630 | Irbesartan-Hydrochlorothiazide | Irbesartan-Hydrochlorothiazide Tab 300-12.5 MG |
| 35356040730 | Irbesartan-Hydrochlorothiazide | Irbesartan-Hydrochlorothiazide Tab 300-25 MG |
| 63629337401 | Irbesartan-Hydrochlorothiazide | Irbesartan-Hydrochlorothiazide Tab 150-12.5 MG |
| 63629337402 | Irbesartan-Hydrochlorothiazide | Irbesartan-Hydrochlorothiazide Tab 150-12.5 MG |
| 63629337403 | Irbesartan-Hydrochlorothiazide | Irbesartan-Hydrochlorothiazide Tab 150-12.5 MG |
| 63629337404 | Irbesartan-Hydrochlorothiazide | Irbesartan-Hydrochlorothiazide Tab 150-12.5 MG |
| 54868546501 | Irbesartan-Hydrochlorothiazide | Irbesartan-Hydrochlorothiazide Tab 300-25 MG |
| 00087287531 | Irbesartan-Hydrochlorothiazide | Irbesartan-Hydrochlorothiazide Tab 150-12.5 MG |
| 00087287532 | Irbesartan-Hydrochlorothiazide | Irbesartan-Hydrochlorothiazide Tab 150-12.5 MG |
| 00087287631 | Irbesartan-Hydrochlorothiazide | Irbesartan-Hydrochlorothiazide Tab 300-12.5 MG |
| 00087287632 | Irbesartan-Hydrochlorothiazide | Irbesartan-Hydrochlorothiazide Tab 300-12.5 MG |
| 51138023130 | Irbesartan-Hydrochlorothiazide | Irbesartan-Hydrochlorothiazide Tab 150-12.5 MG |
| 51138023230 | Irbesartan-Hydrochlorothiazide | Irbesartan-Hydrochlorothiazide Tab 300-12.5 MG |
| 00093723856 | Irbesartan-Hydrochlorothiazide | Irbesartan-Hydrochlorothiazide Tab 150-12.5 MG |
| 00093723898 | Irbesartan-Hydrochlorothiazide | Irbesartan-Hydrochlorothiazide Tab 150-12.5 MG |
| 00093723956 | Irbesartan-Hydrochlorothiazide | Irbesartan-Hydrochlorothiazide Tab 300-12.5 MG |
| 00093723998 | Irbesartan-Hydrochlorothiazide | Irbesartan-Hydrochlorothiazide Tab 300-12.5 MG |
| 00955104530 | Irbesartan-Hydrochlorothiazide | Irbesartan-Hydrochlorothiazide Tab 150-12.5 MG |
| 00955104590 | Irbesartan-Hydrochlorothiazide | Irbesartan-Hydrochlorothiazide Tab 150-12.5 MG |
| 00955104630 | Irbesartan-Hydrochlorothiazide | Irbesartan-Hydrochlorothiazide Tab 300-12.5 MG |
| 00955104690 | Irbesartan-Hydrochlorothiazide | Irbesartan-Hydrochlorothiazide Tab 300-12.5 MG |
| 00087277531 | Irbesartan-Hydrochlorothiazide | Irbesartan-Hydrochlorothiazide Tab 150-12.5 MG |
| 00087277532 | Irbesartan-Hydrochlorothiazide | Irbesartan-Hydrochlorothiazide Tab 150-12.5 MG |
| 00087277631 | Irbesartan-Hydrochlorothiazide | Irbesartan-Hydrochlorothiazide Tab 300-12.5 MG |
| 00087277632 | Irbesartan-Hydrochlorothiazide | Irbesartan-Hydrochlorothiazide Tab 300-12.5 MG |
| 00087278831 | Irbesartan-Hydrochlorothiazide | Irbesartan-Hydrochlorothiazide Tab 300-25 MG |
| 00087278832 | Irbesartan-Hydrochlorothiazide | Irbesartan-Hydrochlorothiazide Tab 300-25 MG |
| 54868449400 | Irbesartan-Hydrochlorothiazide | Irbesartan-Hydrochlorothiazide Tab 150-12.5 MG |
| 54868452600 | Irbesartan-Hydrochlorothiazide | Irbesartan-Hydrochlorothiazide Tab 300-12.5 MG |
| 54868452601 | Irbesartan-Hydrochlorothiazide | Irbesartan-Hydrochlorothiazide Tab 300-12.5 MG |
| 12280037030 | Irbesartan-Hydrochlorothiazide | Irbesartan-Hydrochlorothiazide Tab 150-12.5 MG |
| 12280037090 | Irbesartan-Hydrochlorothiazide | Irbesartan-Hydrochlorothiazide Tab 150-12.5 MG |
| 12280037130 | Irbesartan-Hydrochlorothiazide | Irbesartan-Hydrochlorothiazide Tab 300-12.5 MG |
| 12280037190 | Irbesartan-Hydrochlorothiazide | Irbesartan-Hydrochlorothiazide Tab 300-12.5 MG |
| 54868546500 | Irbesartan-Hydrochlorothiazide | Irbesartan-Hydrochlorothiazide Tab 300-25 MG |
| 35356037415 | Irbesartan-Hydrochlorothiazide | Irbesartan-Hydrochlorothiazide Tab 150-12.5 MG |
| 00006001528 | Lisinopril | Lisinopril Tab 2.5 MG |
| 00006001531 | Lisinopril | Lisinopril Tab 2.5 MG |
| 00006001558 | Lisinopril | Lisinopril Tab 2.5 MG |
| 00006001928 | Lisinopril | Lisinopril Tab 5 MG |
| 00006001954 | Lisinopril | Lisinopril Tab 5 MG |
| 00006001958 | Lisinopril | Lisinopril Tab 5 MG |
| 00006001972 | Lisinopril | Lisinopril Tab 5 MG |
| 00006001982 | Lisinopril | Lisinopril Tab 5 MG |
| 00006001986 | Lisinopril | Lisinopril Tab 5 MG |
| 00006001987 | Lisinopril | Lisinopril Tab 5 MG |
| 00006001994 | Lisinopril | Lisinopril Tab 5 MG |
| 00006010628 | Lisinopril | Lisinopril Tab 10 MG |
| 00006010631 | Lisinopril | Lisinopril Tab 10 MG |
| 00006010654 | Lisinopril | Lisinopril Tab 10 MG |
| 00006010658 | Lisinopril | Lisinopril Tab 10 MG |
| 00006010672 | Lisinopril | Lisinopril Tab 10 MG |
| 00006010682 | Lisinopril | Lisinopril Tab 10 MG |
| 00006010687 | Lisinopril | Lisinopril Tab 10 MG |
| 00006010694 | Lisinopril | Lisinopril Tab 10 MG |
| 00006020728 | Lisinopril | Lisinopril Tab 20 MG |
| 00006020731 | Lisinopril | Lisinopril Tab 20 MG |
| 00006020754 | Lisinopril | Lisinopril Tab 20 MG |
| 00006020758 | Lisinopril | Lisinopril Tab 20 MG |
| 00006020772 | Lisinopril | Lisinopril Tab 20 MG |
| 00006020782 | Lisinopril | Lisinopril Tab 20 MG |
| 00006020787 | Lisinopril | Lisinopril Tab 20 MG |
| 00006020794 | Lisinopril | Lisinopril Tab 20 MG |
| 00006023758 | Lisinopril | Lisinopril Tab 40 MG |
| 00093515701 | Lisinopril | Lisinopril Tab 30 MG |
| 00093111101 | Lisinopril | Lisinopril Tab 2.5 MG |
| 00093111201 | Lisinopril | Lisinopril Tab 5 MG |
| 00093111210 | Lisinopril | Lisinopril Tab 5 MG |
| 00093111301 | Lisinopril | Lisinopril Tab 10 MG |
| 00093111310 | Lisinopril | Lisinopril Tab 10 MG |
| 00093111401 | Lisinopril | Lisinopril Tab 20 MG |
| 00093111410 | Lisinopril | Lisinopril Tab 20 MG |
| 00093111501 | Lisinopril | Lisinopril Tab 40 MG |
| 00093111505 | Lisinopril | Lisinopril Tab 40 MG |
| 00143126501 | Lisinopril | Lisinopril Tab 2.5 MG |
| 00143126510 | Lisinopril | Lisinopril Tab 2.5 MG |
| 00143126601 | Lisinopril | Lisinopril Tab 5 MG |
| 00143126610 | Lisinopril | Lisinopril Tab 5 MG |
| 00143126701 | Lisinopril | Lisinopril Tab 10 MG |
| 00143126710 | Lisinopril | Lisinopril Tab 10 MG |
| 00143126801 | Lisinopril | Lisinopril Tab 20 MG |
| 00143126810 | Lisinopril | Lisinopril Tab 20 MG |
| 00143127001 | Lisinopril | Lisinopril Tab 40 MG |
| 00143127010 | Lisinopril | Lisinopril Tab 40 MG |
| 00172375710 | Lisinopril | Lisinopril Tab 2.5 MG |
| 00172375760 | Lisinopril | Lisinopril Tab 2.5 MG |
| 00172375770 | Lisinopril | Lisinopril Tab 2.5 MG |
| 00172375810 | Lisinopril | Lisinopril Tab 5 MG |
| 00172375860 | Lisinopril | Lisinopril Tab 5 MG |
| 00172375870 | Lisinopril | Lisinopril Tab 5 MG |
| 00172375880 | Lisinopril | Lisinopril Tab 5 MG |
| 00172375910 | Lisinopril | Lisinopril Tab 10 MG |
| 00172375960 | Lisinopril | Lisinopril Tab 10 MG |
| 00172375970 | Lisinopril | Lisinopril Tab 10 MG |
| 00172375980 | Lisinopril | Lisinopril Tab 10 MG |
| 00172375985 | Lisinopril | Lisinopril Tab 10 MG |
| 00172376010 | Lisinopril | Lisinopril Tab 20 MG |
| 00172376060 | Lisinopril | Lisinopril Tab 20 MG |
| 00172376070 | Lisinopril | Lisinopril Tab 20 MG |
| 00172376080 | Lisinopril | Lisinopril Tab 20 MG |
| 00172376085 | Lisinopril | Lisinopril Tab 20 MG |
| 00172376110 | Lisinopril | Lisinopril Tab 40 MG |
| 00172376160 | Lisinopril | Lisinopril Tab 40 MG |
| 00172376170 | Lisinopril | Lisinopril Tab 40 MG |
| 00172376180 | Lisinopril | Lisinopril Tab 40 MG |
| 00172376210 | Lisinopril | Lisinopril Tab 30 MG |
| 00172376260 | Lisinopril | Lisinopril Tab 30 MG |
| 00172376270 | Lisinopril | Lisinopril Tab 30 MG |
| 00185540001 | Lisinopril | Lisinopril Tab 5 MG |
| 00185540010 | Lisinopril | Lisinopril Tab 5 MG |
| 00185540033 | Lisinopril | Lisinopril Tab 5 MG |
| 00185002501 | Lisinopril | Lisinopril Tab 2.5 MG |
| 00185002510 | Lisinopril | Lisinopril Tab 2.5 MG |
| 00185010101 | Lisinopril | Lisinopril Tab 10 MG |
| 00185010110 | Lisinopril | Lisinopril Tab 10 MG |
| 00185010133 | Lisinopril | Lisinopril Tab 10 MG |
| 00185010201 | Lisinopril | Lisinopril Tab 20 MG |
| 00185010210 | Lisinopril | Lisinopril Tab 20 MG |
| 00185010233 | Lisinopril | Lisinopril Tab 20 MG |
| 00185010301 | Lisinopril | Lisinopril Tab 30 MG |
| 00185010310 | Lisinopril | Lisinopril Tab 30 MG |
| 00185010401 | Lisinopril | Lisinopril Tab 40 MG |
| 00185010410 | Lisinopril | Lisinopril Tab 40 MG |
| 00247102300 | Lisinopril | Lisinopril Tab 20 MG |
| 00247102330 | Lisinopril | Lisinopril Tab 20 MG |
| 00247102360 | Lisinopril | Lisinopril Tab 20 MG |
| 00247139630 | Lisinopril | Lisinopril Tab 2.5 MG |
| 00247114710 | Lisinopril | Lisinopril Tab 10 MG |
| 00247114730 | Lisinopril | Lisinopril Tab 10 MG |
| 00247114760 | Lisinopril | Lisinopril Tab 10 MG |
| 00310013010 | Lisinopril | Lisinopril Tab 5 MG |
| 00310013034 | Lisinopril | Lisinopril Tab 5 MG |
| 00310013039 | Lisinopril | Lisinopril Tab 5 MG |
| 00310013110 | Lisinopril | Lisinopril Tab 10 MG |
| 00310013134 | Lisinopril | Lisinopril Tab 10 MG |
| 00310013139 | Lisinopril | Lisinopril Tab 10 MG |
| 00310013173 | Lisinopril | Lisinopril Tab 10 MG |
| 00310013210 | Lisinopril | Lisinopril Tab 20 MG |
| 00310013234 | Lisinopril | Lisinopril Tab 20 MG |
| 00310013239 | Lisinopril | Lisinopril Tab 20 MG |
| 00310013273 | Lisinopril | Lisinopril Tab 20 MG |
| 00310013310 | Lisinopril | Lisinopril Tab 30 MG |
| 00310013410 | Lisinopril | Lisinopril Tab 40 MG |
| 00310013510 | Lisinopril | Lisinopril Tab 2.5 MG |
| 00378207201 | Lisinopril | Lisinopril Tab 2.5 MG |
| 00378207301 | Lisinopril | Lisinopril Tab 5 MG |
| 00378207310 | Lisinopril | Lisinopril Tab 5 MG |
| 00378207401 | Lisinopril | Lisinopril Tab 10 MG |
| 00378207410 | Lisinopril | Lisinopril Tab 10 MG |
| 00378207501 | Lisinopril | Lisinopril Tab 20 MG |
| 00378207510 | Lisinopril | Lisinopril Tab 20 MG |
| 00378207601 | Lisinopril | Lisinopril Tab 40 MG |
| 00378207701 | Lisinopril | Lisinopril Tab 30 MG |
| 00591040501 | Lisinopril | Lisinopril Tab 2.5 MG |
| 00591040505 | Lisinopril | Lisinopril Tab 2.5 MG |
| 00591040601 | Lisinopril | Lisinopril Tab 5 MG |
| 00591040610 | Lisinopril | Lisinopril Tab 5 MG |
| 00591040701 | Lisinopril | Lisinopril Tab 10 MG |
| 00591040710 | Lisinopril | Lisinopril Tab 10 MG |
| 00591040801 | Lisinopril | Lisinopril Tab 20 MG |
| 00591040810 | Lisinopril | Lisinopril Tab 20 MG |
| 00591040901 | Lisinopril | Lisinopril Tab 40 MG |
| 00591040905 | Lisinopril | Lisinopril Tab 40 MG |
| 00591088501 | Lisinopril | Lisinopril Tab 30 MG |
| 00781166501 | Lisinopril | Lisinopril Tab 5 MG |
| 00781166601 | Lisinopril | Lisinopril Tab 10 MG |
| 00781166701 | Lisinopril | Lisinopril Tab 20 MG |
| 00781166801 | Lisinopril | Lisinopril Tab 40 MG |
| 00781166901 | Lisinopril | Lisinopril Tab 2.5 MG |
| 00781167301 | Lisinopril | Lisinopril Tab 30 MG |
| 00904563761 | Lisinopril | Lisinopril Tab 2.5 MG |
| 00904563843 | Lisinopril | Lisinopril Tab 5 MG |
| 00904563846 | Lisinopril | Lisinopril Tab 5 MG |
| 00904563861 | Lisinopril | Lisinopril Tab 5 MG |
| 00904563961 | Lisinopril | Lisinopril Tab 10 MG |
| 00904564043 | Lisinopril | Lisinopril Tab 20 MG |
| 00904564061 | Lisinopril | Lisinopril Tab 20 MG |
| 00904564093 | Lisinopril | Lisinopril Tab 20 MG |
| 00904564161 | Lisinopril | Lisinopril Tab 30 MG |
| 00904564243 | Lisinopril | Lisinopril Tab 40 MG |
| 00904564246 | Lisinopril | Lisinopril Tab 40 MG |
| 00904564248 | Lisinopril | Lisinopril Tab 40 MG |
| 00904564252 | Lisinopril | Lisinopril Tab 40 MG |
| 00904564261 | Lisinopril | Lisinopril Tab 40 MG |
| 00904564293 | Lisinopril | Lisinopril Tab 40 MG |
| 12280012960 | Lisinopril | Lisinopril Tab 30 MG |
| 12280014400 | Lisinopril | Lisinopril Tab 20 MG |
| 44514048018 | Lisinopril | Lisinopril Tab 5 MG |
| 44514048118 | Lisinopril | Lisinopril Tab 10 MG |
| 44514048218 | Lisinopril | Lisinopril Tab 20 MG |
| 44514048318 | Lisinopril | Lisinopril Tab 40 MG |
| 49884055601 | Lisinopril | Lisinopril Tab 2.5 MG |
| 49884055610 | Lisinopril | Lisinopril Tab 2.5 MG |
| 49884055701 | Lisinopril | Lisinopril Tab 5 MG |
| 49884055710 | Lisinopril | Lisinopril Tab 5 MG |
| 49884055801 | Lisinopril | Lisinopril Tab 10 MG |
| 49884055810 | Lisinopril | Lisinopril Tab 10 MG |
| 49884055901 | Lisinopril | Lisinopril Tab 20 MG |
| 49884055910 | Lisinopril | Lisinopril Tab 20 MG |
| 49999018360 | Lisinopril | Lisinopril Tab 20 MG |
| 49884056001 | Lisinopril | Lisinopril Tab 40 MG |
| 49884056010 | Lisinopril | Lisinopril Tab 40 MG |
| 49884063501 | Lisinopril | Lisinopril Tab 30 MG |
| 49999029530 | Lisinopril | Lisinopril Tab 5 MG |
| 49884063510 | Lisinopril | Lisinopril Tab 30 MG |
| 49999018230 | Lisinopril | Lisinopril Tab 10 MG |
| 49999018260 | Lisinopril | Lisinopril Tab 10 MG |
| 49999018330 | Lisinopril | Lisinopril Tab 20 MG |
| 49999046910 | Lisinopril | Lisinopril Tab 40 MG |
| 51079098120 | Lisinopril | Lisinopril Tab 5 MG |
| 51079098157 | Lisinopril | Lisinopril Tab 5 MG |
| 51079098219 | Lisinopril | Lisinopril Tab 10 MG |
| 51079098220 | Lisinopril | Lisinopril Tab 10 MG |
| 51079098257 | Lisinopril | Lisinopril Tab 10 MG |
| 51079098319 | Lisinopril | Lisinopril Tab 20 MG |
| 51079098320 | Lisinopril | Lisinopril Tab 20 MG |
| 51079098357 | Lisinopril | Lisinopril Tab 20 MG |
| 51079098420 | Lisinopril | Lisinopril Tab 40 MG |
| 52959072830 | Lisinopril | Lisinopril Tab 10 MG |
| 52959072930 | Lisinopril | Lisinopril Tab 20 MG |
| 54569175203 | Lisinopril | Lisinopril Tab 10 MG |
| 54569205101 | Lisinopril | Lisinopril Tab 20 MG |
| 54569377100 | Lisinopril | Lisinopril Tab 5 MG |
| 54569330000 | Lisinopril | Lisinopril Tab 5 MG |
| 54569330001 | Lisinopril | Lisinopril Tab 5 MG |
| 54569266501 | Lisinopril | Lisinopril Tab 20 MG |
| 54569266502 | Lisinopril | Lisinopril Tab 20 MG |
| 54569194400 | Lisinopril | Lisinopril Tab 10 MG |
| 54569194401 | Lisinopril | Lisinopril Tab 10 MG |
| 54569194402 | Lisinopril | Lisinopril Tab 10 MG |
| 54569194403 | Lisinopril | Lisinopril Tab 10 MG |
| 54569459600 | Lisinopril | Lisinopril Tab 40 MG |
| 54569472100 | Lisinopril | Lisinopril Tab 2.5 MG |
| 54569537900 | Lisinopril | Lisinopril Tab 2.5 MG |
| 54569543400 | Lisinopril | Lisinopril Tab 10 MG |
| 54569543500 | Lisinopril | Lisinopril Tab 20 MG |
| 54569543700 | Lisinopril | Lisinopril Tab 2.5 MG |
| 54569543800 | Lisinopril | Lisinopril Tab 5 MG |
| 54569547200 | Lisinopril | Lisinopril Tab 40 MG |
| 54868129602 | Lisinopril | Lisinopril Tab 10 MG |
| 54868150100 | Lisinopril | Lisinopril Tab 40 MG |
| 54868150101 | Lisinopril | Lisinopril Tab 40 MG |
| 54868150200 | Lisinopril | Lisinopril Tab 20 MG |
| 54868100101 | Lisinopril | Lisinopril Tab 20 MG |
| 54868196000 | Lisinopril | Lisinopril Tab 5 MG |
| 54868196101 | Lisinopril | Lisinopril Tab 5 MG |
| 54868196102 | Lisinopril | Lisinopril Tab 5 MG |
| 54868197001 | Lisinopril | Lisinopril Tab 10 MG |
| 54868197002 | Lisinopril | Lisinopril Tab 10 MG |
| 54868197003 | Lisinopril | Lisinopril Tab 10 MG |
| 54868129601 | Lisinopril | Lisinopril Tab 10 MG |
| 54868464600 | Lisinopril | Lisinopril Tab 40 MG |
| 54868464602 | Lisinopril | Lisinopril Tab 40 MG |
| 54868465600 | Lisinopril | Lisinopril Tab 2.5 MG |
| 54868465700 | Lisinopril | Lisinopril Tab 10 MG |
| 54868465701 | Lisinopril | Lisinopril Tab 10 MG |
| 54868465702 | Lisinopril | Lisinopril Tab 10 MG |
| 54868465800 | Lisinopril | Lisinopril Tab 20 MG |
| 54868465801 | Lisinopril | Lisinopril Tab 20 MG |
| 54868467800 | Lisinopril | Lisinopril Tab 5 MG |
| 54868467801 | Lisinopril | Lisinopril Tab 5 MG |
| 54868478000 | Lisinopril | Lisinopril Tab 30 MG |
| 55289010630 | Lisinopril | Lisinopril Tab 20 MG |
| 55289050930 | Lisinopril | Lisinopril Tab 10 MG |
| 55289057730 | Lisinopril | Lisinopril Tab 20 MG |
| 55289063801 | Lisinopril | Lisinopril Tab 10 MG |
| 55289063830 | Lisinopril | Lisinopril Tab 10 MG |
| 55289069614 | Lisinopril | Lisinopril Tab 20 MG |
| 55289092908 | Lisinopril | Lisinopril Tab 10 MG |
| 55289092930 | Lisinopril | Lisinopril Tab 10 MG |
| 55887056930 | Lisinopril | Lisinopril Tab 40 MG |
| 55887056960 | Lisinopril | Lisinopril Tab 40 MG |
| 55887056982 | Lisinopril | Lisinopril Tab 40 MG |
| 55887056990 | Lisinopril | Lisinopril Tab 40 MG |
| 55887058130 | Lisinopril | Lisinopril Tab 20 MG |
| 55887058160 | Lisinopril | Lisinopril Tab 20 MG |
| 55887058182 | Lisinopril | Lisinopril Tab 20 MG |
| 55887058190 | Lisinopril | Lisinopril Tab 20 MG |
| 55887058930 | Lisinopril | Lisinopril Tab 5 MG |
| 55887058960 | Lisinopril | Lisinopril Tab 5 MG |
| 55887058982 | Lisinopril | Lisinopril Tab 5 MG |
| 55887058990 | Lisinopril | Lisinopril Tab 5 MG |
| 55887059030 | Lisinopril | Lisinopril Tab 2.5 MG |
| 55887059060 | Lisinopril | Lisinopril Tab 2.5 MG |
| 55887059082 | Lisinopril | Lisinopril Tab 2.5 MG |
| 55887059090 | Lisinopril | Lisinopril Tab 2.5 MG |
| 55887059130 | Lisinopril | Lisinopril Tab 10 MG |
| 55887059160 | Lisinopril | Lisinopril Tab 10 MG |
| 55887059182 | Lisinopril | Lisinopril Tab 10 MG |
| 55887059190 | Lisinopril | Lisinopril Tab 10 MG |
| 57866500001 | Lisinopril | Lisinopril Tab 20 MG |
| 57866500002 | Lisinopril | Lisinopril Tab 20 MG |
| 57866600001 | Lisinopril | Lisinopril Tab 30 MG |
| 57866400001 | Lisinopril | Lisinopril Tab 10 MG |
| 57866400002 | Lisinopril | Lisinopril Tab 10 MG |
| 57866024501 | Lisinopril | Lisinopril Tab 5 MG |
| 57866024601 | Lisinopril | Lisinopril Tab 10 MG |
| 57866200001 | Lisinopril | Lisinopril Tab 5 MG |
| 57866200002 | Lisinopril | Lisinopril Tab 5 MG |
| 57866670002 | Lisinopril | Lisinopril Tab 10 MG |
| 57866670102 | Lisinopril | Lisinopril Tab 20 MG |
| 57866670501 | Lisinopril | Lisinopril Tab 10 MG |
| 57866670601 | Lisinopril | Lisinopril Tab 20 MG |
| 57866798501 | Lisinopril | Lisinopril Tab 5 MG |
| 57866798901 | Lisinopril | Lisinopril Tab 40 MG |
| 58016036214 | Lisinopril | Lisinopril Tab 10 MG |
| 58016036221 | Lisinopril | Lisinopril Tab 10 MG |
| 58016036228 | Lisinopril | Lisinopril Tab 10 MG |
| 58016036300 | Lisinopril | Lisinopril Tab 20 MG |
| 58016036314 | Lisinopril | Lisinopril Tab 20 MG |
| 58016036321 | Lisinopril | Lisinopril Tab 20 MG |
| 58016036330 | Lisinopril | Lisinopril Tab 20 MG |
| 58016036360 | Lisinopril | Lisinopril Tab 20 MG |
| 58016076000 | Lisinopril | Lisinopril Tab 20 MG |
| 58016076030 | Lisinopril | Lisinopril Tab 20 MG |
| 58016076060 | Lisinopril | Lisinopril Tab 20 MG |
| 58016076090 | Lisinopril | Lisinopril Tab 20 MG |
| 58016056400 | Lisinopril | Lisinopril Tab 5 MG |
| 58016056430 | Lisinopril | Lisinopril Tab 5 MG |
| 58016056460 | Lisinopril | Lisinopril Tab 5 MG |
| 58016056490 | Lisinopril | Lisinopril Tab 5 MG |
| 58016064600 | Lisinopril | Lisinopril Tab 40 MG |
| 58016064630 | Lisinopril | Lisinopril Tab 40 MG |
| 58016064660 | Lisinopril | Lisinopril Tab 40 MG |
| 58016064690 | Lisinopril | Lisinopril Tab 40 MG |
| 58016095600 | Lisinopril | Lisinopril Tab 5 MG |
| 58016095630 | Lisinopril | Lisinopril Tab 5 MG |
| 58016095660 | Lisinopril | Lisinopril Tab 5 MG |
| 58016095690 | Lisinopril | Lisinopril Tab 5 MG |
| 58016096300 | Lisinopril | Lisinopril Tab 10 MG |
| 58016096330 | Lisinopril | Lisinopril Tab 10 MG |
| 58016096360 | Lisinopril | Lisinopril Tab 10 MG |
| 58016096390 | Lisinopril | Lisinopril Tab 10 MG |
| 58016099800 | Lisinopril | Lisinopril Tab 20 MG |
| 58016099830 | Lisinopril | Lisinopril Tab 20 MG |
| 58016099860 | Lisinopril | Lisinopril Tab 20 MG |
| 58016099890 | Lisinopril | Lisinopril Tab 20 MG |
| 58016263814 | Lisinopril | Lisinopril Tab 5 MG |
| 58016263821 | Lisinopril | Lisinopril Tab 5 MG |
| 58016263830 | Lisinopril | Lisinopril Tab 5 MG |
| 58016263860 | Lisinopril | Lisinopril Tab 5 MG |
| 58016263890 | Lisinopril | Lisinopril Tab 5 MG |
| 58016091700 | Lisinopril | Lisinopril Tab 5 MG |
| 58016091730 | Lisinopril | Lisinopril Tab 5 MG |
| 58016091760 | Lisinopril | Lisinopril Tab 5 MG |
| 58016091790 | Lisinopril | Lisinopril Tab 5 MG |
| 58864060315 | Lisinopril | Lisinopril Tab 10 MG |
| 58864060330 | Lisinopril | Lisinopril Tab 10 MG |
| 58864060390 | Lisinopril | Lisinopril Tab 10 MG |
| 58864061830 | Lisinopril | Lisinopril Tab 40 MG |
| 58864065430 | Lisinopril | Lisinopril Tab 10 MG |
| 58864000630 | Lisinopril | Lisinopril Tab 20 MG |
| 58864075030 | Lisinopril | Lisinopril Tab 30 MG |
| 58864075330 | Lisinopril | Lisinopril Tab 5 MG |
| 58864075390 | Lisinopril | Lisinopril Tab 5 MG |
| 60346059530 | Lisinopril | Lisinopril Tab 40 MG |
| 60346087103 | Lisinopril | Lisinopril Tab 10 MG |
| 60346087130 | Lisinopril | Lisinopril Tab 10 MG |
| 60346097208 | Lisinopril | Lisinopril Tab 10 MG |
| 60346097230 | Lisinopril | Lisinopril Tab 10 MG |
| 60505018400 | Lisinopril | Lisinopril Tab 2.5 MG |
| 60505018401 | Lisinopril | Lisinopril Tab 2.5 MG |
| 60505018500 | Lisinopril | Lisinopril Tab 5 MG |
| 60505018501 | Lisinopril | Lisinopril Tab 5 MG |
| 60505018600 | Lisinopril | Lisinopril Tab 10 MG |
| 60505018601 | Lisinopril | Lisinopril Tab 10 MG |
| 60505018700 | Lisinopril | Lisinopril Tab 20 MG |
| 60505018701 | Lisinopril | Lisinopril Tab 20 MG |
| 60505018800 | Lisinopril | Lisinopril Tab 30 MG |
| 60505018801 | Lisinopril | Lisinopril Tab 30 MG |
| 60505018900 | Lisinopril | Lisinopril Tab 40 MG |
| 60505018901 | Lisinopril | Lisinopril Tab 40 MG |
| 63304053101 | Lisinopril | Lisinopril Tab 2.5 MG |
| 63304053201 | Lisinopril | Lisinopril Tab 5 MG |
| 63304053210 | Lisinopril | Lisinopril Tab 5 MG |
| 63304053301 | Lisinopril | Lisinopril Tab 10 MG |
| 63304053310 | Lisinopril | Lisinopril Tab 10 MG |
| 63304053401 | Lisinopril | Lisinopril Tab 20 MG |
| 63304053410 | Lisinopril | Lisinopril Tab 20 MG |
| 63304053501 | Lisinopril | Lisinopril Tab 40 MG |
| 63304053510 | Lisinopril | Lisinopril Tab 40 MG |
| 63739034815 | Lisinopril | Lisinopril Tab 5 MG |
| 63739034915 | Lisinopril | Lisinopril Tab 10 MG |
| 63739035015 | Lisinopril | Lisinopril Tab 20 MG |
| 63874055801 | Lisinopril | Lisinopril Tab 5 MG |
| 63874055810 | Lisinopril | Lisinopril Tab 5 MG |
| 63874055814 | Lisinopril | Lisinopril Tab 5 MG |
| 63874055816 | Lisinopril | Lisinopril Tab 5 MG |
| 63874055828 | Lisinopril | Lisinopril Tab 5 MG |
| 63874055830 | Lisinopril | Lisinopril Tab 5 MG |
| 63874055860 | Lisinopril | Lisinopril Tab 5 MG |
| 63874051401 | Lisinopril | Lisinopril Tab 20 MG |
| 63874051410 | Lisinopril | Lisinopril Tab 20 MG |
| 63874051414 | Lisinopril | Lisinopril Tab 20 MG |
| 63874051415 | Lisinopril | Lisinopril Tab 20 MG |
| 63874051416 | Lisinopril | Lisinopril Tab 20 MG |
| 63874051420 | Lisinopril | Lisinopril Tab 20 MG |
| 63874051428 | Lisinopril | Lisinopril Tab 20 MG |
| 63874051430 | Lisinopril | Lisinopril Tab 20 MG |
| 63874051460 | Lisinopril | Lisinopril Tab 20 MG |
| 63874051490 | Lisinopril | Lisinopril Tab 20 MG |
| 63874052201 | Lisinopril | Lisinopril Tab 10 MG |
| 63874052208 | Lisinopril | Lisinopril Tab 10 MG |
| 63874052210 | Lisinopril | Lisinopril Tab 10 MG |
| 63874052214 | Lisinopril | Lisinopril Tab 10 MG |
| 63874052215 | Lisinopril | Lisinopril Tab 10 MG |
| 63874052220 | Lisinopril | Lisinopril Tab 10 MG |
| 63874052221 | Lisinopril | Lisinopril Tab 10 MG |
| 63874052228 | Lisinopril | Lisinopril Tab 10 MG |
| 63874052230 | Lisinopril | Lisinopril Tab 10 MG |
| 63874052260 | Lisinopril | Lisinopril Tab 10 MG |
| 63874052290 | Lisinopril | Lisinopril Tab 10 MG |
| 66116027930 | Lisinopril | Lisinopril Tab 10 MG |
| 66685070301 | Lisinopril | Lisinopril Tab 10 MG |
| 66685070302 | Lisinopril | Lisinopril Tab 10 MG |
| 66685070303 | Lisinopril | Lisinopril Tab 10 MG |
| 66685070401 | Lisinopril | Lisinopril Tab 20 MG |
| 66685070402 | Lisinopril | Lisinopril Tab 20 MG |
| 66685070403 | Lisinopril | Lisinopril Tab 20 MG |
| 66685070501 | Lisinopril | Lisinopril Tab 30 MG |
| 66685070502 | Lisinopril | Lisinopril Tab 30 MG |
| 66685070601 | Lisinopril | Lisinopril Tab 40 MG |
| 66685070602 | Lisinopril | Lisinopril Tab 40 MG |
| 66685070603 | Lisinopril | Lisinopril Tab 40 MG |
| 66685070604 | Lisinopril | Lisinopril Tab 40 MG |
| 66685070101 | Lisinopril | Lisinopril Tab 2.5 MG |
| 66685070102 | Lisinopril | Lisinopril Tab 2.5 MG |
| 66685070201 | Lisinopril | Lisinopril Tab 5 MG |
| 66685070202 | Lisinopril | Lisinopril Tab 5 MG |
| 66685070203 | Lisinopril | Lisinopril Tab 5 MG |
| 68115036130 | Lisinopril | Lisinopril Tab 10 MG |
| 68115036200 | Lisinopril | Lisinopril Tab 20 MG |
| 68115036260 | Lisinopril | Lisinopril Tab 20 MG |
| 68115036330 | Lisinopril | Lisinopril Tab 5 MG |
| 68115039630 | Lisinopril | Lisinopril Tab 5 MG |
| 68115020730 | Lisinopril | Lisinopril Tab 10 MG |
| 68115020760 | Lisinopril | Lisinopril Tab 10 MG |
| 68115020830 | Lisinopril | Lisinopril Tab 20 MG |
| 68115020860 | Lisinopril | Lisinopril Tab 20 MG |
| 68115020890 | Lisinopril | Lisinopril Tab 20 MG |
| 68115020930 | Lisinopril | Lisinopril Tab 40 MG |
| 68030670002 | Lisinopril | Lisinopril Tab 10 MG |
| 68030670102 | Lisinopril | Lisinopril Tab 20 MG |
| 00172375800 | Lisinopril | Lisinopril Tab 5 MG |
| 00172375900 | Lisinopril | Lisinopril Tab 10 MG |
| 00172376000 | Lisinopril | Lisinopril Tab 20 MG |
| 00172376100 | Lisinopril | Lisinopril Tab 40 MG |
| 00440767590 | Lisinopril | Lisinopril Tab 10 MG |
| 00440767690 | Lisinopril | Lisinopril Tab 20 MG |
| 00904563889 | Lisinopril | Lisinopril Tab 5 MG |
| 00904563943 | Lisinopril | Lisinopril Tab 10 MG |
| 00904563946 | Lisinopril | Lisinopril Tab 10 MG |
| 00904563948 | Lisinopril | Lisinopril Tab 10 MG |
| 00904563989 | Lisinopril | Lisinopril Tab 10 MG |
| 00904563993 | Lisinopril | Lisinopril Tab 10 MG |
| 00904564046 | Lisinopril | Lisinopril Tab 20 MG |
| 00904564048 | Lisinopril | Lisinopril Tab 20 MG |
| 00904564089 | Lisinopril | Lisinopril Tab 20 MG |
| 00904564289 | Lisinopril | Lisinopril Tab 40 MG |
| 00904577889 | Lisinopril | Lisinopril Tab 2.5 MG |
| 16590035630 | Lisinopril | Lisinopril Tab 40 MG |
| 16590036030 | Lisinopril | Lisinopril Tab 40 MG |
| 16590036060 | Lisinopril | Lisinopril Tab 40 MG |
| 16590036090 | Lisinopril | Lisinopril Tab 40 MG |
| 23490581501 | Lisinopril | Lisinopril Tab 10 MG |
| 23490581601 | Lisinopril | Lisinopril Tab 20 MG |
| 23490581602 | Lisinopril | Lisinopril Tab 20 MG |
| 23490581702 | Lisinopril | Lisinopril Tab 2.5 MG |
| 23490581802 | Lisinopril | Lisinopril Tab 40 MG |
| 23490581902 | Lisinopril | Lisinopril Tab 5 MG |
| 21695032830 | Lisinopril | Lisinopril Tab 5 MG |
| 21695032930 | Lisinopril | Lisinopril Tab 10 MG |
| 21695033030 | Lisinopril | Lisinopril Tab 20 MG |
| 49999018310 | Lisinopril | Lisinopril Tab 20 MG |
| 49999029500 | Lisinopril | Lisinopril Tab 5 MG |
| 49999046930 | Lisinopril | Lisinopril Tab 40 MG |
| 49999087000 | Lisinopril | Lisinopril Tab 30 MG |
| 51079098101 | Lisinopril | Lisinopril Tab 5 MG |
| 51079098201 | Lisinopril | Lisinopril Tab 10 MG |
| 51079098217 | Lisinopril | Lisinopril Tab 10 MG |
| 51079098301 | Lisinopril | Lisinopril Tab 20 MG |
| 51079098317 | Lisinopril | Lisinopril Tab 20 MG |
| 51079098401 | Lisinopril | Lisinopril Tab 40 MG |
| 54569572800 | Lisinopril | Lisinopril Tab 30 MG |
| 52959072815 | Lisinopril | Lisinopril Tab 10 MG |
| 52959072820 | Lisinopril | Lisinopril Tab 10 MG |
| 52959075300 | Lisinopril | Lisinopril Tab 40 MG |
| 52959075330 | Lisinopril | Lisinopril Tab 40 MG |
| 52959085420 | Lisinopril | Lisinopril Tab 5 MG |
| 54569543403 | Lisinopril | Lisinopril Tab 10 MG |
| 54569543503 | Lisinopril | Lisinopril Tab 20 MG |
| 54868478001 | Lisinopril | Lisinopril Tab 30 MG |
| 54868464603 | Lisinopril | Lisinopril Tab 40 MG |
| 54868465601 | Lisinopril | Lisinopril Tab 2.5 MG |
| 54868465704 | Lisinopril | Lisinopril Tab 10 MG |
| 54868465802 | Lisinopril | Lisinopril Tab 20 MG |
| 55289091790 | Lisinopril | Lisinopril Tab 40 MG |
| 55045377208 | Lisinopril | Lisinopril Tab 20 MG |
| 55045292906 | Lisinopril | Lisinopril Tab 10 MG |
| 55045292908 | Lisinopril | Lisinopril Tab 10 MG |
| 55045293700 | Lisinopril | Lisinopril Tab 10 MG |
| 55045293800 | Lisinopril | Lisinopril Tab 5 MG |
| 55045293808 | Lisinopril | Lisinopril Tab 5 MG |
| 55045297500 | Lisinopril | Lisinopril Tab 40 MG |
| 55045297506 | Lisinopril | Lisinopril Tab 40 MG |
| 55045297508 | Lisinopril | Lisinopril Tab 40 MG |
| 55045305900 | Lisinopril | Lisinopril Tab 2.5 MG |
| 55045305908 | Lisinopril | Lisinopril Tab 2.5 MG |
| 55289063812 | Lisinopril | Lisinopril Tab 10 MG |
| 55289063814 | Lisinopril | Lisinopril Tab 10 MG |
| 55289063890 | Lisinopril | Lisinopril Tab 10 MG |
| 55289063898 | Lisinopril | Lisinopril Tab 10 MG |
| 55289069630 | Lisinopril | Lisinopril Tab 20 MG |
| 55289069690 | Lisinopril | Lisinopril Tab 20 MG |
| 55289069698 | Lisinopril | Lisinopril Tab 20 MG |
| 58016006900 | Lisinopril | Lisinopril Tab 30 MG |
| 58016006930 | Lisinopril | Lisinopril Tab 30 MG |
| 58016006960 | Lisinopril | Lisinopril Tab 30 MG |
| 58016006990 | Lisinopril | Lisinopril Tab 30 MG |
| 60505268808 | Lisinopril | Lisinopril Tab 40 MG |
| 60505268809 | Lisinopril | Lisinopril Tab 40 MG |
| 58864000063 | Lisinopril | Lisinopril Tab 20 MG |
| 60429072945 | Lisinopril | Lisinopril Tab 5 MG |
| 60429072990 | Lisinopril | Lisinopril Tab 5 MG |
| 60429073045 | Lisinopril | Lisinopril Tab 10 MG |
| 60429073090 | Lisinopril | Lisinopril Tab 10 MG |
| 60429073145 | Lisinopril | Lisinopril Tab 20 MG |
| 60429073190 | Lisinopril | Lisinopril Tab 20 MG |
| 60429073345 | Lisinopril | Lisinopril Tab 40 MG |
| 60429073390 | Lisinopril | Lisinopril Tab 40 MG |
| 58864061815 | Lisinopril | Lisinopril Tab 40 MG |
| 60505018507 | Lisinopril | Lisinopril Tab 5 MG |
| 60505018509 | Lisinopril | Lisinopril Tab 5 MG |
| 60505018607 | Lisinopril | Lisinopril Tab 10 MG |
| 60505018609 | Lisinopril | Lisinopril Tab 10 MG |
| 60505018707 | Lisinopril | Lisinopril Tab 20 MG |
| 60505018709 | Lisinopril | Lisinopril Tab 20 MG |
| 60505018908 | Lisinopril | Lisinopril Tab 40 MG |
| 60505018909 | Lisinopril | Lisinopril Tab 40 MG |
| 60505268301 | Lisinopril | Lisinopril Tab 2.5 MG |
| 60505268401 | Lisinopril | Lisinopril Tab 5 MG |
| 60505268408 | Lisinopril | Lisinopril Tab 5 MG |
| 60505268409 | Lisinopril | Lisinopril Tab 5 MG |
| 60505268501 | Lisinopril | Lisinopril Tab 10 MG |
| 60505268508 | Lisinopril | Lisinopril Tab 10 MG |
| 60505268509 | Lisinopril | Lisinopril Tab 10 MG |
| 60505268601 | Lisinopril | Lisinopril Tab 20 MG |
| 60505268608 | Lisinopril | Lisinopril Tab 20 MG |
| 60505268609 | Lisinopril | Lisinopril Tab 20 MG |
| 60505268801 | Lisinopril | Lisinopril Tab 40 MG |
| 65862003701 | Lisinopril | Lisinopril Tab 2.5 MG |
| 65862003705 | Lisinopril | Lisinopril Tab 2.5 MG |
| 65862003801 | Lisinopril | Lisinopril Tab 5 MG |
| 65862003805 | Lisinopril | Lisinopril Tab 5 MG |
| 65862003901 | Lisinopril | Lisinopril Tab 10 MG |
| 65862003905 | Lisinopril | Lisinopril Tab 10 MG |
| 65862004001 | Lisinopril | Lisinopril Tab 20 MG |
| 65862004101 | Lisinopril | Lisinopril Tab 30 MG |
| 65862004201 | Lisinopril | Lisinopril Tab 40 MG |
| 63304059901 | Lisinopril | Lisinopril Tab 30 MG |
| 64679092701 | Lisinopril | Lisinopril Tab 2.5 MG |
| 64679092702 | Lisinopril | Lisinopril Tab 2.5 MG |
| 64679092705 | Lisinopril | Lisinopril Tab 2.5 MG |
| 64679092801 | Lisinopril | Lisinopril Tab 5 MG |
| 64679092805 | Lisinopril | Lisinopril Tab 5 MG |
| 64679092806 | Lisinopril | Lisinopril Tab 5 MG |
| 64679092901 | Lisinopril | Lisinopril Tab 10 MG |
| 64679092905 | Lisinopril | Lisinopril Tab 10 MG |
| 64679092906 | Lisinopril | Lisinopril Tab 10 MG |
| 64679094101 | Lisinopril | Lisinopril Tab 20 MG |
| 64679094105 | Lisinopril | Lisinopril Tab 20 MG |
| 64679094106 | Lisinopril | Lisinopril Tab 20 MG |
| 64679094201 | Lisinopril | Lisinopril Tab 40 MG |
| 64679094202 | Lisinopril | Lisinopril Tab 40 MG |
| 64679094205 | Lisinopril | Lisinopril Tab 40 MG |
| 64679095301 | Lisinopril | Lisinopril Tab 30 MG |
| 64679095302 | Lisinopril | Lisinopril Tab 30 MG |
| 64679095305 | Lisinopril | Lisinopril Tab 30 MG |
| 63739034810 | Lisinopril | Lisinopril Tab 5 MG |
| 63739034910 | Lisinopril | Lisinopril Tab 10 MG |
| 63739035010 | Lisinopril | Lisinopril Tab 20 MG |
| 65243030303 | Lisinopril | Lisinopril Tab 5 MG |
| 65243030309 | Lisinopril | Lisinopril Tab 5 MG |
| 65243030315 | Lisinopril | Lisinopril Tab 5 MG |
| 65243030345 | Lisinopril | Lisinopril Tab 5 MG |
| 65243031403 | Lisinopril | Lisinopril Tab 40 MG |
| 65243031409 | Lisinopril | Lisinopril Tab 40 MG |
| 65243031418 | Lisinopril | Lisinopril Tab 40 MG |
| 65243034109 | Lisinopril | Lisinopril Tab 20 MG |
| 65243034118 | Lisinopril | Lisinopril Tab 20 MG |
| 66336074130 | Lisinopril | Lisinopril Tab 20 MG |
| 66336086730 | Lisinopril | Lisinopril Tab 40 MG |
| 66336097230 | Lisinopril | Lisinopril Tab 10 MG |
| 67544014815 | Lisinopril | Lisinopril Tab 40 MG |
| 67544014830 | Lisinopril | Lisinopril Tab 40 MG |
| 67544014845 | Lisinopril | Lisinopril Tab 40 MG |
| 67544014853 | Lisinopril | Lisinopril Tab 40 MG |
| 67544014860 | Lisinopril | Lisinopril Tab 40 MG |
| 67544015915 | Lisinopril | Lisinopril Tab 10 MG |
| 67544015930 | Lisinopril | Lisinopril Tab 10 MG |
| 67544015945 | Lisinopril | Lisinopril Tab 10 MG |
| 67544015958 | Lisinopril | Lisinopril Tab 10 MG |
| 67544015960 | Lisinopril | Lisinopril Tab 10 MG |
| 67544015980 | Lisinopril | Lisinopril Tab 10 MG |
| 67544017330 | Lisinopril | Lisinopril Tab 5 MG |
| 67544017345 | Lisinopril | Lisinopril Tab 5 MG |
| 67544017353 | Lisinopril | Lisinopril Tab 5 MG |
| 67544017360 | Lisinopril | Lisinopril Tab 5 MG |
| 67544017430 | Lisinopril | Lisinopril Tab 20 MG |
| 67544017445 | Lisinopril | Lisinopril Tab 20 MG |
| 67544017460 | Lisinopril | Lisinopril Tab 20 MG |
| 67544017480 | Lisinopril | Lisinopril Tab 20 MG |
| 67544006230 | Lisinopril | Lisinopril Tab 2.5 MG |
| 67544006260 | Lisinopril | Lisinopril Tab 2.5 MG |
| 67544019230 | Lisinopril | Lisinopril Tab 5 MG |
| 67544019245 | Lisinopril | Lisinopril Tab 5 MG |
| 67544019253 | Lisinopril | Lisinopril Tab 5 MG |
| 67544019260 | Lisinopril | Lisinopril Tab 5 MG |
| 67544021860 | Lisinopril | Lisinopril Tab 2.5 MG |
| 67544021915 | Lisinopril | Lisinopril Tab 10 MG |
| 67544021930 | Lisinopril | Lisinopril Tab 10 MG |
| 67544021945 | Lisinopril | Lisinopril Tab 10 MG |
| 67544021960 | Lisinopril | Lisinopril Tab 10 MG |
| 67544012815 | Lisinopril | Lisinopril Tab 5 MG |
| 67544012830 | Lisinopril | Lisinopril Tab 5 MG |
| 67544012845 | Lisinopril | Lisinopril Tab 5 MG |
| 67544012853 | Lisinopril | Lisinopril Tab 5 MG |
| 67544012860 | Lisinopril | Lisinopril Tab 5 MG |
| 67544013430 | Lisinopril | Lisinopril Tab 20 MG |
| 67544013445 | Lisinopril | Lisinopril Tab 20 MG |
| 67544013460 | Lisinopril | Lisinopril Tab 20 MG |
| 67544013480 | Lisinopril | Lisinopril Tab 20 MG |
| 68115020790 | Lisinopril | Lisinopril Tab 10 MG |
| 68084005801 | Lisinopril | Lisinopril Tab 2.5 MG |
| 68084005811 | Lisinopril | Lisinopril Tab 2.5 MG |
| 68084006001 | Lisinopril | Lisinopril Tab 5 MG |
| 68084006011 | Lisinopril | Lisinopril Tab 5 MG |
| 68084006101 | Lisinopril | Lisinopril Tab 10 MG |
| 68084006111 | Lisinopril | Lisinopril Tab 10 MG |
| 68084006201 | Lisinopril | Lisinopril Tab 20 MG |
| 68084006211 | Lisinopril | Lisinopril Tab 20 MG |
| 68084006401 | Lisinopril | Lisinopril Tab 40 MG |
| 68084006411 | Lisinopril | Lisinopril Tab 40 MG |
| 68180051201 | Lisinopril | Lisinopril Tab 2.5 MG |
| 68180051202 | Lisinopril | Lisinopril Tab 2.5 MG |
| 68180051301 | Lisinopril | Lisinopril Tab 5 MG |
| 68180051303 | Lisinopril | Lisinopril Tab 5 MG |
| 68180051401 | Lisinopril | Lisinopril Tab 10 MG |
| 68180051403 | Lisinopril | Lisinopril Tab 10 MG |
| 68180051501 | Lisinopril | Lisinopril Tab 20 MG |
| 68180051503 | Lisinopril | Lisinopril Tab 20 MG |
| 68180051601 | Lisinopril | Lisinopril Tab 30 MG |
| 68180051602 | Lisinopril | Lisinopril Tab 30 MG |
| 68180051701 | Lisinopril | Lisinopril Tab 40 MG |
| 68180051703 | Lisinopril | Lisinopril Tab 40 MG |
| 68258105801 | Lisinopril | Lisinopril Tab 10 MG |
| 68258105901 | Lisinopril | Lisinopril Tab 20 MG |
| 68258108001 | Lisinopril | Lisinopril Tab 40 MG |
| 00904581046 | Lisinopril | Lisinopril Tab 40 MG |
| 00904581048 | Lisinopril | Lisinopril Tab 40 MG |
| 00904581052 | Lisinopril | Lisinopril Tab 40 MG |
| 00904581089 | Lisinopril | Lisinopril Tab 40 MG |
| 00904581093 | Lisinopril | Lisinopril Tab 40 MG |
| 00904581289 | Lisinopril | Lisinopril Tab 2.5 MG |
| 68387054330 | Lisinopril | Lisinopril Tab 10 MG |
| 51079098130 | Lisinopril | Lisinopril Tab 5 MG |
| 51079098156 | Lisinopril | Lisinopril Tab 5 MG |
| 51079098230 | Lisinopril | Lisinopril Tab 10 MG |
| 51079098256 | Lisinopril | Lisinopril Tab 10 MG |
| 51079098330 | Lisinopril | Lisinopril Tab 20 MG |
| 51079098356 | Lisinopril | Lisinopril Tab 20 MG |
| 63874055890 | Lisinopril | Lisinopril Tab 5 MG |
| 63874061810 | Lisinopril | Lisinopril Tab 40 MG |
| 63874061815 | Lisinopril | Lisinopril Tab 40 MG |
| 63874061830 | Lisinopril | Lisinopril Tab 40 MG |
| 63874061840 | Lisinopril | Lisinopril Tab 40 MG |
| 63874061860 | Lisinopril | Lisinopril Tab 40 MG |
| 63874111409 | Lisinopril | Lisinopril Tab 30 MG |
| 21695032990 | Lisinopril | Lisinopril Tab 10 MG |
| 55887059101 | Lisinopril | Lisinopril Tab 10 MG |
| 55887059120 | Lisinopril | Lisinopril Tab 10 MG |
| 67544014880 | Lisinopril | Lisinopril Tab 40 MG |
| 67544099230 | Lisinopril | Lisinopril Tab 20 MG |
| 67544099730 | Lisinopril | Lisinopril Tab 40 MG |
| 67544099960 | Lisinopril | Lisinopril Tab 20 MG |
| 67544099980 | Lisinopril | Lisinopril Tab 20 MG |
| 00006001572 | Lisinopril | Lisinopril Tab 2.5 MG |
| 00006010686 | Lisinopril | Lisinopril Tab 10 MG |
| 00006020786 | Lisinopril | Lisinopril Tab 20 MG |
| 00038013010 | Lisinopril | Lisinopril Tab 5 MG |
| 00038013034 | Lisinopril | Lisinopril Tab 5 MG |
| 00038013039 | Lisinopril | Lisinopril Tab 5 MG |
| 00038013110 | Lisinopril | Lisinopril Tab 10 MG |
| 00038013134 | Lisinopril | Lisinopril Tab 10 MG |
| 00038013139 | Lisinopril | Lisinopril Tab 10 MG |
| 00038013210 | Lisinopril | Lisinopril Tab 20 MG |
| 00038013234 | Lisinopril | Lisinopril Tab 20 MG |
| 00038013239 | Lisinopril | Lisinopril Tab 20 MG |
| 00038013410 | Lisinopril | Lisinopril Tab 40 MG |
| 00038013439 | Lisinopril | Lisinopril Tab 40 MG |
| 40893065830 | Lisinopril | Lisinopril Tab 20 MG |
| 52246068030 | Lisinopril | Lisinopril Tab 10 MG |
| 52493065430 | Lisinopril | Lisinopril Tab 10 MG |
| 52493065830 | Lisinopril | Lisinopril Tab 20 MG |
| 53506071030 | Lisinopril | Lisinopril Tab 10 MG |
| 53506071330 | Lisinopril | Lisinopril Tab 20 MG |
| 54124056130 | Lisinopril | Lisinopril Tab 10 MG |
| 54124066830 | Lisinopril | Lisinopril Tab 20 MG |
| 54569205100 | Lisinopril | Lisinopril Tab 20 MG |
| 54569205102 | Lisinopril | Lisinopril Tab 20 MG |
| 54569266500 | Lisinopril | Lisinopril Tab 20 MG |
| 54569282900 | Lisinopril | Lisinopril Tab 40 MG |
| 54569175200 | Lisinopril | Lisinopril Tab 10 MG |
| 54569175201 | Lisinopril | Lisinopril Tab 10 MG |
| 54569175202 | Lisinopril | Lisinopril Tab 10 MG |
| 54569175204 | Lisinopril | Lisinopril Tab 10 MG |
| 54569175205 | Lisinopril | Lisinopril Tab 10 MG |
| 54569330002 | Lisinopril | Lisinopril Tab 5 MG |
| 54569330003 | Lisinopril | Lisinopril Tab 5 MG |
| 54569858300 | Lisinopril | Lisinopril Tab 10 MG |
| 54569858400 | Lisinopril | Lisinopril Tab 5 MG |
| 54569851500 | Lisinopril | Lisinopril Tab 10 MG |
| 54569852700 | Lisinopril | Lisinopril Tab 20 MG |
| 55175396403 | Lisinopril | Lisinopril Tab 10 MG |
| 58016113700 | Lisinopril | Lisinopril Tab 10 MG |
| 58016113714 | Lisinopril | Lisinopril Tab 10 MG |
| 58016113721 | Lisinopril | Lisinopril Tab 10 MG |
| 58016113728 | Lisinopril | Lisinopril Tab 10 MG |
| 58016113730 | Lisinopril | Lisinopril Tab 10 MG |
| 58016113760 | Lisinopril | Lisinopril Tab 10 MG |
| 49999029510 | Lisinopril | Lisinopril Tab 5 MG |
| 67544006282 | Lisinopril | Lisinopril Tab 2.5 MG |
| 54868465803 | Lisinopril | Lisinopril Tab 20 MG |
| 00904580843 | Lisinopril | Lisinopril Tab 10 MG |
| 00904580846 | Lisinopril | Lisinopril Tab 10 MG |
| 00904580889 | Lisinopril | Lisinopril Tab 10 MG |
| 00904580893 | Lisinopril | Lisinopril Tab 10 MG |
| 00904581043 | Lisinopril | Lisinopril Tab 40 MG |
| 00904581143 | Lisinopril | Lisinopril Tab 5 MG |
| 00904581146 | Lisinopril | Lisinopril Tab 5 MG |
| 00904581189 | Lisinopril | Lisinopril Tab 5 MG |
| 23490581500 | Lisinopril | Lisinopril Tab 10 MG |
| 23490581606 | Lisinopril | Lisinopril Tab 20 MG |
| 18837027730 | Lisinopril | Lisinopril Tab 5 MG |
| 33358021130 | Lisinopril | Lisinopril Tab 5 MG |
| 33358021230 | Lisinopril | Lisinopril Tab 10 MG |
| 33358021330 | Lisinopril | Lisinopril Tab 20 MG |
| 65243035803 | Lisinopril | Lisinopril Tab 10 MG |
| 00172375700 | Lisinopril | Lisinopril Tab 2.5 MG |
| 00172376200 | Lisinopril | Lisinopril Tab 30 MG |
| 55289091730 | Lisinopril | Lisinopril Tab 40 MG |
| 00440767599 | Lisinopril | Lisinopril Tab 10 MG |
| 00904580861 | Lisinopril | Lisinopril Tab 10 MG |
| 00904580961 | Lisinopril | Lisinopril Tab 20 MG |
| 00904581061 | Lisinopril | Lisinopril Tab 40 MG |
| 00904581161 | Lisinopril | Lisinopril Tab 5 MG |
| 65243031445 | Lisinopril | Lisinopril Tab 40 MG |
| 57866870001 | Lisinopril | Lisinopril Tab 40 MG |
| 00904580848 | Lisinopril | Lisinopril Tab 10 MG |
| 00904580943 | Lisinopril | Lisinopril Tab 20 MG |
| 00904580946 | Lisinopril | Lisinopril Tab 20 MG |
| 00904580948 | Lisinopril | Lisinopril Tab 20 MG |
| 00904580989 | Lisinopril | Lisinopril Tab 20 MG |
| 00904580993 | Lisinopril | Lisinopril Tab 20 MG |
| 21695033090 | Lisinopril | Lisinopril Tab 20 MG |
| 54458099410 | Lisinopril | Lisinopril Tab 40 MG |
| 54458099510 | Lisinopril | Lisinopril Tab 30 MG |
| 54458099610 | Lisinopril | Lisinopril Tab 20 MG |
| 54458099710 | Lisinopril | Lisinopril Tab 10 MG |
| 54458099809 | Lisinopril | Lisinopril Tab 5 MG |
| 54458099909 | Lisinopril | Lisinopril Tab 2.5 MG |
| 54569543404 | Lisinopril | Lisinopril Tab 10 MG |
| 54569543504 | Lisinopril | Lisinopril Tab 20 MG |
| 65243034145 | Lisinopril | Lisinopril Tab 20 MG |
| 64679092709 | Lisinopril | Lisinopril Tab 2.5 MG |
| 64679092810 | Lisinopril | Lisinopril Tab 5 MG |
| 64679092910 | Lisinopril | Lisinopril Tab 10 MG |
| 64679094110 | Lisinopril | Lisinopril Tab 20 MG |
| 64679094209 | Lisinopril | Lisinopril Tab 40 MG |
| 64679095309 | Lisinopril | Lisinopril Tab 30 MG |
| 00143126509 | Lisinopril | Lisinopril Tab 2.5 MG |
| 00143126609 | Lisinopril | Lisinopril Tab 5 MG |
| 00143126630 | Lisinopril | Lisinopril Tab 5 MG |
| 00143126645 | Lisinopril | Lisinopril Tab 5 MG |
| 00143126709 | Lisinopril | Lisinopril Tab 10 MG |
| 00143126718 | Lisinopril | Lisinopril Tab 10 MG |
| 00143126730 | Lisinopril | Lisinopril Tab 10 MG |
| 00143126745 | Lisinopril | Lisinopril Tab 10 MG |
| 00143126809 | Lisinopril | Lisinopril Tab 20 MG |
| 00143126818 | Lisinopril | Lisinopril Tab 20 MG |
| 00143126830 | Lisinopril | Lisinopril Tab 20 MG |
| 00143126845 | Lisinopril | Lisinopril Tab 20 MG |
| 00143127009 | Lisinopril | Lisinopril Tab 40 MG |
| 00143127018 | Lisinopril | Lisinopril Tab 40 MG |
| 00143127030 | Lisinopril | Lisinopril Tab 40 MG |
| 00143127045 | Lisinopril | Lisinopril Tab 40 MG |
| 00904580880 | Lisinopril | Lisinopril Tab 10 MG |
| 00904580980 | Lisinopril | Lisinopril Tab 20 MG |
| 00904581080 | Lisinopril | Lisinopril Tab 40 MG |
| 00904581180 | Lisinopril | Lisinopril Tab 5 MG |
| 00904581240 | Lisinopril | Lisinopril Tab 2.5 MG |
| 49999018210 | Lisinopril | Lisinopril Tab 10 MG |
| 67544021882 | Lisinopril | Lisinopril Tab 2.5 MG |
| 00440767490 | Lisinopril | Lisinopril Tab 5 MG |
| 00440767530 | Lisinopril | Lisinopril Tab 10 MG |
| 00440767614 | Lisinopril | Lisinopril Tab 20 MG |
| 00440767630 | Lisinopril | Lisinopril Tab 20 MG |
| 00440767645 | Lisinopril | Lisinopril Tab 20 MG |
| 00440767790 | Lisinopril | Lisinopril Tab 40 MG |
| 66336074190 | Lisinopril | Lisinopril Tab 20 MG |
| 66336086790 | Lisinopril | Lisinopril Tab 40 MG |
| 54868467802 | Lisinopril | Lisinopril Tab 5 MG |
| 55289088430 | Lisinopril | Lisinopril Tab 5 MG |
| 60429072801 | Lisinopril | Lisinopril Tab 2.5 MG |
| 60429072810 | Lisinopril | Lisinopril Tab 2.5 MG |
| 60429072901 | Lisinopril | Lisinopril Tab 5 MG |
| 60429072910 | Lisinopril | Lisinopril Tab 5 MG |
| 60429073001 | Lisinopril | Lisinopril Tab 10 MG |
| 60429073010 | Lisinopril | Lisinopril Tab 10 MG |
| 60429073101 | Lisinopril | Lisinopril Tab 20 MG |
| 60429073110 | Lisinopril | Lisinopril Tab 20 MG |
| 60429073301 | Lisinopril | Lisinopril Tab 40 MG |
| 65243035809 | Lisinopril | Lisinopril Tab 10 MG |
| 65243035845 | Lisinopril | Lisinopril Tab 10 MG |
| 60429073310 | Lisinopril | Lisinopril Tab 40 MG |
| 66336097290 | Lisinopril | Lisinopril Tab 10 MG |
| 67544134845 | Lisinopril | Lisinopril Tab 10 MG |
| 67544134860 | Lisinopril | Lisinopril Tab 10 MG |
| 67544135045 | Lisinopril | Lisinopril Tab 40 MG |
| 67544135060 | Lisinopril | Lisinopril Tab 40 MG |
| 67544137245 | Lisinopril | Lisinopril Tab 5 MG |
| 49999018390 | Lisinopril | Lisinopril Tab 20 MG |
| 49999046960 | Lisinopril | Lisinopril Tab 40 MG |
| 49999046990 | Lisinopril | Lisinopril Tab 40 MG |
| 43353035060 | Lisinopril | Lisinopril Tab 40 MG |
| 43353036515 | Lisinopril | Lisinopril Tab 5 MG |
| 43353036530 | Lisinopril | Lisinopril Tab 5 MG |
| 43353036545 | Lisinopril | Lisinopril Tab 5 MG |
| 43353036560 | Lisinopril | Lisinopril Tab 5 MG |
| 43353037145 | Lisinopril | Lisinopril Tab 20 MG |
| 43353037160 | Lisinopril | Lisinopril Tab 20 MG |
| 52959072990 | Lisinopril | Lisinopril Tab 20 MG |
| 60429072830 | Lisinopril | Lisinopril Tab 2.5 MG |
| 60429072930 | Lisinopril | Lisinopril Tab 5 MG |
| 60429073030 | Lisinopril | Lisinopril Tab 10 MG |
[truncated: 316,322 more chars]
